# Identification of Blood Pressure‐Associated Metabolites by Integrating Metabolomic and Genetic Analysis

**DOI:** 10.1002/mco2.70718

**Published:** 2026-04-01

**Authors:** Yuanjiao Liu, Chunxiao Cheng, Xiong‐Fei Pan, Wei Shao, Dan Zhou, Yimin Zhu

**Affiliations:** ^1^ Department of Respiratory Disease Sir Run Run Shaw Hospital and Department of Epidemiology and Biostatistics Zhejiang University School of Medicine Hangzhou Zhejiang China; ^2^ The Second Affiliated Hospital and School of Public Health Zhejiang University School of Medicine Hangzhou China; ^3^ The Key Laboratory of Intelligent Preventive Medicine of Zhejiang Province Hangzhou Zhejiang China; ^4^ Section of Epidemiology and Population Health & Department of Gynecology and Obstetrics Ministry of Education Key Laboratory of Birth Defects and Related Diseases of Women and Children & Children's Medicine Key Laboratory of Sichuan Province West China Second University Hospital Sichuan University Chengdu China; ^5^ West China Biomedical Big Data Center West China Hospital Sichuan University Chengdu China; ^6^ Shuangliu Institute of Women's and Children's Health Shuangliu Maternal and Child Health Hospital Chengdu China; ^7^ Department of Internal Medicine Putuo District People's Hospital Zhoushan Zhejiang China; ^8^ State Key Laboratory of Transvascular Implantation Devices Hangzhou Zhejiang China

**Keywords:** blood pressure, metabolites, metabolite genome‐wide association study, Mendelian randomization

## Abstract

This study aimed to identify blood pressure‐associated metabolites and explore their underlying pathways using multiomics data from 1188 Chinese participants. Serum metabolite levels were profiled using untargeted and widely targeted metabolomic technologies. The associations of metabolites as well as ratios with blood pressure were assessed using generalized linear models (GLM). Targeted metabolomics was used to replicate a subset of metabolites. Genome‐wide association studies (GWAS) were performed on all metabolites identified. Potential causality was examined using two‐sample Mendelian randomization (MR) analyses, with partial validation against GWAS results from an independent cohort. This study identified 10 blood pressure‐associated metabolites supported by GLM and MR analyses. Cortisol demonstrated the strongest association with blood pressure, with l‐glutamic acid and its ratios identified as key drivers. Multiomics integration revealed that a genetic variant near the omega‐3 metabolism genes (*FADS1*/*FADS2*) may influence blood pressure regulation by modulating prostaglandin E3 levels. Mediation analysis indicated that l‐glutamic acid statistically mediated 12.16–31.53% of the effect of lifestyle factors on blood pressure. These findings enhance our understanding of metabolic mechanisms underlying hypertension and highlight potential biomarkers and therapeutic targets for further investigation.

## Graphical abstract

This study identified 10 potential causality between metabolites and blood pressure and found potential genetic regulators of two metabolites. Multiomics integration revealed that a genetic variant near the omega‐3 metabolism genes (*FADS1*/*FADS2*) may influence blood pressure regulation by modulating prostaglandin E3 levels. The mediating effects of lifestyle–glutamic acid–blood pressure were also explored. These findings provide novel insights into metabolic mechanisms and genetic regulation underlying hypertension.

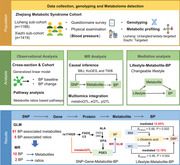

## 1 Introduction

Elevated blood pressure is a crucial risk factor for a range of chronic diseases such as cardiovascular disease, chronic kidney disease, and dementia [1]. According to the latest data, the global age‐standardized prevalence of hypertension in adults aged 30–79 years in 2019 was 32% in women and 34% in men [2]. Among American adults, the age‐standardized prevalence of diagnosed hypertension was approximately 30% during 2017–2021 [3]. In China, a national survey of 642,523 adults reported a standardized prevalence of hypertension of 24.7% in 2018 [4]. As the prevalence of hypertension continues to rise in the coming decades, hypertension poses an increasing public health challenge [5]. Therefore, it is paramount to deepen our understanding of its pathogenesis and to identify novel therapeutic strategies.

Metabolites comprise a broad range of biochemical compound including sugars, lipids, amino acids, and lipids. These metabolites participate in fundamental biological processes including energy metabolism, cell signaling, and oxidative stress responses, thereby serving as sensitive indicators of physiological and pathological states. Metabolomics provides insights into disease mechanisms and specific biological conditions [6]. This technology holds great promise for identifying metabolites that could enhance the diagnosis and prognosis of diseases [7]. Numerous studies have implicated various metabolites and metabolic pathways in the regulation of blood pressure and the pathogenesis of hypertension [8, 9]. For example, the Bogalusa Heart Study (BHS), involving European 1249 participants, identified 24 metabolites significantly associated with blood pressure through untargeted metabolomics, which detected 1466 metabolites [10]. Similarly, the TwinUK study of 3980 European women utilized untargeted metabolomics and found that hexadecanedioate exhibited a consistent association with blood pressure and mortality [11]. While prior research, primarily in European populations, has identified numerous metabolites associated with blood pressure regulation, findings may not be fully generalizable to other ethnic groups due to substantial differences in genetic background, diet, and lifestyle. Recent Chinese studies have identified various metabolites associated with blood pressure phenotypes. For instance, a case–control study conducted in Chengdu, China, identified oleic acid and myoinositol as the most significant differential metabolites between hypertensive patients and controls [12]. However, studies focusing on Chinese populations have been relatively limited, with most involving relatively small sample sizes and limited metabolite detection, thereby hindering a comprehensive understanding of hypertension's pathogenesis in Chinese [12, 13, 14, 15, 16, 17, 18].

Compared with traditional observational analysis, Mendelian randomization (MR) analysis mitigates the effects of potential confounding and reverse causality by utilizing genetic variations as instrumental variables (IVs) [19]. Several studies based on MR analyses have reported potential causal associations between blood pressure and metabolites, primarily in European populations [20, 21, 22]. Based on the lipidomics, genetically predicted levels of sphingomyelin (OH) C32:2 were inversely associated with both systolic blood pressure (SBP) and diastolic blood pressure (DBP) in Chinese [23]. However, the potential causal role of metabolites in hypertension within Chinese cohorts remains underexplored. Furthermore, integrating multiomics data could significantly deepen our understanding of the molecular pathways underlying blood pressure regulation.

A substantial body of evidence has consistently demonstrated that healthy lifestyle is significantly associated with both increased longevity and reduced incidence of major chronic diseases [24, 25, 26]. Multiple previous studies have uncovered metabolites related to healthy lifestyle, and obesity, and dietary approaches for hypertension prevention, which are recognized as key factors of hypertension [27, 28, 29, 30]. For example, a case–control study involving children from South west China identified phosphatidylglycerol (43:6) and phosphatidylcholine (18:0/20:3) as mediating variables between nut intake and blood pressure levels [31]. However, large‐scale population studies that systematically examine the mediating roles of metabolites in the relationship between comprehensive lifestyle factors and blood pressure were limited.

Therefore, this study aims to systematically explore the metabolic underpinnings of blood pressure in Chinese adults. We identified blood pressure‐associated metabolites and integrating multiomics evidence to pinpoint potential molecular mechanisms. Finally, we conducted mediation analysis to evaluate the role of identified metabolites in mediating key lifestyle factors to blood pressure, thereby offering novel insights into the prevention of hypertension in Chinese population.

## 2 Results

### 2.1 Overview

**FIGURE 1:**
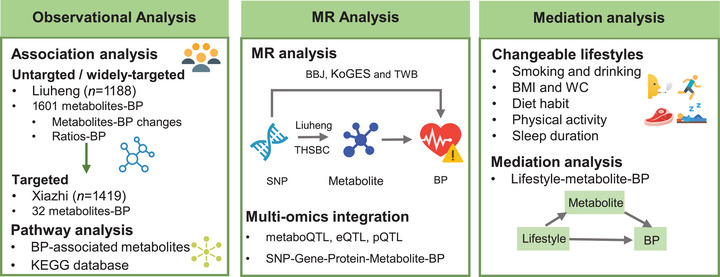
Flowchart of the study design. BBJ, the BioBank Japan; BMI, body mass index; BP, blood pressure; CVD, cardiovascular disease; GWAS, genome‐wide association studies; KEGG, the Kyoto Encyclopedia of Genes and Genomes; KoGES, the Korean Genome and Epidemiology Study; MR analysis, Mendelian randomization analysis; SNP, single nucleotide polymorphisms; THSBC, the Tongji‐Huaxi‐Shuangliu Birth Cohort; TWB, the Taiwan Biobank; WC, waist circumference.

Figure 1 illustrates the study design. A total of 1188 participants with untargeted and widely targeted metabolomic profiling were derived from the Liuheng sub‐cohort of the Zhejiang Metabolic Syndrome Cohort (ZMSC). Xiazhi sub‐cohort were included as the replication for available targeted metabolites. First, we analyzed the associations between metabolites and baseline blood pressure. Based on these blood pressure‐associated metabolites, we further investigated metabolites linked to changes in blood pressure and examined the associations between the identified relevant metabolite ratios and blood pressure levels. Next, we performed metabolome‐based genome‐wide association study (metabo‐GWAS) to explore the genetic determinants of metabolites, with the Liuheng sub‐cohort serving as the discovery set and the Tongji‐Huaxi‐Shuangliu Birth Cohort (THSBC) used for validation. Then, two‐sample MR analyses were conducted to investigate potential causal relationships between metabolites and blood pressure by using public GWAS summary data of blood pressure‐related traits from the BioBank Japan (BBJ), the Korean Genome and Epidemiology Study (KoGES), and the Taiwan Biobank (TWB). Furthermore, multiomics integration was employed to elucidate the underlying molecular mechanisms. Finally, mediation analysis was conducted to assess the potential mediating effects of metabolites in the association between modifiable lifestyles and blood pressure.

### 2.2 Characteristics of the Study Participants

A total of 1188 individuals (370 males and 818 females) were included in this study from Liuheng sub‐cohort (Table 1). The mean age was 58.8 (9.3) years. The proportion of smoking and drinking was 21.1 and 22.6%, respectively. The hypertension prevalence was 54.6%. A total of 166 incident hypertension cases (46 males, 120 females) were identified over a 5‐year follow‐up period among 1160 individuals.

**TABLE 1:** Basic characteristics of the study participants.

Variable	Overall	Male	Female
*N*	1188	370	818
Age, mean (SD)	58.8 (9.3)	61.4 (9.9)	57.6 (8.8)
Smoking, *n* (%)	251 (21.1)	251 (67.8)	0 (0.0)
Drinking, *n* (%)	268 (22.6)	250 (67.6)	18 (2.2)
BMI, mean (SD)	23.53 (3.30)	23.65 (3.19)	23.48 (3.35)
WC, mean (SD)	78.74 (9.42)	81.44 (9.20)	77.51 (9.26)
FPG, mean (SD)	5.63 (0.89)	5.65 (0.74)	5.63 (0.95)
TG, mean (SD)	1.49 (0.98)	1.36 (0.99)	1.55 (0.98)
HDLC, mean (SD)	1.28 (0.27)	1.27 (0.29)	1.28 (0.26)
SBP, mean (SD)	137.6 (19.9)	137.6 (19.8)	137.6 (20.0)
DBP, mean (SD)	82.4 (10.7)	81.8 (10.5)	82.6 (10.8)
Prevalent hypertension, *n* (%)	649 (54.6)	218 (58.9)	431 (52.7)
Baseline antihypertensive drug, *n* (%)	278 (23.4)	107 (28.9)	171 (20.9)
Incident hypertension, *n* (%)^a^, ^b^	166 (14.3)	46 (12.7)	120 (15.0)
Follow‐up antihypertensive drug, *n* (%)^a^	418 (36.0)	150 (41.6)	268 (33.5)

*Notes*: Data are presented as mean (standard deviation [SD]) or number (percentage).

Abbreviations: BMI, body mass index; WC, waist circumference; FPG, fasting plasma glucose; TG, triglyceride; HDLC, high density lipoprotein cholesterol; SBP, systolic blood pressure; DBP, diastolic blood pressure.

^a^ The 2020 follow‐up included 1160 individuals from the 2015 baseline study, comprising 361 males and 799 females.

^b^ The 5‐year cumulative incidence was determined using the formula: (number of new cases)/(number of individuals in the follow‐up population).

### 2.3 Metabolic Profiling

A total of 1601 serum metabolites were identified and quantified in this study spanning 23 major classes (Figure S1 and Table S1) using widely targeted metabolomic approaches. The most abundant class of metabolites were amino acids and their derivatives, followed by benzene derivatives.

### 2.4 Blood Pressure‐Associated Metabolites

A total of 91 metabolites (false discovery rate [FDR]‐adjusted *p* < 0.05 for any blood pressure trait) were identified as robustly associated with blood pressure traits, including 37 metabolites for SBP, 81 for DBP, and nine for hypertension (Table 2 and Figures S2 and S3). Notably, six metabolites were associated with all three blood pressure traits, 24 were associated with two blood pressure traits, and 61 were associated with a single blood pressure trait. These associations remained consistent both in analyses including participants taking antihypertensive drugs and in sex‐stratified analyses (Tables S4 and S5).

**TABLE 2:** Blood pressure‐associated metabolites in generalized linear models.

Metabolites	Class	Beta(95%CI) for SBP	*p* for SBP	FDR adjusted *p* for SBP	Beta(95%CI) for DBP	*p* for DBP	FDR adjusted *p* for DBP	OR(95%CI) for hypertension	*p* for hypertension	FDR adjusted *p* for hypertension
Lythramine	Alcohol and amines	0.86(−0.25–1.98)	1.30e−01	4.12e−01	1.02(0.37–1.66)	2.04e−03	**3.23e**−**02**	1.03(0.89–1.19)	7.38e−01	9.34e−01
Bis(1‐inositol)‐3,1′‐phosphate 1‐phosphate	Alcohol and amines	1.77(0.65–2.88)	1.95e−03	5.05e−02	1.29(0.64–1.93)	9.55e−05	**4.25e**−**03**	1.18(1.02–1.36)	2.90e−02	4.77e−01
Perseitol	Alcohol and amines	−1.69(−2.81–0.57)	3.19e−03	6.52e−02	−1.23(−1.88–0.58)	2.20e−04	**7.76e**−**03**	0.85(0.74–0.99)	3.74e−02	4.94e−01
Cucurbitacin B	Aldehyde, ketones, and esters	−1.53(−2.68–0.39)	8.48e−03	1.24e−01	−1.18(−1.84–0.52)	4.68e−04	**1.34e**−**02**	0.95(0.82–1.10)	4.68e−01	8.39e−01
Butylate	Aldehyde, ketones, and esters	−1.90(−2.98–0.83)	5.47e−04	**2.74e−02**	−0.67(−1.29–0.04)	3.70e−02	2.01e−01	0.84(0.73–0.97)	1.50e−02	4.77e−01
Methyl reserpate	Alkaloids	2.44(1.14–3.74)	2.52e−04	**1.77e−02**	1.37(0.61–2.12)	3.93e−04	**1.19e**−**02**	1.24(1.05–1.47)	1.32e−02	4.70e−01
l‐Glutamic acid	Amino acid and its metabolites	0.86(−0.35–2.07)	1.65e−01	4.68e−01	1.19(0.49–1.89)	8.68e−04	**1.96e**−**02**	1.09(0.93–1.27)	3.11e−01	7.88e−01
Asp–phe	Amino acid and its metabolites	0.57(−0.56–1.71)	3.23e−01	6.37e−01	1.10(0.44–1.76)	1.05e−03	**2.17e**−**02**	1.05(0.90–1.21)	5.38e−01	8.58e−01
N‐lactoyl‐phenylalanine	Amino acid and its metabolites	3.06(1.88–4.25)	4.92e−07	**3.94e−04**	2.00(1.31–2.69)	1.47e−08	**1.18e**−**05**	1.37(1.17–1.61)	1.21e−04	**3.88e**−**02**
Nα‐acetyl‐l‐glutamine	Amino acid and its metabolites	2.09(0.93–3.25)	4.31e−04	**2.47e−02**	0.97(0.29–1.64)	5.06e−03	5.85e−02	1.19(1.02–1.38)	2.96e−02	4.77e−01
N,N‐dimethylarginine	Amino acid and its metabolites	2.06(0.90–3.23)	5.42e−04	**2.74e−02**	0.62(−0.06–1.29)	7.59e−02	2.97e−01	1.25(1.07–1.46)	4.08e−03	2.84e−01
3‐Hydroxyanthranilic acid	Benzene and substituted derivatives	1.29(0.13–2.44)	2.89e−02	2.28e−01	1.16(0.49–1.82)	6.89e−04	**1.69e**−**02**	1.16(1.00–1.35)	5.13e−02	5.48e−01
3‐Amino‐4‐hydroxybenzoic acid	Benzene and substituted derivatives	1.29(0.13–2.44)	2.89e−02	2.28e−01	1.16(0.49–1.82)	6.89e−04	**1.69e**−**02**	1.16(1.00–1.35)	5.13e−02	5.48e−01
4‐Acetoxyphenol	Benzene and substituted derivatives	0.82(−0.34–1.99)	1.65e−01	4.68e−01	1.05(0.37–1.72)	2.31e−03	**3.56e**−**02**	1.09(0.94–1.26)	2.73e−01	7.73e−01
CP 47,497‐C8‐homolog C‐8‐hydroxy metabolite	Benzene and substituted derivatives	1.53(0.42–2.65)	7.03e−03	1.14e−01	0.99(0.34–1.63)	2.74e−03	**3.95e**−**02**	1.16(1.00–1.35)	4.38e−02	5.12e−01
6,6′‐(1,2‐Phenylene)bis(1,3,5‐triazine‐2,4‐diamine)	Benzene and substituted derivatives	1.79(0.68–2.90)	1.60e−03	**4.70e−02**	0.70(0.06–1.35)	3.32e−02	1.91e−01	1.23(1.06–1.42)	6.48e−03	3.38e−01
Melibiose	Carbohydrates and Its metabolites	−1.38(−2.48–0.28)	1.43e−02	1.64e−01	−0.97(−1.61–0.33)	3.10e−03	**4.27e**−**02**	0.89(0.77–1.02)	9.79e−02	6.26e−01
N‐acetyl‐d‐lactosamine	Carbohydrates and Its metabolites	2.50(1.41–3.59)	7.63e−06	**2.15e−03**	1.03(0.40–1.67)	1.48e−03	**2.79e**−**02**	1.33(1.15–1.54)	1.59e−04	**4.06e**−**02**
Lactitol	Carbohydrates and Its metabolites	1.90(0.77–3.03)	9.94e−04	**4.02e−02**	0.80(0.14–1.45)	1.78e−02	1.28e−01	1.18(1.02–1.37)	3.10e−02	4.77e−01
FFA(18:4)	FA	2.02(0.88–3.16)	5.30e−04	**2.74e−02**	1.46(0.80–2.12)	1.56e−05	**1.39e**−**03**	1.14(0.98–1.32)	9.12e−02	6.21e−01
FFA(17:1)	FA	0.66(−0.45–1.77)	2.45e−01	5.63e−01	0.97(0.33–1.61)	3.10e−03	**4.27e**−**02**	1.09(0.94–1.26)	2.49e−01	7.71e−01
FFA(16:2)	FA	1.86(0.75–2.97)	1.01e−03	**4.02e−02**	1.30(0.66–1.94)	7.34e−05	**3.67e**−**03**	1.14(0.98–1.31)	8.56e−02	6.21e−01
FFA(14:1)	FA	0.78(−0.37–1.92)	1.83e−01	4.88e−01	0.98(0.32–1.64)	3.67e−03	**4.59e**−**02**	1.10(0.95–1.28)	2.01e−01	7.47e−01
Carnitine C20:5	FA	1.82(0.63–3.00)	2.71e−03	6.04e−02	1.30(0.62–1.99)	2.06e−04	**7.48e**−**03**	1.18(1.01–1.38)	3.64e−02	4.94e−01
Carnitine C16:1	FA	1.87(0.72–3.02)	1.45e−03	**4.70e−02**	0.70(0.03–1.37)	3.96e−02	2.10e−01	1.20(1.03–1.39)	1.81e−02	4.77e−01
Carnitine C16‐OH	FA	2.10(0.95–3.25)	3.56e−04	**2.11e−02**	1.00(0.33–1.67)	3.49e−03	**4.40e**−**02**	1.33(1.15–1.55)	2.17e−04	**4.35e**−**02**
Bovinic acid	FA	2.44(1.30–3.57)	2.81e−05	**3.87e−03**	1.46(0.81–2.12)	1.42e−05	**1.34e**−**03**	1.23(1.06–1.43)	7.22e−03	3.38e−01
13‐Tetradecynoic acid	FA	1.79(0.67–2.90)	1.67e−03	**4.70e−02**	0.79(0.14–1.44)	1.71e−02	1.25e−01	1.11(0.96–1.29)	1.53e−01	7.13e−01
5,8,11‐Eicosatrienoic acid	FA	1.32(0.19–2.44)	2.16e−02	2.05e−01	1.03(0.38–1.68)	1.96e−03	**3.23e**−**02**	1.06(0.91–1.22)	4.50e−01	8.36e−01
Ethyl palmitoleate	FA	1.97(0.85–3.10)	5.84e−04	**2.75e−02**	1.06(0.41–1.71)	1.47e−03	**2.79e**−**02**	1.22(1.05–1.42)	8.24e−03	3.38e−01
MG(0:0/22:6/0:0)	GL	1.29(0.13–2.46)	2.94e−02	2.28e−01	1.22(0.55–1.89)	3.81e−04	**1.19e**−**02**	1.14(0.98–1.32)	8.84e−02	6.21e−01
MG(22:6/0:0/0:0)	GL	1.29(0.13–2.46)	2.94e−02	2.28e−01	1.22(0.55–1.89)	3.81e−04	**1.19e**−**02**	1.14(0.98–1.32)	8.84e−02	6.21e−01
LPE(14:0/0:0)	GP	1.56(0.44–2.69)	6.45e−03	1.05e−01	1.25(0.60–1.90)	1.71e−04	**6.38e**−**03**	1.10(0.95–1.28)	1.98e−01	7.47e−01
LPA(0:0/18:0)	GP	1.03(−0.06–2.13)	6.51e−02	3.16e−01	1.08(0.44–1.71)	8.99e−04	**1.97e**−**02**	1.11(0.96–1.28)	1.70e−01	7.13e−01
LPA(0:0/16:0)	GP	1.59(0.50–2.69)	4.45e−03	8.48e−02	1.25(0.62–1.89)	1.09e−04	**4.49e**−**03**	1.12(0.97–1.29)	1.35e−01	6.88e−01
LPE(0:0/16:1)	GP	1.06(−0.08–2.19)	6.90e−02	3.20e−01	1.01(0.36–1.67)	2.55e−03	**3.74e**−**02**	1.05(0.90–1.21)	5.49e−01	8.58e−01
LPA(16:0/0:0)	GP	0.96(−0.14–2.06)	8.83e−02	3.47e−01	1.05(0.42–1.69)	1.22e−03	**2.51e**−**02**	1.07(0.93–1.24)	3.34e−01	7.98e−01
LPG(16:0)	GP	1.38(0.26–2.49)	1.56e−02	1.70e−01	1.16(0.52–1.81)	4.33e−04	**1.28e**−**02**	1.06(0.92–1.23)	4.40e−01	8.34e−01
LPA(22:6)	GP	1.19(0.09–2.29)	3.40e−02	2.48e−01	0.97(0.33–1.61)	2.88e−03	**4.11e**−**02**	1.09(0.95–1.26)	2.33e−01	7.60e−01
LPC(0:0/14:0)	GP	2.05(0.93–3.17)	3.30e−04	**2.11e−02**	1.71(1.07–2.36)	2.11e−07	**1.12e**−**04**	1.15(0.99–1.33)	6.09e−02	5.78e−01
PC(12:0/12:0)	GP	1.24(0.13–2.36)	2.90e−02	2.28e−01	1.03(0.38–1.67)	1.84e−03	**3.09e**−**02**	1.07(0.92–1.24)	3.71e−01	8.16e−01
LPC(22:5/0:0)	GP	1.21(0.10–2.31)	3.26e−02	2.45e−01	1.03(0.39–1.67)	1.56e−03	**2.90e**−**02**	1.06(0.92–1.22)	4.36e−01	8.34e−01
LPC(0:0/20:3)	GP	1.27(0.16–2.38)	2.47e−02	2.17e−01	1.04(0.40–1.68)	1.47e−03	**2.79e**−**02**	1.10(0.95–1.28)	1.87e−01	7.38e−01
LPC(20:3/0:0)	GP	1.38(0.27–2.49)	1.52e−02	1.66e−01	1.12(0.48–1.76)	6.63e−04	**1.69e**−**02**	1.11(0.96–1.29)	1.62e−01	7.13e−01
LPC(18:0/0:0)	GP	1.22(0.12–2.32)	2.94e−02	2.28e−01	1.22(0.59–1.86)	1.67e−04	**6.37e**−**03**	1.04(0.90–1.19)	6.32e−01	8.92e−01
LPC(0:0/16:0)	GP	1.71(0.61–2.82)	2.40e−03	5.67e−02	1.33(0.69–1.96)	5.01e−05	**2.97e**−**03**	1.09(0.95–1.26)	2.23e−01	7.60e−01
LPC(12:0/0:0)	GP	1.24(0.15–2.33)	2.61e−02	2.22e−01	0.99(0.35–1.62)	2.26e−03	**3.51e**−**02**	1.10(0.95–1.27)	2.02e−01	7.47e−01
LPC(0:0/18:0)	GP	1.18(0.08–2.28)	3.51e−02	2.49e−01	1.12(0.49–1.76)	5.56e−04	**1.53e**−**02**	1.03(0.89–1.19)	6.87e−01	9.18e−01
PC(O‐1:0/O‐16:0)	GP	1.71(0.60–2.81)	2.60e−03	6.00e−02	1.41(0.77–2.05)	1.69e−05	**1.40e**−**03**	1.10(0.95–1.27)	2.13e−01	7.54e−01
2,3‐Dihydroxypropyl 2‐[(octadec‐9‐enoyl)amino]ethyl hydrogen phosphate	GP	0.85(−0.26–1.96)	1.33e−01	4.16e−01	0.95(0.31–1.59)	3.74e−03	**4.63e**−**02**	1.05(0.91–1.21)	5.20e−01	8.54e−01
Glycerophospho‐N‐palmitoyl ethanolamine	GP	1.64(0.53–2.76)	3.91e−03	7.63e−02	1.43(0.79–2.07)	1.38e−05	**1.34e**−**03**	1.13(0.98–1.31)	9.56e−02	6.24e−01
1,2‐Dihexanoyl‐sn‐glycero‐3‐phosphocholine	GP	−2.10(−3.22–0.98)	2.54e−04	**1.77e−02**	−1.38(−2.03–0.73)	3.38e−05	**2.09e**−**03**	0.90(0.77–1.04)	1.48e−01	7.13e−01
1‐Oleoyl lysophosphatidic acid sodium salt	GP	1.44(0.34–2.55)	1.05e−02	1.39e−01	1.21(0.57−1.84)	2.25e−04	**7.76e**−**03**	1.11(0.96–1.29)	1.47e−01	7.13e−01
Hydroxypiperazic acid	Heterocyclic compounds	−1.06(−2.19–0.07)	6.47e−02	3.16e−01	−0.83(−1.48–0.17)	1.31e−02	1.09e−01	0.75(0.65–0.87)	1.77e−04	**4.06e**−**02**
Imidazoleacetic acid	Heterocyclic compounds	2.52(1.39–3.64)	1.29e−05	**2.45e−03**	1.39(0.74–2.05)	3.23e−05	**2.09e**−**03**	1.35(1.16–1.57)	1.15e−04	**3.88e**−**02**
4‐Amino‐5‐hydroxymethyl‐2‐methylpyrimidine	Heterocyclic compounds	2.81(1.67–3.96)	1.77e−06	**9.43e−04**	1.34(0.67–2.01)	9.26e−05	**4.25e**−**03**	1.33(1.14–1.55)	3.09e−04	**4.95e**−**02**
(1R,2R,5R,8R,9S,10R,12S)‐12‐Hydroxy‐11‐methyl‐6‐methylidene‐16‐oxo‐15‐oxapentacyclo[9.3.2.15,8.01,10.02,8]heptadecane‐9‐carboxylic acid	Heterocyclic compounds	1.42(0.30–2.53)	1.29e−02	1.57e−01	1.33(0.69–1.98)	5.38e−05	**2.97e**−**03**	1.08(0.94–1.25)	2.84e−01	7.73e−01
Foetidin	Heterocyclic compounds	2.12(1.03–3.22)	1.50e−04	**1.34e−02**	1.45(0.81–2.08)	8.51e−06	**9.73e**−**04**	1.16(1.01–1.34)	4.21e−02	5.10e−01
Cortisol	Hormones and hormone related compounds	3.71(2.61–4.81)	5.81e−11	**9.31e−08**	2.00(1.36–2.63)	1.22e−09	**1.95e**−**06**	1.42(1.23–1.66)	4.60e−06	**7.36e**−**03**
20,26‐Dihydroxyecdysone	Hormones and hormone related compounds	1.75(0.64–2.86)	2.00e−03	5.05e−02	1.41(0.77–2.05)	1.75e−05	**1.40e**−**03**	1.10(0.95–1.27)	1.86e−01	7.37e−01
7‐Ketocholesterol	Hormones and hormone related compounds	−0.61(−1.71–0.50)	2.80e−01	5.94e−01	−1.05(−1.69–0.41)	1.32e−03	**2.64e**−**02**	1.08(0.93–1.24)	3.06e−01	7.88e−01
Cortisone	Hormones and hormone related compounds	1.72(0.60–2.83)	2.62e−03	6.00e−02	1.28(0.64–1.93)	9.59e−05	**4.25e**−**03**	1.12(0.97–1.29)	1.34e−01	6.88e−01
11‐Dehydro‐TXB3	Hormones and hormone related compounds	1.85(0.73–2.96)	1.18e−03	**4.49e−02**	0.67(0.02–1.32)	4.32e−02	2.21e−01	1.11(0.96–1.28)	1.72e−01	7.13e−01
16‐Phenoxy tetranor prostaglandin A2	Hormones and hormone related compounds	3.00(1.57–4.43)	4.19e−05	**5.16e−03**	1.40(0.57–2.23)	9.59e−04	**2.05e**−**02**	1.26(1.04–1.52)	1.64e−02	4.77e−01
Prednisone	Hormones and hormone related compounds	1.48(0.38–2.59)	8.66e−03	1.25e−01	1.39(0.75–2.02)	2.19e−05	**1.60e**−**03**	1.06(0.92–1.23)	4.13e−01	8.28e−01
Prostaglandin E3	Hormones and hormone related compounds	1.93(0.78–3.08)	1.03e−03	**4.02e−02**	1.07(0.40–1.73)	1.81e−03	**3.08e**−**02**	1.20(1.03–1.40)	1.71e−02	4.77e−01
11‐Deoxyprostaglandin F1alpha	Hormones and hormone related compounds	−1.04(−2.14–0.06)	6.40e−02	3.13e−01	−0.96(−1.59–0.32)	3.29e−03	**4.38e**−**02**	0.93(0.80–1.07)	2.84e−01	7.73e−01
1‐Methylxanthine	Nucleotide and its metabolites	1.78(0.68–2.88)	1.54e−03	**4.70e−02**	0.99(0.35–1.63)	2.50e−03	**3.70e**−**02**	1.28(1.11–1.48)	1.01e−03	9.55e−02
Xanthine	Nucleotide and its metabolites	0.99(−0.18–2.15)	9.84e−02	3.61e−01	1.09(0.41–1.77)	1.59e−03	**2.91e**−**02**	1.11(0.96–1.29)	1.72e−01	7.13e−01
3‐Methylxanthine	Nucleotide and its metabolites	1.78(0.68–2.88)	1.54e−03	**4.70e−02**	0.99(0.35–1.63)	2.50e−03	**3.70e**−**02**	1.28(1.11–1.48)	1.01e−03	9.55e−02
7‐Methylxanthine	Nucleotide and its metabolites	1.78(0.68–2.88)	1.54e−03	**4.70e−02**	0.99(0.35–1.63)	2.50e−03	**3.70e**−**02**	1.28(1.11–1.48)	1.01e−03	9.55e−02
2‐(Dimethylamino)guanosine	Nucleotide and its metabolites	1.68(0.48–2.89)	6.13e−03	1.02e−01	0.99(0.29–1.69)	5.40e−03	6.13e−02	1.41(1.20–1.65)	3.75e−05	**2.00e**−**02**
Creatine phosphate	Nucleotide and its metabolites	1.38(0.24–2.52)	1.75e−02	1.83e−01	1.31(0.65–1.96)	9.82e−05	**4.25e**−**03**	1.23(1.06–1.43)	6.69e−03	3.38e−01
N6‐(2‐Hydroxyethyl)adenosine	Nucleotide and its metabolites	1.84(0.63–3.06)	3.02e−03	6.32e−02	1.09(0.38–1.79)	2.61e−03	**3.80e**−**02**	1.43(1.22–1.69)	1.63e−05	**1.30e**−**02**
2‐Hydroxyisocaproic acid	Organic acid and its derivatives	1.99(0.76–3.21)	1.48e−03	**4.70e−02**	1.06(0.35–1.77)	3.34e−03	**4.38e**−**02**	1.19(1.02–1.40)	3.27e−02	4.77e−01
l‐Lactic acid	Organic acid and its derivatives	2.17(1.05–3.28)	1.44e−04	**1.34e−02**	1.62(0.98–2.26)	8.78e−07	**2.81e**−**04**	1.22(1.06–1.42)	7.68e−03	3.38e−01
5‐Hydroxyhexanoic acid	Organic acid and its derivatives	1.99(0.76–3.21)	1.48e−03	**4.70e−02**	1.06(0.35–1.77)	3.34e−03	**4.38e**−**02**	1.19(1.02–1.40)	3.27e−02	4.77e−01
Uric acid	Organic acid and its derivatives	1.44(0.26–2.62)	1.71e−02	1.81e−01	1.11(0.43–1.80)	1.44e−03	**2.79e**−**02**	1.10(0.94–1.28)	2.47e−01	7.67e−01
2‐Hydroxy‐2‐methyl butyric acid	Organic acid and its derivatives	2.19(0.94–3.45)	6.41e−04	**2.93e−02**	1.27(0.54–2.00)	6.48e−04	**1.69e**−**02**	1.20(1.02–1.42)	2.83e−02	4.77e−01
(S)‐Leucic acid	Organic acid and its derivatives	1.99(0.76–3.21)	1.48e−03	**4.70e−02**	1.06(0.35–1.77)	3.34e−03	**4.38e**−**02**	1.19(1.02–1.40)	3.27e−02	4.77e−01
2‐Hydroxyhexanoic acid	Organic acid and its derivatives	1.99(0.76–3.21)	1.48e−03	**4.70e−02**	1.06(0.35–1.77)	3.34e−03	**4.38e**−**02**	1.19(1.02–1.40)	3.27e−02	4.77e−01
2‐Hydroxy‐3‐methyl butanoic acid	Organic acid and its derivatives	2.17(0.91–3.42)	7.45e−04	**3.32e−02**	1.26(0.54–1.99)	6.96e−04	**1.69e**−**02**	1.20(1.01–1.41)	3.41e−02	4.92e−01
2‐Methoxyacetic acid	Organic acid and its derivatives	1.32(0.23–2.42)	1.82e−02	1.87e−01	0.99(0.36–1.63)	2.18e−03	**3.43e**−**02**	1.16(1.01–1.35)	3.83e−02	4.94e−01
3‐Amino‐5‐hydroxybenzoic acid	Organic acid and its derivatives	1.29(0.13–2.44)	2.89e−02	2.28e−01	1.16(0.49–1.82)	6.89e−04	**1.69e**−**02**	1.16(1.00–1.35)	5.13e−02	5.48e−01
2‐Methyllactic acid	Organic acid and its derivatives	1.89(0.73–3.05)	1.41e−03	**4.70e−02**	1.58(0.91–2.25)	3.87e−06	**4.98e**−**04**	1.21(1.04–1.40)	1.49e−02	4.77e−01
(R)‐2‐Hydroxybutyric acid	Organic acid and its derivatives	1.89(0.73–3.05)	1.41e−03	**4.70e−02**	1.58(0.91–2.25)	3.87e−06	**4.98e**−**04**	1.21(1.04–1.40)	1.49e−02	4.77e−01
Spermidic acid	Organic acid and its derivatives	2.49(1.34–3.64)	2.41e−05	**3.86e−03**	1.31(0.64–1.98)	1.35e−04	**5.42e**−**03**	1.31(1.12–1.52)	6.46e−04	7.38e−02
Icosa‐5,14‐dienoic acid	Organic acid and its derivatives	1.64(0.52–2.76)	4.26e−03	8.22e−02	1.01(0.36–1.66)	2.39e−03	**3.64e**−**02**	1.15(0.99–1.33)	6.68e−02	5.78e−01
Uridine triacetate	Others	1.33(0.22–2.44)	1.85e−02	1.89e−01	1.21(0.57–1.85)	2.28e−04	**7.76e**−**03**	1.08(0.93–1.24)	3.24e−01	7.95e−01
(Z)‐2‐tetracos‐15‐enamidoethanesulfonic acid	Others	1.70(0.59–2.81)	2.72e−03	6.04e−02	1.33(0.68–1.97)	5.34e−05	**2.97e**−**03**	1.12(0.97–1.29)	1.34e−01	6.88e−01
SPH(d18:1)	SL	1.13(0.00–2.27)	4.97e−02	2.81e−01	1.19(0.54–1.85)	3.77e−04	**1.19e**−**02**	1.09(0.94–1.26)	2.64e−01	7.73e−01

*Notes*: For blood pressure‐associated metabolites with FDR adjusted *p* < 0.05 in any of blood pressure trait. Statistically significant associations (FDR‐adjusted *p* < 0.05) are highlighted in bold.

Abbreviations: CI, confidential interval; DBP, diastolic blood pressure; FA, fatty acids; GL, glycerolipids; GP, glycerophospholipids; OR, odds ratio; SBP, systolic blood pressure; SL, sphingolipids.

Cortisol, a steroid hormone, was consistently and significantly associated with all three blood pressure traits, with the following associations: SBP (*β* = 3.71, 95%CI: 2.61–4.81, *p *= 5.81 × 10^−11^), DBP (*β* = 2.00, 95%CI: 1.36–2.63, *p *= 1.22 × 10^−9^), and hypertension (OR = 1.42, 95%CI: 1.23–1.66, *p *= 4.60 × 10^−6^). Its related metabolite, cortisone, was also identified as a potential risk factor for elevated DBP (*β* = 1.28, 95%CI: 0.64–1.93, *p *= 9.59 × 10^−5^). Moreover, prostaglandin E3 (PGE3), a hormone related compound, was associated with a higher SBP (*β *= 1.93, 95%CI: 0.78–3.08, *p *= 1.03 × 10^−3^) and DBP (*β *= 1.07, 95%CI: 0.40–1.73, *p *= 1.81 × 10^−3^) in the generalized linear models (GLMs).

In the class of amino acid and its metabolites, l‐glutamic acid demonstrated a significant positive association with elevated DBP (*β *= 1.19, 95%CI: 0.49–1.89, *p *= 8.68 × 10^−4^). Similarly, higher levels of the dipeptide asp–phe was also associated with increased DBP (*β *= 1.10, 95%CI: 0.44–1.76, *p *= 1.05 × 10^−3^).

Additionally, a subset of lysophospholipids such as lysophosphatidic acid (LPA), lysophosphatidylcholine (LPC), and lysophosphatidylethanolamine (LPE) exhibited a consistently positive association with blood pressure. Notably, the effects of two LPC, including LPC (0:0/14:0) and LPC (0:0/16:0) were replicated in Xiazhi sub‐cohorts (Table S6). These findings demonstrate robust and replicable associations between these metabolites and blood pressure across independent cohorts.

Additionally, N‐lactoyl‐phenylalanine was significantly associated with incident hypertension (OR = 1.24, 95%CI: 1.01–1.53, *p* = 4.07 × 10^−2^), while N‐acetyl‐d‐lactosamine exhibited a significant association with DBP change (*β* = 0.31, 95%CI: 0.07–0.55, *p* = 1.19 × 10^−2^), demonstrating a consistent direction with the baseline blood pressure associations (Table S7).

### 2.5 Blood Pressure‐Associated Ratios of Metabolites

Based on 91 blood pressure‐associated metabolites, metabolite ratios were identified and calculated. Eight metabolite ratios demonstrated significant associations with any of blood pressure traits (FDR adjusted *p *< 0.05; Table 3).

**TABLE 3:** Blood pressure‐associated ratios of metabolites in generalized linear models.

Ratios of metabolites	Pathway	Enzyme	Beta(95%CI) for SBP	*p* for SBP	FDR adjusted *p* for SBP	Beta(95%CI) for DBP	*p* for DBP	FDR adjusted *p* for DBP	OR(95%CI) for hypertension	*p* for Hypertension	FDR adjusted *p* for hypertension
l‐Glutamaic acid/l‐glutamine	Alanine, aspartate and glutamate metabolism	Glutamate–ammonia ligase; glutaminase	0.93(−0.28–2.14)	1.33e−01	5.04e−01	1.21(0.51–1.91)	7.17e−04	**7.49e**−**03**	1.17(1.00–1.38)	5.03e−02	5.16e−01
Creatine/creatine phosphate	Arginine and proline metabolism	Creatine kinase B	−1.63(−2.79–0.48)	5.64e−03	7.70e−02	−1.15(−1.82–0.49)	7.31e−04	**7.49e**−**03**	0.77(0.66–0.90)	8.21e−04	**1.68e**−**02**
7‐Methylxanthine/7‐methyluric acid	Caffeine metabolism	Xanthine oxidoreductase	1.60(0.51–2.70)	4.11e−03	7.70e−02	0.90(0.27–1.54)	5.32e−03	**2.72e**−**02**	1.24(1.07–1.43)	3.72e−03	5.08e−02
l‐Glutamic acid/γ‐Glu–Cys	Glutathione metabolism	Glutamate–cysteine ligase	1.17(−0.05–2.38)	5.96e−02	3.49e−01	1.37(0.67–2.07)	1.33e−04	**2.72e**−**03**	1.07(0.92–1.26)	3.82e−01	7.85e−01
Pyroglutamic acid/l‐glutamic acid	Glutathione metabolism	5‐Oxoprolinase; ATP‐hydrolyzing	−0.49(−1.70–0.72)	4.28e−01	7.15e−01	−1.07(−1.77–0.37)	2.65e−03	**1.81e**−**02**	0.92(0.79–1.08)	3.23e−01	7.85e−01
Hypoxanthine/xanthine	Purine metabolism	Xanthine oxidoreductase	−1.20(−2.37–0.02)	4.59e−02	3.49e−01	−0.98(−1.66–0.30)	5.00e−03	**2.72e**−**02**	0.90(0.77–1.05)	1.82e−01	7.85e−01
SPH(d18:1)/1‐beta‐d‐galactosylsphingosine	Sphingolipid metabolism	Uridine diphosphate glycosyltransferase 8	1.13(−0.01–2.28)	5.29e−02	3.49e−01	1.09(0.43–1.75)	1.29e−03	**1.06e**−**02**	1.14(0.99–1.33)	7.72e−02	6.33e−01
Cortisol/cortisone	Steroid hormone biosynthesis	Hydroxysteroid 11‐beta dehydrogenase 2; hydroxysteroid 11‐beta dehydrogenase 1	3.74(2.64–4.85)	5.33e−11	**2.19e**−**09**	1.77(1.13–2.42)	9.72e−08	**3.99e**−**06**	1.47(1.26–1.71)	1.17e−06	**4.78e**−**05**

*Notes*: For blood pressure‐associated ratios of metabolites with FDR adjusted *p* < 0.05 in any of blood pressure trait. Statistically significant associations (FDR‐adjusted *p* < 0.05) are highlighted in bold.

Abbreviations: CI, confidential interval; DBP, diastolic blood pressure; FA, fatty acids; GL, glycerolipids; GP, glycerophospholipids; OR, odds ratio; SBP, systolic blood pressure; SL, sphingolipids.

**FIGURE 2:**
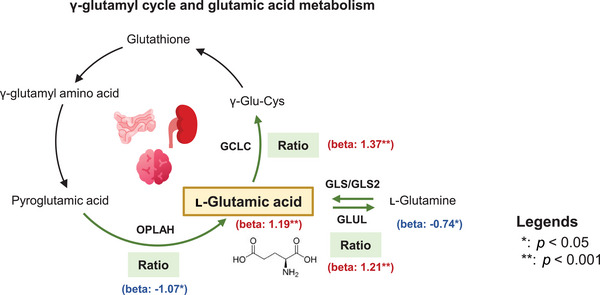
Integrated associations of l‐glutamic acid and related ratios with diastolic blood pressure. This diagram illustrates the significant associations (beta coefficients and *p* from generalized linear models) between l‐glutamic acid, its derived ratios, and DBP within the γ‐glutamyl cycle and glutamic acid metabolism pathways. The “ratio” refers to the ratio of upstream to downstream metabolites, serving as a proxy for enzymes. Beta values in red indicate a positive association with DBP (higher levels correlate with higher blood pressure), while those in blue indicate an inverse association (higher levels correlate with lower blood pressure). Statistical significance is denoted as *p < 0.05 and **p < 0.001. γ‐Glu–Cys, gamma‐glutamylcysteine; DBP diastolic blood pressure; GCLC, glutamate–cysteine ligase; GLS/GLS2, glutaminase; GLUL, glutamate–ammonia ligase; OPLAH, 5‐oxoprolinase, ATP‐hydrolyzing.

Cortisol, identified as the most significantly blood pressure‐associated metabolite, undergoes bidirectional conversion to cortisone within the steroid hormone biosynthesis pathway. The cortisol‐to‐cortisone ratio emerged as the top metabolite ratio associated with blood pressure, demonstrating significant associations with SBP (*β *= 3.74, 95%CI: 2.64–4.85, *p *= 5.33 × 10^−11^), DBP (*β *= 1.77, 95%CI: 1.13–2.42, *p *= 9.72 × 10^−8^), and hypertension (*β *= 1.47, 95%CI: 1.26–1.71, *p *= 1.17 × 10^−6^).

l‐Glutamic acid was identified as a potential risk factor of higher DBP. Integrated evidence supporting the association between l‐glutamic acid and its related metabolites with DBP is summarized in Figure 2. Within the γ‐glutamyl cycle, the conversion of pyroglutamic acid to l‐glutamic acid is catalyzed by 5‐oxoprolinase, ATP‐hydrolyzing (OPLAH), while glutamic acid–cysteine ligase (GCLC), a rate‐limiting enzyme in glutathione synthesis, catalyzes the formation of γ‐Glu–Cys (γ‐glutamylcysteine) from l‐glutamic acid. The ratio of pyroglutamic acid to l‐glutamic acid (reflecting OPLAH activity) was negatively associated with DBP (*β *= −1.07, 95%CI: −1.77–0.37, *p *= 2.65 × 10^−3^), whereas the ratio of l‐glutamic acid to γ‐Glu–Cys (reflecting GCLC activity) showed a positive association with DBP (*β *= 1.37, 95%CI: 0.67–2.07, *p *= 1.33 × 10^−4^). Although not reaching strict statistical significance, l‐glutamine showed a suggestive protective association with DBP (*β *= −0.738, 95%CI: −1.404–0.071, *p *= 3.01 × 10^−2^). In glutamic acid metabolism, the bidirectional conversion between l‐glutamic acid and l‐glutamine is facilitated by glutaminase (GLS/GLS2) and glutamate–ammonia ligase (GLUL). The ratio of l‐glutamic acid to l‐glutamine was significantly positively associated with DBP (*β *= 1.21, 95%CI: 0.51–1.91, *p *= 7.17 × 10^−4^). Collectively, these findings indicated that l‐glutamic acid and its metabolic derivatives may play important roles in elevating DBP.

### 2.6 Metabolic Pathway Analysis

Pathway analysis was conducted using the Kyoto Encyclopedia of Genes and Genomes (KEGG) database. Two pathways met the significance criteria (*p* < 0.05, pathway impact > 0.1): caffeine metabolism and glycerophospholipid metabolism (Figure S4 and Table S8).

### 2.7 Potential Causality Between Metabolites and Blood Pressure

MR analysis was conducted to explore the causality between metabolites and blood pressure based on the 91 blood pressure‐associated metabolites. The mean *F*‐statistics for 10 metabolites were presented in Table S9 confirming there was no weak IV. Ten genetically predicted metabolites were identified to be associated with higher level of SBP or DBP (Table 4). As shown in Table 4, PGE3, sphingosine (d18:1) (SPH[d18:1]), N‐acetyl‐d‐lactosamine, and 13‐tetradecynoic acid were novel metabolites that have not been previously reported to be associated with blood pressure, while six had been previously reported.

**TABLE 4:** Significant blood pressure‐associated metabolites with the same effect direction in generalized linear models and Mendelian randomization analyses.

			Generalized linear model		Mendelian randomization analysis			
Metabolites	Previously reported	Trait	Beta(95%CI)	FDR adjusted *p*	Blood pressure GWAS	IVs	Beta(95%CI)	*p*
Asp–phe	Yes	DBP	1.10(0.44–1.76)	2.17e−02	BBJ	11	0.023(0.011–0.036)	3.09e−04
l‐Glutamic acid	Yes	DBP	1.19(0.49–1.89)	1.96e−02	BBJ	9	0.015(0.000–0.031)	4.71e−02
Nα‐acetyl‐l‐glutamine	Yes	SBP	2.09(0.93–3.25)	2.47e−02	KoGES	5	0.023(0.000–0.046)	4.76e−02
N‐acetyl‐d‐lactosamine	No	DBP	1.03(0.40–1.67)	2.79e−02	KoGES	10	0.026(0.010–0.042)	1.16e−03
N‐acetyl‐d‐lactosamine	No	DBP	1.03(0.40–1.67)	2.79e−02	TWB	11	0.018(0.004–0.031)	8.85e−03
N‐acetyl‐d‐lactosamine	No	SBP	2.50(1.41–3.59)	2.15e−03	TWB	11	0.014(0.001–0.028)	3.41e−02
13‐Tetradecynoic acid	No	SBP	1.79(0.67–2.90)	4.70e−02	TWB	5	0.020(0.002–0.038)	3.00e−02
Carnitine C16:1	Yes	SBP	1.87(0.72–3.02)	4.70e−02	TWB	5	0.020(0.002–0.038)	2.82e−02
Cortisol	Yes	SBP	3.71(2.61–4.81)	9.31e−08	BBJ	5	0.024(0.006–0.042)	8.95e−03
Prostaglandin E3	No	DBP	1.07(0.40–1.73)	3.08e−02	KoGES	6	0.024(0.003–0.045)	2.34e−02
Creatine phosphate	Yes	DBP	1.31(0.65–1.96)	4.25e−03	BBJ	6	0.016(0.003–0.029)	1.59e−02
SPH(d18:1)	No	DBP	1.19(0.54–1.85)	1.19e−02	BBJ	7	0.013(0.001–0.024)	3.41e−02

Abbreviations: BBJ, the BioBank Japan; CI, confidential interval; DBP, diastolic blood pressure; FDR, false discovery rate; GWAS, genome‐wide association study; KoGES, the Korean Genome and Epidemiology Study; SBP, systolic blood pressure; SNP, single nucleotide polymorphisms; SPH, sphingosine; TWB, the Taiwan Biobank; IVs, instrumental variables.

Cortisol, identified as the top blood pressure‐associated metabolite, showed a potential causal and statistically significant positive relationship with SBP in MR analysis using the BBJ dataset (*β*_IVW_ = 0.024, 95%CI: 0.006–0.042, *P*_IVW_ = 8.95 × 10^−3^, five IVs). A higher genetically predicted PGE3 level was associated with an increased DBP (*β*_IVW_ *=* 0.024, 95%CI: 0.003–0.045, *P*_IVW_ = 2.34 × 10^−2^, six IVs) using the KoGES GWAS summary data.

Additionally, l‐glutamic acid, a key component of multiple blood pressure‐associated metabolite ratios, exhibited a significant association with DBP in BBJ (*β*_IVW_ = 0.015, 95%CI: 0.000–0.031, *P*_IVW_ = 4.71 × 10^−2^, nine IVs). The dipeptide asp–phe was significantly associated with DBP in BBJ (*β*_IVW_ = 0.023, 95%CI: 0.011–0.036, *P*_IVW_ = 3.09 × 10^−4^, 11 IVs).

Importantly, three of the 10 identified metabolites including asp–phe, N‐acetyl‐d‐lactosamine, and creatine phosphate were repeatedly measured in the THSBC cohort, enabling cross‐validation of our findings (Table S10). Asp–phe, N‐acetyl‐d‐lactosamine, and creatine phosphate were identified as the risking roles of hypertension using BBJ GWAS summary data.

Reverse MR analysis indicated that most metabolites exhibited no significant evidence of reverse causation, as detailed in Table S11. Sensitivity analyses using MR Robust Adjusted Profile Scoring (MR‐RAPS) and MR‐Egger methods yielded consistent results with the main findings (Tables S12 and S15).

### 2.8 Potential Causality Between Ratios of Metabolites and Blood Pressure

Two metabolite ratios showed potential causal relationship with blood pressure. The mean *F*‐statistics for 2 ratios of metabolites were presented in Table S9 confirming there was no weak IV. The genetically predicted ratios of cortisol/cortisone and creatine/phosphocreatine exhibited significant association with blood pressure (Table 5). Sensitivity analyses based on MR‐RAPS and MR‐Egger methods showed consistent results with the main associations (Table S14).

**TABLE 5:** Significant blood pressure‐associated ratios of metabolites with the same effect direction for GLM and MR analyses.

		Generalized linear model		Mendelian randomization analysis			
Ratios of metabolites	Trait	Beta(95%CI)	FDR adjusted *p*	Blood pressure GWAS	IVs	Beta(95%CI)	*p*
Creatine/phosphocreatine	DBP	−1.15(−1.82–0.49)	7.31e−04	BBJ	8	−0.017(−0.032–0.002)	2.57e−02
Cortisol/cortisone	SBP	3.74(2.64–4.85)	5.33e−11	BBJ	5	0.020(0.005–0.034)	7.19e−03

Abbreviations: BBJ, the BioBank Japan; CI, confidential interval; DBP, diastolic blood pressure; FDR, false discovery rate; GLM, generalized linear model; GWAS, genome‐wide association study; IVs, instrumental variables; KoGES, the Korean Genome and Epidemiology Study; MR, Mendelian randomization analysis; SBP, systolic blood pressure; SNP, single nucleotide polymorphisms; TWB, the Taiwan Biobank.

### 2.9 Multiomics Evidence for the Metabolite–Blood Pressure Association

**FIGURE 3:**
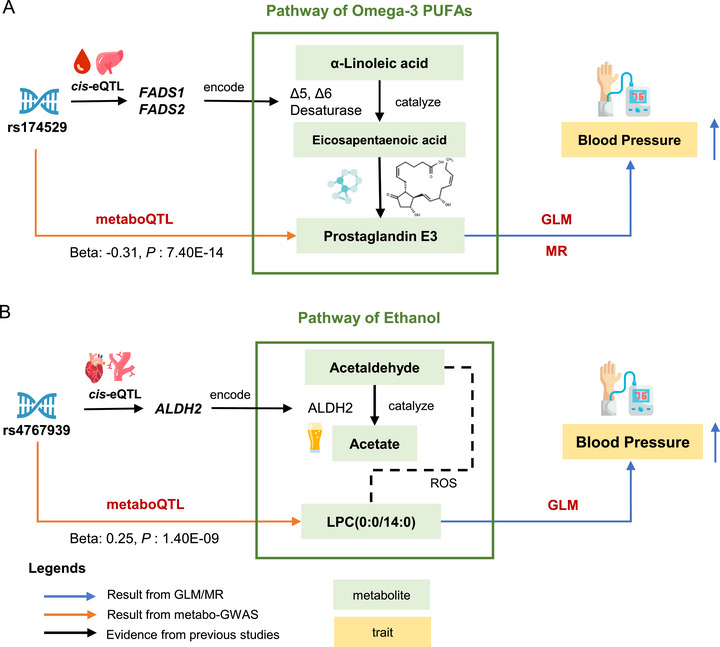
The putative genetics–gene expression–metabolite–trait evidence chain for prostaglandin E3 and LPC(0:0/14:0). (A) Prostaglandin E3 (PGE3). In GLM and MR analysis, increased level of PGE3 was associated with higher blood pressure (indicated by right blue arrows). To further explore the association, molecular signals from eQTLs and metaboQTLs were incorporated. In metabo‐GWAS, metaboQTL rs174529 at *FADS1* and *FADS2* was associated with PGE3. The genetic variant rs174529 was identified as a *cis*‐eQTL for *FADS1* in whole blood and liver *FADS2* in whole blood in GTEx. The genes *FADS1* and *FADS2* encode Δ5‐desaturase and Δ6‐desaturase, respectively. In pathway of omega‐3 PUFAs, Δ5‐desaturase and Δ6‐desaturase are important enzymes catalyzing ALA into EPA, and Δ6‐desaturase is the rate‐limiting enzyme. EPA could be further synthesized into PGE3. The mechanism responsible for the increased concentrations of PGE3 with higher blood pressure may be due to an increased expression of *FADS1* and *FADS2* and the higher activity of Δ5‐desaturase and Δ6‐desaturase, highlighting the potential role of PUFA metabolism in the development of hypertension. (B) LPC (0:0/14:0). In the GLM, LPC (0:0/14:0) was positively associated with elevated blood pressure, a finding further validated in the Xiazhi cohort using targeted metabolomics. The SNP rs4767939 was identified as a metaboQTL for LPC (0:0/14:0) and a *cis*‐eQTL for *ALDH2* in arterial and cardiac tissues, as documented in the GTEx database. The *ALDH2* gene encodes ALDH2, a critical enzyme in ethanol metabolism. Following alcohol consumption, ethanol is initially oxidized to acetaldehyde by ADH and subsequently metabolized to acetate by ALDH2. Acetaldehyde may elevate the levels of proinflammatory lysophospholipids through a reactive oxygen species (ROS)‐dependent mechanism. Consequently, the observed increase in LPC (0:0/14:0) levels associated with higher blood pressure may be attributed to the inflammatory effects resulting from reduced ALDH2 activity and subsequent acetaldehyde accumulation. ADH, alcohol dehydrogenase; ALA, α‐linoleic acid; ALDH2, aldehyde dehydrogenase 2; EPA, eicosapentaenoic acid; eQTL, expression quantitative trait locus; *FADS1*, fatty acid desaturase 1; *FADS2*, fatty acid desaturase 2; GLM, generalized linear model; GWAS, genome wide association study; LPC, lysophosphatidylcholine; metaboQTL, metabolite quantitative trait locus; MR, Mendelian randomization analysis; PGE3, prostaglandin E3; PUFA, polyunsaturated fatty acid.

PGE3 was robustly associated with a higher DBP supported by metabolome and MR analyses. Furthermore, single nucleotide polymorphisms (SNP) rs174529 was identified as a potential genetic regulator of PGE3 through metabo‐GWAS (effect allele: C, *β *= −0.31, *p *= 7.40 × 10^−14^) (Figures 3A and S5 and Table S16). This SNP was found to be a *cis*‐expression quantitative trait locus (eQTL) for fatty acid desaturase 1 (*FADS1*) gene (Table S17). In the pathway of omega‐3 polyunsaturated fatty acid (PUFAs), *FADS1* and *FADS2* encode key enzymes (Δ5‐ and Δ6‐desaturases) involved in the conversion of α‐linoleic acid (ALA) to eicosapentaenoic acid (EPA), a precursor of PGE3. The mechanism linking PGE3 to increased blood pressure may be mediated by enhanced expression of *FADS1* and *FADS2*, and the higher enzymatic activity of Δ5‐ and Δ6‐desaturases, suggesting the potential role of omega‐3 PUFA metabolism in hypertension development.

In the association analysis, LPC (0:0/14:0) exhibited a positive association with elevated blood pressure. The SNP rs4767939 was identified as a metabolite quantitative trait locus (metaboQTL) for LPC (0:0/14:0) and a *cis*‐eQTL for acetaldehyde dehydrogenase 2 (*ALDH2*) (Figures 3B and S6 and Tables S16 and S17). The *ALDH2* gene encodes ALDH2, a key enzyme in ethanol metabolism. Following alcohol consumption, ethanol is first oxidized to acetaldehyde by alcohol dehydrogenase (ADH) and subsequently converted to acetate by ALDH2. Therefore, the observed positive association between LPC (0:0/14:0) levels associated and increased blood pressure may be mediated by reduced *ALDH2* activity and the consequent accumulation of acetaldehyde.

### 2.10 Potential Nonlinear Metabolite–Blood Pressure Association

Restricted cubic splines (RCS) analyses showed that nearly all metabolites and ratios exhibited approximately linear relationships with the three blood pressure traits (*p* for nonlinear > 0.05; Figures S7 and S8).

### 2.11 Mediation Effects of Metabolites Between Lifestyle and Blood Pressure

The significant associations between lifestyle factors and metabolites, as well as between lifestyle factors and blood pressure, are presented in Tables S19 and 20, respectively.

In mediation analysis, our results showed that l‐glutamic acid statistically mediated the associations of body mass index (BMI) and waist circumference (WC) with DBP, with mediation proportions of 12.95 and 12.16%, respectively (both *p* < 0.05) (Figure S9 and Table S21). Furthermore, the healthy lifestyle score (HLS) demonstrated a favorable association with DBP statistically mediated by l‐glutamic acid, accounting for 31.53% of the effect. Additionally, the associations of BMI and WC with SBP were statistically mediated by Nα‐acetyl‐l‐glutamine and N‐acetyl‐d‐lactosamine, with mediation proportions ranging from 6.48 to 10.03%, respectively (both *p* < 0.05) (Table S21).

## 3 Discussion

This study provides comprehensive insights into the metabolic underpinnings of blood pressure among Chinese population. A total of 91 blood pressure‐associated metabolites and eight metabolite ratios were identified in the metabolome analysis. Furthermore, MR analyses revealed potential causality between 10 metabolites as well as two metabolite ratios and blood pressure. Putative SNP–gene expression–metabolite–trait evidence chains for PGE3 and LPC (0:0/14:0) were constructed by integrating multiomics data. Last, three metabolites were identified as mediators in the relationship between modifiable lifestyle factors and blood pressure. These findings introduced novel metabolites for blood pressure and elucidated the potential molecular mechanisms underlying blood pressure regulation.

Previous studies in European population have reported some blood pressure‐associated metabolites and metabolic pathways. Of the 24 novel metabolites robustly associated with blood pressure in the BHS, three were amino acid and nucleotide metabolites, seven were cofactor/vitamin or xenobiotic metabolites, 10 were lipids, and four remained unnamed [10]. Among them, l‐glutamic acid was consistently associated with higher blood pressure in both the BHS and our study. Our work expands upon this by elucidating the roles of its related metabolite ratios and enzymes in blood pressure regulation. By profiling 1601 metabolites, our study significantly expands the metabolic landscape and deepens the understanding of blood pressure's metabolic underpinnings. In our study, 10 metabolites were identified to be associated with blood pressure based on the metabolome analysis and MR analysis. Among these, PGE3, SPH[d18:1], N‐acetyl‐d‐lactosamine, and 13‐tetradecynoic acid were novel metabolites that have not been previously reported to be associated with blood pressure. Furthermore, the associations between six metabolites including cortisol, l‐glutamic acid, asp–phe and blood pressure were validated. As for prior MR studies established associations, we further integrated multiomics data and uncovered two novel molecular pathways of PGE3 and LPC (0:0/14:0) to improve the understanding of metabolic mechanism of blood pressure regulation [21, 22].

Several blood pressure‐associated metabolites were identified, and the potential causality were further substantiated. For example, cortisol emerged as a key metabolite positively associated with SBP, DBP, and hypertension in GLMs and had potential positive causal relationship with blood pressure in MR analysis. These finding were consistent with numerous prior studies [32, 33, 34, 35]. Moreover, cortisone, the primary inactive metabolite of cortisol, along with the cortisol/cortisone ratio, exhibited a robust positive correlation with SBP and DBP. The conversion of cortisol to cortisone is catalyzed by 11 *β*‐hydroxysteroid dehydrogenase 2 (11 *β*‐HSD2), while 11 *β*‐HSD1 promotes the reverse reaction [33]. Therefore, the ratio of cortisol and cortisone may serve as a biomarker to global 11 *β*‐HSD activity [33, 36, 37, 38]. These results provided the evidence that cortisol and the cortisol/cortisone ratio may be essential metabolites for blood pressure regulation.

l‐Glutamic acid demonstrated a significant positive association with blood pressure in both association and MR analyses. This result aligns with earlier studies suggesting glutamic acid's involvement in blood pressure regulation [39, 40, 41, 42]. Glutamic acid is also a critical excitatory neurotransmitter and plays a well‐established role in the central regulation of blood pressure, as extensively described in prior research [43]. Elevated glutamic acid release within key brain regions, such as the hypothalamus and brainstem, has been shown to induce a significant increase in blood pressure through its action on central cardiovascular control pathways [43]. While some studies suggest that glutamic acid receptor activation may exacerbate endothelial cell dysfunction and contribute to blood pressure elevation, others point to its protective effects by promoting nitric oxide (NO) bioavailability, which can induce vasodilation [44, 45, 46, 47, 48, 49]. This dual role in blood pressure regulation warrants further exploration through functional studies. Moreover, the ratio of l‐glutamic acid to l‐glutamine was positively correlated with blood pressure, which is associated with a higher risk of metabolic abnormity and stroke in other studies [50, 51]. The two metabolites could convert into each other by GLUL and GLS1/GLS2 [52]. Glutamine exerts potent antioxidant and anti‐inflammatory effects to promote cardiovascular health and drop the blood pressure by serving as an l‐arginine precursor to optimize NO synthesis [53]. Consistent with previous research, glutamine was negatively related to DBP and hypertension with *p* value <0.05 in our study [54, 55]. Moreover, the ratio of l‐glutamic acid and γ‐Glu–Cys representing GCLC, a rate‐limiting enzyme in glutathione metabolism, was positively associated with blood pressure. Therefore, glutamic acid and related glutathione metabolism may be important metabolic factors in blood pressure regulation.

In addition to previously identified metabolites, several novel blood pressure‐associated metabolites were uncovered. PGE3 was positively associated with blood pressure. The SNP rs174529, a *cis*‐eQTL for *FADS1* and *FADS2*, was identified as a key genetic regulator of PGE3 in our metabo‐GWAS. Other metabo‐GWASs showed that *FADS1* and *FADS2* genetic loci were strongly associated with plasma lipids, glucose metabolism, and cardiovascular disease [56, 57]. The two genes encode Δ5‐desaturase and Δ6‐desaturase, respectively, which are the essential enzymes for PUFA conversion and serve as major determinants of PUFA levels. Altered activity of the two enzymes has been reported to be associated with several diseases, including metabolic derangements, type 2 diabetes, cardiovascular disease, and inflammation [56]. The observed elevation in PGE3 concentrations associated with higher blood pressure may be attributed to upregulated expression of *FADS1* and *FADS2*, coupled with enhanced activity of Δ5‐desaturase and Δ6‐desaturase. Therefore, the potential regulation chain from SNP to blood pressure was uncovered for PGE3 by integration of multiomics data.

In our study, the elevated blood pressure effects associated with BMI and WC were partially mediated by l‐glutamic acid, accounting for approximately 12% of the observed effects. This finding aligns with previous studies that have demonstrated positive associations between BMI, WC, and glutamic acid levels [40, 58, 59]. Mechanistically, glutamic acid plays a critical role in the biosynthesis of γ‐aminobutyric acid, a neurotransmitter implicated in appetite regulation [59]. Furthermore, the favorable blood pressure effects of the HLS were also mediated by l‐glutamic acid, with a mediation proportion of up to 31.53%. Prior research has consistently linked lower levels of glutamic acid with healthier lifestyle factors, including the absence of chronic or infectious diseases, normal weight status, high physical activity levels, nonsmoking behavior, and adherence to a healthy dietary pattern [27]. Following the lifestyle intervention, significant reductions were observed in 64 metabolites, including glutamic acid [60]. These findings collectively suggested that modifiable lifestyle factors may influence blood pressure through mechanisms involving glutamic acid, providing a strong rationale for future lifestyle intervention studies aimed at blood pressure management.

The major strength of the current study consisted in the combination of relative large‐scale cross‐sectional and prospective data, and the fact that the application of untargeted metabolomic and widely targeted metabolomics approaches with 1601 metabolites, which enabled the identification of novel blood pressure metabolites. MR and analysis were used to determination of the causal relationship. Additionally, the combination of genetic, transcriptomic, and metabolomic data provides a comprehensive framework for understanding blood pressure regulation.

This study also had several limitations that deserve consideration. First, the GWAS data for metabolites were derived from Chinese participants, while the GWAS for blood pressure and hypertension were based on Japanese and Korean cohorts, which may present slight genetic differences. Nevertheless, these populations share genetic similarities with East Asians. Second, caution should be taken when generalizing these results to populations outside of Chinese, as the cohort consisted primarily of middle‐aged and elderly individuals. Third, the number of targeted metabolites measured in Xiazhi sub‐cohort was relatively small. Further large‐scale targeted replications are needed. Fourth, it should be noted that the metabolite GWASs combined data from community‐based and pregnancy cohorts, a design that may lead to heterogeneity. The empirical data indicated that cohort‐specific differences in effect sizes were limited [61]. Furthermore, two identified associations were validated in the THSBC cohort, demonstrating high consistency across diverse populations. Last, we did not investigate detailed medical information such as the number of medications, specific drug classes, or dosage. Medication was included only as a binary variable. Although sensitivity analyses showed consistent results, further research is needed to examine the more complex effects of medications.

## 4 Conclusion

In conclusion, we identified 91 blood pressure‐associated metabolites and further prioritized 10 metabolites that exhibited potential causal effect on blood pressure, supported by genetic evidence. By integrating multiomics data, we uncovered putative genetic–metabolite–trait evidence chains that provide valuable insights into blood pressure regulation. These findings advance our understanding of blood pressure biomarkers and may contribute to future research into the pathophysiology of hypertension.

## 5 Methods

### 5.1 Study Population

This study utilized data from middle‐aged and older Chinese adults enrolled in the Liuheng sub‐cohort of the ZMSC. A comprehensive description of the ZMSC has been provided elsewhere [62]. The Liuheng sub‐cohort consists of participants from Liuheng island in Zhoushan, Zhejiang. Baseline survey (*N *= 2414) was conducted in 2015. The participants aged ≥18 years and having information on blood pressure, genome, and serum metabolome were included from the baseline survey. Individuals were excluded if they had cancers or cardiovascular diseases. This study included 1188 individuals from the baseline population. Among them, 1160 subjects were followed up as the same protocol in 2020. The information of Xiazhi sub‐cohort and the THSBC were detailed in Supporting Information: Methods.

### 5.2 Questionnaire Interview

Participants were interviewed face‐to‐face using a structured questionnaire that collected demographic information (e.g., date of birth, sex), lifestyle factors (e.g., smoking, alcohol consumption), family history, diagnosed diseases, and corresponding drugs. Hypertension‐related information was collected by trained staff and included two binary variables: (1) self‐reported hypertension, defined as an affirmative response to the question “Has a doctor ever diagnosed you with hypertension?”; and (2) use of antihypertensive medication, defined as an affirmative response to the question “Have you ever taken prescribed medication for hypertension?”. Hypertension status (prevalent and 5‐year incident cases) and use of antihypertensive medication were ascertained through the structured questionnaire interviews. Physical activity was assessed using the International Physical Activity Questionnaire, with the metabolic equivalent (MET) hours per week (MET‐h/week) calculated for each activity level (low, moderate, and high). A semi‐quantitative food frequency questionnaire was used to assess the average frequency and amount of consumption of key food items (vegetables, fruits, red meat, and aquatic products and seafood) during the previous year. Red meat included pork, beef, and lamb. Aquatic products and seafood encompass a diverse range of marine and freshwater organisms. In addition, average daily sleep duration was also recorded by trained interviewers.

### 5.3 Assessment of Anthropometric and Biochemical Indicators

Physical examinations included weight, height, WC. The BMI was calculated as weight (kg)/height (m)^2^.

Blood pressure was measured three times at 30‐s intervals by the trained personnel following a standardized protocol in a seated position using a mercury sphygmomanometer. The blood pressure value was calculated as the average of the three measurements. Hypertension was defined as SBP ≥ 140 mmHg, DBP ≥ 90 mmHg, self‐reported diagnosis of hypertension, or current use of antihypertensive medication [63]. Additionally, the changes in SBP and DBP per year were calculated by dividing the difference between levels of after follow‐up and baseline blood pressure by the number of years between two surveys. Incident hypertension patients were defined as individuals who did not have hypertension at baseline but developed hypertension during follow‐up.

Blood TG, HDLC, and FPG levels were assessed by standard clinical methods.

### 5.4 Metabolites Measurement

Both untargeted and widely targeted metabolomic profiling were performed for the serum samples from the Liuheng sub‐cohort. The details of untargeted and widely targeted detection were described in Supporting Information: Methods. A total of 1912 metabolites were identified in the Liuheng sub‐cohort. A total of 1601 out of 1912 metabolites were quantified (qualitative level ≤ 2; Table S1) and chosen in this study.

The procedures of targeted metabolic profiling for sample from Xiazhi were shown in Supporting Information: Methods. Among the 1601 metabolites, 40 were measured repeated in Xiazhi (32 unique metabolites in Xiazhi; Table S2).

In the THSBC cohort, metabolomic profiling was performed using a widely targeted approach, as detailed elsewhere [61]. A total of 484 out of 1601 metabolites were measured repeatedly in the THSBC (Table S3).

### 5.5 Genotype Genotyping, Imputation, and Quality Control

In Liuheng sub‐cohort, genotyping was performed using the Illumina ASA‐750K array, following standard quality control procedures. In the THSBC, genotyping was performed using Affymetrix Genome‐Wide Human SNP Array 6.0 Chips or Illumina Infinium OmniZhongHua‐8 Chips. SNPs were excluded based on the following criteria: Hardy‐Weinberg equilibrium *p* < 1.00 × 10^−5^, minor allele frequency < 0.05 or missing rate ≥ 0.05. The analysis process also excluded individuals with high or low proportion of heterozygous genotypes (outliers were defined as more than or less than 3 SD from the mean), sex mismatch or different ancestral ancestry (the first two principal components were more than or less than 5 SD from the mean), and genotype missing > 0.05. The imputation was performed using the Michigan Imputation Server (https://imputationserver.sph.umich.edu/index.html#!), with the reference panel based on the East Asian population from the 1000 Genomes Project (Phase 3 v5). Genetic variants with an imputation quality score > 0.3 were retained. Last, a total of 4 million SNPs were included for subsequent analysis.

### 5.6 Statistical Analysis

#### 5.6.1 Descriptive Analysis

Descriptive analysis was performed for all participants, males and females. Continuous variables are expressed as means (SD). Categorical variables are presented as counts (percentages).

#### 5.6.2 Association Analysis

Regarding missing data, participants with missing values for key variables, including age, sex, or blood pressure measurement, were excluded from the analysis to ensure data completeness and reliability. Prior to analysis, metabolite levels were log‐transformed. Then, outliers exceeding 5 SD were removed, followed by an inverse normal transformation. GLMs were employed to identify blood pressure‐associated metabolites. Considering the effects of antihypertensive medication on blood pressure, we excluded the individuals taking antihypertensive medications in the models of metabolites and continuous blood pressure variables, SBP and DBP and added medication usage as a confounding factor in the models of metabolites and hypertension. Using multiple linear models, the coefficients (*β*) and 95% confidence intervals (CIs) for each metabolite in relation to SBP, DBP were estimated adjusting for age, sex, BMI, smoking status, and alcohol consumption. In the analysis of blood pressure changes, baseline blood pressure levels were further adjusted. Using multiple logistic models, the odds ratios (OR) and 95% CIs for prevalent hypertension were calculated after adjusting for age, sex, BMI, smoking, alcohol consumption, and antihypertensive drug use. Statistical significance was determined using FDR correction for each blood pressure trait based on the number of 1601 metabolites, with an FDR threshold of <0.05 considered significant. FDR‐adjusted *p* values were calculated using the p.adjust() function in R with the default Benjamini–Hochberg method (method = “fdr”). Drugs and noninformative small peptides were excluded from the list of blood pressure‐associated metabolites.

For metabolites identified as blood pressure‐associated in the GLMs, the associations between metabolites and longitudinal blood pressure changes as well as incident hypertension were also analyzed the same analytical approach. Metabolites were considered significantly associated with blood pressure progression if they satisfied the following criteria: (1) *p *< 0.05; and (2) the direction of effect was consistent with that observed for baseline blood pressure in the corresponding trait.

Based on blood pressure‐associated metabolites, 41 metabolite ratios were identified using the KEGG database and calculated by dividing the upstream metabolite by the downstream metabolite within the same metabolic pathway, followed by inverse normal transformation. These ratios were subsequently analyzed in the same manner to assess their associations with baseline blood pressure. FDR correction were conducted for each trait based on the number of 41 ratios.

For sensitivity analysis, stratified analyses were performed by sex. Individuals taking antihypertensive medication were included in the analyses of both SBP and DBP in baseline. The detailed procedures for the association analysis conducted in the external replication using the Xiazhi sub‐cohort are described in the Supporting Information: Methods.

#### 5.6.3 Pathway Analysis

For further biological interpretation, pathway analyses were conducted on metabolites significantly associated with blood pressure, utilizing the KEGG database through MetaboAnalyst 6.0 platform (http://www.metaboanalyst.ca/).

#### 5.6.4 MR Analysis

For metabolite–blood pressure associations supported by observational evidence (FDR < 0.05), a two‐sample MR analysis was performed to explore the potential causality between metabolites and blood pressure. GWASs for each metabolite were used as exposures. Blood pressure‐related public GWAS data from the BBJ, the KoGES, and the TWB were included as outcome in our study [64, 65, 66]. GWAS summary statistics for SBP and DBP were available from all three biobanks, and GWAS results for antihypertensive drugs and hypertension were obtained from BBJ and KoGES, respectively.

The IVs utilized in MR analysis should satisfy three fundamental assumptions: (1) IVs must exhibit a statistically significant association with the exposure (metabolites); (2) IVs should influence the outcome exclusively through the exposure (metabolites), with no direct or alternative pathways; and (3) IVs must be independent of any confounding factors [67].

GWAS was conducted using the mixed linear model‐based association analysis implemented in the Genome‐wide Complex Trait Analysis software (version 1.94.0 beta) with adjustment for age, sex, and the first 10 genetic principal components [68]. IVs for MR analysis were obtained by identifying linkage‐independent significant SNPs associated with each metabolite using the “–clump” function in PLINK v1.90b, with the following parameters: clump‐p1 1 × 10^−5^, clump‐r2 0.10, clump‐kb 1000 [69]. Metabo‐GWAS in the Liuheng and THSBC cohorts were conducted following the same analytical procedures.

MR analysis was conducted for metabolites with more than three IVs. Mainly, the estimates for potential causal effects were derived using inverse variance weighting (IVW) and MR‐Egger. The potential for horizontal pleiotropy was assessed using the intercept test in MR‐Egger [70]. Cochran's Q statistic was employed to assess heterogeneity among the IVs [71]. The potential causal effects should pass the pleiotropy and heterogeneity tests (*p* > 0.05). To mitigate the bias brought by weak IVs, MR‐RAPS was employed as a supplementary analytical method with default settings [72]. Based on blood pressure‐associated metabolites and ratios, we calculated the mean *F*‐statistics (*F*‐statistics > 10) to confirm there were no weak instruments. For each metabolite, the *F*‐statistic was first calculated for every instrumental SNP using *F* = (*β*/se)^2^, and then averaged to obtain a mean *F*‐statistic.

Metabolites that met the following criteria were considered to have a potential causal association with blood pressure: (1) *P*_IVW_ < 0.05; (2) the direction of association should be consistent with the GLM results for the corresponding blood pressure traits.

For replication analyses, we included overlapping metabolites from the THSBC. Based on the blood pressure‐associated metabolites supported by GLM and MR analyses, this study further conducted MR analyses (GWAS of metabolites from the THSBC cohort as exposures; GWAS of blood pressure traits from BBJ, KoGES, and TWB as outcomes) using the same methodology as described above.

Furthermore, reverse MR analysis was conducted to explore potential reverse causality (from blood pressure to metabolites) based on the findings of MR analysis. The GWAS significance threshold of selecting IV for blood pressure was set at *p *< 5.00 × 10^−8^. For the metabolite ratios identified above, both MR and reverse MR were performed with identical procedures.

#### 5.6.5 Multiomics Evidence for the Metabolite–Blood Pressure Association

To broaden the SNP–metabolite–trait associations using metabolites as anchors, we first conducted GWAS for blood pressure‐related metabolites to identify metaboQTLs. The lead metaboQTLs were identified by clumping (clump‐p1 5 × 10^−8^, clump‐p2 1, clump‐r2 0.10) [61]. Next, *cis*‐eQTLs from GTEx V10 database (liver, artery, heart, and whole blood tissues) and protein QTLs (pQTLs) from the UK Biobank (UKB, Europeans) and the Guangzhou Nutrition and Health Study (GNHS; East Asians: Han Chinese) were integrated with metaboQTLs [73, 74]. In the UKB, 2923 plasma proteins were included based on Olink panels, while the GNHS dataset included 304 proteins and 1298 peptides tested by data‐independent acquisition mass spectrometry.

For eQTLs, an FDR < 0.05 was applied as the significance criterion. The threshold for pQTLs was set at *p *< 1.70 × 10^−11^ (adjusted for 2923 unique proteins) for UKB and *p *< 5 × 10^−5^ for GNHS. Integrating eQTLs, pQTLs and metaboQTLs provided insights into potential molecular regulatory pathways initiated by genetic variation.

#### 5.6.6 RCS Analysis

RCS analyses were employed to evaluate potential nonlinear relationships. This part focused on blood pressure‐associated metabolites as well as ratios of metabolites with consistent observational and genetic evidence. All models were adjusted for age, sex, smoking status, drinking status, and BMI, and were fitted using three knots at the 25th, 50th, and 75th percentiles.

#### 5.6.7 Mediation Analysis

Mediation analysis was performed to examine the mediating impact of metabolites on modifiable lifestyle factors and blood pressure. Lifestyle factors included smoking, drinking, BMI, WC, diet (vegetables, fruits, red meat, and aquatic products and seafood), physical activity, sleep duration, and HLS. Continuous variables were inverse normal transformed. The blood pressure‐associated metabolites supported by both GLM and MR analysis were included. The associations of lifestyles–metabolites and lifestyles–blood pressure were assessed using GLMs adjusted for age and sex. Given that the previously identified potential causal relationships between metabolites and blood pressure were all based on SBP and DBP, these two phenotypes were selected as outcomes in this part. Lifestyle factors significantly associated with blood pressure were selected using an FDR < 0.05 threshold based on the number of 1601 metabolites for each blood pressure trait. Similarly, significant lifestyle–metabolite associations were corrected based on the number of 10 metabolites for each lifestyle, also applying an FDR < 0.05 threshold.

Mediation analysis was then performed using the “mediation” package in R. A significant mediation effect was defined by a mediation proportion > 5% and mediation *p* value < 0.05.

All statistical analyses were conducted using R 4.2.2 (The R Foundation for Statistical Computing, Vienna, Austria). Two‐sided *p* < 0.05 was considered statistically significant.

## Author Contributions

Conceptualization: Y.L., C.C., D.Z., and Y.Z. Supervision: X.P., Y.Z., and D.Z. Methodology: Y.L., C.C., D.Z., and Y.Z. Data collection: Y.L., C.C., and W.S. Data analysis: Y.L. and C.C. Writing – original draft: Y.L. Writing – review and editing: Y.L., C.C., Y.Z., and D.Z. Visualization: Y.L. and C.C. All authors read and approved the final manuscript.

## Funding Information

We thank the support from the Starry Night Science Fund of Zhejiang University Shanghai Institute for Advanced Study (SN‐ZJU‐SIAS‐0021, D.Z. and Y.Z.), the National Key Research and Development Program of China (2017YFC0907004, Y.Z.), and the National Natural Sciences Foundation of China (82204118, D.Z.).

## Ethics Statement

All participants provided informed consent. The ZMSC was approved by the Ethics Committee of the School of Public Health, Zhejiang University, China (ZGL202312‐7). The THSBC study was approved by the Ethics Committee of Tongji Medical College, Huazhong University of Science and Technology (2017‐S225‐1).

## Conflicts of Interest

The authors declare no conflicts of interest.

## Supporting information




**Figure S1**: Numbers of identified metabolite by classes. *Abbreviations*: FA, fatty acids; GP, glycerophospholipids; SL, sphingolipids; GL, glycerolipids.
**Figure S2**: Overlap of metabolites significantly associated with blood pressure. The Venn diagram showed the overlap of metabolites significantly associated with SBP, DBP, and hypertension. The numbers represent the count of metabolites with FDR < 0.05 for each trait and their intersections. 91 metabolites (FDR < 0.05 for any blood pressure trait) were identified as being robustly associated with any of blood pressure traits, including 37 metabolites for SBP, 81 for DBP, and nine for hypertension. Moreover, 6 metabolites were associated with all three blood pressure traits, 24 with two blood pressure traits, and 61 with a single blood pressure trait. *Abbreviations*: FDR, false discovery rate; SBP, systolic blood pressure; DBP, diastolic blood pressure.
**Figure S3**: Blood pressure‐associated metabolites. (A) Metabolites and SBP, (B) metabolites and DBP, and (C) metabolites and hypertension. Beta was calculated with multivariable linear regression models adjusting for age, sex, body mass index, smoking status and drinking status in participants without antihypertensive treatment. OR was calculated with multivariable logistic regression models adjusting for age, sex, body mass index, smoking status, drinking status, and antihypertensive drug. Due to space constraints, only the top 10 metabolites ranked by *p* values (with FDR < 0.05) are labeled in panels (A) and (B). Nine metabolites significantly associated with hypertension (FDR < 0.05) are all annotated in panel (C). *Abbreviations*: FDR, false discovery rate; SBP, systolic blood pressure; DBP, diastolic blood pressure; OR, odds ratio.
**Figure S4**: Pathway analysis for metabolites associated with blood pressure. The *X*‐axis represents pathway impact, and the *Y*‐axis represents −log10 (*p*). (A) Caffeine metabolism; (B) glycerophospholipid metabolism. *Abbreviations*: KEGG, Kyoto Encyclopedia of Genes and Genomes.
**Figure S5**: The Manhattan plot for prostaglandin E3. The *X*‐axis represents the chromosomal position, and the *Y*‐axis represents −log10 (*p*). The red line shows the *p* value threshold at 5.00 × 10^−8^.
**Figure S6**: The Manhattan plot for LPC (0:0/14:0). The *X*‐axis represents the chromosomal position, and the *Y*‐axis represents −log10 (*p*). The red line shows the *p* value threshold at 5.00 × 10^−8^. *Abbreviation*: LPC, lysophosphatidylcholine.
**Figure S7**: Testing for nonlinear association between metabolites and blood pressure. RCS analysis based on linear regression (SBP and DBP) and logistic regression (hypertension) were conducted with adjustment for age, sex, smoking status, drinking status, and BMI. The solid line is the estimated *β* values or OR, and the shaded area is the 95% CI. *Abbreviations*: RCS, restricted cubic spline analysis; SBP, systolic blood pressure; DBP, diastolic blood pressure; BMI, body mass index; OR, odds ratio; CI, confidence interval.
**Figure S8**: Testing for nonlinear association between ratios of metabolites and blood pressure. RCS analysis based on linear regression (SBP and DBP) and logistic regression (hypertension) were conducted with adjustment for age, sex, smoking status, drinking status and BMI. The solid line is the estimated *β* values or OR, and the shaded area is the 95% CI. *Abbreviations*: RCS, restricted cubic spline analysis; SBP, systolic blood pressure; DBP, diastolic blood pressure; BMI, body mass index; OR, odds ratio; CI, confidence interval.
**Figure S9**: Examples of lifestyle–metabolite–blood pressure mediation effects. (A) Association between BMI and WC and DBP mediated by l‐glutamic acid, (B) association between HLS and DBP mediated by l‐glutamic acid. l‐Glutamic acid mediated the association between BMI, WC, HLS, and DBP with the mediation proportions of 12.95, 12.16, and 31.53%, respectively. The rectangles with blue, yellow, and orange backgrounds indicate lifestyle, metabolites, and blood pressure, respectively. *Abbreviations*: BMI, body mass index; WC, waist circumference; DBP, diastolic blood pressure; HLS, healthy lifestyle score.
**Table S1**: Information for the 1601 unique serum metabolites profiled in Liuheng.
**Table S2**: Comparison of targeted metabolites measured in Xiazhi with metabolites in Liuheng.
**Table S3**: Comparison of metabolites measured in THSBC with metabolites in Liuheng.
**Table S4**: Blood pressure‐associated metabolites including those taking antihypertensive medication in generalized linear models.
**Table S5**: Blood pressure‐associated metabolites stratified by gender in generalized linear models.
**Table S6**: Replicated blood pressure‐associated metabolites in Xiazhi.
**Table S7**: Blood pressure change‐associated metabolite in generalized linear models.
**Table S8**: Summary of pathway analysis of blood pressure‐associated metabolites.
**Table S9**: *F*‐statistics for blood pressure‐associated metabolites and ratios in Liuheng.
**Table S10**: Replicated potential causal relationships of metabolites with blood pressure in THSBC.
**Table S11**: Potential causal relationships of blood pressure with metabolites in IVW analysis in Liuheng.
**Table S12**: Potential causal relationships of metabolites with blood pressure in MR‐RAP and Egger analysis in Liuheng.
**Table S13**: Potential causal relationships of blood pressure with metabolites in MR‐RAP and Egger analysis in Liuheng.
**Table S14**: Potential causal relationships of metabolites ratios with blood pressure in MR‐RAP and Egger analysis in Liuheng.
**Table S15**: Potential causal relationships of metabolites with blood pressure in MR‐RAPS and Egger analysis in THSBC.
**Table S16**: Summary for all identified independent metaboQTLs.
**Table S17**: The metaboQTLs overlapping with liver and eQTLs from the GTEx project.
**Table S18**: The metaboQTLs overlapping with serum pQTLs identified in the UK Biobank.
**Table S19**: Significant associations between metabolites and lifestyles.
**Table S20**: Significant associations between lifestyles and blood pressure.
**Table S21**: Mediation effects of metabolites in associations between lifestyle and blood pressure.

## Data Availability

The GWAS data for all metabolites analyzed in this study, including data from the ZMSC, were previously released and are publicly accessible through an interactive web resource (https://omics.lab.westlake.edu.cn/data/metabolites/) [61]. Summary statistics for the KoGES were obtained from the KoGES Zenodo repository (https://zenodo.org/record/7042518) [65]. Summary statistics for the Biobank Japan (BBJ) were sourced from the BBJ PheWeb (https://pheweb.jp/), and summary statistics for the TWB were retrieved from the GWAS Catalog (https://www.ebi.ac.uk/gwas/publications/38116116) [64, 66].
