# Supplementary material for: Identification of Blood Pressure‐Associated Metabolites by Integrating Metabolomic and Genetic Analysis
Source: MedComm (2020). 2026 Apr 1;7(4):e70718. doi: 10.1002/mco2.70718 (PMC13045211; doi:10.1002/mco2.70718)
Supplement: Supplementary file 1 — Figure S1: Numbers of identified metabolite by classes. Abbreviations: FA, fatty acids; GP, glycerophospholipids; SL, sphingolipids; GL, glycerolipids. Figure S2: Overlap of metabolites significantly associated with blood pressure. The Venn diagram showed the overlap of metabolites significantly associated with SBP, DBP, and hypertension. The numbers represent the count of metabolites with FDR < 0.05 for each trait and their intersections. 91 metabolites (FDR < 0.05 for any blood pressure trait) were identified as being robustly associated with any of blood pressure traits, including 37 metabolites for SBP, 81 for DBP, and nine for hypertension. Moreover, 6 metabolites were associated with all three blood pressure traits, 24 with two blood pressure traits, and 61 with a single blood pressure trait. Abbreviations: FDR, false discovery rate; SBP, systolic blood pressure; DBP, diastolic blood pressure. Figure S3: Blood pressure‐associated metabolites. (A) Metabolites and SBP, (B) metabolites and DBP, and (C) metabolites and hypertension. Beta was calculated with multivariable linear regression models adjusting for age, sex, body mass index, smoking status and drinking status in participants without antihypertensive treatment. OR was calculated with multivariable logistic regression models adjusting for age, sex, body mass index, smoking status, drinking status, and antihypertensive drug. Due to space constraints, only the top 10 metabolites ranked by p values (with FDR < 0.05) are labeled in panels (A) and (B). Nine metabolites significantly associated with hypertension (FDR < 0.05) are all annotated in panel (C). Abbreviations: FDR, false discovery rate; SBP, systolic blood pressure; DBP, diastolic blood pressure; OR, odds ratio. Figure S4: Pathway analysis for metabolites associated with blood pressure. The X‐axis represents pathway impact, and the Y‐axis represents −log10 (p). (A) Caffeine metabolism; (B) glycerophospholipid metabolism. Abbreviations: KEGG, Kyoto En [file MCO2-7-e70718-s001.docx]

# Title page

**Identification of Blood Pressure-Associated Metabolites by Integrating Metabolomic and Genetic Analysis**

Yuanjiao Liu^1^, Chunxiao Cheng^2,3^, Xiong-Fei Pan ^4,5,6^, Wei Shao^7*^, Dan Zhou^2,8*^, Yimin Zhu^1*^

^1^Department of Respiratory Disease, Sir Run Run Shaw Hospital, and Department of Epidemiology and Biostatistics, Zhejiang University School of Medicine, Hangzhou 310058, Zhejiang, China.

^2^The Second Affiliated Hospital and School of Public Health, Zhejiang University School of Medicine, Hangzhou 310058, China;^3^The Key Laboratory of Intelligent Preventive Medicine of Zhejiang Province, Hangzhou 310058, Zhejiang, China;

^4^Section of Epidemiology and Population Health & Department of Gynecology and Obstetrics, Ministry of Education Key Laboratory of Birth Defects and Related Diseases of Women and Children & Children's Medicine Key Laboratory of Sichuan Province, West China Second University Hospital, Sichuan University, Chengdu, China;

^5^West China Biomedical Big Data Center, West China Hospital, Sichuan University, Chengdu, China;

^6^Shuangliu Institute of Women’s and Children’s Health, Shuangliu Maternal and Child Health Hospital, Chengdu, China;

^7^Department of Internal Medicine, Putuo District People’s Hospital, Zhoushan 316100, Zhejiang, China.

^8^State Key Laboratory of Transvascular Implantation Devices, Hangzhou 310009, Zhejiang, China

Correspondence to:

Yimin Zhu, zhuym@zju.edu.cn (Lead Contact)

Wei Shao, shaowei_putuo01@126.com

Dan Zhou, danzhou@zju.edu.cn

**Supplementary Figures**

Figures S1-9

**Supplementary Methods**

**Supplementary Tables**

Tables S1-21

**Supplementary Figures**

**Figure S1. Numbers of identified metabolite by classes.**

**Figure S2. Overlap of metabolites significantly associated with blood pressure.**

**Figure S3. Blood pressure-associated metabolites.**

**Figure S4. Pathway analysis for metabolites associated with blood pressure.**

**Figure S5. The Manhattan plot for prostaglandin E3.**

**Figure S6. The Manhattan plot for LPC (0:0/14:0).**

**Figure S7. Testing for nonlinear association between metabolites and blood pressure.**

**Figure S8. Testing for nonlinear association between ratios of metabolites and blood pressure in ZMSC.**

**Figure S9. Examples of lifestyle-metabolite-blood pressure mediation effects.**


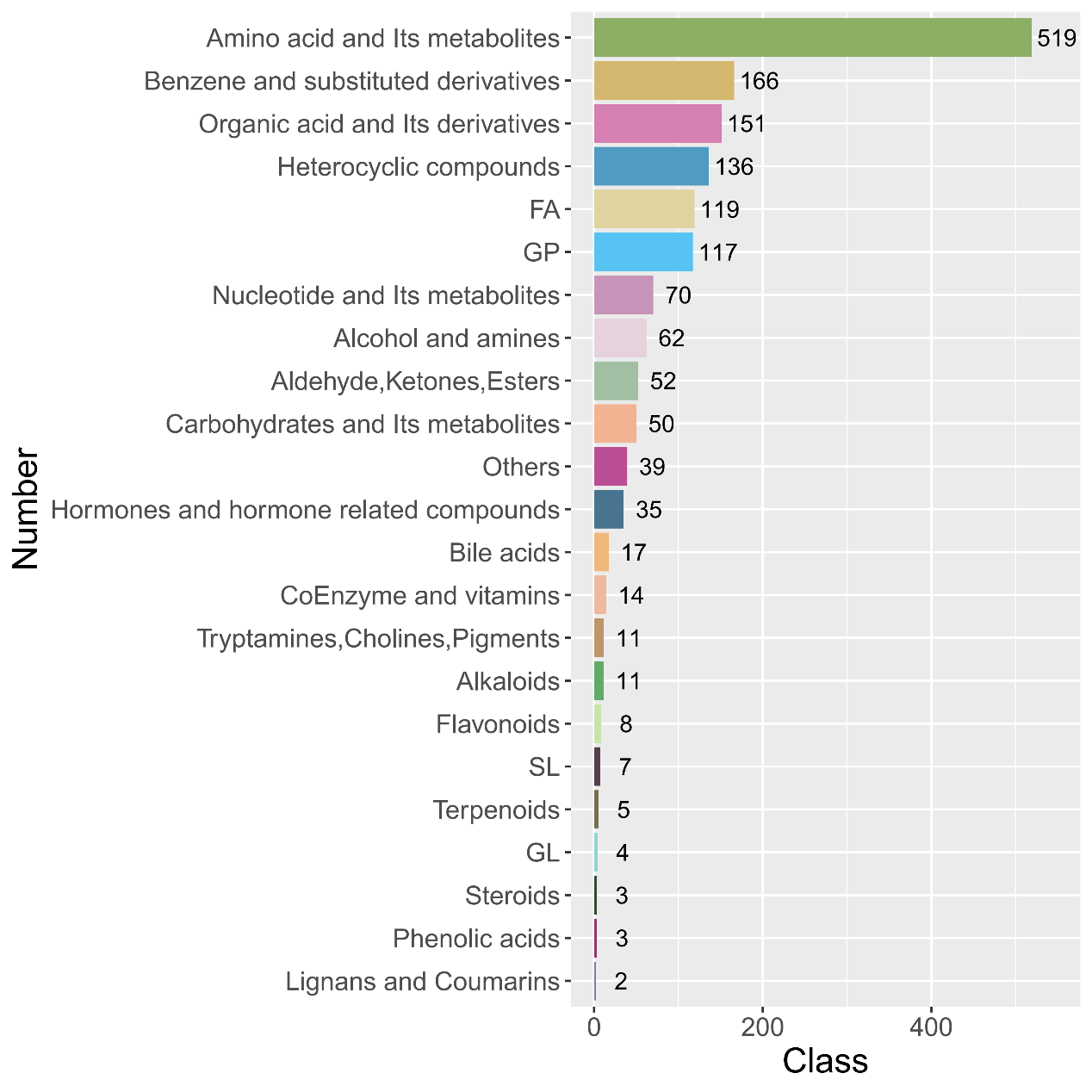


**Figure S1. Numbers of identified metabolite by classes.** FA, fatty acids; GP, glycerophospholipids; SL, sphingolipids; GL, glycerolipids.


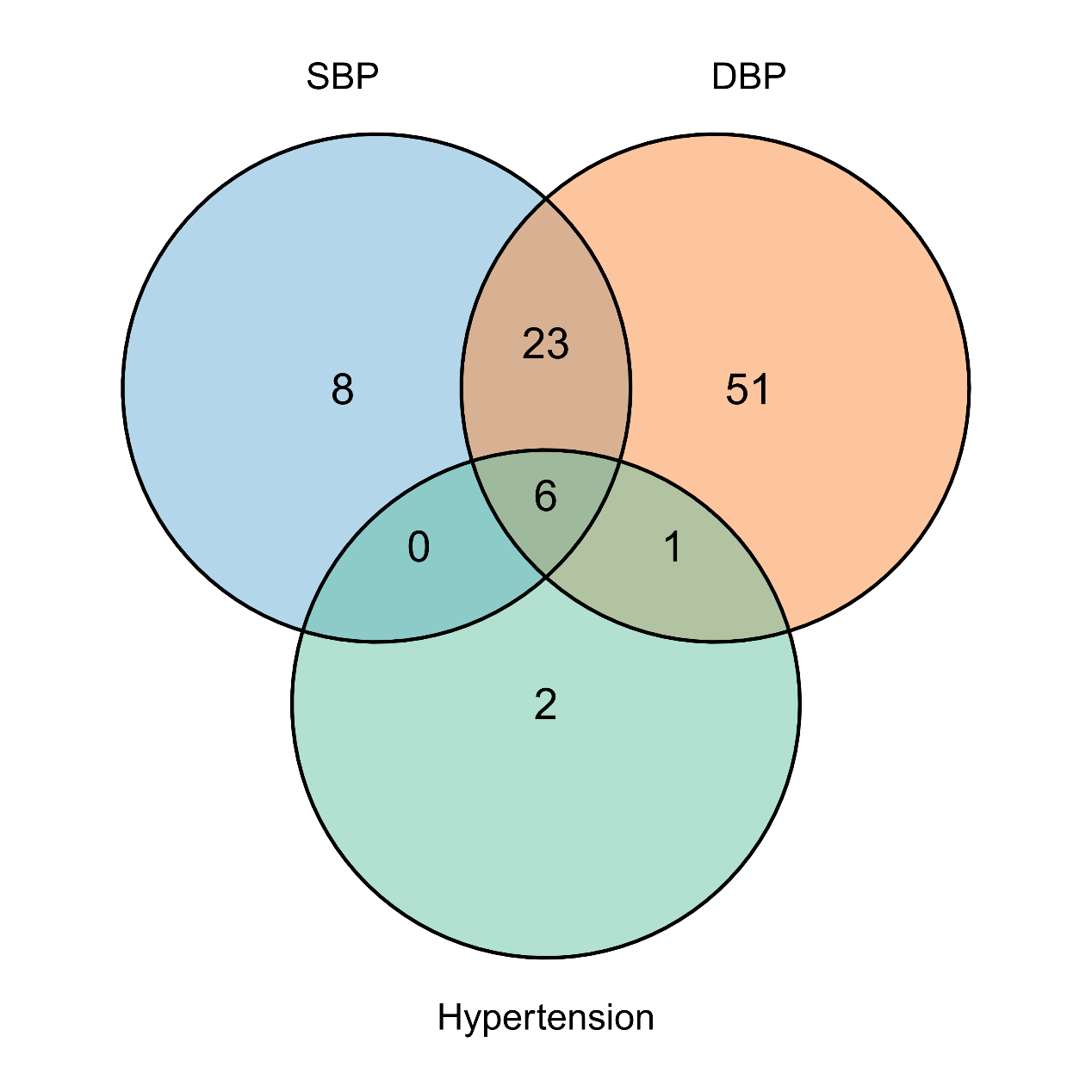


**Figure S2. Overlap of metabolites significantly associated with blood pressure.** The venn diagram showed the overlap of metabolites significantly associated with SBP, DBP, and hypertension. The numbers represent the count of metabolites with FDR < 0.05 for each trait and their intersections. 91 metabolites (FDR < 0.05 for any blood pressure trait) were identified as being robustly associated with any of blood pressure traits, including 37 metabolites for SBP, 81 for DBP, and nine for hypertension. Moreover, 6 metabolites were associated with all three blood pressure traits, 24 with two blood pressure traits, and 61 with a single blood pressure trait. FDR, false discovery rate; SBP, systolic blood pressure; DBP, diastolic blood pressure.


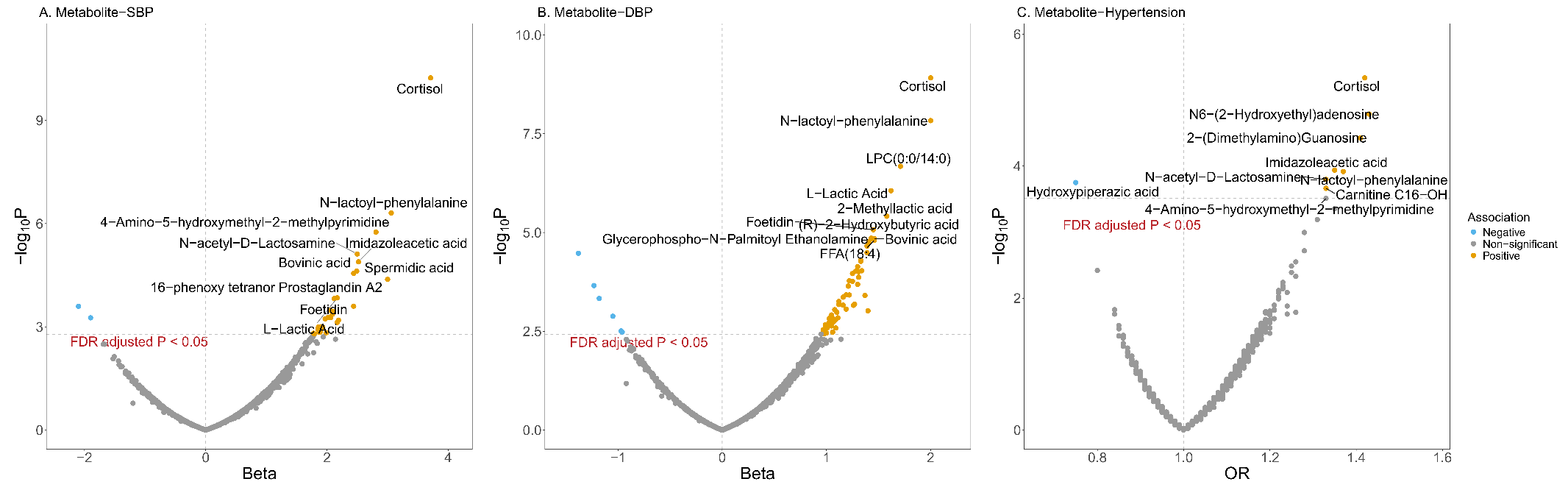


**Figure S3. Blood pressure-associated metabolites.** (A) metabolites and SBP, (B) metabolites and DBP, (C) metabolites and hypertension. Beta was calculated with multivariable linear regression models adjusting for age, sex, body mass index, smoking status and drinking status in participants without anti-hypertensive treatment. OR was calculated with multivariable logistic regression models adjusting for age, sex, body mass index, smoking status, drinking status, and anti-hypertensive drug. Due to space constraints, only the top ten metabolites ranked by *P*-values (with FDR < 0.05) are labeled in panels (A) and (B). Nine metabolites significantly associated with hypertension (FDR < 0.05) are all annotated in panel (C). FDR, false discovery rate; SBP, systolic blood pressure; DBP, diastolic blood pressure; OR, Odds ratio.


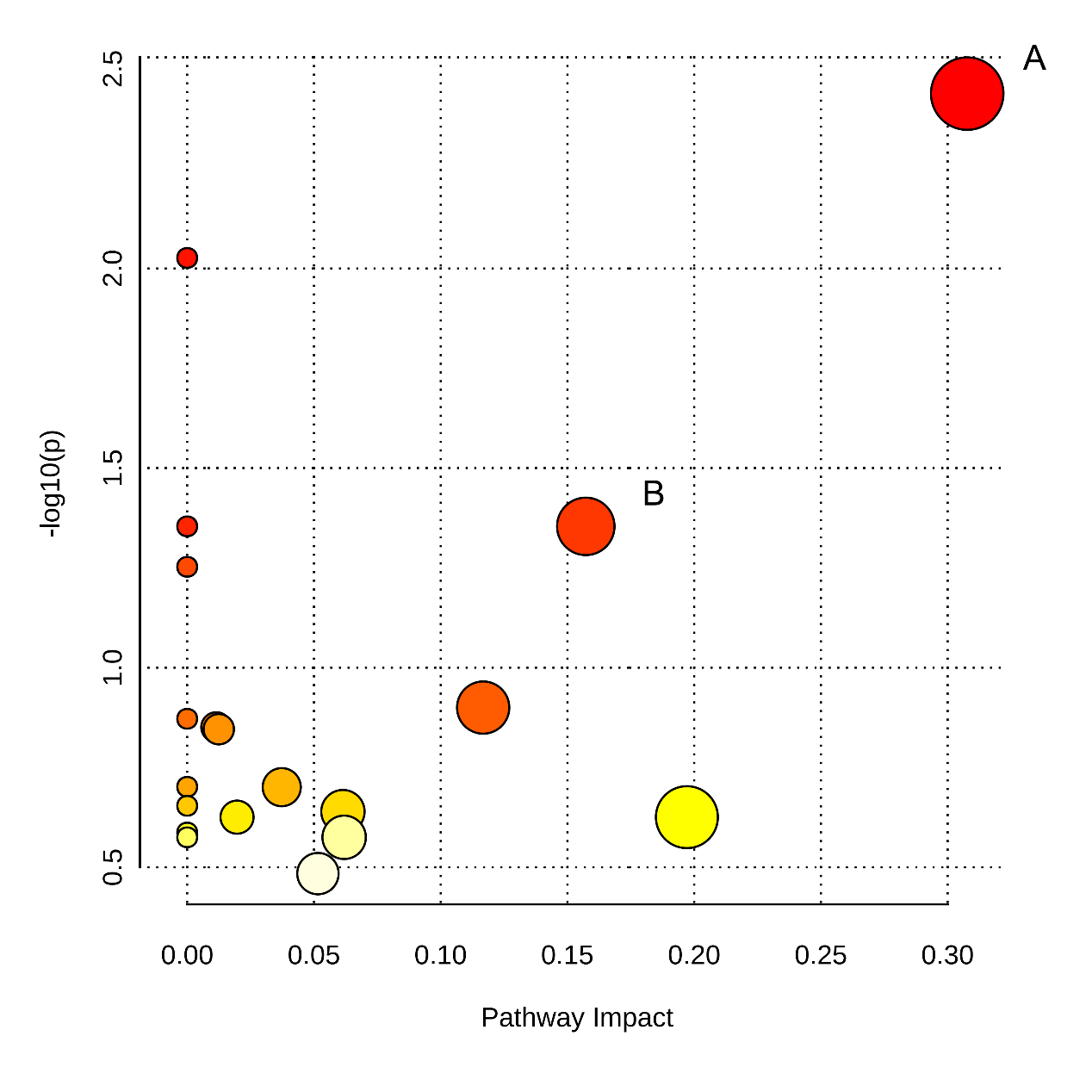


**Figure S4. Pathway analysis for metabolites associated with blood pressure.** The X-axis represents pathway impact, and the Y-axis represents -log10 (P). (A) caffeine metabolism; (B) glycerophospholipid metabolism. KEGG, Kyoto Encyclopedia of Genes and Genomes.


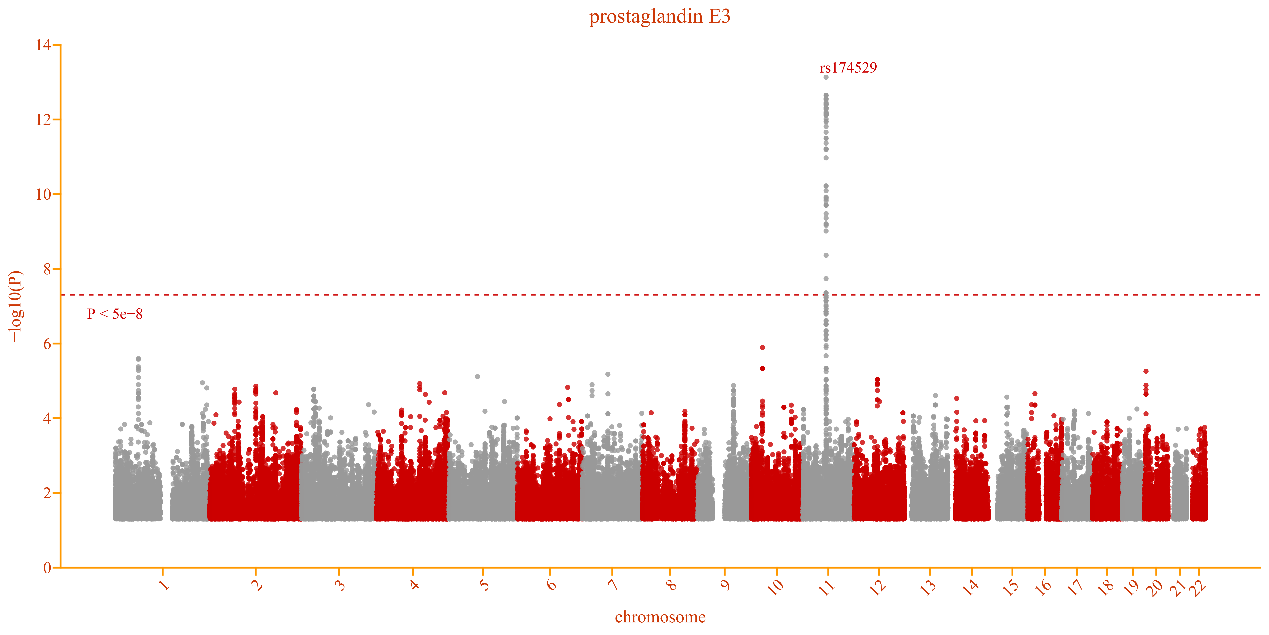


**Figure S5. The Manhattan plot for prostaglandin E3.** The X-axis represents the chromosomal position, and the Y-axis represents -log10 (P). The red line shows the *P-*value threshold at 5.00×10^-8^.


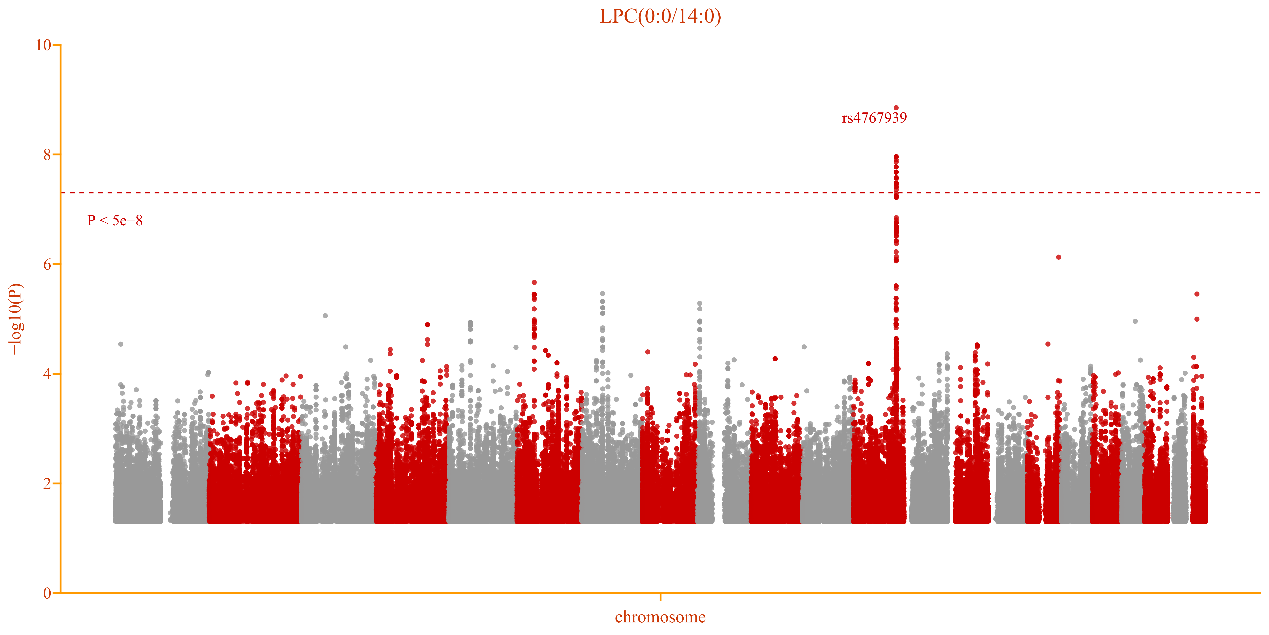


**Figure S6. The Manhattan plot for LPC (0:0/14:0).** The X-axis represents the chromosomal position, and the Y-axis represents -log10 (P). The red line shows the *P-*value threshold at 5.00×10^-8^. LPC, lysophosphatidylcholine.


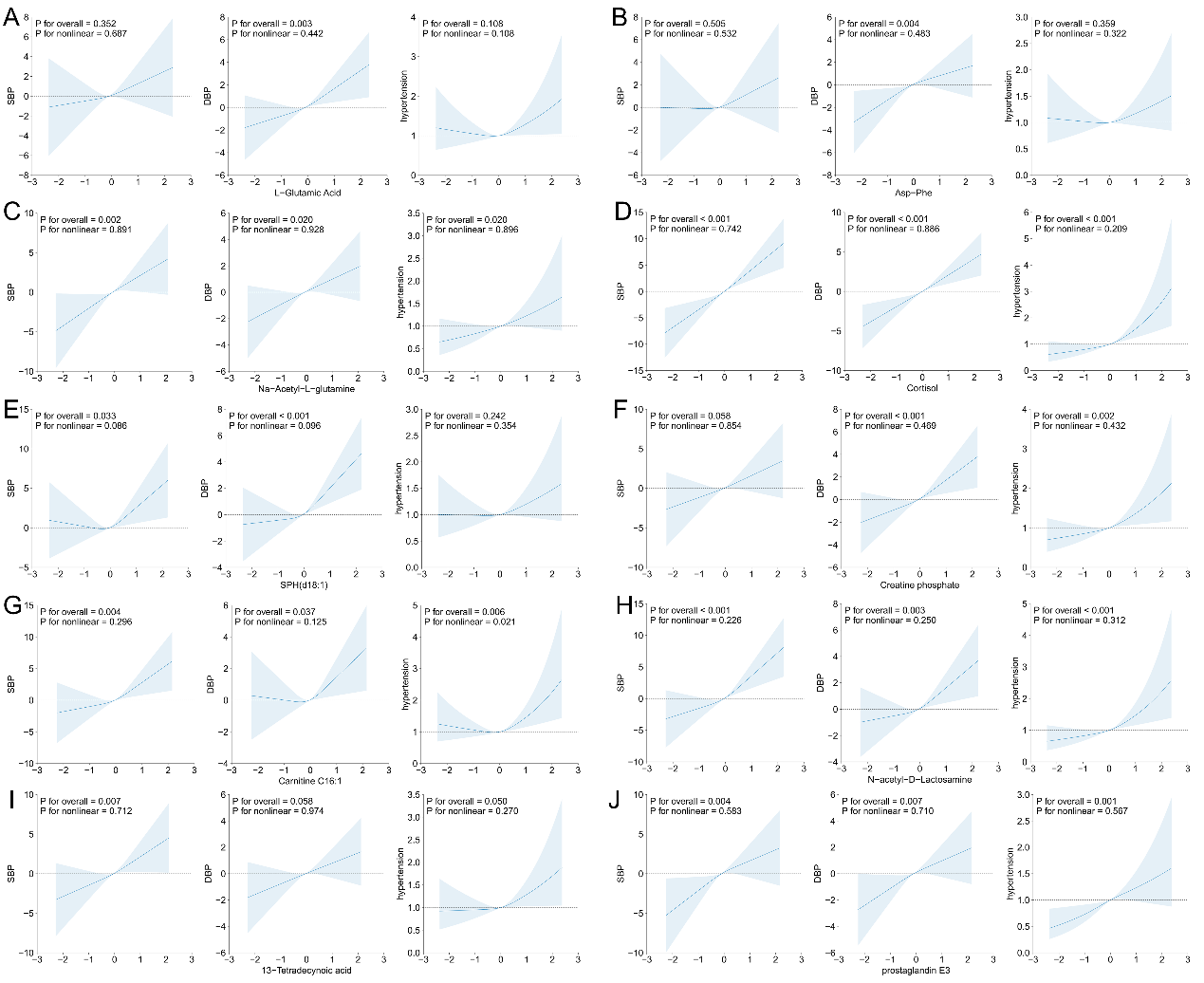
 **Figure S7. Testing for nonlinear association between metabolites and blood pressure.** RCS analysis based on linear regression (SBP and DBP) and logistic regression (hypertension) were conducted with adjustment for age, sex, smoking status, drinking status and BMI. The solid line is the estimated β values or OR, and the shaded area is the 95% CI. RCS, Restricted cubic spline analysis; SBP, systolic blood pressure; DBP, diastolic blood pressure; BMI, body mass index; OR, Odds ratio; CI, confidence interval.


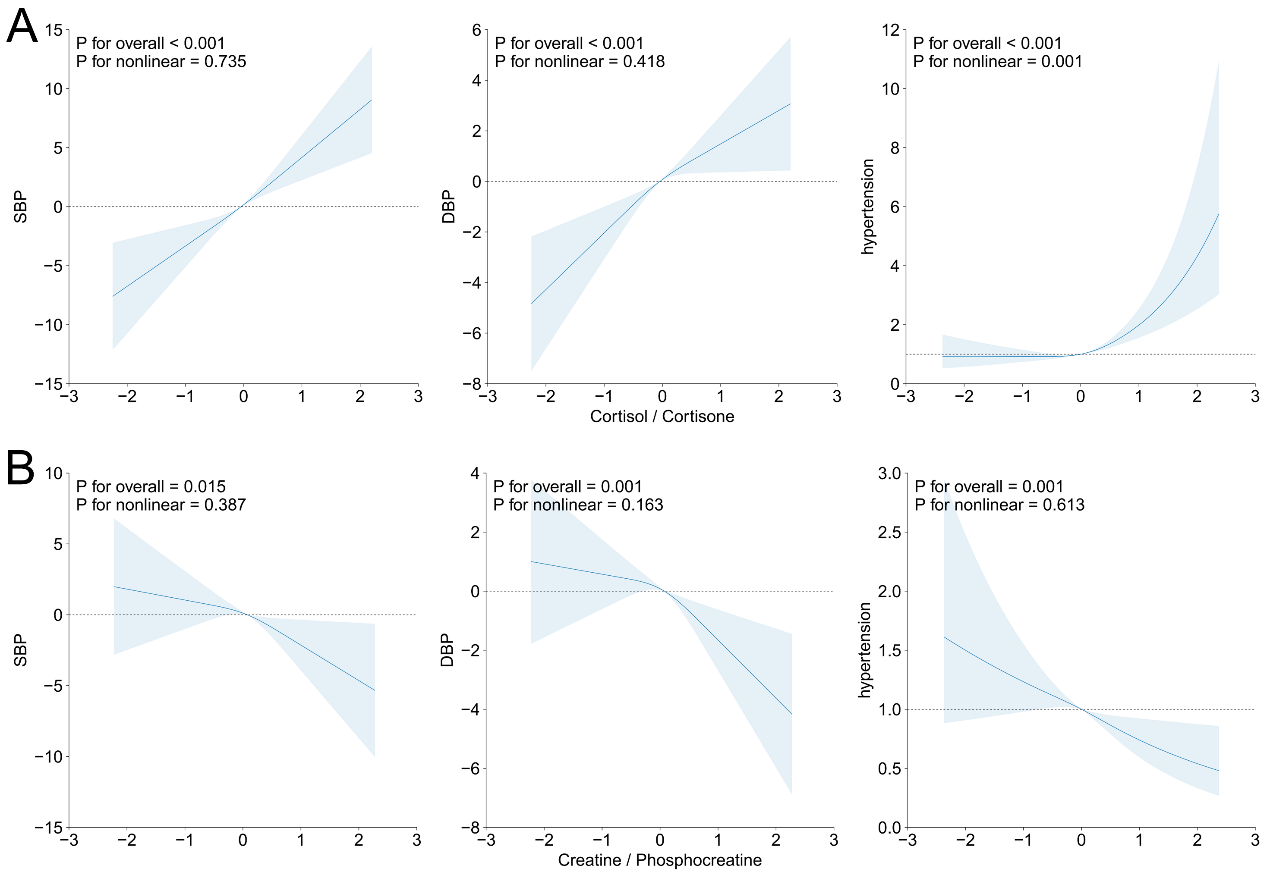


**Figure S8. Testing for nonlinear association between ratios of metabolites and blood pressure.** RCS analysis based on linear regression (SBP and DBP) and logistic regression (hypertension) were conducted with adjustment for age, sex, smoking status, drinking status and BMI. The solid line is the estimated β values or OR, and the shaded area is the 95% CI. RCS, Restricted cubic spline analysis; SBP, systolic blood pressure; DBP, diastolic blood pressure; BMI, body mass index; OR, Odds ratio; CI, confidence interval.


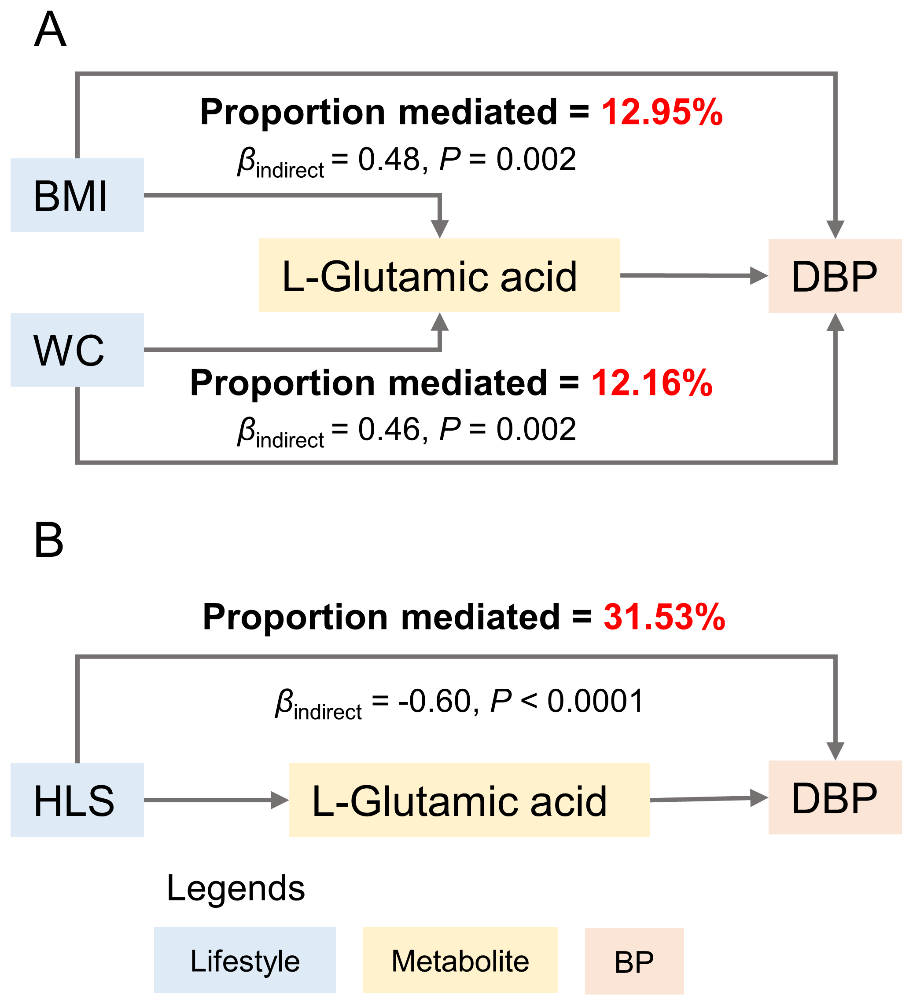


**Figure S9. Examples of lifestyle-metabolite-blood pressure mediation effects.** (A) Association between BMI and WC and DBP mediated by L-glutamic acid, (B) Association between HLS and DBP mediated by L-glutamic acid. L-glutamic acid mediated the association between BMI, WC, HLS and DBP with the mediation proportions of 12.95%, 12.16 and 31.53% and respectively. The rectangles with blue, yellow and orange backgrounds indicate lifestyle, metabolites and blood pressure, respectively. BMI, body mass index; WC, waist circumference; DBP, diastolic blood pressure; HLS, healthy lifestyle score.

**Supplementary Methods**

**Study population**

In brief, the ZMSC is an ongoing prospective community-based cohort study that was initiated in 2009 in Zhejiang Province, a coastal region in southeastern China. The study protocol incorporated comprehensive surveys through standardized epidemiological questionnaires, systematic clinical health examinations and routine biochemical assessments with collection of fasting blood and urine specimens. Fasting status was defined as no consumption of food or water after 8:00 p.m. on the day preceding the survey.

Moreover, replication of metabolome analysis was performed using targeted metabolomics approach in another sub-population of the ZMSC, namely Xiazhi with 1,419 individuals having data on blood pressure and metabolites. For replication of MR analysis, this study utilized GWAS data comprising 484 overlapping metabolites from the THSBC. The THSBC cohort consists of 869 pregnant women aged 18-40 years who attended their first antenatal visit at Shuangliu Maternal and Child Health Hospital during early pregnancy (gestational weeks 6-15) [1]. Notably, the metabolite profiling in the THSBC was limited to widely targeted metabolomics.

All participants provided informed consent. The ZMSC was approved by the Ethics Committee of the School of Public Health, Zhejiang University, China (ZGL202312-7). The THSBC study was approved by the Ethics Committee of Tongji Medical College, Huazhong University of Science and Technology (2017-S225-1).

**Calculation of HLS**

HLS was determined by five lifestyle factors based on previous studies and Dietary Guidelines for Chinese Residents including smoking, drinking, physical activity, diet, and body composition ^[68,69]^. In the present study, never smoking and never drinking was defined as low-risk. High physical activity, defined as the highest sex-specific quartile based on the MET-h/week calculation, was considered low-risk. For diet, a healthy diet score was generated containing three components: fruits, vegetables and red meat. A healthy diet, categorized as low-risk, was defined as meeting at least two optimal dietary criteria: being in the highest quartile for fruit and vegetable intake and the lowest quartile for red meat consumption. Body composition was assessed using WC as an indicator of abdominal obesity, with low-risk thresholds defined as <90 cm for males and <85 cm for females^[70]^.

The overall HLS was calculated by summing the number of low-risk lifestyle behaviors for each participant. This composite score, ranging from 0 to 5, reflected an individual's adherence to healthy lifestyle patterns, with higher scores indicating more favorable lifestyles.

**Quantitative analysis and quality control of metabolites measurement**

Blood samples of were collected after an 8-hour overnight fast and stored at -80°C. For untargeted detection of samples from Liuheng, the data acquisition instrument system included mainly ultra-high-performance liquid chromatography (UPLC, ExionLC AD, https://sciex.com.cn/) and quadrupole time-of-flight mass spectrometry (Q-TOF-MS, TripleTOF 6600, AB SCIEX). The UPLC and Tandem mass spectrometry (MS/MS, QTRAP®, <https://sciex.com>) systems were used for widely targeted detection. The metabolites detection was conducted by Wuhan Metware Biotechnology Co., Ltd.

For samples in Liuheng sub-cohort, Qualitative analysis of the precursor ion and fragments spectra was performed based on self-built databases, such as METLIN, the Human Metabolome Database (HMDB), and the Kyoto Encyclopedia of Genes and Genomes (KEGG)[2-4]. Mass spectrometry data were processed using Analyst 1.6.3 software (AB Sciex). For quantification, multiple reaction monitoring (MRM) mode was applied using triple quadrupole mass spectrometry.

For quality control, raw signals with a coefficient of variation (CV) ≥ 50% were excluded from the analysis. Metabolites with missing values ≥ 50% within a group were replaced with zero, while those with missing rates < 50% were imputed using the k-nearest neighbors (KNN) algorithm. To minimize potential batch effects, data were adjusted and integrated using the support vector regression (SVR) algorithm.

For external replication of a few metabolites, targeted metabolomics profiling of fasting serum was performed using high-performance liquid chromatography-tandem mass spectrometry (UPLC-MS/MS) at Shanghai Applied Protein Technology Co., Ltd in the Xiazhi sub-cohort. Dichloromethane was used to extract the serum metabolites. The detailed chromatographic conditions and MS parameters have been described elsewhere^[5]^.

A total of 59 metabolites were detected and 32 metabolites could be matched with that of Liuheng sub-cohort (Table S2). Metabolite levels underwent a similarly standardized preprocessing pipeline, which included log transformation to normalize the data distribution, removal of outliers exceeding 5 SD to minimize extreme value influence, and application of inverse normal transformation to ensure data normality for downstream analyses.

**Association analysis of external validation**

Among the 91 blood pressure-associated metabolites identified in the Liuheng sub-cohort, 36 metabolite pairs were consistently matched in both cohorts. For external validation in the Xiazhi sub-cohort, we applied the same analytical models used in Liuheng. The false discovery rate (FDR)-adjusted *P* values for the Xiazhi cohort were calculated specifically for these 36 overlapping metabolites.

Metabolites were considered successfully validated if they met two predefined replication criteria: (1) significant at a threshold of nominal *P* < 0.05 and (2) consistency in the direction of association effects across two independent analyses.

Reference

[1] Wang Y, Huang Y, Wu P, et al. Plasma lipidomics in early pregnancy and risk of gestational diabetes mellitus: a prospective nested case-control study in Chinese women[J]. Am J Clin Nutr, 2021, 114(5): 1763-1773.

[2] Guijas C, Montenegro-Burke J R, Domingo-Almenara X, et al. METLIN: A Technology Platform for Identifying Knowns and Unknowns[J]. Anal Chem, 2018, 90(5): 3156-3164.

[3] Wishart D S, Tzur D, Knox C, et al. HMDB: the Human Metabolome Database[J]. Nucleic Acids Res, 2007, 35(Database issue): D521-6.

[4] Kanehisa M, Goto S. KEGG: kyoto encyclopedia of genes and genomes[J]. Nucleic Acids Res, 2000, 28(1): 27-30.

[5] Wu Q, Li J, Sun X, et al. Multi-stage metabolomics and genetic analyses identified metabolite biomarkers of metabolic syndrome and their genetic determinants[J]. EBioMedicine, 2021, 74: 103707.

**Supplementary Tables**

**Table S1. Information for the 1,601 unique serum metabolites profiled in Liuheng**

**Table S2. Comparison of targeted metabolites measured in Xiazhi with metabolites in Liuheng**

**Table S3. Comparison of metabolites measured in THSBC with metabolites in Liuheng**

**Table S4. Blood pressure associated metabolites including those taking antihypertensive medication in generalized linear models**

**Table S5. Blood pressure associated metabolites stratified by gender in generalized linear models**

**Table S6. Replicated Blood pressure-associated metabolites in Xiazhi**

**Table S7. Blood pressure change associated metabolite in generalized linear models**

**Table S8. Summary of pathway analysis of blood pressure associated metabolites**

**Table S9. F-statistics for blood pressure-associated metabolites and ratios in Liuheng**

**Table S10. Replicated potential causal relationships of metabolites with blood pressure in THSBC**

**Table S11. Potential causal relationships of blood pressure with metabolites in IVW analysis in Liuheng**

**Table S12. Potential causal relationships of metabolites with blood pressure in MR-RAP and Egger analysis in Liuheng**

**Table S13. Potential causal relationships of blood pressure with metabolites in MR-RAP and Egger analysis in Liuheng**

**Table S14. Potential causal relationships of metabolites ratios with blood pressure in MR-RAP and Egger analysis in Liuheng**

**Table S15. Potential causal relationships of metabolites with blood pressure in MR-RAPS and Egger analysis in THSBC**

**Table S16. Summary for all identified independent metaboQTLs**

**Table S17. The metaboQTLs overlapping with liver and eQTLs from the GTEx project**

**Table S18. The metaboQTLs overlapping with serum pQTLs identified in the UK Biobank**

**Table S19. Significant associations between metabolites and lifestyles**

**Table S20. Significant associations between lifestyles and blood pressure**

**Table S21. Mediation effects of metabolites in associations between lifestyle and blood pressure**

| **Table S1. Information for the 1,601 unique serum metabolites profiled in Liuheng** | | | | |
| --- | --- | --- | --- | --- |
| Compound | Class I | Class II | Level | HMDB |
| (+)-Aschantin | Lignans and Coumarins | Lignans | 2 | - |
| (1-(4-Cyanobutyl)-3-(naphthalen-1-oyl)indole) | Heterocyclic compounds | Indole and Its derivatives | 2 | - |
| (1-hexyl-5-phenyl-1H-pyrrol-3-yl)(naphthalen-1-yl)methanone | Benzene and substituted derivatives | Benzene and substituted derivatives | 2 | - |
| (10R,13S,17S)-10,13-Dimethyl-3,6-dioxo-2,3,6,7,8,9,10, 11,12,13,14,15,16,17-tetradecahydro-1H-cyclopenta[a]phenanthren-17-yl heptanoate | Aldehyde,Ketones,Esters | Esters | 2 | - |
| (1E,4Z,6E)-5-hydroxy-1,7-bis(4-hydroxyphenyl)hepta-1,4,6-trien-3-one | Aldehyde,Ketones,Esters | Ketones | 2 | - |
| (1R,2R,5R,8R,9S,10R,12S)-12-Hydroxy-11-methyl-6-methylidene-16-oxo-15-oxapentacyclo[9.3.2.15,8.01,10.02,8]heptadecane-9-carboxylic acid | Heterocyclic compounds | Heterocyclic compounds | 2 | - |
| (1R,6S)-6-amino-5-oxocyclohexyl-2-ene-1-carboxylic-acid-ester | Aldehyde,Ketones,Esters | Esters | 2 | - |
| (2-Hydroxy-2-oxo-1,2lambda5-oxaphospholan-5-yl)methyl (Z)-octadec-9-enoate | FA | FFA | 2 | - |
| (24S)-24,25-dihydroxyvitamin D3 | CoEnzyme and vitamins | CoEnzyme and vitamins | 2 | - |
| (2R)-1-O-beta-D-Galactopyranosylglycerol | Carbohydrates and Its metabolites | Carbohydrates and Its metabolites | 2 | HMDB0038664 |
| (2R)-2-[[4-(2,6-dipyrrolidin-1-ylpyrimidin-4-yl)piperazin-1-yl]methyl]-2,5,7,8-tetramethyl-3,4-dihydrochromen-6-ol;dihydrochloride | Others | Others | 2 | - |
| (2R,6S,7R,9R,12S,16S)-6-hydroxy-15-[(1S)-1-[5-(hydroxymethyl)-4-methyl-6-oxo-2,3-dihydropyran-2-yl]ethyl]-2,16-dimethyl-8-oxapentacyclo[9.7.0.02,7.07,9.012,16]octadec-4-en-3-one | Others | Others | 2 | - |
| (2S)-2-[[(2S)-2-formamido-4-methylsulfanylbutanoyl]amino]propanoic acid | Heterocyclic compounds | Heterocyclic compounds | 2 | - |
| (2Z,4E)-5-[(1S)-1-hydroxy-2,6,6-trimethyl-4-oxocyclohex-2-en-1-yl]-3-methylpenta-2,4-dienoic acid | Organic acid and Its derivatives | Organic acid and Its derivatives | 2 | HMDB0035140 |
| (2s)-2-Amino-4-sulfinobutanoic acid | Amino acid and Its metabolites | Amino acid derivatives | 2 | - |
| (3S,3aS,6R,8aS)-3,8-Dimethyl-5-(propan-2-ylidene)-2,3,4,5,6,8a-hexahydro-1H-3a,6-epoxyazulen-6-ol | Alcohol and amines | Alcohols | 2 | HMDB0033960 |
| (4Z,7Z,10Z,13Z,16Z,19Z)-Eicosahexaenoate | Aldehyde,Ketones,Esters | Esters | 2 | - |
| (5S,6R)-5,6-dihydroxyicosa-7,9,11,14-tetraenoic acid | Organic acid and Its derivatives | Organic acid and Its derivatives | 2 | - |
| (6R,7R)-7-[[(2S)-2-azanyl-2-phenyl-ethanoyl]amino]-3-chloranyl-8-oxidanylidene-5-thia-1-azabicyclo[4.2.0]oct-2-ene-2-carboxylic acid | Others | Medicine | 2 | HMDB0014971 |
| (6aR,10aR)-9-(hydroxymethyl)-6,6-dimethyl-3-(2-methyloctan-2-yl)-6a,7,10,10a-tetrahydrobenzo[c]chromen-1-ol | Benzene and substituted derivatives | Benzene and substituted derivatives | 2 | - |
| (9Z)-N-[2-(5-hydroxy-1H-indol-3-yl)ethyl]octadec-9-enamide | Tryptamines,Cholines,Pigments | Tryptamines | 2 | - |
| (E)-11-methyldodec-2-enoic acid | FA | FFA | 2 | - |
| (E)-Guggulsterone | CoEnzyme and vitamins | CoEnzyme and vitamins | 2 | - |
| (E,Z)-2-Amino-3,14-octadecadien-1-ol | Alcohol and amines | Alcohols | 1 | - |
| (R)-(-)-1-Amino-2-propanol | Alcohol and amines | Amines | 1 | - |
| (R)-(-)-2-Phenylpropionic Acid | Organic acid and Its derivatives | Organic acid and Its derivatives | 1 | - |
| (R)-(-)-2-phenylglycine | Amino acid and Its metabolites | Amino acid derivatives | 1 | - |
| (R)-2-Hydroxy-3-phenylpropionic acid | Organic acid and Its derivatives | Organic acid and Its derivatives | 2 | HMDB0000563 |
| (R)-2-Hydroxybutyric acid | Organic acid and Its derivatives | Organic acid and Its derivatives | 1 | - |
| (R)-2-hydroxystearic acid | FA | Oxidized lipids | 2 | - |
| (R)-3-Hydroxy-tetradecanoic acid | Organic acid and Its derivatives | Organic acid and Its derivatives | 2 | HMDB0010731 |
| (R)-4-((3S,5R,8R,9S,10S,13R,14S,17R)-3-hydroxy-4,4,10,13,14-pentamethyl-7,11-dioxohexadecahydro-1H-cyclopenta[a]phenanthren-17-yl)pentanoic acid | Others | Others | 2 | - |
| (S)-2,3-Dihydro-5,7-dihydroxy-2-(4-hydroxyphenyl)-6,8-dimethyl-4-benzopyrone | Heterocyclic compounds | Heterocyclic compounds | 2 | HMDB0130571 |
| (S)-Leucic acid | Organic acid and Its derivatives | Organic acid and Its derivatives | 2 | HMDB0000746 |
| (Z)-2-tetracos-15-enamidoethanesulfonic acid | Others | Others | 2 | - |
| (±)12-HETE | FA | Oxidized lipids | 1 | HMDB0006111 |
| (±)13-HpODE | FA | Oxidized lipids | 1 | HMDB0003871 |
| (±)5-HETE | FA | Oxidized lipids | 2 | HMDB0011134 |
| 1,2-Dihexanoyl-sn-glycero-3-phosphocholine | GP | PC | 2 | - |
| 1,2-di-[(11Z)-eicosenoyl]-sn-glycero-3-phosphocholine | GP | PC | 2 | HMDB0008308 |
| 1,3-Benzenediol, 2-[3-methyl-6-(1-methylethenyl)-2-cyclohexen-1-yl]-5-pentyl-, (1R-trans)- | Alcohol and amines | Alcohols | 2 | - |
| 1,3-Diaminopropane | Alcohol and amines | Amines | 2 | HMDB0000002 |
| 1,3-Dicyclohexylurea | Alcohol and amines | Polyamines | 2 | - |
| 1,4-Dihydro-1-Methyl-4-Oxo-3-Pyridinecarboxamide | Heterocyclic compounds | Pteridines and derivatives | 1 | HMDB0004194 |
| 1,5-Diaminopentane | Alcohol and amines | Polyamines | 2 | HMDB0002322 |
| 1,6-anhydro-β-D-glucose | Carbohydrates and Its metabolites | Sugars | 2 | HMDB0000640 |
| 1,7-Dimethylxanthine | Nucleotide and Its metabolites | Nucleotide and Its metabolites | 1 | HMDB0001860 |
| 1-(1Z-hexadecenyl)-sn-glycero-3-phosphocholine | GP | LPC-P | 2 | HMDB0010407 |
| 1-Aminocyclobutanecarboxylic acid | Amino acid and Its metabolites | Amino acid derivatives | 1 | - |
| 1-Aminocyclohexanecarboxylic acid | Organic acid and Its derivatives | Organic acid and Its derivatives | 1 | - |
| 1-Aminocyclopropane-1-carboxylic acid | Organic acid and Its derivatives | Organic acid and Its derivatives | 1 | - |
| 1-Aminopentadecane | Organic acid and Its derivatives | Organic acid and Its derivatives | 1 | - |
| 1-Aminopropan-2-ol | Alcohol and amines | Alcohols | 2 | HMDB0012136 |
| 1-Deoxyvaleric acid | Organic acid and Its derivatives | Organic acid and Its derivatives | 1 | - |
| 1-Fluorocyclohexadiene-cis,cis-1,2-diol | Alcohol and amines | Alcohols | 2 | - |
| 1-Hexadecyl-2-butyryl-sn-glycero-3-phosphocholine | GP | PA | 2 | - |
| 1-Hexadecyl-sn-glycerol 3-phosphate | GP | LPA | 2 | HMDB0062325 |
| 1-Hydroxylamino-2-phenylethane | Benzene and substituted derivatives | Benzene and substituted derivatives | 1 | - |
| 1-Methyladenosine | Nucleotide and Its metabolites | Nucleotide and Its metabolites | 1 | - |
| 1-Methylguanine | Nucleotide and Its metabolites | Nucleotide and Its metabolites | 1 | HMDB0003282 |
| 1-Methylguanosine | Nucleotide and Its metabolites | Nucleotide and Its metabolites | 1 | HMDB0001563 |
| 1-Methylhistidine | Amino acid and Its metabolites | Amino acid derivatives | 1 | HMDB0000001 |
| 1-Methylinosine | Nucleotide and Its metabolites | Nucleotide and Its metabolites | 1 | HMDB0002721 |
| 1-Methylpiperidine-2-carboxylic acid | Amino acid and Its metabolites | Amino acid derivatives | 2 | - |
| 1-Methylxanthine | Nucleotide and Its metabolites | Nucleotide and Its metabolites | 1 | HMDB0010738 |
| 1-Myristoyl-2-stearoyl-sn-glycero-3-phosphocholine | GP | PC | 2 | HMDB0006727 |
| 1-Naphthylamine | Benzene and substituted derivatives | Benzene and substituted derivatives | 1 | - |
| 1-O-(cis-9-Octadecenyl)-2-O-acetyl-sn-glycero-3-phosphocholine | GP | PC | 2 | - |
| 1-O-Hexadecyl-2-O-ethyl-SN-glycero-3-phosphorylcholine | GP | PC | 2 | - |
| 1-O-Hexadecyl-2-deoxy-2-thio-S-acetyl-sn-glyceryl-3-phosphorylcholine | Organic acid and Its derivatives | Phosphoric acids | 2 | - |
| 1-O-Hexadecyl-lyso-sn-glycero-3-phosphocholine | GP | LPC | 2 | - |
| 1-O-Hexadecyl-sn-glycero-3-phosphocholine | GP | PC | 1 | - |
| 1-O-vanillyl-β-D-glucose | Benzene and substituted derivatives | Benzene and substituted derivatives | 2 | - |
| 1-Oleoyl lysophosphatidic acid sodium salt | GP | PA | 2 | - |
| 1-Phenylpiperazine | Heterocyclic compounds | Heterocyclic compounds | 2 | HMDB0243993 |
| 1-Stearoyl-2-arachidonoyl-sn-glycero-3-phospho-(1'-myo-inositol) | GP | PI | 2 | HMDB0009815 |
| 1-[1-(4-fluorophenyl)-2,5-dimethyl-1H-pyrrol-3-yl]-2-(pyrrolidin-1-yl)ethan-1-one | Aldehyde,Ketones,Esters | Ketones | 2 | - |
| 1-acetylindole | Heterocyclic compounds | Indole and Its derivatives | 1 | - |
| 1-beta-D-Galactosylsphingosine | SL | SL | 2 | HMDB0000648 |
| 1-desoxymethylsphinganine | SL | SM | 2 | - |
| 1-methylhistamine | Alcohol and amines | Polyamines | 1 | HMDB0000898 |
| 1-pyrroline-4-hydroxy-2-carboxylate | Heterocyclic compounds | Heterocyclic compounds | 1 | HMDB0002234 |
| 10-Hydroxycamptothecin | Alkaloids | Alkaloids | 2 | - |
| 10-Hydroxydecanoic acid | Organic acid and Its derivatives | Organic acid and Its derivatives | 2 | HMDB0244272 |
| 10-Hydroxystearic Acid | FA | Oxidized lipids | 2 | HMDB0037396 |
| 10-deoxyformamycin | Others | Others | 1 | - |
| 11-Carbonyl-beta-acetyl-boswellic acid | Organic acid and Its derivatives | Organic acid and Its derivatives | 1 | HMDB0036672 |
| 11-Deoxyprostaglandin F1alpha | Hormones and hormone related compounds | Hormones and hormone related compounds | 2 | - |
| 11-Oxoursolic acid acetate | Aldehyde,Ketones,Esters | Esters | 2 | - |
| 11-dehydro-TXB3 | Hormones and hormone related compounds | Hormones and hormone related compounds | 2 | - |
| 11-deoxy-PGE2 | Hormones and hormone related compounds | Hormones and hormone related compounds | 2 | - |
| 12,13-DiHOME | FA | Oxidized lipids | 1 | - |
| 12-Hydroxyjasmonic acid | FA | Oxidized lipids | 2 | - |
| 12-Hydroxyoctadecanoic acid | FA | FFA | 1 | HMDB0061706 |
| 12-Oxo-phytodienoic acid | FA | Oxidized lipids | 2 | HMDB0301804 |
| 12-ketolithocholic acid | Bile acids | Bile acids | 1 | HMDB0000328 |
| 13(R)-HODE | FA | Oxidized lipids | 1 | - |
| 13-Tetradecynoic acid | FA | Oxidized lipids | 2 | - |
| 15(S)-HETE-biotin | Heterocyclic compounds | Heterocyclic compounds | 2 | - |
| 16-phenoxy tetranor Prostaglandin A2 | Hormones and hormone related compounds | Hormones and hormone related compounds | 2 | - |
| 17-Ketostypolhydroperoxide | FA | Others | 2 | - |
| 18-AMINOABIETA-8,11,13-TRIENE | Others | Others | 2 | - |
| 19-Noretiocholanolone | Aldehyde,Ketones,Esters | Ketones | 2 | HMDB0005886 |
| 19-[(2S,3S)-3,6-Dihydro-3-methyl-6-oxo-2H-pyran-2-yl]-17-ethyl-6-hydroxy-3,5S,7S,9R,11,15R,hexamethyl-8-oxo-2E,10E,12E,16Z,18E-nonadecapentaenoic acid | FA | Others | 2 | - |
| 2',4'-Dihydroxyacetophenone | Benzene and substituted derivatives | Benzene and substituted derivatives | 2 | HMDB0029659 |
| 2'-Aenylic Acid | Nucleotide and Its metabolites | Nucleotide and Its metabolites | 2 | - |
| 2'-Hydroxy-5'-methylacetophenone | Benzene and substituted derivatives | Benzene and substituted derivatives | 2 | HMDB0032592 |
| 2'-O-Methylguanosine | Nucleotide and Its metabolites | Nucleotide and Its metabolites | 1 | - |
| 2'-O-methylcytidine | Nucleotide and Its metabolites | Nucleotide and Its metabolites | 2 | - |
| 2'-O-methyluridine | Nucleotide and Its metabolites | Nucleotide and Its metabolites | 2 | HMDB0240328 |
| 2,3-Dihydroxypropyl 2-[(octadec-9-enoyl)amino]ethyl hydrogen phosphate | GP | LPG | 2 | - |
| 2,3-dinor Fluprostenol | Hormones and hormone related compounds | Hormones and hormone related compounds | 2 | - |
| 2,3-dinor Prostaglandin E1 | Hormones and hormone related compounds | Hormones and hormone related compounds | 2 | - |
| 2,4-Di-tert-butylphenol | Benzene and substituted derivatives | Phenolics | 2 | HMDB0013816 |
| 2,4-Dihydroxypteridine | Heterocyclic compounds | Pteridines and derivatives | 2 | - |
| 2,4-Dinitrophenol | Benzene and substituted derivatives | Phenolics | 2 | HMDB0245462 |
| 2,4-Quinolinediol | Heterocyclic compounds | Heterocyclic compounds | 2 | - |
| 2,4-diacetamino-2,4,6-triphenoxy-D-mannopyranose | Carbohydrates and Its metabolites | Sugar derivatives | 2 | - |
| 2,5-Furandicarboxylicacid | Organic acid and Its derivatives | Organic acid and Its derivatives | 2 | HMDB0004812 |
| 2,6-Di-tert-butyl-4-(hydroxymethyl)phenol | Benzene and substituted derivatives | Benzene and substituted derivatives | 2 | HMDB0032048 |
| 2,6-Diamino-5-hydroxyhexanoic acid | Amino acid and Its metabolites | Amino acid derivatives | 1 | - |
| 2,6-Dihydroxyacetophenone | Benzene and substituted derivatives | Benzene and substituted derivatives | 1 | HMDB0029660 |
| 2-(4-hydroxyphenyl) propionate | Benzene and substituted derivatives | Phenolic acids | 2 | HMDB0041683 |
| 2-(Acetylamino)-2-deoxy-A-D-glucopyranose | Alcohol and amines | Amines | 2 | - |
| 2-(Dimethylamino)Guanosine | Nucleotide and Its metabolites | Nucleotide and Its metabolites | 1 | HMDB0004824 |
| 2-Amino-2-methyl-1,3-propanediol | Alcohol and amines | Alcohols | 2 | - |
| 2-Amino-3-phosphonopropionic acid | Organic acid and Its derivatives | Phosphoric acids | 2 | HMDB0000370 |
| 2-Amino-4-methylphenol | Benzene and substituted derivatives | Benzene and substituted derivatives | 2 | - |
| 2-Aminohexadecanoic acid | Organic acid and Its derivatives | Organic acid and Its derivatives | 2 | - |
| 2-Aminomethylpyrimidine | Nucleotide and Its metabolites | Nucleotide and Its metabolites | 1 | - |
| 2-Butyl-3-(4-hydroxybenzoyl)benzofuran | Benzene and substituted derivatives | Benzene and substituted derivatives | 2 | - |
| 2-Chloro-3-Deazaadenosine | Nucleotide and Its metabolites | Nucleotide and Its metabolites | 2 | - |
| 2-Deoxyribose 1-Phosphate | Carbohydrates and Its metabolites | Phosphate sugars | 2 | HMDB0001351 |
| 2-Dodecyl-3-methylbutanedioic acid | Organic acid and Its derivatives | Organic acid and Its derivatives | 2 | - |
| 2-Hexadecanoylthio-1-ethylphosphorylcholine | FA | Others | 2 | - |
| 2-Hexyldecanoic acid | FA | FFA | 2 | - |
| 2-Hydroxy-2-Methyl Butyric acid | Organic acid and Its derivatives | Organic acid and Its derivatives | 1 | HMDB0001987 |
| 2-Hydroxy-2-methylbutanenitrile | Others | Others | 2 | HMDB0060309 |
| 2-Hydroxy-3-Methyl Butanoic Acid | Organic acid and Its derivatives | Organic acid and Its derivatives | 1 | - |
| 2-Hydroxyadenosine | Nucleotide and Its metabolites | Nucleotide and Its metabolites | 1 | - |
| 2-Hydroxybutanoic Acid | Organic acid and Its derivatives | Organic acid and Its derivatives | 1 | HMDB0000008 |
| 2-Hydroxycaprylic acid | Organic acid and Its derivatives | Organic acid and Its derivatives | 1 | HMDB0000711 |
| 2-Hydroxycinnamic acid | Benzene and substituted derivatives | Phenolic acids | 2 | HMDB0002641 |
| 2-Hydroxyethanesulfonate | Organic acid and Its derivatives | Sulfonic acids | 1 | HMDB0003903 |
| 2-Hydroxyethanesulfonic acid;6-methyl-7-nitro-5-(piperidin-1-ylmethyl)-1,4-dihydroquinoxaline-2,3-dione | Heterocyclic compounds | Heterocyclic compounds | 2 | - |
| 2-Hydroxyhexanoic acid | Organic acid and Its derivatives | Organic acid and Its derivatives | 2 | HMDB0001624 |
| 2-Hydroxyhippuric acid | Benzene and substituted derivatives | Phenolics | 1 | HMDB0000840 |
| 2-Hydroxyibuprofen | Heterocyclic compounds | Heterocyclic compounds | 2 | HMDB0060920 |
| 2-Hydroxyimipramine | Heterocyclic compounds | Heterocyclic compounds | 2 | HMDB0060952 |
| 2-Hydroxyisocaproic Acid | Organic acid and Its derivatives | Organic acid and Its derivatives | 2 | HMDB0000665 |
| 2-Hydroxyquinoline | Aldehyde,Ketones,Esters | Ketones | 2 | - |
| 2-Indanone oxime | Benzene and substituted derivatives | Benzene and substituted derivatives | 2 | - |
| 2-Mercaptobenzothiazole | Heterocyclic compounds | Heterocyclic compounds | 1 | - |
| 2-Methoxy-5-nitrophenol | Benzene and substituted derivatives | Benzene and substituted derivatives | 2 | - |
| 2-Methoxyacetic acid | Organic acid and Its derivatives | Organic acid and Its derivatives | 2 | HMDB0041929 |
| 2-Methyl-1-Pyrroline | Heterocyclic compounds | Heterocyclic compounds | 1 | - |
| 2-Methyl-5-hydroxytryptamine | Tryptamines,Cholines,Pigments | Tryptamines | 2 | HMDB0245222 |
| 2-Methylglutaric Acid | Organic acid and Its derivatives | Organic acid and Its derivatives | 1 | HMDB0000422 |
| 2-Methylguanosine | Nucleotide and Its metabolites | Nucleotide and Its metabolites | 2 | HMDB0005862 |
| 2-Methyllactic acid | Organic acid and Its derivatives | Organic acid and Its derivatives | 2 | - |
| 2-Methylsuccinic Acid | Organic acid and Its derivatives | Organic acid and Its derivatives | 2 | HMDB0001844 |
| 2-Naphthalenesulfonic acid | Benzene and substituted derivatives | Benzene and substituted derivatives | 2 | - |
| 2-Octanamidoacetic acid | Amino acid and Its metabolites | Amino acids | 1 | HMDB0000832 |
| 2-Pentyl-3-phenyl-2-propenal | Aldehyde,Ketones,Esters | Aldehydes | 2 | HMDB0031313 |
| 2-Phenoxyethanol | Alcohol and amines | Alcohols | 2 | HMDB0041607 |
| 2-Phenylacetamide | Benzene and substituted derivatives | Benzene and substituted derivatives | 1 | HMDB0010715 |
| 2-Phenylglycine | Amino acid and Its metabolites | Amino acid derivatives | 1 | HMDB0002210 |
| 2-Phenylpropylamine | Benzene and substituted derivatives | Benzene and substituted derivatives | 1 | - |
| 2-Picoline | Heterocyclic compounds | Pteridines and derivatives | 2 | HMDB0061888 |
| 2-Propyl-2-pentenoic acid | Organic acid and Its derivatives | Organic acid and Its derivatives | 1 | HMDB0013902 |
| 2-Pyrrolidineacetic acid | Amino acid and Its metabolites | Amino acid derivatives | 2 | HMDB0029444 |
| 2-Pyrrolidinone | Heterocyclic compounds | Heterocyclic compounds | 1 | HMDB0002039 |
| 2-Thio-PAF | FA | Others | 2 | - |
| 2-[(1r,3s)-3-Hydroxycyclohexyl]-5-(2-methyloctan-2-yl)phenol | Benzene and substituted derivatives | Benzene and substituted derivatives | 2 | - |
| 2-[4-[3-[3,4-Dihydroxy-4-(hydroxymethyl)oxolan-2-yl]oxy-4,5-dihydroxy-6-(hydroxymethyl)oxan-2-yl]oxyphenyl]-7-hydroxy-2,3-dihydrochromen-4-one | Heterocyclic compounds | Heterocyclic compounds | 2 | HMDB0037491 |
| 2-amino-4-oxovaleric acid | Organic acid and Its derivatives | Organic acid and Its derivatives | 2 | - |
| 2-amino-6-methylmercaptopurine | Nucleotide and Its metabolites | Nucleotide and Its metabolites | 2 | - |
| 2-dehydro-D-gluconic acid | Carbohydrates and Its metabolites | Carbohydrates and Its metabolites | 1 | - |
| 2-ethyl-2-hydroxybutyric acid | Organic acid and Its derivatives | Organic acid and Its derivatives | 2 | HMDB0001975 |
| 20,26-dihydroxyecdysone | Hormones and hormone related compounds | Hormones and hormone related compounds | 2 | - |
| 20-Hydroxy Prostaglandin F2α | FA | Oxidized lipids | 2 | - |
| 20-ethyl Prostaglandin F2alpha | Hormones and hormone related compounds | Hormones and hormone related compounds | 2 | - |
| 3'-Adenylic acid | Nucleotide and Its metabolites | Nucleotide and Its metabolites | 2 | HMDB0003540 |
| 3'-N'-Acetylfusarochromanone | Aldehyde,Ketones,Esters | Ketones | 2 | HMDB0037499 |
| 3,3',5-Triiodo-L-Thyronine | Hormones and hormone related compounds | Hormones and hormone related compounds | 2 | HMDB0000265 |
| 3,4'-Dihydroxyflavonoid | Benzene and substituted derivatives | Benzene and substituted derivatives | 2 | - |
| 3,4-Dichloroaniline | Benzene and substituted derivatives | Benzene and substituted derivatives | 2 | - |
| 3,4-Dihydro-8-hydroxy-3-(3-hydroxy-4-methoxyphenyl) 1H-2-benzopyran-1-one | Benzene and substituted derivatives | Phenolics | 2 | HMDB0030807 |
| 3,4-Dimethoxycinnamic acid | Benzene and substituted derivatives | Benzene and substituted derivatives | 2 | HMDB0034315 |
| 3,4-Dimethylbenzoic acid | Benzene and substituted derivatives | Benzene and substituted derivatives | 2 | HMDB0002237 |
| 3,5-Dinitrocatechol | Benzene and substituted derivatives | Phenolics | 2 | HMDB0246069 |
| 3,6,9,12,15,18,21-Heptaoxatricosane-21,23-diol | Benzene and substituted derivatives | Benzene and substituted derivatives | 1 | - |
| 3-(2-Hydroxyphenyl)propanoic acid | Benzene and substituted derivatives | Phenolic acids | 1 | HMDB0033752 |
| 3-(3-Hydroxyphenyl)-3-hydroxypropanoic acid | Organic acid and Its derivatives | Organic acid and Its derivatives | 2 | HMDB0002643 |
| 3-(3-Hydroxyphenyl)Propionate Acid | Organic acid and Its derivatives | Organic acid and Its derivatives | 1 | HMDB0000375 |
| 3-(3-methylbut-2-en-1-yl)-3H-purin-6-amine | Heterocyclic compounds | Heterocyclic compounds | 2 | - |
| 3-(imidazol-4-yl)propionic acid | Heterocyclic compounds | Heterocyclic compounds | 1 | HMDB0002271 |
| 3-(pyrazol-1-yl)-L-alanine | Amino acid and Its metabolites | Amino acid derivatives | 2 | - |
| 3-Amino-2-piperidinone | Heterocyclic compounds | Heterocyclic compounds | 2 | HMDB0000323 |
| 3-Amino-4-Hydroxybenzoic Acid | Benzene and substituted derivatives | Phenolic acids | 2 | - |
| 3-Amino-5-hydroxybenzoic acid | Organic acid and Its derivatives | Organic acid and Its derivatives | 2 | - |
| 3-Amino-6-chloropyridazine | Heterocyclic compounds | Heterocyclic compounds | 2 | HMDB0062412 |
| 3-Aminoquinoline | Benzene and substituted derivatives | Benzene and substituted derivatives | 2 | HMDB0036827 |
| 3-Carboxypropyltrimethylammonium | Organic acid and Its derivatives | Organic acid and Its derivatives | 1 | - |
| 3-Chloroaniline | Benzene and substituted derivatives | Benzene and substituted derivatives | 1 | - |
| 3-Deaza-2'-deoxyadenosine | Nucleotide and Its metabolites | Nucleotide and Its metabolites | 2 | - |
| 3-Epideoxycholic acid | Bile acids | Bile acids | 1 | HMDB0000438 |
| 3-Fluoroethcathinone | Aldehyde,Ketones,Esters | Ketones | 2 | - |
| 3-Guanidinopropionic acid | Organic acid and Its derivatives | Organic acid and Its derivatives | 1 | HMDB0013222 |
| 3-Hydroxy-2-methylbutanoic acid | Organic acid and Its derivatives | Organic acid and Its derivatives | 1 | HMDB0000354 |
| 3-Hydroxy-3-Methyl Butyric Acid | Organic acid and Its derivatives | Organic acid and Its derivatives | 1 | HMDB0000754 |
| 3-Hydroxy-L-phenylalanine | Amino acid and Its metabolites | Amino acid derivatives | 1 | - |
| 3-Hydroxyanthranilic Acid | Benzene and substituted derivatives | Phenolic acids | 2 | HMDB0001476 |
| 3-Hydroxybutanoic acid | Organic acid and Its derivatives | Organic acid and Its derivatives | 1 | HMDB0000011 |
| 3-Hydroxycinnamic acid | Organic acid and Its derivatives | Organic acid and Its derivatives | 2 | HMDB0001713 |
| 3-Hydroxyhippuric Acid | Amino acid and Its metabolites | Amino acid derivatives | 1 | HMDB0006116 |
| 3-Hydroxylidocaine | Others | Medicine | 2 | - |
| 3-Hydroxyphenylacetic acid | Organic acid and Its derivatives | Organic acid and Its derivatives | 1 | HMDB0000440 |
| 3-Hydroxyphenylurea | Benzene and substituted derivatives | Benzene and substituted derivatives | 2 | - |
| 3-Hydroxytetradecanoic acid | Organic acid and Its derivatives | Organic acid and Its derivatives | 2 | HMDB0061656 |
| 3-Indoleacetonitrile | Heterocyclic compounds | Indole and Its derivatives | 1 | HMDB0006524 |
| 3-Indolebutyric Acid | Heterocyclic compounds | Indole and Its derivatives | 1 | HMDB0002096 |
| 3-Indolepropionic Acid | Heterocyclic compounds | Indole and Its derivatives | 1 | HMDB0002302 |
| 3-Isobutyl-1-methylxanthine | Heterocyclic compounds | Heterocyclic compounds | 2 | HMDB0245912 |
| 3-Methoxyphenylacetic acid | Organic acid and Its derivatives | Organic acid and Its derivatives | 2 | HMDB0059969 |
| 3-Methoxytyramine | Benzene and substituted derivatives | Phenolics | 2 | HMDB0000022 |
| 3-Methylcrotonyl Glycine | Organic acid and Its derivatives | Organic acid and Its derivatives | 2 | HMDB0000459 |
| 3-Methyluridine | Nucleotide and Its metabolites | Nucleotide and Its metabolites | 2 | HMDB0004813 |
| 3-Methylxanthine | Nucleotide and Its metabolites | Nucleotide and Its metabolites | 1 | HMDB0001886 |
| 3-O-Methyldopa | Alcohol and amines | Polyamines | 2 | - |
| 3-O-alpha-mycarosylerythronolide B | Aldehyde,Ketones,Esters | Ketones | 2 | - |
| 3-Oxostearic acid | Organic acid and Its derivatives | Organic acid and Its derivatives | 2 | - |
| 3-Phosphoglycerate | Organic acid and Its derivatives | Organic acid and Its derivatives | 2 | HMDB0000807 |
| 3-aminobenzamide | Benzene and substituted derivatives | Benzene and substituted derivatives | 2 | - |
| 3-carboxy-4-methyl-5-propyl-2-furanpropionic acid | Benzene and substituted derivatives | Benzene and substituted derivatives | 2 | - |
| 3-p-Coumaroylquinic acid | Phenolic acids | Phenolic acids | 2 | HMDB0029681 |
| 3alpha-Hydroxy-5beta-pregnane-20-one sulfate | Hormones and hormone related compounds | Hormones and hormone related compounds | 2 | HMDB0240590 |
| 4'-Hydroxywarfarin | Heterocyclic compounds | Heterocyclic compounds | 2 | HMDB0140989 |
| 4,4'-Dihydroxybenzophenone | Benzene and substituted derivatives | Benzene and substituted derivatives | 2 | - |
| 4-(3-Azidopropyl)cyclohexane-1,3-dione | Aldehyde,Ketones,Esters | Ketones | 2 | - |
| 4-(3-Hydroxybutyl)phenol | Benzene and substituted derivatives | Phenolics | 2 | - |
| 4-(4-Hydroxyaniline)-6,7-Dimethoxyquinazoline | Benzene and substituted derivatives | Benzene and substituted derivatives | 1 | - |
| 4-(Butylamino)benzoic acid | Benzene and substituted derivatives | Benzene and substituted derivatives | 2 | HMDB0246301 |
| 4-(Hydroxyamino)quinoline 1-oxide | Benzene and substituted derivatives | Benzene and substituted derivatives | 2 | - |
| 4-(Prop-2-en-1-yl)decahydro-8H-1,5-methanopyrido[1,2-a][1,5]diazocin-8-one | Heterocyclic compounds | Heterocyclic compounds | 2 | - |
| 4-Acetylaminobenzoic acid | Organic acid and Its derivatives | Organic acid and Its derivatives | 1 | - |
| 4-Amino-3-hydroxybutyric acid | Others | Medicine | 1 | - |
| 4-Amino-3-phenylbutanoic acid | Benzene and substituted derivatives | Benzene and substituted derivatives | 2 | HMDB0246346 |
| 4-Amino-5-hydroxymethyl-2-methylpyrimidine | Heterocyclic compounds | Heterocyclic compounds | 2 | HMDB0247327 |
| 4-Aminomethylpyrimidine | Heterocyclic compounds | Heterocyclic compounds | 1 | - |
| 4-Chloro-2-[(furan-2-ylmethyl)ammonio]-5-sulfamoylbenzoate | Benzene and substituted derivatives | Benzene and substituted derivatives | 2 | HMDB0001933 |
| 4-Chloro-2-nitrobenzylalcohol | Heterocyclic compounds | Heterocyclic compounds | 2 | HMDB0249542 |
| 4-Chloro-5-sulfamoylanthranilic acid | Benzene and substituted derivatives | Benzene and substituted derivatives | 2 | HMDB0060761 |
| 4-Chloro-L-phenylalanine | Amino acid and Its metabolites | Amino acids | 1 | HMDB0244605 |
| 4-Fluoro-3-nitrobenzotrifluoride | Benzene and substituted derivatives | Benzene and substituted derivatives | 2 | - |
| 4-Guanidinobutyric Acid | Organic acid and Its derivatives | Organic acid and Its derivatives | 1 | HMDB0003464 |
| 4-Hydroxy-2-Oxoglutaric Acid | Organic acid and Its derivatives | Organic acid and Its derivatives | 2 | HMDB0002070 |
| 4-Hydroxy-3-methoxybenzaldehyde | Benzene and substituted derivatives | Phenolics | 1 | HMDB0012308 |
| 4-Hydroxy-4-(pyridin-2-yl)butan-2-one | Heterocyclic compounds | Pyridine and pyridine derivatives | 2 | - |
| 4-Hydroxy-L-phenylglycine | Amino acid and Its metabolites | Amino acid derivatives | 2 | - |
| 4-Hydroxybenzaldehyde | Benzene and substituted derivatives | Benzene and substituted derivatives | 1 | HMDB0011718 |
| 4-Hydroxybenzyl alcohol | Benzene and substituted derivatives | Benzene and substituted derivatives | 1 | HMDB0011724 |
| 4-Hydroxyhippurate | Amino acid and Its metabolites | Amino acid derivatives | 1 | HMDB0013678 |
| 4-Hydroxyquinoline | Benzene and substituted derivatives | Benzene and substituted derivatives | 1 | - |
| 4-Hydroxytryptamine | Tryptamines,Cholines,Pigments | Tryptamines | 1 | - |
| 4-Methoxysalicylic Acid | Benzene and substituted derivatives | Phenolic acids | 1 | - |
| 4-Methylhippuric Acid | Amino acid and Its metabolites | Amino acid derivatives | 1 | HMDB0013292 |
| 4-Methylumbelliferone sulfate | Others | Others | 2 | - |
| 4-Methylumbelliferyl glucuronide | Heterocyclic compounds | Heterocyclic compounds | 2 | HMDB0240464 |
| 4-Phenylbutyric acid | Organic acid and Its derivatives | Organic acid and Its derivatives | 2 | HMDB0000543 |
| 4-Pyridoxic Acid | Heterocyclic compounds | Pteridines and derivatives | 2 | HMDB0000017 |
| 4-acetoxyphenol | Benzene and substituted derivatives | Phenolics | 2 | - |
| 4-amino-MX | Benzene and substituted derivatives | Benzene and substituted derivatives | 2 | HMDB0061009 |
| 4-tert-Octylphenol | Benzene and substituted derivatives | Phenolics | 2 | HMDB0013825 |
| 4-tert-butylbenzoic acid | Benzene and substituted derivatives | Phenolic acids | 1 | - |
| 5'-Deoxy-5'-(Methylthio) Adenosine | Nucleotide and Its metabolites | Nucleotide and Its metabolites | 1 | HMDB0001173 |
| 5,6-Dihydro-5-Methyluracil | Nucleotide and Its metabolites | Nucleotide and Its metabolites | 2 | HMDB0000079 |
| 5,6-Dimethylbenzimidazole | Heterocyclic compounds | Heterocyclic compounds | 2 | HMDB0003701 |
| 5,7-Dihydroxy-2-phenyl-6-[3,4,5-trihydroxy-6-(hydroxymethyl)oxan-2-yl]-8-(3,4,5-trihydroxyoxan-2-yl)chromen-4-one | Aldehyde,Ketones,Esters | Ketones | 2 | HMDB0140256 |
| 5,8,11-Eicosatrienoic acid | FA | FFA | 1 | HMDB0010378 |
| 5-(7-Acetyloxy-4-methoxycarbonyl-9-methyl-11-oxo-10-oxatricyclo[6.3.2.01,7]tridec-3-en-9-yl)-2-methylpenta-2,4-dienoic acid | Organic acid and Its derivatives | Organic acid and Its derivatives | 2 | HMDB0242611 |
| 5-Acetylamino-6-amino-3-methyluracil | Nucleotide and Its metabolites | Nucleotide and Its metabolites | 2 | HMDB0004400 |
| 5-Amino-2-(p-toluidino)benzenesulphonic acid | Benzene and substituted derivatives | Benzene and substituted derivatives | 2 | - |
| 5-Aminoimidazole ribonucleotide | Nucleotide and Its metabolites | Nucleotide and Its metabolites | 1 | HMDB0001235 |
| 5-Aminovaleric Acid | Amino acid and Its metabolites | Amino acid derivatives | 2 | HMDB0003355 |
| 5-Carboxyvanillic Acid | Organic acid and Its derivatives | Organic acid and Its derivatives | 2 | - |
| 5-Fluorouridine | Nucleotide and Its metabolites | Nucleotide and Its metabolites | 2 | HMDB0060396 |
| 5-Hydroxy-2'-deoxyuridine | Nucleotide and Its metabolites | Nucleotide and Its metabolites | 2 | - |
| 5-Hydroxyhexanoic Acid | Organic acid and Its derivatives | Organic acid and Its derivatives | 2 | HMDB0000525 |
| 5-Hydroxyindolepyruvate | Heterocyclic compounds | Indole and Its derivatives | 2 | - |
| 5-Hydroxyisourate | Organic acid and Its derivatives | Organic acid and Its derivatives | 2 | HMDB0030097 |
| 5-Hydroxymethyluracil | Nucleotide and Its metabolites | Nucleotide and Its metabolites | 2 | HMDB0000469 |
| 5-Methoxysalicylic Acid | Benzene and substituted derivatives | Benzene and substituted derivatives | 2 | HMDB0001868 |
| 5-Methoxytryptamine | Alcohol and amines | Amines | 2 | HMDB0004095 |
| 5-Methylcytidine | Nucleotide and Its metabolites | Nucleotide and Its metabolites | 1 | HMDB0000982 |
| 5-Methylcytosine | Nucleotide and Its metabolites | Nucleotide and Its metabolites | 1 | HMDB0002894 |
| 5-Methyluridine | Nucleotide and Its metabolites | Nucleotide and Its metabolites | 2 | HMDB0000884 |
| 5-Nitrosalicylic acid | Benzene and substituted derivatives | Benzene and substituted derivatives | 2 | - |
| 5-Oxoproline | Amino acid and Its metabolites | Amino acid derivatives | 1 | HMDB0000805 |
| 5-Phenylvaleric Acid | Organic acid and Its derivatives | Organic acid and Its derivatives | 1 | HMDB0002043 |
| 5-nitrobenzimidazole | Heterocyclic compounds | Heterocyclic compounds | 2 | - |
| 6,6'-(1,2-phenylene)bis(1,3,5-triazine-2,4-diamine) | Benzene and substituted derivatives | Benzene and substituted derivatives | 2 | - |
| 6-Aminocaproic Acid | Amino acid and Its metabolites | Amino acid derivatives | 1 | HMDB0001901 |
| 6-Chloropurine | Heterocyclic compounds | Heterocyclic compounds | 2 | HMDB0012245 |
| 6-Deoxyfagomine | Alkaloids | Alkaloids | 1 | HMDB0036382 |
| 6-Diazo-5-oxo-L-norleucine | Amino acid and Its metabolites | Amino acid derivatives | 2 | - |
| 6-Methylcoumarin | Heterocyclic compounds | Heterocyclic compounds | 1 | HMDB0032394 |
| 6-Methylnicotinamide | Heterocyclic compounds | Pteridines and derivatives | 1 | HMDB0013704 |
| 6-Methylthiopurine 5'-monophosphate ribonucleotide | Nucleotide and Its metabolites | Nucleotide and Its metabolites | 2 | HMDB0060414 |
| 6-O-methylguanine | Nucleotide and Its metabolites | Nucleotide and Its metabolites | 1 | - |
| 6-aminonicotinamide | Nucleotide and Its metabolites | Nucleotide and Its metabolites | 1 | - |
| 6-trans-12-epi Leukotriene B4 | FA | Oxidized lipids | 1 | HMDB0005088 |
| 6β-hydroxytestosterone | Hormones and hormone related compounds | Hormones and hormone related compounds | 2 | HMDB0006259 |
| 7,8,4'-Trimethoxyisoflavone | Flavonoids | Flavonoid | 2 | - |
| 7-(alpha-D-glucosyl)-N(6)-isopentenyladenine | Nucleotide and Its metabolites | Nucleotide and Its metabolites | 1 | - |
| 7-Hydroxywarfarin | Heterocyclic compounds | Heterocyclic compounds | 2 | HMDB0140988 |
| 7-Keto-dehydroepiandrosterone | Aldehyde,Ketones,Esters | Ketones | 2 | - |
| 7-Ketocholesterol | Hormones and hormone related compounds | Hormones and hormone related compounds | 2 | HMDB0000501 |
| 7-Methylguanine | Nucleotide and Its metabolites | Nucleotide and Its metabolites | 2 | HMDB0000897 |
| 7-Methylguanosine | Nucleotide and Its metabolites | Nucleotide and Its metabolites | 1 | HMDB0001107 |
| 7-Methyluric Acid | Amino acid and Its metabolites | Amino acid derivatives | 2 | HMDB0011107 |
| 7-Methylxanthine | Nucleotide and Its metabolites | Nucleotide and Its metabolites | 1 | HMDB0001991 |
| 7-ketolithocholic acid | Bile acids | Bile acids | 1 | - |
| 7alpha,12beta-Dihydroxy-5alpha-cholan-24-oic acid | Tryptamines,Cholines,Pigments | Cholines | 1 | - |
| 7alpha-Hydroxy-3-oxo-4-cholestenoic acid | Bile acids | Bile acids | 2 | HMDB0012458 |
| 7alpha-Hydroxytestosterone | Hormones and hormone related compounds | Hormones and hormone related compounds | 2 | HMDB0003956 |
| 8-Aminooctanoic Acid | Organic acid and Its derivatives | Organic acid and Its derivatives | 2 | HMDB0247418 |
| 8-Azaguanine | Nucleotide and Its metabolites | Nucleotide and Its metabolites | 1 | - |
| 8-Desoxygartanin | Heterocyclic compounds | Heterocyclic compounds | 2 | HMDB0030701 |
| 8-Geranylumbelliferone | Benzene and substituted derivatives | Benzene and substituted derivatives | 2 | - |
| 8-Methylnonenoate | Organic acid and Its derivatives | Organic acid and Its derivatives | 2 | HMDB0012183 |
| 8Z,11Z,14Z,17Z-eicosatetraenoic acid | FA | Oxidized lipids | 1 | - |
| 9(S),12(S),13(S)-TriHOME | FA | Oxidized lipids | 1 | HMDB0004708 |
| 9(S)-HpOTrE | FA | Oxidized lipids | 1 | - |
| 9,10-DiHOME | FA | Oxidized lipids | 2 | HMDB0004704 |
| 9,10-Epoxy-18-hydroxyoctadecanoic acid | FA | Oxidized lipids | 2 | - |
| 9,10-Phenanthrenequinone | Benzene and substituted derivatives | Benzene and substituted derivatives | 2 | - |
| 9S,15S-dihydroxy-5Z,13E-prostadienoic acid | FA | Oxidized lipids | 2 | - |
| AA | FA | FFA | 1 | HMDB0001043 |
| AMC Arachidonoyl Amide | FA | Others | 2 | - |
| Abn-cbd | Benzene and substituted derivatives | Benzene and substituted derivatives | 2 | - |
| Abu-His-OH | Amino acid and Its metabolites | Amino acid derivatives | 2 | - |
| Ac-DEVD-CHO | Amino acid and Its metabolites | Small Peptide | 2 | - |
| Acetaminophen sulfate | Benzene and substituted derivatives | Benzene and substituted derivatives | 2 | - |
| Acetanilide | Benzene and substituted derivatives | Benzene and substituted derivatives | 2 | HMDB0001250 |
| Acetylcholine | Alcohol and amines | Polyamines | 1 | HMDB0000895 |
| Acetylvalerenolic acid | Terpenoids | Sesquiterpenoids | 2 | HMDB0035687 |
| Acetylvaline | Amino acid and Its metabolites | Amino acid derivatives | 2 | HMDB0011757 |
| Acipimox | Heterocyclic compounds | Heterocyclic compounds | 2 | - |
| Acrylamide | Alcohol and amines | Amines | 2 | HMDB0004296 |
| Adenine | Nucleotide and Its metabolites | Nucleotide and Its metabolites | 1 | HMDB0000034 |
| Adenosine | Nucleotide and Its metabolites | Nucleotide and Its metabolites | 1 | HMDB0000050 |
| Adipic Acid | Organic acid and Its derivatives | Organic acid and Its derivatives | 2 | HMDB0000448 |
| Adrenosterone | Hormones and hormone related compounds | Hormones and hormone related compounds | 2 | HMDB0006772 |
| Agmatine | Alcohol and amines | Polyamines | 1 | HMDB0001432 |
| Ala-Ala | Amino acid and Its metabolites | Small Peptide | 2 | HMDB0003459 |
| Ala-Ala-Ala-Arg-Phe | Amino acid and Its metabolites | Small Peptide | 2 | - |
| Ala-Arg-Pro-Lys-Leu | Amino acid and Its metabolites | Small Peptide | 2 | - |
| Ala-Asn-Val-Asp | Amino acid and Its metabolites | Small Peptide | 2 | - |
| Ala-Asp-Phe-Asp | Amino acid and Its metabolites | Small Peptide | 2 | - |
| Ala-Gln-Phe-Ile-Met | Amino acid and Its metabolites | Small Peptide | 2 | - |
| Ala-Glu-Ile-Lys | Amino acid and Its metabolites | Small Peptide | 2 | - |
| Ala-Lys | Amino acid and Its metabolites | Amino acid derivatives | 1 | HMDB0028692 |
| Ala-Met-Leu-Asn-Asp | Amino acid and Its metabolites | Small Peptide | 2 | - |
| Ala-Nap-OH | Amino acid and Its metabolites | Small Peptide | 2 | - |
| Ala-Pro-Ala | Amino acid and Its metabolites | Small Peptide | 2 | - |
| Ala-Pro-Arg-Lys-Lys | Amino acid and Its metabolites | Small Peptide | 2 | - |
| Ala-Ser | Amino acid and Its metabolites | Small Peptide | 2 | - |
| Ala-Ser-His | Amino acid and Its metabolites | Small Peptide | 2 | - |
| Ala-Thr-Ile-Lys | Amino acid and Its metabolites | Small Peptide | 2 | - |
| Ala-Thr-Tyr-Lys | Amino acid and Its metabolites | Small Peptide | 2 | - |
| Ala-Val-Asn-Asp | Amino acid and Its metabolites | Small Peptide | 2 | - |
| Aldehydo-D-altrose | Carbohydrates and Its metabolites | Sugars | 2 | - |
| Alisol A | Aldehyde,Ketones,Esters | Ketones | 2 | - |
| Allopurinol | Nucleotide and Its metabolites | Nucleotide and Its metabolites | 1 | HMDB0014581 |
| Aloe emodin | Benzene and substituted derivatives | Benzene and substituted derivatives | 2 | HMDB0030829 |
| Alpha-Solanin | Alkaloids | Alkaloids | 2 | HMDB0034202 |
| Altretamine | Alcohol and amines | Amines | 2 | HMDB0014631 |
| Aminomalonic Acid | Organic acid and Its derivatives | Organic acid and Its derivatives | 2 | HMDB0001147 |
| Amlodipine | Heterocyclic compounds | Heterocyclic compounds | 2 | HMDB0005018 |
| Ammelide | Heterocyclic compounds | Heterocyclic compounds | 2 | - |
| Ammeline | Heterocyclic compounds | Heterocyclic compounds | 2 | - |
| Anandamide 0-phosphate | Alcohol and amines | Amines | 2 | - |
| Anaprel | Alkaloids | Alkaloids | 2 | - |
| Androstenediol | Hormones and hormone related compounds | Hormones and hormone related compounds | 2 | HMDB0003818 |
| Androsterone Enanthate | Hormones and hormone related compounds | Hormones and hormone related compounds | 2 | - |
| Aniline | Alcohol and amines | Amines | 1 | HMDB0003012 |
| Antanapeptin A | Benzene and substituted derivatives | Benzene and substituted derivatives | 2 | - |
| Antineoplaston A10 | Amino acid and Its metabolites | Amino acid derivatives | 2 | - |
| Apocholic acid | Bile acids | Bile acids | 1 | - |
| Arachidonoyl Glycine-d8 | Benzene and substituted derivatives | Benzene and substituted derivatives | 2 | - |
| Arachidonoyl LPA | GP | LPA | 2 | - |
| Arachidonylcyclopropylamide | Benzene and substituted derivatives | Benzene and substituted derivatives | 2 | - |
| Arainosine | Nucleotide and Its metabolites | Nucleotide and Its metabolites | 1 | HMDB0247301 |
| Arbutin | Benzene and substituted derivatives | Benzene and substituted derivatives | 2 | HMDB0029943 |
| Arg-Arg-Phe | Amino acid and Its metabolites | Small Peptide | 2 | - |
| Arg-Asn-Gln-Glu | Amino acid and Its metabolites | Small Peptide | 2 | - |
| Arg-Asp | Amino acid and Its metabolites | Small Peptide | 2 | HMDB0028705 |
| Arg-Asp-Arg | Amino acid and Its metabolites | Small Peptide | 2 | - |
| Arg-Asp-Asn | Amino acid and Its metabolites | Small Peptide | 2 | - |
| Arg-Glu-Ser-Leu-Glu | Amino acid and Its metabolites | Small Peptide | 2 | - |
| Arg-Gly-Glu-Val-Leu | Amino acid and Its metabolites | Small Peptide | 2 | - |
| Arg-Ile-His | Amino acid and Its metabolites | Small Peptide | 2 | - |
| Arg-Ile-Thr-Glu-Ala | Amino acid and Its metabolites | Small Peptide | 2 | - |
| Arg-Leu-Ala | Amino acid and Its metabolites | Small Peptide | 2 | - |
| Arg-Leu-Asn-Arg | Amino acid and Its metabolites | Small Peptide | 2 | - |
| Arg-Leu-Leu-Asn-Asn | Amino acid and Its metabolites | Small Peptide | 2 | - |
| Arg-Lys | Amino acid and Its metabolites | Small Peptide | 2 | - |
| Arg-Lys-Asp-Lys-Glu | Amino acid and Its metabolites | Small Peptide | 2 | - |
| Arg-Met | Amino acid and Its metabolites | Small Peptide | 2 | HMDB0028715 |
| Arg-Pro | Amino acid and Its metabolites | Small Peptide | 1 | HMDB0028717 |
| Arg-Ser-Lys-Arg | Amino acid and Its metabolites | Small Peptide | 2 | - |
| Arg-Thr-Ala-Arg | Amino acid and Its metabolites | Small Peptide | 2 | - |
| Arg-Thr-Lys-Arg | Amino acid and Its metabolites | Small Peptide | 2 | - |
| Arg-Tyr-Ala-Arg | Amino acid and Its metabolites | Small Peptide | 2 | - |
| Arg-Tyr-Asn-Glu | Amino acid and Its metabolites | Small Peptide | 2 | - |
| Arg-Tyr-Leu-Lys | Amino acid and Its metabolites | Small Peptide | 2 | - |
| Arg-Val-Ile-Trp-Gly | Amino acid and Its metabolites | Small Peptide | 2 | - |
| Argininosuccinic acid | Organic acid and Its derivatives | Organic acid and Its derivatives | 2 | HMDB0000052 |
| Asn-Arg-Glu-Ser-Leu | Amino acid and Its metabolites | Small Peptide | 2 | - |
| Asn-Arg-Phe-Lys | Amino acid and Its metabolites | Small Peptide | 2 | - |
| Asn-Asn-Leu-Asn-Val | Amino acid and Its metabolites | Small Peptide | 2 | - |
| Asn-Asn-Phe-Ser-Lys | Amino acid and Its metabolites | Small Peptide | 2 | - |
| Asn-Glu-Ile-Lys | Amino acid and Its metabolites | Small Peptide | 2 | - |
| Asn-Hyp | Amino acid and Its metabolites | Small Peptide | 2 | - |
| Asn-Ile-Asp-Lys | Amino acid and Its metabolites | Small Peptide | 2 | - |
| Asn-Leu-Pro-Ala-Lys | Amino acid and Its metabolites | Small Peptide | 2 | - |
| Asn-Lys-Arg-Asp | Amino acid and Its metabolites | Small Peptide | 2 | - |
| Asn-Phe-Ala-Arg | Amino acid and Its metabolites | Small Peptide | 2 | - |
| Asn-Phe-Ser-Arg | Amino acid and Its metabolites | Small Peptide | 2 | - |
| Asn-Thr-Lys | Amino acid and Its metabolites | Small Peptide | 2 | - |
| Asn-Trp | Amino acid and Its metabolites | Small Peptide | 2 | - |
| Asn-Val-Asp-Glu-Val | Amino acid and Its metabolites | Small Peptide | 2 | - |
| Asn-Val-Phe-Lys | Amino acid and Its metabolites | Small Peptide | 2 | - |
| Asp-Arg-Gln-Arg | Amino acid and Its metabolites | Small Peptide | 2 | - |
| Asp-Arg-Val-Lys-Asp | Amino acid and Its metabolites | Small Peptide | 2 | - |
| Asp-Asn-Gly-Asn-Phe | Amino acid and Its metabolites | Small Peptide | 2 | - |
| Asp-Cys-Arg-Val-Ser | Amino acid and Its metabolites | Small Peptide | 2 | - |
| Asp-Gly-Lys | Amino acid and Its metabolites | Small Peptide | 2 | - |
| Asp-Ile | Amino acid and Its metabolites | Small Peptide | 1 | - |
| Asp-Leu | Amino acid and Its metabolites | Small Peptide | 1 | - |
| Asp-Leu-Ala-Glu | Amino acid and Its metabolites | Small Peptide | 2 | - |
| Asp-Lys-Arg-Glu-Lys | Amino acid and Its metabolites | Small Peptide | 2 | - |
| Asp-Met-Leu-Asp-Leu | Amino acid and Its metabolites | Small Peptide | 2 | - |
| Asp-Phe | Amino acid and Its metabolites | Small Peptide | 1 | HMDB0000706 |
| Asp-Phe4Cl-OH | Amino acid and Its metabolites | Small Peptide | 2 | - |
| Aspartylmethionine | Amino acid and Its metabolites | Small Peptide | 2 | HMDB0028759 |
| Astaxanthin | Others | Others | 2 | HMDB0002204 |
| Asterina-330 | Amino acid and Its metabolites | Amino acid derivatives | 2 | - |
| Azacitidine | Heterocyclic compounds | Heterocyclic compounds | 1 | HMDB0015063 |
| Azelaic Acid | Organic acid and Its derivatives | Organic acid and Its derivatives | 1 | HMDB0000784 |
| Azelaoyl PAF | FA | Others | 2 | - |
| Barbital | Heterocyclic compounds | Heterocyclic compounds | 1 | HMDB0062202 |
| Barbituric acid | Heterocyclic compounds | Heterocyclic compounds | 1 | HMDB0041833 |
| Bardoxolone | Organic acid and Its derivatives | Organic acid and Its derivatives | 2 | - |
| Bardoxolone methyl | Organic acid and Its derivatives | Organic acid and Its derivatives | 2 | - |
| Benzaldehyde | Benzene and substituted derivatives | Benzene and substituted derivatives | 1 | HMDB0006115 |
| Benzamidine | Benzene and substituted derivatives | Benzene and substituted derivatives | 2 | HMDB0248970 |
| Benzenesulfonamide, 2-(cyclohexylamino)-5-nitro-N-((pentylamino)carbonyl)- | Benzene and substituted derivatives | Benzene and substituted derivatives | 2 | - |
| Benzidine | Benzene and substituted derivatives | Benzene and substituted derivatives | 2 | HMDB0041835 |
| Benzimidazole | Benzene and substituted derivatives | Benzene and substituted derivatives | 2 | HMDB0248993 |
| Benzyl n-[(2s)-4-methyl-1-[[(2r)-4-methyl-1-[[(2s)-4-methyl-1-oxopentan-2-yl]amino]-1-oxopentan-2-yl]amino]-1-oxopentan-2-yl]carbamate | Organic acid and Its derivatives | Organic acid and Its derivatives | 2 | - |
| Bepridil | Benzene and substituted derivatives | Benzene and substituted derivatives | 2 | HMDB0015374 |
| Beraprost | Benzene and substituted derivatives | Benzene and substituted derivatives | 2 | - |
| Betaine | Others | Others | 1 | HMDB0000043 |
| Betamethasone acetate | Aldehyde,Ketones,Esters | Esters | 2 | - |
| Bezafibrate | Benzene and substituted derivatives | Benzene and substituted derivatives | 2 | HMDB0015465 |
| Biotinamide | Alcohol and amines | Polyamines | 2 | HMDB0001458 |
| Bis(1-inositol)-3,1'-phosphate 1-phosphate | Alcohol and amines | Alcohols | 1 | - |
| Bovinic acid | FA | FFA | 1 | HMDB0003797 |
| Brinzolamide | Alcohol and amines | Amines | 2 | HMDB0015325 |
| Bromhexine | Benzene and substituted derivatives | Benzene and substituted derivatives | 2 | - |
| Buprenorphine | Benzene and substituted derivatives | Benzene and substituted derivatives | 2 | HMDB0015057 |
| Bupropion | Benzene and substituted derivatives | Benzene and substituted derivatives | 2 | HMDB0001510 |
| Butanoic acid, 4-((1,2-dioxohexadecyl)amino)-, ethyl ester | Organic acid and Its derivatives | Organic acid and Its derivatives | 2 | - |
| Butenoyl-PAF | Others | Others | 1 | - |
| Butylamine | Alcohol and amines | Amines | 2 | HMDB0031321 |
| Butylate | Aldehyde,Ketones,Esters | Esters | 2 | - |
| BzATP | Nucleotide and Its metabolites | Nucleotide and Its metabolites | 2 | - |
| CAY10577 | Benzene and substituted derivatives | Benzene and substituted derivatives | 2 | - |
| CMPF | Organic acid and Its derivatives | Organic acid and Its derivatives | 2 | HMDB0061112 |
| CMPentylF | Organic acid and Its derivatives | Organic acid and Its derivatives | 1 | - |
| CP 47,497-C8-homolog C-8-hydroxy metabolite | Benzene and substituted derivatives | Benzene and substituted derivatives | 2 | - |
| CYCLOPIAZONIC ACID | Organic acid and Its derivatives | Sulfonic acids | 2 | - |
| Caffeic Acid | Organic acid and Its derivatives | Organic acid and Its derivatives | 2 | HMDB0001964 |
| Caffeine | Heterocyclic compounds | Heterocyclic compounds | 1 | HMDB0001847 |
| Caldine | Alcohol and amines | Amines | 2 | - |
| Candesartan cilexetil | Benzene and substituted derivatives | Benzene and substituted derivatives | 2 | - |
| Capecitabine | Nucleotide and Its metabolites | Nucleotide and Its metabolites | 2 | HMDB0015233 |
| Capivasertib | Alcohol and amines | Amines | 2 | - |
| Carbamoyl phosphate | Organic acid and Its derivatives | Phosphoric acids | 2 | HMDB0001096 |
| Carnitine C10:0 | FA | CAR | 1 | - |
| Carnitine C10:1 | FA | CAR | 1 | - |
| Carnitine C10:2 | FA | CAR | 1 | - |
| Carnitine C11:0 | FA | CAR | 2 | - |
| Carnitine C11:DC | FA | CAR | 1 | - |
| Carnitine C12-OH | FA | CAR | 1 | - |
| Carnitine C12:0 | FA | CAR | 1 | - |
| Carnitine C12:1 | FA | CAR | 1 | - |
| Carnitine C13:0 | FA | CAR | 1 | HMDB0241308 |
| Carnitine C14-OH | FA | CAR | 1 | - |
| Carnitine C14:0 | FA | CAR | 2 | - |
| Carnitine C14:1 | FA | CAR | 1 | - |
| Carnitine C14:1-OH | FA | CAR | 1 | - |
| Carnitine C14:2-OH | FA | CAR | 2 | - |
| Carnitine C14:3 | FA | CAR | 1 | - |
| Carnitine C15:1 | FA | CAR | 1 | - |
| Carnitine C16-OH | FA | CAR | 2 | HMDB0013336 |
| Carnitine C16:1 | FA | CAR | 1 | - |
| Carnitine C16:2 | FA | CAR | 1 | - |
| Carnitine C18:1-OH | FA | CAR | 1 | - |
| Carnitine C18:2 | FA | CAR | 1 | HMDB0006469 |
| Carnitine C18:3 | FA | CAR | 2 | - |
| Carnitine C20:5 | FA | CAR | 2 | - |
| Carnitine C22:2 | FA | CAR | 1 | - |
| Carnitine C2:0 | FA | CAR | 1 | HMDB0000201 |
| Carnitine C3:0 | FA | CAR | 1 | HMDB0000824 |
| Carnitine C4:0 | FA | CAR | 1 | HMDB0002013 |
| Carnitine C4:DC | FA | CAR | 2 | - |
| Carnitine C5-OH | FA | CAR | 2 | HMDB0062555 |
| Carnitine C5:0 | FA | CAR | 1 | - |
| Carnitine C5:1 | FA | CAR | 1 | HMDB0002366 |
| Carnitine C6:0 | FA | CAR | 1 | HMDB0000705 |
| Carnitine C6:1 | FA | CAR | 1 | HMDB0013161 |
| Carnitine C7-OH | FA | CAR | 1 | HMDB0241677 |
| Carnitine C7:1 | FA | CAR | 2 | - |
| Carnitine C7:1 Isomer1 | FA | CAR | 2 | - |
| Carnitine C8-OH | FA | CAR | 1 | - |
| Carnitine C8:0 | FA | CAR | 1 | - |
| Carnitine C8:1 | FA | CAR | 1 | - |
| Carnitine C9:0 | FA | CAR | 1 | - |
| Carnitine C9:1 | FA | CAR | 2 | - |
| Carnitine C9:1-OH | FA | CAR | 1 | HMDB0015334 |
| Carnitine C9:DC | FA | CAR | 2 | - |
| Carnitine isoC4:0 | FA | CAR | 1 | HMDB0000736 |
| Carnitine ph-C1 | FA | CAR | 2 | - |
| Carnitine-2-methyl-C4 | FA | CAR | 1 | HMDB0000378 |
| Carprofen | Others | Medicine | 2 | HMDB0014959 |
| Chaetochromin | Others | Medicine | 2 | - |
| Chaps | Bile acids | Bile acids | 2 | - |
| Chenodeoxycholic Acid | Bile acids | Bile acids | 1 | HMDB0000518 |
| Chlorbromuron | Benzene and substituted derivatives | Benzene and substituted derivatives | 2 | - |
| Cholesterol | Hormones and hormone related compounds | Hormones and hormone related compounds | 2 | HMDB0000067 |
| Cholesterol sulfate | Hormones and hormone related compounds | Hormones and hormone related compounds | 2 | HMDB0000653 |
| Cholic acid | Bile acids | Bile acids | 1 | - |
| Choline | Tryptamines,Cholines,Pigments | Cholines | 1 | HMDB0000097 |
| Chromocarb | Organic acid and Its derivatives | Organic acid and Its derivatives | 1 | HMDB0250209 |
| Cis-4-Hydroxy-L-Proline | Amino acid and Its metabolites | Amino acid derivatives | 1 | HMDB0060460 |
| Cis-Aconitic Acid | Organic acid and Its derivatives | Organic acid and Its derivatives | 2 | HMDB0000072 |
| Clomipramine hydrochloride | Others | Medicine | 1 | - |
| Clopidogrel | Organic acid and Its derivatives | Phosphoric acids | 2 | HMDB0005011 |
| Cocamidopropyl betaine | FA | Others | 2 | - |
| Coenzyme-II (β-NADP) | CoEnzyme and vitamins | CoEnzyme and vitamins | 2 | HMDB0000217 |
| Conessine | Heterocyclic compounds | Heterocyclic compounds | 2 | - |
| Confertifoline | Aldehyde,Ketones,Esters | Esters | 1 | - |
| Cork-oximate | Organic acid and Its derivatives | Organic acid and Its derivatives | 2 | - |
| Corticosterone | Hormones and hormone related compounds | Hormones and hormone related compounds | 1 | HMDB0001547 |
| Cortisol | Hormones and hormone related compounds | Hormones and hormone related compounds | 1 | HMDB0000063 |
| Cortisone | Hormones and hormone related compounds | Hormones and hormone related compounds | 2 | HMDB0002802 |
| Creatine | Organic acid and Its derivatives | Organic acid and Its derivatives | 1 | HMDB0000064 |
| Creatine phosphate | Nucleotide and Its metabolites | Nucleotide and Its metabolites | 2 | HMDB0001511 |
| Creatinine | Organic acid and Its derivatives | Organic acid and Its derivatives | 1 | HMDB0000562 |
| Cucurbitacin B | Aldehyde,Ketones,Esters | Esters | 2 | HMDB0034927 |
| Cumyluron | Benzene and substituted derivatives | Benzene and substituted derivatives | 2 | - |
| Curcumol | Alcohol and amines | Alcohols | 2 | HMDB0038122 |
| Cyanazine | Alcohol and amines | Alcohols | 2 | - |
| Cyasterone | Aldehyde,Ketones,Esters | Ketones | 2 | - |
| Cyclic Amp | Nucleotide and Its metabolites | Nucleotide and Its metabolites | 1 | HMDB0000058 |
| Cyclo(Ala-Pro) | Amino acid and Its metabolites | Small Peptide | 2 | - |
| Cyclo(Phe-Glu) | Amino acid and Its metabolites | Small Peptide | 2 | - |
| Cyclo(Phe-Pro) | Amino acid and Its metabolites | Small Peptide | 1 | - |
| Cyclo(Pro-Leu) | Amino acid and Its metabolites | Small Peptide | 1 | HMDB0034276 |
| Cyclo(Pro-Phe) | Amino acid and Its metabolites | Small Peptide | 2 | - |
| Cyclo(Pro-Val) | Amino acid and Its metabolites | Small Peptide | 2 | - |
| Cyclo(Tyr-Ala) | Amino acid and Its metabolites | Small Peptide | 2 | - |
| Cyclocreatine | Nucleotide and Its metabolites | Nucleotide and Its metabolites | 2 | - |
| Cycloleucine | Amino acid and Its metabolites | Amino acid derivatives | 1 | HMDB0062225 |
| Cyclopentylglycine | Amino acid and Its metabolites | Amino acid derivatives | 2 | - |
| Cyromazine | Heterocyclic compounds | Heterocyclic compounds | 1 | HMDB0029862 |
| Cys-Gly | Amino acid and Its metabolites | Small Peptide | 2 | HMDB0000078 |
| Cys-Pro | Amino acid and Its metabolites | Small Peptide | 2 | - |
| Cytidine 5'-diphosphate | Nucleotide and Its metabolites | Nucleotide and Its metabolites | 2 | - |
| Cytidine-5-Monophosphate | Nucleotide and Its metabolites | Nucleotide and Its metabolites | 2 | HMDB0000095 |
| Cytochalasin H | Hormones and hormone related compounds | Hormones and hormone related compounds | 1 | - |
| D-(+)-sucrose | Carbohydrates and Its metabolites | Sugars | 1 | HMDB0000258 |
| D-Alloisoleucine | Amino acid and Its metabolites | Amino acids | 1 | - |
| D-Allose | Carbohydrates and Its metabolites | Sugars | 2 | - |
| D-Arabinose | Carbohydrates and Its metabolites | Sugars | 2 | HMDB0029942 |
| D-Cysteine | Organic acid and Its derivatives | Organic acid and Its derivatives | 2 | HMDB0003417 |
| D-Erythronolactone | Aldehyde,Ketones,Esters | Esters | 1 | HMDB0000349 |
| D-Fructose | Carbohydrates and Its metabolites | Sugars | 1 | HMDB0062538 |
| D-Fructose 6-Phosphate-Disodium Salt | Carbohydrates and Its metabolites | Phosphate sugars | 2 | - |
| D-Galacturonic Acid | Carbohydrates and Its metabolites | Sugar acids | 2 | HMDB0002545 |
| D-Gluconic Acid | Carbohydrates and Its metabolites | Sugar acids | 2 | HMDB0000625 |
| D-Glucoronic Acid | Carbohydrates and Its metabolites | Sugar acids | 2 | HMDB0000127 |
| D-Glucosaminic acid | Carbohydrates and Its metabolites | Sugar acids | 2 | - |
| D-Glucose | Carbohydrates and Its metabolites | Sugars | 1 | HMDB0000122 |
| D-Glucose 6-Phosphate | Carbohydrates and Its metabolites | Phosphate sugars | 2 | HMDB0001401 |
| D-Glutamic acid | Amino acid and Its metabolites | Amino acids | 2 | HMDB0003339 |
| D-Glycero-D-gulo-heptose | Carbohydrates and Its metabolites | Sugars | 2 | - |
| D-Kynurenine | Amino acid and Its metabolites | Amino acid derivatives | 2 | HMDB0250769 |
| D-Malic acid | Organic acid and Its derivatives | Organic acid and Its derivatives | 2 | HMDB0031518 |
| D-Mannitol | Carbohydrates and Its metabolites | Sugar alcohols | 2 | HMDB0000765 |
| D-Mannose | Carbohydrates and Its metabolites | Sugars | 2 | HMDB0000169 |
| D-Mannose 6-phosphate | Carbohydrates and Its metabolites | Phosphate sugars | 2 | - |
| D-Myo-inositol-2,4,5-triphosphate | Organic acid and Its derivatives | Organic acid and Its derivatives | 2 | - |
| D-Ornithine | Amino acid and Its metabolites | Amino acids | 1 | - |
| D-Quinovose | Carbohydrates and Its metabolites | Carbohydrates and Its metabolites | 1 | - |
| D-Sedoheptuiose 7-Phosphate | Carbohydrates and Its metabolites | Phosphate sugars | 2 | HMDB0001068 |
| D-Sorbitol | Carbohydrates and Its metabolites | Sugar alcohols | 2 | HMDB0000247 |
| D-Tagatose | Carbohydrates and Its metabolites | Sugars | 1 | HMDB0003418 |
| D-Talose | Carbohydrates and Its metabolites | Sugars | 2 | - |
| D-Trehalose | Carbohydrates and Its metabolites | Sugars | 2 | HMDB0000975 |
| D-Turanose | Others | Others | 2 | - |
| D-Xylose | Carbohydrates and Its metabolites | Sugars | 2 | HMDB0000098 |
| D-phenylalanine | Amino acid and Its metabolites | Amino acids | 1 | - |
| DHA | FA | FFA | 1 | HMDB0002183 |
| DL-2-Methylglutamic acid | Amino acid and Its metabolites | Amino acids | 2 | - |
| DL-3,4-Dihydroxyphenyl glycol | Benzene and substituted derivatives | Benzene and substituted derivatives | 2 | HMDB0000318 |
| DL-Arginine | Amino acid and Its metabolites | Amino acids | 1 | HMDB0251511 |
| DL-Carnitine | FA | CAR | 1 | HMDB0000062 |
| DL-Leucine | Amino acid and Its metabolites | Amino acids | 1 | HMDB0062203 |
| DL-O-tyrosine | Amino acid and Its metabolites | Amino acid derivatives | 1 | HMDB0006050 |
| DL-phenylmercapto uric acid | Benzene and substituted derivatives | Benzene and substituted derivatives | 2 | - |
| Daidzein | Benzene and substituted derivatives | Benzene and substituted derivatives | 1 | HMDB0003312 |
| Daidzein 4'-O-glucuronide | Flavonoids | Isoflavones | 2 | HMDB0041717 |
| Debromohymenialdisine | Heterocyclic compounds | Heterocyclic compounds | 2 | - |
| Deferasirox | Heterocyclic compounds | Heterocyclic compounds | 2 | HMDB0015547 |
| Dehydroacetic acid | Others | Medicine | 1 | HMDB0250943 |
| Dehydroascorbic acid | Organic acid and Its derivatives | Organic acid and Its derivatives | 2 | HMDB0001264 |
| Deoxycholic acid | Bile acids | Bile acids | 1 | HMDB0000626 |
| Deoxysappanone B 7,3'-dimethyl ether | Aldehyde,Ketones,Esters | Ketones | 2 | - |
| Deoxyuridine triphosphate | Nucleotide and Its metabolites | Nucleotide and Its metabolites | 2 | HMDB0001191 |
| Dexamethasone | Others | Medicine | 2 | HMDB0015364 |
| Diacerein | Benzene and substituted derivatives | Benzene and substituted derivatives | 2 | - |
| Diacetyl-8-gingerdiol | Heterocyclic compounds | Heterocyclic compounds | 2 | - |
| Dibenz(b,f)(1,4)oxazepine-10(11H)-carboxylic acid, 8-chloro-, 2-(1-oxo-3-(4-pyridinyl)propyl)hydrazide, monohydrochloride | Benzene and substituted derivatives | Benzene and substituted derivatives | 2 | - |
| Dichlorprop | Benzene and substituted derivatives | Benzene and substituted derivatives | 2 | HMDB0251202 |
| Dicumarol | Heterocyclic compounds | Heterocyclic compounds | 2 | HMDB0014411 |
| Dicyclohexylamine | Alcohol and amines | Amines | 2 | HMDB0251214 |
| Diethyl-2-methyl-3-oxosuccinate | Aldehyde,Ketones,Esters | Aldehydes | 2 | HMDB0032306 |
| Diethyl-phosphate | Aldehyde,Ketones,Esters | Esters | 2 | HMDB0012209 |
| Diflunisal | Benzene and substituted derivatives | Benzene and substituted derivatives | 2 | HMDB0014999 |
| Dihydrocaffeic acid | Organic acid and Its derivatives | Organic acid and Its derivatives | 2 | HMDB0000423 |
| Dihydrodaidzein | Benzene and substituted derivatives | Benzene and substituted derivatives | 2 | HMDB0005760 |
| Dihydrogambogic acid | Organic acid and Its derivatives | Organic acid and Its derivatives | 2 | - |
| Dihydrorhodamine 123 | Heterocyclic compounds | Heterocyclic compounds | 2 | HMDB0251329 |
| Dimethylmalonic acid | Organic acid and Its derivatives | Organic acid and Its derivatives | 2 | HMDB0002001 |
| Dinaciclib | Heterocyclic compounds | Heterocyclic compounds | 2 | - |
| Diphenhydramine | Benzene and substituted derivatives | Benzene and substituted derivatives | 1 | HMDB0001927 |
| Dl-2-Aminooctanoic Acid | Organic acid and Its derivatives | Organic acid and Its derivatives | 2 | HMDB0000991 |
| Docodiendioicacid | Organic acid and Its derivatives | Organic acid and Its derivatives | 2 | - |
| Docosatrienoic acid | FA | FFA | 2 | HMDB0002823 |
| Dodecanedioic Acid | FA | FFA | 1 | HMDB0000623 |
| Dodecylphosphocholine | Organic acid and Its derivatives | Phosphoric acids | 2 | - |
| Doramapimod | Heterocyclic compounds | Heterocyclic compounds | 2 | HMDB0251587 |
| Doxifluridine | Nucleotide and Its metabolites | Nucleotide and Its metabolites | 2 | HMDB0060406 |
| Dronedarone | Aldehyde,Ketones,Esters | Ketones | 2 | HMDB0251621 |
| Dulcitol | Carbohydrates and Its metabolites | Sugar alcohols | 2 | HMDB0000107 |
| EDTA | Organic acid and Its derivatives | Organic acid and Its derivatives | 2 | - |
| Ectoine | Heterocyclic compounds | Heterocyclic compounds | 2 | - |
| Eicosa-8,11,14-trien-5-ynoic acid | FA | Oxidized lipids | 1 | - |
| Emetine | Benzene and substituted derivatives | Benzene and substituted derivatives | 2 | - |
| Endorphine-2-trifluoroacetate | Benzene and substituted derivatives | Benzene and substituted derivatives | 2 | HMDB0005774 |
| Epipodophyllotoxin, 4'-demethyl-, 9-(4,6-O-2-thenylidene-beta-D-glucopyranoside) | Benzene and substituted derivatives | Benzene and substituted derivatives | 2 | HMDB0014587 |
| Eplerenone | Aldehyde,Ketones,Esters | Ketones | 1 | HMDB0014838 |
| Ergocornine | Alkaloids | Alkaloids | 2 | - |
| Estradiol cypionate | Hormones and hormone related compounds | Hormones and hormone related compounds | 2 | - |
| Ethambutol | Alcohol and amines | Alcohols | 2 | HMDB0014474 |
| Ethionamide | Heterocyclic compounds | Pteridines and derivatives | 2 | HMDB0014747 |
| Ethyl 2-amino-4-methylthiazole-5-carboxylate | Aldehyde,Ketones,Esters | Esters | 2 | - |
| Ethyl 3-Indoleacetate | Heterocyclic compounds | Indole and Its derivatives | 2 | - |
| Ethyl hydrogen malonate | Aldehyde,Ketones,Esters | Esters | 2 | HMDB0000576 |
| Ethyl palmitoleate | FA | Others | 2 | - |
| Ethyl stearate | FA | Others | 2 | HMDB0034156 |
| Ethyl sulfate | Others | Others | 2 | HMDB0031233 |
| Ethylene thiourea | Aldehyde,Ketones,Esters | Ketones | 2 | - |
| Ethylmalonate | Organic acid and Its derivatives | Organic acid and Its derivatives | 2 | HMDB0000622 |
| Ethylsalicylate | Organic acid and Its derivatives | Organic acid and Its derivatives | 2 | HMDB0029817 |
| Euxanthone | Flavonoids | Xanthone | 1 | HMDB0030724 |
| FAA(18:1) | FA | FFA | 1 | HMDB0240219 |
| FAHFA(6:0/18:3) | FA | FFA | 2 | - |
| FAHFA(8:0/10:0) | FA | FFA | 2 | - |
| FFA(12:0) | FA | FFA | 1 | HMDB0000638 |
| FFA(14:0) | FA | FFA | 1 | HMDB0000806 |
| FFA(14:1) | FA | FFA | 1 | HMDB0002000 |
| FFA(15:1) | FA | FFA | 1 | - |
| FFA(16:1) | FA | FFA | 1 | HMDB0003229 |
| FFA(16:2) | FA | FFA | 1 | HMDB0000477 |
| FFA(17:1) | FA | FFA | 2 | - |
| FFA(18:0) | FA | FFA | 1 | HMDB0000827 |
| FFA(18:2) | FA | FFA | 1 | HMDB0000673 |
| FFA(18:3) | FA | FFA | 1 | HMDB0003073 |
| FFA(18:4) | FA | FFA | 1 | - |
| FFA(18:5) | FA | FFA | 1 | - |
| FFA(20:2) | FA | FFA | 1 | HMDB0005060 |
| FFA(20:4) | FA | FFA | 1 | - |
| FFA(22:1) | FA | FFA | 1 | HMDB0002068 |
| FFA(22:4) | FA | FFA | 1 | HMDB0002226 |
| FFA(22:5) | FA | FFA | 2 | - |
| FFA(22:6) | FA | FFA | 1 | - |
| FR 122047 hydrochloride | Others | Others | 2 | - |
| Flibanserin | Others | Medicine | 1 | HMDB0252304 |
| Flunixin | Benzene and substituted derivatives | Benzene and substituted derivatives | 2 | - |
| Fluometuron | Benzene and substituted derivatives | Benzene and substituted derivatives | 2 | - |
| Fluphenazine sulfoxide | Heterocyclic compounds | Heterocyclic compounds | 2 | HMDB0252402 |
| Foetidin | Heterocyclic compounds | Heterocyclic compounds | 2 | HMDB0033271 |
| Formetanate | Benzene and substituted derivatives | Benzene and substituted derivatives | 2 | HMDB0252444 |
| Fumagillol | Heterocyclic compounds | Heterocyclic compounds | 2 | - |
| Furaltadone | Aldehyde,Ketones,Esters | Ketones | 2 | - |
| Fusidic acid | Others | Medicine | 2 | HMDB0015570 |
| GW4869 dihydrochloride | Heterocyclic compounds | Heterocyclic compounds | 2 | - |
| Gabazine | Heterocyclic compounds | Heterocyclic compounds | 2 | HMDB0252568 |
| Gal1-3[Fuc1-4]GlcNAcSp | Carbohydrates and Its metabolites | Sugar derivatives | 2 | - |
| Gal1-4[Fuc1-3]GlcNAcSp | Carbohydrates and Its metabolites | Sugar derivatives | 2 | - |
| Gamma-Mercholic Acid | Bile acids | Bile acids | 1 | - |
| Ganoderic acid H | FA | FFA | 2 | HMDB0035987 |
| Garcinolic acid | Organic acid and Its derivatives | Organic acid and Its derivatives | 2 | - |
| Garcinone C | Aldehyde,Ketones,Esters | Ketones | 2 | HMDB0029511 |
| Geldanamycin | Heterocyclic compounds | Heterocyclic compounds | 2 | - |
| Geniposidic acid | Organic acid and Its derivatives | Organic acid and Its derivatives | 2 | HMDB0034942 |
| Ginkgoic acid | Organic acid and Its derivatives | Organic acid and Its derivatives | 2 | HMDB0033897 |
| Ginkgolide C | Heterocyclic compounds | Heterocyclic compounds | 2 | HMDB0036860 |
| Gln-Gln | Amino acid and Its metabolites | Small Peptide | 2 | - |
| Gln-Gln-Gln-Glu-Gln | Amino acid and Its metabolites | Small Peptide | 2 | - |
| Gln-Gly | Amino acid and Its metabolites | Small Peptide | 2 | HMDB0028797 |
| Gln-Leu-Val-Arg-Ala | Amino acid and Its metabolites | Small Peptide | 2 | - |
| Gln-Lys-Phe-Arg | Amino acid and Its metabolites | Small Peptide | 2 | - |
| Gln-Phe | Amino acid and Its metabolites | Small Peptide | 2 | HMDB0028804 |
| Gln-Phe-Leu-Glu | Amino acid and Its metabolites | Small Peptide | 2 | - |
| Glu-Ala-Gln | Amino acid and Its metabolites | Small Peptide | 2 | - |
| Glu-Asn-Ile-Ile-Asp | Amino acid and Its metabolites | Small Peptide | 2 | - |
| Glu-Gln-Lys-Arg | Amino acid and Its metabolites | Small Peptide | 2 | - |
| Glu-Gln-Lys-Asp-Arg | Amino acid and Its metabolites | Small Peptide | 2 | - |
| Glu-Gly | Amino acid and Its metabolites | Small Peptide | 1 | HMDB0028819 |
| Glu-Ile | Amino acid and Its metabolites | Small Peptide | 2 | HMDB0028822 |
| Glu-Ile-Asp-Thr-Lys | Amino acid and Its metabolites | Small Peptide | 2 | - |
| Glu-Leu | Amino acid and Its metabolites | Small Peptide | 1 | HMDB0028823 |
| Glu-Lys-Ala-Lys-Ser | Amino acid and Its metabolites | Small Peptide | 2 | - |
| Glu-Lys-His-Cys-Arg | Amino acid and Its metabolites | Small Peptide | 2 | - |
| Glu-Lys-Leu-Thr-His | Amino acid and Its metabolites | Small Peptide | 2 | - |
| Glu-Met | Amino acid and Its metabolites | Small Peptide | 2 | HMDB0028825 |
| Glu-Phe | Amino acid and Its metabolites | Small Peptide | 2 | HMDB0029156 |
| Glu-Phe-Ala | Amino acid and Its metabolites | Small Peptide | 2 | - |
| Glu-Ser-Gln-Arg-Gln | Amino acid and Its metabolites | Small Peptide | 2 | - |
| Glu-Ser-Leu-Glu-Lys | Amino acid and Its metabolites | Small Peptide | 2 | - |
| Glu-Thr | Amino acid and Its metabolites | Small Peptide | 2 | HMDB0028829 |
| Glu-Tyr | Amino acid and Its metabolites | Small Peptide | 1 | HMDB0028831 |
| Glu-Val | Amino acid and Its metabolites | Small Peptide | 2 | HMDB0028832 |
| Glutaminyl-arginine | Amino acid and Its metabolites | Small Peptide | 2 | HMDB0028791 |
| Glutamylcysteine | Amino acid and Its metabolites | Small Peptide | 2 | HMDB0028816 |
| Glutaric Acid | Organic acid and Its derivatives | Organic acid and Its derivatives | 2 | HMDB0000661 |
| Glutathione Oxidized | Amino acid and Its metabolites | Small Peptide | 2 | - |
| Gly-Ala-Asn-Val-Arg | Amino acid and Its metabolites | Small Peptide | 2 | - |
| Gly-Asn-Leu-Arg-Lys | Amino acid and Its metabolites | Small Peptide | 2 | - |
| Gly-Gln | Amino acid and Its metabolites | Small Peptide | 2 | - |
| Gly-Glu | Amino acid and Its metabolites | Small Peptide | 1 | - |
| Gly-Glu-Gly-Phe-Lys | Amino acid and Its metabolites | Small Peptide | 2 | - |
| Gly-Gly-Phe | Amino acid and Its metabolites | Small Peptide | 1 | - |
| Gly-His | Amino acid and Its metabolites | Small Peptide | 2 | HMDB0028843 |
| Gly-Leu-Arg-Asn-Gln | Amino acid and Its metabolites | Small Peptide | 2 | - |
| Gly-Leu-Arg-Val-Phe | Amino acid and Its metabolites | Small Peptide | 2 | - |
| Gly-Leu-Asp-Val-Trp | Amino acid and Its metabolites | Small Peptide | 2 | - |
| Gly-Leu-Ser-Pro-Lys | Amino acid and Its metabolites | Small Peptide | 2 | - |
| Gly-Lys | Amino acid and Its metabolites | Small Peptide | 1 | - |
| Gly-Phe | Amino acid and Its metabolites | Amino acid derivatives | 1 | HMDB0028848 |
| Gly-Phe-Asn-Thr-Phe | Amino acid and Its metabolites | Small Peptide | 2 | - |
| Gly-Phe-His | Amino acid and Its metabolites | Small Peptide | 2 | - |
| Gly-Phe-Phe | Amino acid and Its metabolites | Small Peptide | 2 | - |
| Gly-Pro | Amino acid and Its metabolites | Small Peptide | 2 | HMDB0000721 |
| Gly-Ser-His | Amino acid and Its metabolites | Small Peptide | 2 | - |
| Gly-Thr | Amino acid and Its metabolites | Small Peptide | 2 | - |
| Gly-Val | Amino acid and Its metabolites | Small Peptide | 2 | HMDB0028854 |
| Glycerol tricaprylate | GL | TG | 2 | HMDB0011187 |
| Glycerophospho-N-Arachidonoyl Ethanolamine | GP | LPE | 1 | - |
| Glycerophospho-N-Oleoyl Ethanolamine | GP | PE | 2 | - |
| Glycerophospho-N-Palmitoyl Ethanolamine | GP | PE | 2 | - |
| Glycodeoxycholic acid | Bile acids | Bile acids | 1 | HMDB0000631 |
| Glycohyodeoxycholic acid | Bile acids | Bile acids | 1 | - |
| Glycoursodeoxycholic Acid | Bile acids | Bile acids | 1 | HMDB0000708 |
| Guanidine | Alcohol and amines | Polyamines | 2 | HMDB0001842 |
| Guanidineacetic Acid | Organic acid and Its derivatives | Organic acid and Its derivatives | 2 | HMDB0000128 |
| Guanidinoethyl Sulfonate | Organic acid and Its derivatives | Sulfonic acids | 1 | HMDB0003584 |
| Guanosine | Nucleotide and Its metabolites | Nucleotide and Its metabolites | 1 | HMDB0000133 |
| HMB-Val-Ser-Leu-VE | Benzene and substituted derivatives | Benzene and substituted derivatives | 2 | - |
| Heptethylene-glycol | Alcohol and amines | Alcohols | 2 | HMDB0061835 |
| Heterodendrin | Others | Others | 2 | - |
| Hexaconazole | Benzene and substituted derivatives | Benzene and substituted derivatives | 2 | - |
| Hexadecanedioic acid | FA | FFA | 1 | HMDB0000672 |
| Hexaethylene-glycol | Alcohol and amines | Alcohols | 1 | HMDB0061822 |
| Hexamethylphosphoramide | Alcohol and amines | Amines | 2 | HMDB0253142 |
| Hexanoyl Glycine | Amino acid and Its metabolites | Amino acid derivatives | 1 | HMDB0000701 |
| His-Ala-Arg-Glu | Amino acid and Its metabolites | Small Peptide | 2 | - |
| His-Gln-Tyr-Arg | Amino acid and Its metabolites | Small Peptide | 2 | - |
| His-Glu-Gln-Lys | Amino acid and Its metabolites | Small Peptide | 2 | - |
| His-Glu-Phe-Glu | Amino acid and Its metabolites | Small Peptide | 2 | - |
| His-Glu-Tyr-Lys | Amino acid and Its metabolites | Small Peptide | 2 | - |
| His-Gly-Ser | Amino acid and Its metabolites | Small Peptide | 2 | - |
| His-Gly-Tyr-Val-Glu | Amino acid and Its metabolites | Small Peptide | 2 | - |
| His-Gly-Val-Asp-Lys | Amino acid and Its metabolites | Small Peptide | 2 | - |
| His-Leu-Tyr-Lys | Amino acid and Its metabolites | Small Peptide | 2 | - |
| His-Lys-Met | Amino acid and Its metabolites | Small Peptide | 2 | - |
| His-Phe-Tyr-Asp | Amino acid and Its metabolites | Small Peptide | 2 | - |
| His-Ser | Amino acid and Its metabolites | Small Peptide | 2 | HMDB0028894 |
| His-Ser-Lys-Lys | Amino acid and Its metabolites | Small Peptide | 2 | - |
| His-Thr-Gln-Glu | Amino acid and Its metabolites | Small Peptide | 2 | - |
| His-Trp | Amino acid and Its metabolites | polypeptide | 2 | HMDB0028896 |
| His-Trp-Asp-His-Leu | Amino acid and Its metabolites | Small Peptide | 2 | - |
| His-Tyr-Ser | Amino acid and Its metabolites | Small Peptide | 2 | - |
| Histidylcysteine | Amino acid and Its metabolites | Small Peptide | 2 | HMDB0028882 |
| Huperzine A | Alcohol and amines | Amines | 2 | - |
| Hydralazine | Heterocyclic compounds | Heterocyclic compounds | 2 | HMDB0015400 |
| Hydromorphone | Others | Medicine | 2 | HMDB0014472 |
| Hydroumbellic acid | Benzene and substituted derivatives | Phenolics | 2 | HMDB0126386 |
| Hydroxyatrazine | Heterocyclic compounds | Heterocyclic compounds | 2 | HMDB0062766 |
| Hydroxyphenyllactic acid | Organic acid and Its derivatives | Organic acid and Its derivatives | 2 | HMDB0000755 |
| Hydroxypiperazic acid | Heterocyclic compounds | Heterocyclic compounds | 1 | - |
| Hydroxyquinoline | Heterocyclic compounds | Heterocyclic compounds | 1 | - |
| Hyodeoxycholic acid | Bile acids | Bile acids | 1 | HMDB0000733 |
| Hyp-Thr | Amino acid and Its metabolites | Small Peptide | 2 | - |
| Hypotaurocyamine | Alcohol and amines | Polyamines | 2 | - |
| Hypoxanthine | Nucleotide and Its metabolites | Nucleotide and Its metabolites | 1 | HMDB0000157 |
| ISODUARTIN METHYL ETHER | Heterocyclic compounds | Heterocyclic compounds | 2 | - |
| Ibuprofen | Heterocyclic compounds | Heterocyclic compounds | 2 | HMDB0001925 |
| Icosa-5,14-dienoic acid | Organic acid and Its derivatives | Organic acid and Its derivatives | 2 | - |
| Ile-Ala | Amino acid and Its metabolites | Small Peptide | 2 | - |
| Ile-Arg | Amino acid and Its metabolites | Small Peptide | 2 | - |
| Ile-Arg-Thr-Asp | Amino acid and Its metabolites | Small Peptide | 2 | - |
| Ile-Asp | Amino acid and Its metabolites | Small Peptide | 1 | HMDB0028903 |
| Ile-Asp-Lys-Lys | Amino acid and Its metabolites | Small Peptide | 2 | - |
| Ile-Gln | Amino acid and Its metabolites | Small Peptide | 2 | - |
| Ile-Glu | Amino acid and Its metabolites | Small Peptide | 2 | - |
| Ile-Glu-His-Lys | Amino acid and Its metabolites | Small Peptide | 2 | - |
| Ile-Glu-Leu-Lys | Amino acid and Its metabolites | Small Peptide | 2 | - |
| Ile-Glu-Tyr-Cys-Lys | Amino acid and Its metabolites | Small Peptide | 2 | - |
| Ile-His | Amino acid and Its metabolites | Small Peptide | 2 | - |
| Ile-His-His-Gly-Val | Amino acid and Its metabolites | Small Peptide | 2 | - |
| Ile-Ile-Glu-Glu-Val | Amino acid and Its metabolites | Small Peptide | 2 | - |
| Ile-Leu | Amino acid and Its metabolites | Small Peptide | 2 | - |
| Ile-Leu-Leu-Gly-Ala | Amino acid and Its metabolites | Small Peptide | 2 | - |
| Ile-Lys | Amino acid and Its metabolites | Small Peptide | 2 | HMDB0028912 |
| Ile-Phe-Gln-Glu | Amino acid and Its metabolites | Small Peptide | 2 | - |
| Ile-Phe-Val-Lys | Amino acid and Its metabolites | Small Peptide | 2 | - |
| Ile-Ser | Amino acid and Its metabolites | Small Peptide | 2 | - |
| Ile-Ser-Val-Asp | Amino acid and Its metabolites | Small Peptide | 2 | - |
| Ile-Thr | Amino acid and Its metabolites | Small Peptide | 2 | HMDB0028917 |
| Ile-Thr-Val-Lys-Leu | Amino acid and Its metabolites | Small Peptide | 2 | - |
| Ile-Tyr-Arg-Glu | Amino acid and Its metabolites | Small Peptide | 2 | - |
| Ile-Tyr-Lys-Ile-Arg | Amino acid and Its metabolites | Small Peptide | 2 | - |
| Ile-Val | Amino acid and Its metabolites | Small Peptide | 2 | - |
| Ile-Val-Ile-Phe-Asn | Amino acid and Its metabolites | Small Peptide | 2 | - |
| Ile-Val-Leu-Glu | Amino acid and Its metabolites | Small Peptide | 2 | - |
| Imazamox | Organic acid and Its derivatives | Organic acid and Its derivatives | 2 | HMDB0253404 |
| Imidazole-4-methanol | Heterocyclic compounds | Heterocyclic compounds | 1 | - |
| Imidazoleacetic acid | Heterocyclic compounds | Indole and Its derivatives | 1 | HMDB0002024 |
| Indole | Heterocyclic compounds | Indole and Its derivatives | 1 | HMDB0000738 |
| Indole 3-carbinol | Heterocyclic compounds | Indole and Its derivatives | 1 | HMDB0005785 |
| Indole-3-Carboxaldehyde | Heterocyclic compounds | Indole and Its derivatives | 1 | HMDB0029737 |
| Indole-3-acetamide | Heterocyclic compounds | Indole and Its derivatives | 1 | HMDB0029739 |
| Indole-3-carboxylic acid | Heterocyclic compounds | Indole and Its derivatives | 2 | HMDB0003320 |
| Indole-3-glyoxal | Heterocyclic compounds | Indole and Its derivatives | 2 | - |
| Indole-4-carboxaldehyde | Heterocyclic compounds | Indole and Its derivatives | 1 | - |
| Indoleacetaldehyde | Heterocyclic compounds | Indole and Its derivatives | 1 | HMDB0001190 |
| Inosine | Nucleotide and Its metabolites | Nucleotide and Its metabolites | 1 | HMDB0000195 |
| Inositol 1,3,4-trisphosphate | Organic acid and Its derivatives | Phosphoric acids | 2 | HMDB0001143 |
| Inositol 1-phosphate | Alcohol and amines | Alcohols | 2 | HMDB00213 |
| Irinotecan | Heterocyclic compounds | Heterocyclic compounds | 2 | HMDB0014900 |
| Isoangustone A | Aldehyde,Ketones,Esters | Ketones | 2 | HMDB0038905 |
| Isobavachalcone | Aldehyde,Ketones,Esters | Ketones | 2 | - |
| Isochodeoxycholic acid | Bile acids | Bile acids | 1 | HMDB0000361 |
| Isocitric acid | Organic acid and Its derivatives | Organic acid and Its derivatives | 2 | HMDB0000193 |
| Isofenphos-methyl | Benzene and substituted derivatives | Benzene and substituted derivatives | 2 | - |
| Isonicotinic acid | Organic acid and Its derivatives | Organic acid and Its derivatives | 2 | HMDB0060665 |
| Isoprenaline | Hormones and hormone related compounds | Hormones and hormone related compounds | 2 | - |
| Isopropyl 3-(3,4-dihydroxyphenyl)-2-hydroxypropanoate | Benzene and substituted derivatives | Benzene and substituted derivatives | 2 | - |
| Isorhamnetin | Flavonoids | Flavonoid | 2 | HMDB0002655 |
| Ivacaftor | Benzene and substituted derivatives | Benzene and substituted derivatives | 2 | HMDB0015705 |
| KOdiA-PC | GP | PC | 2 | - |
| Keto-Deoxy-Nonulonic acid | Organic acid and Its derivatives | Organic acid and Its derivatives | 2 | - |
| Ketoleucine | Organic acid and Its derivatives | Organic acid and Its derivatives | 1 | HMDB0000695 |
| Khelloside | Heterocyclic compounds | Heterocyclic compounds | 2 | - |
| Kynurenic Acid | Amino acid and Its metabolites | Amino acid derivatives | 2 | HMDB0000715 |
| L-3-Phenyllactic acid | Organic acid and Its derivatives | Organic acid and Its derivatives | 2 | HMDB0000748 |
| L-Allothreonine | Amino acid and Its metabolites | Amino acid derivatives | 1 | HMDB0004041 |
| L-Arginine | Amino acid and Its metabolites | Amino acids | 2 | HMDB0000517 |
| L-Ascorbate | CoEnzyme and vitamins | CoEnzyme and vitamins | 2 | HMDB0000044 |
| L-Ascorbyl 6-palmitate | Aldehyde,Ketones,Esters | Esters | 2 | HMDB0039883 |
| L-Asparagine Anhydrous | Amino acid and Its metabolites | Amino acids | 2 | HMDB0000168 |
| L-Citrulline | Amino acid and Its metabolites | Amino acids | 1 | HMDB0000904 |
| L-Cysteine | Amino acid and Its metabolites | Amino acids | 1 | HMDB0000574 |
| L-Cystine | Amino acid and Its metabolites | Amino acids | 2 | HMDB0000192 |
| L-Dihydroorotic Acid | Organic acid and Its derivatives | Organic acid and Its derivatives | 1 | HMDB0003349 |
| L-Dopa | Amino acid and Its metabolites | Amino acid derivatives | 2 | HMDB0000181 |
| L-Fucose | Carbohydrates and Its metabolites | Sugars | 1 | HMDB0000174 |
| L-Glutamic Acid | Amino acid and Its metabolites | Amino acids | 1 | HMDB0000148 |
| L-Glutamine | Amino acid and Its metabolites | Amino acids | 1 | HMDB0000641 |
| L-Glycine | Amino acid and Its metabolites | Amino acids | 1 | HMDB0000123 |
| L-Gulonolactone | Carbohydrates and Its metabolites | Sugar acids | 1 | HMDB0003466 |
| L-Gulose | Carbohydrates and Its metabolites | Carbohydrates and Its metabolites | 1 | - |
| L-Histidine | Amino acid and Its metabolites | Amino acids | 1 | HMDB0000177 |
| L-Homoarginine | Amino acid and Its metabolites | Amino acids | 1 | HMDB0000670 |
| L-Homocitrulline | Amino acid and Its metabolites | Amino acids | 1 | HMDB0000679 |
| L-Homocysteic acid | Organic acid and Its derivatives | Organic acid and Its derivatives | 2 | HMDB0002205 |
| L-Homoserine | Organic acid and Its derivatives | Organic acid and Its derivatives | 1 | HMDB0000719 |
| L-Iditol | Alcohol and amines | Alcohols | 1 | HMDB0011632 |
| L-Isoleucine | Amino acid and Its metabolites | Amino acids | 1 | HMDB0000172 |
| L-Isserine | Amino acid and Its metabolites | Amino acid derivatives | 1 | - |
| L-Lactic Acid | Organic acid and Its derivatives | Organic acid and Its derivatives | 1 | HMDB0000190 |
| L-Lysine | Amino acid and Its metabolites | Amino acids | 1 | HMDB0000182 |
| L-Methionine | Amino acid and Its metabolites | Amino acids | 1 | HMDB0000696 |
| L-Norleucine | Amino acid and Its metabolites | Amino acids | 1 | HMDB0001645 |
| L-Norvaline | Amino acid and Its metabolites | Amino acids | 1 | - |
| L-Octanoylcarnitine | FA | CAR | 2 | HMDB0000791 |
| L-Ornithine | Amino acid and Its metabolites | Amino acids | 1 | HMDB0000214 |
| L-Phenylalanine | Amino acid and Its metabolites | Amino acids | 1 | HMDB0000159 |
| L-Proline | Amino acid and Its metabolites | Amino acids | 1 | HMDB0000162 |
| L-Rhamnonic acid | Carbohydrates and Its metabolites | Sugar acids | 2 | - |
| L-Serine | Amino acid and Its metabolites | Amino acids | 1 | HMDB0000187 |
| L-Theanine | Amino acid and Its metabolites | Amino acids | 1 | HMDB0034365 |
| L-Thyroxine | Hormones and hormone related compounds | Hormones and hormone related compounds | 1 | HMDB0000248 |
| L-Tryptophan | Amino acid and Its metabolites | Amino acids | 1 | HMDB0000929 |
| L-Tryptophanamide | Amino acid and Its metabolites | Amino acid derivatives | 2 | HMDB0013318 |
| L-Tyrosine ethyl ester | Aldehyde,Ketones,Esters | Esters | 2 | - |
| L-Valine | Amino acid and Its metabolites | Amino acids | 1 | HMDB0000883 |
| L-kynurenine | Amino acid and Its metabolites | Amino acid derivatives | 1 | HMDB0000684 |
| L-lyxose | Carbohydrates and Its metabolites | Carbohydrates and Its metabolites | 2 | - |
| L-threo-3-Methylaspartate | Amino acid and Its metabolites | Amino acid derivatives | 2 | - |
| LPA(0:0/16:0) | GP | LPA | 1 | HMDB0007849 |
| LPA(0:0/18:0) | GP | LPA | 1 | - |
| LPA(16:0/0:0) | GP | LPA | 1 | HMDB0007853 |
| LPA(18:0/0:0) | GP | LPA | 2 | - |
| LPA(18:1/0:0) | GP | LPA | 1 | HMDB0007855 |
| LPA(20:0/0:0) | GP | LPA | 2 | - |
| LPA(22:6) | GP | LPA | 2 | - |
| LPC(0:0/12:0) | GP | LPC | 1 | - |
| LPC(0:0/14:0) | GP | LPC | 1 | - |
| LPC(0:0/15:0) | GP | LPC | 1 | - |
| LPC(0:0/16:0) | GP | LPC | 1 | - |
| LPC(0:0/18:0) | GP | LPC | 1 | - |
| LPC(0:0/18:1) | GP | LPC | 1 | - |
| LPC(0:0/18:2) | GP | LPC | 1 | - |
| LPC(0:0/18:3) | GP | LPC | 1 | - |
| LPC(0:0/20:1) | GP | LPC | 1 | - |
| LPC(0:0/20:2) | GP | LPC | 1 | - |
| LPC(0:0/20:3) | GP | LPC | 1 | - |
| LPC(0:0/20:4) | GP | LPC | 1 | - |
| LPC(0:0/20:5) | GP | LPC | 1 | - |
| LPC(0:0/22:4) | GP | LPC | 1 | - |
| LPC(0:0/22:5) | GP | LPC | 1 | - |
| LPC(12:0/0:0) | GP | LPC | 1 | - |
| LPC(13:0/0:0) | GP | LPC | 1 | - |
| LPC(16:2/0:0) | GP | LPC | 1 | - |
| LPC(17:0/0:0) | GP | LPC | 1 | HMDB0012108 |
| LPC(17:2/0:0) | GP | LPC | 2 | - |
| LPC(18:0/0:0) | GP | LPC | 1 | HMDB0010384 |
| LPC(18:1/0:0) | GP | LPC | 1 | HMDB0010385 |
| LPC(18:2/0:0) | GP | LPC | 1 | HMDB0010386 |
| LPC(18:3/0:0) | GP | LPC | 1 | HMDB0010387 |
| LPC(20:3/0:0) | GP | LPC | 1 | - |
| LPC(22:5/0:0) | GP | LPC | 1 | - |
| LPC(22:6/0:0) | GP | LPC | 1 | - |
| LPC(24:6/0:0) | GP | LPC | 2 | - |
| LPC(24:6e) | GP | LPC | 2 | - |
| LPC(O-16:0) | GP | LPC-O | 1 | - |
| LPC(O-16:1) | GP | LPC-O | 1 | - |
| LPC(O-18:1) | GP | LPC-O | 1 | - |
| LPC(O-18:3) | GP | LPC-O | 2 | - |
| LPE(0:0/14:0) | GP | LPE | 2 | HMDB0011470 |
| LPE(0:0/15:0) | GP | LPE | 1 | - |
| LPE(0:0/16:0) | GP | LPE | 1 | - |
| LPE(0:0/16:1) | GP | LPE | 1 | - |
| LPE(0:0/18:0) | GP | LPE | 1 | - |
| LPE(0:0/18:1) | GP | LPE | 2 | HMDB0011476 |
| LPE(0:0/18:2) | GP | LPE | 1 | - |
| LPE(0:0/18:3) | GP | LPE | 1 | - |
| LPE(0:0/20:2) | GP | LPE | 1 | - |
| LPE(0:0/20:3) | GP | LPE | 2 | - |
| LPE(0:0/20:5) | GP | LPE | 1 | - |
| LPE(0:0/22:5) | GP | LPE | 1 | - |
| LPE(0:0/22:6) | GP | LPE | 1 | - |
| LPE(14:0/0:0) | GP | LPE | 1 | HMDB0011500 |
| LPE(16:0/0:0) | GP | LPE | 1 | - |
| LPE(16:1/0:0) | GP | LPE | 1 | HMDB0011474 |
| LPE(17:0/0:0) | GP | LPE | 2 | - |
| LPE(17:1/0:0) | GP | LPE | 1 | - |
| LPE(18:0/0:0) | GP | LPE | 1 | HMDB0011130 |
| LPE(18:1/0:0) | GP | LPE | 1 | HMDB0011506 |
| LPE(18:2/0:0) | GP | LPE | 1 | HMDB0011507 |
| LPE(18:3/0:0) | GP | LPE | 1 | - |
| LPE(20:1/0:0) | GP | LPE | 2 | HMDB0011512 |
| LPE(20:2/0:0) | GP | LPE | 1 | - |
| LPE(20:3/0:0) | GP | LPE | 1 | - |
| LPE(20:4/0:0) | GP | LPE | 1 | - |
| LPE(20:5/0:0) | GP | LPE | 1 | - |
| LPE(22:5/0:0) | GP | LPE | 1 | - |
| LPE(22:6/0:0) | GP | LPE | 1 | - |
| LPE(O-17:1) | GP | LPE | 2 | - |
| LPE(O-18:1/0:0) | GP | LPE | 2 | - |
| LPE(O-18:2) | GP | LPE | 2 | - |
| LPE(P-16:0) | GP | LPE-P | 2 | HMDB0011152 |
| LPE(P-17:0) | GP | LPE-P | 2 | - |
| LPE(P-18:0) | GP | LPE-P | 2 | HMDB0240598 |
| LPE(P-18:1) | GP | LPE-P | 2 | - |
| LPG(0:0/18:2) | GP | LPG | 2 | - |
| LPG(16:0) | GP | LPG | 2 | - |
| LPG(16:1) | GP | LPG | 2 | - |
| LPG(17:0) | GP | LPG | 2 | - |
| LPG(18:0) | GP | LPG | 2 | - |
| LPG(18:1) | GP | LPG | 1 | HMDB0240602 |
| LPG(19:0) | GP | LPG | 2 | - |
| LPG(22:5) | GP | LPG | 2 | - |
| LPI(18:1) | GP | LPI | 1 | - |
| LPS(18:0) | GP | LPS | 2 | HMDB0240606 |
| LPS(20:0) | GP | LPS | 2 | - |
| Labetalol | Benzene and substituted derivatives | Benzene and substituted derivatives | 2 | HMDB0014736 |
| Lactitol | Carbohydrates and Its metabolites | Sugars | 2 | HMDB0040937 |
| Lactose | Carbohydrates and Its metabolites | Sugars | 2 | HMDB0000186 |
| Lactulose | Carbohydrates and Its metabolites | Sugars | 2 | HMDB0000740 |
| Leu-Ala | Amino acid and Its metabolites | Small Peptide | 2 | HMDB0028922 |
| Leu-Ala-Asn-Phe-Lys | Amino acid and Its metabolites | Small Peptide | 2 | - |
| Leu-Ala-His-Tyr-Asn | Amino acid and Its metabolites | Small Peptide | 2 | - |
| Leu-Ala-Met-Glu-Arg | Amino acid and Its metabolites | Small Peptide | 2 | - |
| Leu-Ala-Pro-Leu-Glu | Amino acid and Its metabolites | Small Peptide | 2 | - |
| Leu-Arg | Amino acid and Its metabolites | Small Peptide | 2 | - |
| Leu-Arg-Asn-Arg | Amino acid and Its metabolites | Small Peptide | 2 | - |
| Leu-Arg-Asp-Lys | Amino acid and Its metabolites | Small Peptide | 2 | - |
| Leu-Arg-Pro-Thr-Leu | Amino acid and Its metabolites | Small Peptide | 2 | - |
| Leu-Asp | Amino acid and Its metabolites | Small Peptide | 1 | - |
| Leu-Asp-Gln-Gln-Val | Amino acid and Its metabolites | Small Peptide | 2 | - |
| Leu-Asp-His-Arg | Amino acid and Its metabolites | Small Peptide | 2 | - |
| Leu-Gln-Arg-Arg | Amino acid and Its metabolites | Small Peptide | 2 | - |
| Leu-Gln-Ile-Arg | Amino acid and Its metabolites | Small Peptide | 2 | - |
| Leu-Glu | Amino acid and Its metabolites | Small Peptide | 2 | - |
| Leu-Glu-Lys-Glu | Amino acid and Its metabolites | Small Peptide | 2 | - |
| Leu-Gly | Amino acid and Its metabolites | Small Peptide | 2 | - |
| Leu-Gly-Phe-Glu-Val | Amino acid and Its metabolites | Small Peptide | 2 | - |
| Leu-His-Gly-Phe-His | Amino acid and Its metabolites | Small Peptide | 2 | - |
| Leu-Ile | Amino acid and Its metabolites | Small Peptide | 2 | - |
| Leu-Leu | Amino acid and Its metabolites | Small Peptide | 2 | HMDB0028933 |
| Leu-Leu-Asp-Leu-Leu | Amino acid and Its metabolites | Small Peptide | 2 | - |
| Leu-Leu-Leu-Pro-Gly | Amino acid and Its metabolites | Small Peptide | 2 | - |
| Leu-Leu-Lys-Gln-Gly | Amino acid and Its metabolites | Small Peptide | 2 | - |
| Leu-Leu-Ser-Pro-Tyr | Amino acid and Its metabolites | Small Peptide | 2 | - |
| Leu-Met-Ala-His-Leu | Amino acid and Its metabolites | Small Peptide | 2 | - |
| Leu-Nap-OH | Amino acid and Its metabolites | Small Peptide | 2 | - |
| Leu-Phe-Lys-Lys | Amino acid and Its metabolites | Small Peptide | 2 | - |
| Leu-Ser-Ser-Asp-Ile | Amino acid and Its metabolites | Small Peptide | 2 | - |
| Leu-Thr | Amino acid and Its metabolites | Small Peptide | 2 | - |
| Leu-Trp-Asn-Gly-Asp | Amino acid and Its metabolites | Small Peptide | 2 | - |
| Leu-Tyr-Thr-Lys | Amino acid and Its metabolites | Small Peptide | 2 | - |
| Leu-Val | Amino acid and Its metabolites | Small Peptide | 1 | HMDB0028942 |
| Leu-Val-Leu-Gly-Phe | Amino acid and Its metabolites | Small Peptide | 2 | - |
| Leu-Val-Lys | Amino acid and Its metabolites | Small Peptide | 2 | - |
| Leu-Val-Phe-Ala-Ile | Amino acid and Its metabolites | Small Peptide | 2 | - |
| Leu-Val-Val-Val-Asp | Amino acid and Its metabolites | Small Peptide | 2 | - |
| Leukotriene C4 methyl ester | Aldehyde,Ketones,Esters | Esters | 2 | - |
| Leukotriene C4-d5 | FA | Oxidized lipids | 2 | - |
| Levofloxacin | Heterocyclic compounds | Heterocyclic compounds | 1 | HMDB0001929 |
| Licochalcone B | Benzene and substituted derivatives | Benzene and substituted derivatives | 2 | HMDB0037320 |
| Lidocaine | Benzene and substituted derivatives | Benzene and substituted derivatives | 2 | HMDB0014426 |
| Linarin | Flavonoids | Flavonoid | 2 | - |
| Longicamphenylone | Aldehyde,Ketones,Esters | Esters | 2 | - |
| Losartan | Benzene and substituted derivatives | Benzene and substituted derivatives | 2 | HMDB0014816 |
| Lucidin | Benzene and substituted derivatives | Benzene and substituted derivatives | 2 | - |
| Lupulone | Aldehyde,Ketones,Esters | Ketones | 2 | - |
| Lys-Ala | Amino acid and Its metabolites | Small Peptide | 2 | HMDB0028944 |
| Lys-Ala-Leu-Glu | Amino acid and Its metabolites | Small Peptide | 2 | - |
| Lys-Arg-Leu-Glu | Amino acid and Its metabolites | Small Peptide | 2 | - |
| Lys-Asn-Leu-Tyr-Thr | Amino acid and Its metabolites | Small Peptide | 2 | - |
| Lys-Asp-Trp-Ser-Phe | Amino acid and Its metabolites | Small Peptide | 2 | - |
| Lys-Glu-Glu | Amino acid and Its metabolites | Small Peptide | 2 | - |
| Lys-Gly | Amino acid and Its metabolites | Small Peptide | 2 | HMDB0028951 |
| Lys-His-Ile-Glu-Glu | Amino acid and Its metabolites | Small Peptide | 2 | - |
| Lys-His-Met | Amino acid and Its metabolites | Small Peptide | 2 | - |
| Lys-Ile | Amino acid and Its metabolites | Small Peptide | 2 | HMDB0028954 |
| Lys-Ile-Glu-Arg | Amino acid and Its metabolites | Small Peptide | 2 | - |
| Lys-Ile-His | Amino acid and Its metabolites | Small Peptide | 2 | - |
| Lys-Ile-Val-Lys | Amino acid and Its metabolites | Small Peptide | 2 | - |
| Lys-Leu | Amino acid and Its metabolites | Small Peptide | 2 | HMDB0028955 |
| Lys-Leu-Thr-Asp-Glu | Amino acid and Its metabolites | Small Peptide | 2 | - |
| Lys-Lys-Arg | Amino acid and Its metabolites | Small Peptide | 2 | - |
| Lys-Lys-Lys-Gln-Tyr | Amino acid and Its metabolites | Small Peptide | 2 | - |
| Lys-Met | Amino acid and Its metabolites | Small Peptide | 2 | - |
| Lys-Met-His | Amino acid and Its metabolites | Small Peptide | 2 | - |
| Lys-Met-Val-Ser-Arg | Amino acid and Its metabolites | Small Peptide | 2 | - |
| Lys-Phe-Leu-Glu | Amino acid and Its metabolites | Small Peptide | 2 | - |
| Lys-Phe-Met | Amino acid and Its metabolites | Small Peptide | 2 | - |
| Lys-Ser | Amino acid and Its metabolites | Small Peptide | 2 | - |
| Lys-Thr | Amino acid and Its metabolites | Small Peptide | 2 | HMDB0028961 |
| Lys-Tyr-Ile-Glu | Amino acid and Its metabolites | Small Peptide | 2 | - |
| Lys-Tyr-Thr-Arg | Amino acid and Its metabolites | Small Peptide | 2 | - |
| Lys-Tyr-Val-Met-Leu | Amino acid and Its metabolites | Small Peptide | 2 | - |
| Lys-Val | Amino acid and Its metabolites | Small Peptide | 2 | HMDB0028964 |
| Lythramine | Alcohol and amines | Polyamines | 2 | - |
| Lyxo-2-Hexulose | Carbohydrates and Its metabolites | Carbohydrates and Its metabolites | 1 | HMDB0000660 |
| M-toluene acetic acid | Organic acid and Its derivatives | Organic acid and Its derivatives | 2 | HMDB0002222 |
| MG(0:0/22:6/0:0) | GL | MG | 2 | - |
| MG(16:0/0:0/0:0) | GL | MG | 1 | HMDB0011564 |
| MG(22:6/0:0/0:0) | GL | MG | 2 | - |
| Maleic Acid | Organic acid and Its derivatives | Organic acid and Its derivatives | 1 | HMDB0000176 |
| Malonic acid | Organic acid and Its derivatives | Organic acid and Its derivatives | 1 | HMDB0000691 |
| Maltitol | Carbohydrates and Its metabolites | Sugar alcohols | 2 | HMDB0002928 |
| Maltose | Carbohydrates and Its metabolites | Sugars | 1 | HMDB0000163 |
| Mebeverine | Benzene and substituted derivatives | Benzene and substituted derivatives | 2 | HMDB0254388 |
| Meglumine | Alcohol and amines | Amines | 2 | HMDB0240291 |
| Melanostatin | Heterocyclic compounds | Heterocyclic compounds | 2 | HMDB0005764 |
| Melibiose | Carbohydrates and Its metabolites | Sugars | 2 | HMDB0000048 |
| Mepanipyrim | Benzene and substituted derivatives | Benzene and substituted derivatives | 2 | - |
| Mesaconic acid | Organic acid and Its derivatives | Organic acid and Its derivatives | 1 | HMDB0000749 |
| Met-Arg | Amino acid and Its metabolites | Small Peptide | 2 | - |
| Met-Arg-Val | Amino acid and Its metabolites | Small Peptide | 2 | - |
| Met-Asp | Amino acid and Its metabolites | Small Peptide | 2 | - |
| Met-Asp-Gly | Amino acid and Its metabolites | Small Peptide | 2 | - |
| Met-Glu | Amino acid and Its metabolites | Small Peptide | 2 | - |
| Met-Gly-Met | Amino acid and Its metabolites | Small Peptide | 2 | - |
| Met-His-Lys | Amino acid and Its metabolites | Small Peptide | 2 | - |
| Met-Leu-Arg-Ala-Ala | Amino acid and Its metabolites | Small Peptide | 2 | - |
| Met-Lys-Lys | Amino acid and Its metabolites | Small Peptide | 2 | - |
| Met-Phe-His | Amino acid and Its metabolites | Small Peptide | 2 | - |
| Met-Phe-Thr-Glu-Asp | Amino acid and Its metabolites | Small Peptide | 2 | - |
| Met-Trp-Glu | Amino acid and Its metabolites | Small Peptide | 2 | - |
| Met-Val | Amino acid and Its metabolites | Small Peptide | 2 | - |
| Methamidophos | Alcohol and amines | Amines | 2 | HMDB0031803 |
| Methanesulfonic acid | Organic acid and Its derivatives | Sulfonic acids | 1 | - |
| Methimazole | Heterocyclic compounds | Heterocyclic compounds | 2 | HMDB0014901 |
| Methionine Sulfoxide | Amino acid and Its metabolites | Amino acid derivatives | 2 | HMDB0002005 |
| Methyl 10-gingerol | Aldehyde,Ketones,Esters | Esters | 2 | - |
| Methyl 2,4-dihydroxy-3,6-dimethylbenzoate | Benzene and substituted derivatives | Benzene and substituted derivatives | 2 | - |
| Methyl 4-hydroxycinnamate | Aldehyde,Ketones,Esters | Esters | 1 | HMDB0131168 |
| Methyl Indole-3-Acetate | Heterocyclic compounds | Indole and Its derivatives | 1 | HMDB0029738 |
| Methyl caffeate | Benzene and substituted derivatives | Benzene and substituted derivatives | 2 | - |
| Methyl dioxindole-3-acetate | Heterocyclic compounds | Indole and Its derivatives | 1 | HMDB0038991 |
| Methyl hippurate | Aldehyde,Ketones,Esters | Esters | 2 | HMDB0000859 |
| Methyl reserpate | Alkaloids | Alkaloids | 2 | - |
| Methyl-L-alaninate | Amino acid and Its metabolites | Amino acid derivatives | 2 | - |
| Methylcysteine | Amino acid and Its metabolites | Amino acids | 2 | HMDB0002108 |
| Methyldiphenylphosphine oxide | Benzene and substituted derivatives | Benzene and substituted derivatives | 2 | - |
| Methyldopa | Amino acid and Its metabolites | Amino acid derivatives | 2 | HMDB0011754 |
| Methylergonovine | Alkaloids | Alkaloids | 2 | HMDB0014497 |
| Methylgingerol | Phenolic acids | Phenolic acids | 2 | HMDB0029852 |
| Methylguanidine | Alcohol and amines | Amines | 2 | - |
| Methylmalonic Acid | Organic acid and Its derivatives | Organic acid and Its derivatives | 1 | HMDB0000202 |
| Methylparaben | Benzene and substituted derivatives | Phenolic acids | 1 | HMDB0032572 |
| Methylprednisolone | Hormones and hormone related compounds | Hormones and hormone related compounds | 2 | HMDB0015094 |
| Miltefosine | Organic acid and Its derivatives | Phosphoric acids | 2 | HMDB0254735 |
| Mitomycin | Heterocyclic compounds | Heterocyclic compounds | 2 | HMDB0014450 |
| Molinate | Others | Others | 2 | - |
| Morphine N-oxide | Alkaloids | Isoquinoline alkaloids | 2 | HMDB0029382 |
| Multifidol | Benzene and substituted derivatives | Phenolics | 2 | - |
| Murideoxycholic acid | Bile acids | Bile acids | 2 | HMDB0000811 |
| Mycosporine-glycine | Amino acid and Its metabolites | Amino acid derivatives | 1 | - |
| Myoinositol | Carbohydrates and Its metabolites | Sugar alcohols | 1 | HMDB0000211 |
| N'-Formylkynurenine | Amino acid and Its metabolites | Amino acid derivatives | 1 | HMDB0001200 |
| N'-Hydroxy-4-methylbenzenecarboximidamide | Benzene and substituted derivatives | Benzene and substituted derivatives | 2 | - |
| N(Alpha)-Acetyl-Epsilon-(2-Propenal)Lysine | Amino acid and Its metabolites | Small Peptide | 2 | - |
| N,N-Bis(2-hydroxyethyl)dodecanamide | FA | Others | 1 | HMDB0032358 |
| N,N-Dimethylaniline | Benzene and substituted derivatives | Benzene and substituted derivatives | 2 | HMDB0001020 |
| N,N-Dimethylarginine | Amino acid and Its metabolites | Amino acid derivatives | 2 | HMDB0001539 |
| N,N′-dicyclohexylcarbodiimide | Alcohol and amines | Polyamines | 2 | - |
| N-(2,2,2-Trifluoroethyl)-N-{4-[2,2,2-trifluoro-1-hydroxy-1-(trifluoromethyl)ethyl]phenyl}benzenesulfonamide | Benzene and substituted derivatives | Benzene and substituted derivatives | 2 | HMDB0258663 |
| N-(3-Hydroxypropyl)phthalimide | Benzene and substituted derivatives | Benzene and substituted derivatives | 2 | - |
| N-(5-Adamant-1-yl methoxy)pentyl deoxynojirimycin | Heterocyclic compounds | Heterocyclic compounds | 2 | HMDB0248353 |
| N-1-Naphthylbenzamide | Benzene and substituted derivatives | Benzene and substituted derivatives | 2 | - |
| N-Acetyl-L-Histidine | Amino acid and Its metabolites | Amino acid derivatives | 1 | HMDB0032055 |
| N-Acetyl-L-Leucine | Amino acid and Its metabolites | Amino acid derivatives | 2 | HMDB0011756 |
| N-Acetyl-L-Tyrosine | Amino acid and Its metabolites | Amino acid derivatives | 1 | HMDB0000866 |
| N-Acetyl-L-alanine | Amino acid and Its metabolites | Amino acid derivatives | 1 | HMDB0000766 |
| N-Acetyl-L-methionine | Amino acid and Its metabolites | Amino acid derivatives | 2 | HMDB0011745 |
| N-Acetyl-L-phenylalanine | Amino acid and Its metabolites | Amino acid derivatives | 1 | HMDB0000512 |
| N-Acetylaminooctanoic acid | Organic acid and Its derivatives | Organic acid and Its derivatives | 2 | - |
| N-Acetylaspartate | Amino acid and Its metabolites | Amino acid derivatives | 2 | HMDB0000812 |
| N-Acetylcysteine | Amino acid and Its metabolites | Amino acid derivatives | 1 | HMDB0001890 |
| N-Acetylglucosamine 1-Phosphate | Carbohydrates and Its metabolites | Phosphate sugars | 2 | HMDB0001367 |
| N-Acetylglycine | Amino acid and Its metabolites | Amino acid derivatives | 2 | HMDB0000532 |
| N-Acetylhistamine | Alcohol and amines | Amines | 1 | HMDB0013253 |
| N-Acetylproline | Amino acid and Its metabolites | Amino acid derivatives | 2 | HMDB0094701 |
| N-Acetylthreonine | Amino acid and Its metabolites | Amino acid derivatives | 2 | HMDB0062557 |
| N-Alpha-Acetyl-L-Asparagine | Amino acid and Its metabolites | Amino acid derivatives | 1 | HMDB0006028 |
| N-Arachidonoyl dopamine | Benzene and substituted derivatives | Phenolics | 2 | - |
| N-Arachidonoyl-3-hydroxy-Aminobutyric Acid | Amino acid and Its metabolites | Amino acid derivatives | 2 | - |
| N-Benzyloxycarbonyl-L-leucine | Amino acid and Its metabolites | Amino acid derivatives | 2 | - |
| N-Butyrylglycine | Amino acid and Its metabolites | Amino acid derivatives | 2 | HMDB0000808 |
| N-Cyclopropyl-11-(2-hexyl-5-hydroxyphenoxy)undecanamide | Benzene and substituted derivatives | Phenolics | 2 | - |
| N-Ethylglycine | Amino acid and Its metabolites | Amino acid derivatives | 1 | HMDB0041945 |
| N-Formylglycine | Amino acid and Its metabolites | Amino acids | 1 | - |
| N-Isovaleroylglycine | Amino acid and Its metabolites | Amino acid derivatives | 1 | HMDB0000678 |
| N-MethyTrans-4-Hydroxy-Proline | Amino acid and Its metabolites | Amino acid derivatives | 2 | - |
| N-Methyl-L-Glutamate | Amino acid and Its metabolites | Amino acid derivatives | 2 | HMDB0062660 |
| N-Methyl-α-aminoisobutyric acid | Amino acid and Its metabolites | Amino acid derivatives | 1 | HMDB0002141 |
| N-Methylalanine | Amino acid and Its metabolites | Amino acid derivatives | 1 | HMDB0094692 |
| N-Methyltryptamine | Tryptamines,Cholines,Pigments | Tryptamines | 2 | HMDB0004370 |
| N-Myristoylglycine | Amino acid and Its metabolites | Amino acid derivatives | 2 | HMDB0013250 |
| N-Oleoyl Glycine | Alcohol and amines | Amines | 2 | HMDB0013631 |
| N-Palmitoylglycine | Amino acid and Its metabolites | Amino acid derivatives | 2 | HMDB0013034 |
| N-Propionylglycine | Amino acid and Its metabolites | Amino acids | 2 | HMDB0000783 |
| N-acetyl-D-Lactosamine | Carbohydrates and Its metabolites | Carbohydrates and Its metabolites | 2 | HMDB0001542 |
| N-acetyl-D-phenylalanine | Amino acid and Its metabolites | Amino acid derivatives | 2 | - |
| N-acetyl-beta-alanine | Amino acid and Its metabolites | Amino acid derivatives | 2 | HMDB0061880 |
| N-acetylornithine | Amino acid and Its metabolites | Amino acid derivatives | 1 | HMDB0003357 |
| N-acetylpyrrolidine | Heterocyclic compounds | Heterocyclic compounds | 2 | - |
| N-lactoyl-phenylalanine | Amino acid and Its metabolites | Amino acid derivatives | 1 | HMDB0062175 |
| N-methylundec-10-enamide | FA | Others | 2 | - |
| N-octanoylsphingosine | SL | Cer | 2 | - |
| N-octanoylsphingosine 1-phosphate | SL | Cer | 2 | - |
| N1,N8-diacetylspermidine | Alcohol and amines | Amines | 2 | HMDB0041947 |
| N1-Acetylspermidine | Alcohol and amines | Amines | 2 | - |
| N1-Acetylspermine | Organic acid and Its derivatives | Organic acid and Its derivatives | 2 | HMDB0001186 |
| N4-Acetylcytidine | Nucleotide and Its metabolites | Nucleotide and Its metabolites | 1 | HMDB0005923 |
| N6,N6,N6-Trimethyl-L-lysine | Amino acid and Its metabolites | Amino acid derivatives | 1 | - |
| N6-(2-Hydroxyethyl)adenosine | Nucleotide and Its metabolites | Nucleotide and Its metabolites | 1 | - |
| N6-Acetyl-L-Lysine | Amino acid and Its metabolites | Amino acid derivatives | 1 | HMDB0000206 |
| N6-methyladenosine | Nucleotide and Its metabolites | Nucleotide and Its metabolites | 1 | HMDB0004044 |
| NHS-Biotin | Heterocyclic compounds | Heterocyclic compounds | 2 | - |
| Nandrolone decanoate | Hormones and hormone related compounds | Hormones and hormone related compounds | 2 | HMDB0015694 |
| Nap-Asp-OH | Amino acid and Its metabolites | Small Peptide | 2 | - |
| Nap-Met-OH | Amino acid and Its metabolites | Small Peptide | 2 | - |
| Natamycin | Aldehyde,Ketones,Esters | Esters | 2 | HMDB0014964 |
| Nb-Palmitoyltryptamine | Tryptamines,Cholines,Pigments | Tryptamines | 2 | HMDB0040815 |
| Nbd-ceramide | Benzene and substituted derivatives | Benzene and substituted derivatives | 2 | - |
| Niclosamide | Others | Medicine | 2 | - |
| Nicotinamide | CoEnzyme and vitamins | CoEnzyme and vitamins | 1 | HMDB0001406 |
| Nicotinate mononucleotide | Nucleotide and Its metabolites | Nucleotide and Its metabolites | 2 | HMDB0001132 |
| Nicotinic Acid | CoEnzyme and vitamins | CoEnzyme and vitamins | 1 | HMDB0001488 |
| Nintedanib | Others | Medicine | 2 | HMDB0255615 |
| Nitazoxanide | Benzene and substituted derivatives | Benzene and substituted derivatives | 2 | HMDB0014649 |
| Nitrendipine | Heterocyclic compounds | Heterocyclic compounds | 2 | HMDB0015187 |
| Nitroxoline | Heterocyclic compounds | Heterocyclic compounds | 1 | HMDB0015491 |
| Nizatidine | Heterocyclic compounds | Heterocyclic compounds | 2 | HMDB0014723 |
| Nomifensine maleate | Benzene and substituted derivatives | Benzene and substituted derivatives | 2 | - |
| Nomilin | Terpenoids | Triterpene | 2 | HMDB0035772 |
| Nordihydrocapsiate | Phenolic acids | Phenolic acids | 1 | HMDB0034779 |
| Norepinephrine | Hormones and hormone related compounds | Hormones and hormone related compounds | 2 | HMDB0000216 |
| Norfloxacin | Others | Medicine | 2 | HMDB0015192 |
| Norfludiazepam | Heterocyclic compounds | Heterocyclic compounds | 2 | HMDB0061161 |
| Nα-Acetyl-L-Arginine | Amino acid and Its metabolites | Amino acid derivatives | 1 | HMDB0004620 |
| Nα-Acetyl-L-glutamine | Amino acid and Its metabolites | Amino acid derivatives | 1 | HMDB0006029 |
| O-Acetyl-L-serine | Amino acid and Its metabolites | Amino acid derivatives | 2 | HMDB0003011 |
| O-Phospho-L-Serine | Amino acid and Its metabolites | Amino acid derivatives | 2 | HMDB0000272 |
| O-Phosphorylethanolamine | GP | Others | 2 | HMDB0000224 |
| ONO-8711 | Benzene and substituted derivatives | Benzene and substituted derivatives | 2 | - |
| Obacunone | Terpenoids | Triterpene | 2 | HMDB0035858 |
| Octadecanedioic acid | Organic acid and Its derivatives | Organic acid and Its derivatives | 2 | HMDB0000782 |
| Octyl-Beta-D-Glucopyranoside | Carbohydrates and Its metabolites | Sugars | 2 | - |
| Omeprazole sulfone | Aldehyde,Ketones,Esters | Ketones | 2 | HMDB0014012 |
| Orotidine | Nucleotide and Its metabolites | Nucleotide and Its metabolites | 2 | HMDB0000788 |
| Oryzalin | Benzene and substituted derivatives | Benzene and substituted derivatives | 2 | - |
| Ouabagenin | Steroids | Steroidal saponins | 2 | - |
| Ox bile extract | Organic acid and Its derivatives | Organic acid and Its derivatives | 1 | HMDB0249206 |
| Oxaceprol | Amino acid and Its metabolites | Amino acid derivatives | 2 | - |
| Oxaloacetic acid | Organic acid and Its derivatives | Organic acid and Its derivatives | 2 | HMDB0000223 |
| Oxazepam | Heterocyclic compounds | Heterocyclic compounds | 2 | HMDB0014980 |
| Oxidopamine | Alcohol and amines | Polyamines | 1 | HMDB0001537 |
| Oxycanthine | Lignans and Coumarins | Lignans | 2 | HMDB0030172 |
| Oxypurinol | Nucleotide and Its metabolites | Nucleotide and Its metabolites | 2 | HMDB0000786 |
| P-Coumaric Acid | Benzene and substituted derivatives | Phenolic acids | 1 | HMDB0002035 |
| PA(18:1(9Z)/18:1(9Z)) | GP | PA | 2 | HMDB0007865 |
| PA(5:0/18:0) | GP | PA | 2 | HMDB0115486 |
| PAF C-18:1 | GP | LPC | 2 | HMDB0011148 |
| PC(12:0/12:0) | GP | PC | 1 | - |
| PC(16:0/2:0) | GP | PC | 1 | - |
| PC(34:2) | GP | PC | 2 | - |
| PC(36:3) | GP | PC | 2 | - |
| PC(O-1:0/O-16:0) | GP | PC-O | 1 | - |
| PI(15:0/2:0) | GP | PI | 2 | - |
| Palonosetron hydrochloride | Others | Medicine | 2 | - |
| Pantetheine | CoEnzyme and vitamins | CoEnzyme and vitamins | 2 | HMDB0003426 |
| Pantothenate | CoEnzyme and vitamins | CoEnzyme and vitamins | 2 | HMDB0000210 |
| Pantothenol | CoEnzyme and vitamins | CoEnzyme and vitamins | 2 | HMDB0004231 |
| Pelargonin | Flavonoids | Anthocyanidins | 2 | HMDB0033681 |
| Pendimethalin | Benzene and substituted derivatives | Benzene and substituted derivatives | 2 | HMDB0256221 |
| Pentostatin | Heterocyclic compounds | Heterocyclic compounds | 2 | HMDB0014692 |
| Perseitol | Alcohol and amines | Alcohols | 2 | HMDB0033750 |
| Phe-Ala | Amino acid and Its metabolites | Small Peptide | 2 | HMDB0028988 |
| Phe-Ala-Leu | Amino acid and Its metabolites | Small Peptide | 2 | - |
| Phe-Asn | Amino acid and Its metabolites | Small Peptide | 2 | - |
| Phe-Asn-Ile-Glu | Amino acid and Its metabolites | Small Peptide | 2 | - |
| Phe-Asn-Thr-Lys | Amino acid and Its metabolites | Small Peptide | 2 | - |
| Phe-Asp | Amino acid and Its metabolites | Small Peptide | 2 | - |
| Phe-Asp-Phe-Gln-Asn | Amino acid and Its metabolites | Small Peptide | 2 | - |
| Phe-Cys-Thr | Amino acid and Its metabolites | Small Peptide | 2 | - |
| Phe-Gln-Ala-Arg | Amino acid and Its metabolites | Small Peptide | 2 | - |
| Phe-Gln-Asp-Arg | Amino acid and Its metabolites | Small Peptide | 2 | - |
| Phe-Glu | Amino acid and Its metabolites | Small Peptide | 2 | HMDB0028994 |
| Phe-Gly | Amino acid and Its metabolites | Small Peptide | 2 | - |
| Phe-Hyp | Amino acid and Its metabolites | Amino acid derivatives | 1 | HMDB0011176 |
| Phe-Ile-Asp-Leu-Asn | Amino acid and Its metabolites | Small Peptide | 2 | - |
| Phe-Ile-Gln-Lys | Amino acid and Its metabolites | Small Peptide | 2 | - |
| Phe-Ile-Val-Gly-Asp | Amino acid and Its metabolites | Small Peptide | 2 | - |
| Phe-Leu-Gln-Lys | Amino acid and Its metabolites | Small Peptide | 2 | - |
| Phe-Leu-Tyr-Asp | Amino acid and Its metabolites | Small Peptide | 2 | - |
| Phe-Leu-Val-Gly-Gly | Amino acid and Its metabolites | Small Peptide | 2 | - |
| Phe-Lys | Amino acid and Its metabolites | Small Peptide | 2 | - |
| Phe-Lys-Asp | Amino acid and Its metabolites | Small Peptide | 2 | - |
| Phe-Met | Amino acid and Its metabolites | Small Peptide | 1 | - |
| Phe-Phe | Amino acid and Its metabolites | Small Peptide | 1 | HMDB0013302 |
| Phe-Phe-Val | Amino acid and Its metabolites | Small Peptide | 2 | - |
| Phe-Pro | Amino acid and Its metabolites | Amino acid derivatives | 1 | HMDB0011177 |
| Phe-Ser | Amino acid and Its metabolites | Small Peptide | 2 | HMDB0029004 |
| Phe-Ser-Leu-Phe-Asp | Amino acid and Its metabolites | Small Peptide | 2 | - |
| Phe-Ser-Met | Amino acid and Its metabolites | Small Peptide | 2 | - |
| Phe-Thr | Amino acid and Its metabolites | Small Peptide | 2 | HMDB0029005 |
| Phe-Thr-Lys-Lys | Amino acid and Its metabolites | Small Peptide | 2 | - |
| Phe-Thr-Thr | Amino acid and Its metabolites | Small Peptide | 2 | - |
| Phe-Trp | Amino acid and Its metabolites | Small Peptide | 2 | HMDB0029006 |
| Phe-Tyr-Val-Lys | Amino acid and Its metabolites | Small Peptide | 2 | - |
| Phe-Val | Amino acid and Its metabolites | Small Peptide | 2 | HMDB0029008 |
| Phe-Val-Ser-Arg | Amino acid and Its metabolites | Small Peptide | 2 | - |
| Phe4Cl-Asp-OH | Amino acid and Its metabolites | Small Peptide | 2 | - |
| Phe4Cl-Lys-OH | Amino acid and Its metabolites | Small Peptide | 2 | - |
| Phe4Cl-Tyr-OH | Amino acid and Its metabolites | Small Peptide | 2 | - |
| Phencyclidine | Others | Others | 2 | HMDB0256399 |
| Phenethylamine | Alcohol and amines | Amines | 1 | HMDB0012275 |
| Phenoxyacetic acid | Benzene and substituted derivatives | Phenolic acids | 2 | HMDB0031609 |
| Phenylacetyl-L-Glutamine | Amino acid and Its metabolites | Amino acid derivatives | 1 | HMDB0006344 |
| Phenylbutyrylglutamine | Alcohol and amines | Amines | 2 | HMDB0011687 |
| Phosphatidylethanolamine lyso alkenyl 16:0 | GP | LPE | 2 | - |
| Phosphatidylethanolamine lyso alkenyl 18:3 | GP | LPE | 2 | - |
| Phosphoenolpyruvate | Organic acid and Its derivatives | Phosphoric acids | 2 | HMDB0000263 |
| Phosphonoacetic acid | Organic acid and Its derivatives | Phosphoric acids | 1 | HMDB0004110 |
| Phytolaccasaponin G | Terpenoids | Triterpene | 2 | HMDB0034636 |
| Phytosphingosine | SL | SPH | 2 | HMDB0004610 |
| Pinolenic acid | FA | FFA | 2 | - |
| Piperacillin | Others | Medicine | 2 | HMDB0014464 |
| Piperazine | Heterocyclic compounds | Heterocyclic compounds | 2 | - |
| Piperic acid | Organic acid and Its derivatives | Organic acid and Its derivatives | 2 | HMDB0032613 |
| Piperidine acid | Organic acid and Its derivatives | Organic acid and Its derivatives | 1 | HMDB0005960 |
| Piperidone | Heterocyclic compounds | Heterocyclic compounds | 2 | - |
| Piperine | Heterocyclic compounds | Heterocyclic compounds | 1 | HMDB0029377 |
| Ponatinib | Benzene and substituted derivatives | Benzene and substituted derivatives | 2 | HMDB0240214 |
| Porphobilinogen | Organic acid and Its derivatives | Organic acid and Its derivatives | 2 | HMDB0000245 |
| Pramipexole dihydrochloride | Others | Medicine | 1 | - |
| Prasterone enanthate | Hormones and hormone related compounds | Hormones and hormone related compounds | 2 | - |
| Prednisone | Hormones and hormone related compounds | Hormones and hormone related compounds | 2 | HMDB0014773 |
| Pregnanetriol | Alcohol and amines | Alcohols | 2 | - |
| Pro-Arg | Amino acid and Its metabolites | Small Peptide | 2 | - |
| Pro-Asn | Amino acid and Its metabolites | Small Peptide | 2 | - |
| Pro-Asn-Gln-Met-Ser | Amino acid and Its metabolites | Small Peptide | 2 | - |
| Pro-Asp | Amino acid and Its metabolites | Small Peptide | 2 | - |
| Pro-Gln-Gln-Asp-Glu | Amino acid and Its metabolites | Small Peptide | 2 | - |
| Pro-Glu-Glu-Leu-Lys | Amino acid and Its metabolites | Small Peptide | 2 | - |
| Pro-Gly-Met-Ile-Lys | Amino acid and Its metabolites | Small Peptide | 2 | - |
| Pro-His-Arg | Amino acid and Its metabolites | Small Peptide | 2 | - |
| Pro-His-Phe-Asp-Lys | Amino acid and Its metabolites | Small Peptide | 2 | - |
| Pro-Ile | Amino acid and Its metabolites | Small Peptide | 2 | - |
| Pro-Ile-His-Asp-Arg | Amino acid and Its metabolites | Small Peptide | 2 | - |
| Pro-Lys | Amino acid and Its metabolites | Small Peptide | 2 | - |
| Pro-Lys-Lys-Val-Gly | Amino acid and Its metabolites | Small Peptide | 2 | - |
| Pro-Pro-Asp-Gln-Gln | Amino acid and Its metabolites | Small Peptide | 2 | - |
| Pro-Ser | Amino acid and Its metabolites | Small Peptide | 1 | - |
| Procymidone | Aldehyde,Ketones,Esters | Ketones | 2 | HMDB0256790 |
| Proline betaine | Amino acid and Its metabolites | Amino acid derivatives | 1 | HMDB0004827 |
| Prolyl-Histidine | Amino acid and Its metabolites | Small Peptide | 2 | HMDB0029019 |
| Prolylhistidine | Amino acid and Its metabolites | Small Peptide | 2 | HMDB0029019 |
| Prolyltryptophan | Amino acid and Its metabolites | Small Peptide | 2 | HMDB0029028 |
| Propofol | Benzene and substituted derivatives | Benzene and substituted derivatives | 2 | HMDB0014956 |
| Propylparaben | Benzene and substituted derivatives | Phenolic acids | 2 | HMDB0032574 |
| Prosapogenin A | Steroids | Steroidal saponins | 2 | HMDB0029312 |
| Prostaglandin K2 | Hormones and hormone related compounds | Hormones and hormone related compounds | 2 | - |
| Prosulfocarb | Benzene and substituted derivatives | Benzene and substituted derivatives | 2 | - |
| Prothioconazole | Benzene and substituted derivatives | Benzene and substituted derivatives | 2 | - |
| Pseudotropine | Others | Others | 2 | - |
| Purine | Nucleotide and Its metabolites | Nucleotide and Its metabolites | 2 | HMDB0001366 |
| Pyrazine-2-carboxylic acid | Organic acid and Its derivatives | Organic acid and Its derivatives | 2 | HMDB0059734 |
| Pyridoxamine | Heterocyclic compounds | Pteridines and derivatives | 1 | HMDB0001431 |
| Pyridoxamine phosphate | Organic acid and Its derivatives | Organic acid and Its derivatives | 2 | HMDB0001555 |
| Pyrimidine-4-carboxylic acid | Heterocyclic compounds | Heterocyclic compounds | 2 | - |
| Pyrocatechol | Benzene and substituted derivatives | Phenolics | 1 | HMDB0000957 |
| Pyroglutamic acid | Amino acid and Its metabolites | Amino acids | 1 | HMDB0000267 |
| Pyroquilon | Aldehyde,Ketones,Esters | Ketones | 2 | HMDB0037113 |
| Pyrrolidine | Heterocyclic compounds | Heterocyclic compounds | 1 | HMDB0031641 |
| Pyrroloquinoline Quinone | Organic acid and Its derivatives | Organic acid and Its derivatives | 2 | HMDB0013636 |
| Quinmerac | Benzene and substituted derivatives | Benzene and substituted derivatives | 2 | HMDB0257043 |
| Quinoline-4-carboxylic acid | Heterocyclic compounds | Pteridines and derivatives | 2 | - |
| Quinolinic acid | Heterocyclic compounds | Heterocyclic compounds | 2 | HMDB0000232 |
| Quizalofop | Benzene and substituted derivatives | Benzene and substituted derivatives | 2 | - |
| Ramiprilat | Amino acid and Its metabolites | Amino acid derivatives | 2 | HMDB0060579 |
| Raphanusamic acid | Organic acid and Its derivatives | Organic acid and Its derivatives | 2 | HMDB0041280 |
| Rauwolscine | Benzene and substituted derivatives | Benzene and substituted derivatives | 2 | - |
| Resolvin D2 | FA | Oxidized lipids | 2 | - |
| Rhizoxin | Heterocyclic compounds | Heterocyclic compounds | 2 | - |
| Riboflavin | CoEnzyme and vitamins | CoEnzyme and vitamins | 1 | HMDB0000244 |
| Rimantadine | Alcohol and amines | Amines | 2 | HMDB0014621 |
| Rinderine | Alkaloids | Alkaloids | 2 | - |
| Ronidazole | Heterocyclic compounds | Heterocyclic compounds | 2 | - |
| Rutaecarpine | Heterocyclic compounds | Heterocyclic compounds | 1 | HMDB0042010 |
| S-(5-Adenosy)-L-Homocysteine | Amino acid and Its metabolites | Amino acid derivatives | 1 | HMDB0000939 |
| S-Methyl-L-Cysteine-S-oxide | Amino acid and Its metabolites | Amino acid derivatives | 2 | - |
| S-Sulfo-L-Cysteine | Amino acid and Its metabolites | Amino acid derivatives | 2 | HMDB0000731 |
| SDMA | Organic acid and Its derivatives | Organic acid and Its derivatives | 1 | HMDB0003334 |
| SPH(d16:1) | SL | SPH | 2 | - |
| SPH(d18:1) | SL | SPH | 2 | HMDB0000252 |
| ST-638 | Organic acid and Its derivatives | Organic acid and Its derivatives | 2 | - |
| Salannin | Terpenoids | Triterpene | 2 | - |
| Salicylaldehyde | Benzene and substituted derivatives | Benzene and substituted derivatives | 2 | HMDB0034170 |
| Salidroside | Benzene and substituted derivatives | Benzene and substituted derivatives | 2 | - |
| Salvianolic acid A | Organic acid and Its derivatives | Organic acid and Its derivatives | 2 | - |
| Samidin | Heterocyclic compounds | Heterocyclic compounds | 2 | - |
| Sarcosine | Amino acid and Its metabolites | Amino acids | 2 | HMDB0000271 |
| Schisandrin | Heterocyclic compounds | Heterocyclic compounds | 2 | HMDB0258165 |
| Sebacate | Organic acid and Its derivatives | Organic acid and Its derivatives | 1 | HMDB0000792 |
| Securinine | Amino acid and Its metabolites | Amino acid derivatives | 2 | - |
| Sedoheptulose | Carbohydrates and Its metabolites | Carbohydrates and Its metabolites | 1 | HMDB0003219 |
| Selexipag | Heterocyclic compounds | Heterocyclic compounds | 2 | - |
| Ser-Ala | Amino acid and Its metabolites | Amino acid derivatives | 2 | - |
| Ser-Arg-Glu-Val-Ser | Amino acid and Its metabolites | Small Peptide | 2 | - |
| Ser-Arg-Tyr-Glu | Amino acid and Its metabolites | Small Peptide | 2 | - |
| Ser-Gln-Asp-Val-Ser | Amino acid and Its metabolites | Small Peptide | 2 | - |
| Ser-Gln-Gly | Amino acid and Its metabolites | Small Peptide | 2 | - |
| Ser-Glu | Amino acid and Its metabolites | Small Peptide | 2 | - |
| Ser-Glu-Lys-Ile-Asp | Amino acid and Its metabolites | Small Peptide | 2 | - |
| Ser-Glu-Phe-Arg | Amino acid and Its metabolites | Small Peptide | 2 | - |
| Ser-His-Val-Lys | Amino acid and Its metabolites | Small Peptide | 2 | - |
| Ser-Ile-His-Arg | Amino acid and Its metabolites | Small Peptide | 2 | - |
| Ser-Leu | Amino acid and Its metabolites | Small Peptide | 2 | HMDB0029043 |
| Ser-Leu-Glu-Asp | Amino acid and Its metabolites | Small Peptide | 2 | - |
| Ser-Lys | Amino acid and Its metabolites | Small Peptide | 2 | HMDB0029044 |
| Ser-Pro | Amino acid and Its metabolites | Small Peptide | 2 | - |
| Ser-Pro-Thr-Phe-Leu | Amino acid and Its metabolites | Small Peptide | 2 | - |
| Ser-Ser | Amino acid and Its metabolites | Small Peptide | 2 | - |
| Ser-Tyr-Arg | Amino acid and Its metabolites | Small Peptide | 2 | - |
| Ser-Val | Amino acid and Its metabolites | Small Peptide | 2 | HMDB0029052 |
| Ser-Val-Lys-Arg | Amino acid and Its metabolites | Small Peptide | 2 | - |
| Sericetin | Heterocyclic compounds | Heterocyclic compounds | 2 | - |
| Serotonin | Tryptamines,Cholines,Pigments | Tryptamines | 1 | HMDB0000259 |
| Sethoxydim | Aldehyde,Ketones,Esters | Ketones | 2 | - |
| Sinensetin | Flavonoids | Flavonoid | 2 | HMDB0036633 |
| Skimmianine | Benzene and substituted derivatives | Benzene and substituted derivatives | 2 | HMDB0258333 |
| Sn-Glycero-3-Phosphocholine | Tryptamines,Cholines,Pigments | Cholines | 1 | HMDB0000086 |
| Sodium cholate | Others | Medicine | 1 | - |
| Sodium dodecyl sulfate | Others | Others | 2 | - |
| Spermidic acid | Organic acid and Its derivatives | Organic acid and Its derivatives | 2 | HMDB0002201 |
| Spermidine | Alcohol and amines | Polyamines | 2 | HMDB0001257 |
| Subericacid | Organic acid and Its derivatives | Organic acid and Its derivatives | 1 | HMDB0000893 |
| Succinic Acid | Organic acid and Its derivatives | Organic acid and Its derivatives | 1 | HMDB0000254 |
| Sulfachlorpyridazine | Benzene and substituted derivatives | Benzene and substituted derivatives | 2 | - |
| Sulfadimethoxine | Benzene and substituted derivatives | Benzene and substituted derivatives | 1 | HMDB0015621 |
| Sulfamethazine | Benzene and substituted derivatives | Benzene and substituted derivatives | 2 | HMDB0015522 |
| Synephrine | Benzene and substituted derivatives | Phenolic acids | 2 | HMDB0004826 |
| Syringetin-3-o-glucoside | Flavonoids | Flavonoid | 2 | - |
| TXB2 | FA | Oxidized lipids | 2 | HMDB0003252 |
| Tacalcitol | CoEnzyme and vitamins | CoEnzyme and vitamins | 2 | - |
| Tamibarotene | Benzene and substituted derivatives | Benzene and substituted derivatives | 2 | HMDB0015605 |
| Tapentadol | Benzene and substituted derivatives | Benzene and substituted derivatives | 2 | - |
| Taurine | Organic acid and Its derivatives | Sulfonic acids | 1 | HMDB0000251 |
| Taurolithocholic acid | Bile acids | Bile acids | 1 | - |
| Tenovin-6 | Benzene and substituted derivatives | Benzene and substituted derivatives | 2 | - |
| Tenoxicam | Heterocyclic compounds | Heterocyclic compounds | 2 | HMDB0014612 |
| Terbinafine | Benzene and substituted derivatives | Benzene and substituted derivatives | 2 | HMDB0014995 |
| Terephthalic Acid | Benzene and substituted derivatives | Phenolic acids | 2 | HMDB0002428 |
| Testosterone | Hormones and hormone related compounds | Hormones and hormone related compounds | 2 | HMDB0000234 |
| Tetracaine | Benzene and substituted derivatives | Benzene and substituted derivatives | 2 | HMDB0258868 |
| Tetrachlorosalicylanilide | Benzene and substituted derivatives | Phenolics | 2 | - |
| Tetradecanedioic acid | FA | FFA | 1 | HMDB0000872 |
| Tetraethylammonium | Alcohol and amines | Amines | 2 | - |
| Tetraethylene-glycol | Alcohol and amines | Alcohols | 2 | HMDB0094708 |
| Tetragastrin | Amino acid and Its metabolites | Amino acid derivatives | 2 | HMDB0005775 |
| Tetramisole | Heterocyclic compounds | Heterocyclic compounds | 2 | - |
| Theobromine | Nucleotide and Its metabolites | Nucleotide and Its metabolites | 2 | HMDB0002825 |
| Thiacloprid | Heterocyclic compounds | Heterocyclic compounds | 2 | HMDB0258967 |
| Thiamine | CoEnzyme and vitamins | CoEnzyme and vitamins | 1 | HMDB0000235 |
| Thioctic acid | Organic acid and Its derivatives | Organic acid and Its derivatives | 2 | HMDB0251530 |
| Thr-Arg | Amino acid and Its metabolites | Small Peptide | 2 | - |
| Thr-Asp | Amino acid and Its metabolites | Small Peptide | 2 | - |
| Thr-Gln | Amino acid and Its metabolites | Small Peptide | 1 | - |
| Thr-Glu-Ala | Amino acid and Its metabolites | Small Peptide | 2 | - |
| Thr-His-Gly | Amino acid and Its metabolites | Small Peptide | 2 | - |
| Thr-Ile-Val-Arg | Amino acid and Its metabolites | Small Peptide | 2 | - |
| Thr-Lys-Gln-Lys | Amino acid and Its metabolites | Small Peptide | 2 | - |
| Thr-Met | Amino acid and Its metabolites | Small Peptide | 2 | - |
| Thr-Phe | Amino acid and Its metabolites | Small Peptide | 2 | HMDB0029068 |
| Thr-Ser-Lys | Amino acid and Its metabolites | Small Peptide | 2 | - |
| Thr-Tyr-Arg-Lys | Amino acid and Its metabolites | Small Peptide | 2 | - |
| Thr-Val-Ile-Thr-Gly | Amino acid and Its metabolites | Small Peptide | 2 | - |
| Thr-Val-Leu | Amino acid and Its metabolites | Small Peptide | 2 | - |
| Thr-Val-Leu-Thr-Ser | Amino acid and Its metabolites | Small Peptide | 2 | - |
| Thr-Val-Lys-Glu | Amino acid and Its metabolites | Small Peptide | 2 | - |
| Thr-Val-Thr-Phe-Tyr | Amino acid and Its metabolites | Small Peptide | 2 | - |
| Threoninyl-Isoleucine | Amino acid and Its metabolites | Small Peptide | 2 | HMDB0029064 |
| Thymidine-5'-triphosphate | Nucleotide and Its metabolites | Nucleotide and Its metabolites | 2 | HMDB0001342 |
| Thymolphthalein | Heterocyclic compounds | Heterocyclic compounds | 2 | - |
| Tiaprofenic acid | Organic acid and Its derivatives | Organic acid and Its derivatives | 2 | HMDB0015538 |
| Tobramycin | Heterocyclic compounds | Heterocyclic compounds | 2 | HMDB0014822 |
| Tomatidine | Alkaloids | Steroid alkaloids | 2 | HMDB0034731 |
| Tranilast | Heterocyclic compounds | Heterocyclic compounds | 2 | - |
| Trans-3-Hydroxycotinine | Heterocyclic compounds | Heterocyclic compounds | 1 | HMDB0001390 |
| Traumatic acid | Organic acid and Its derivatives | Organic acid and Its derivatives | 1 | HMDB0000933 |
| Triacetin | Aldehyde,Ketones,Esters | Esters | 1 | HMDB0029592 |
| Triamcinolone acetonide | Hormones and hormone related compounds | Hormones and hormone related compounds | 2 | - |
| Tricyclazole | Heterocyclic compounds | Heterocyclic compounds | 2 | HMDB0031809 |
| Tridecanedioic acid | FA | FFA | 1 | - |
| Triethanolamine | Alcohol and amines | Alcohols | 2 | HMDB0032538 |
| Triethyl-phosphate | Organic acid and Its derivatives | Phosphoric acids | 2 | HMDB0012228 |
| Triethylamine | Others | Hydrocarbon derivatives | 2 | HMDB0032539 |
| Triethylenetetramine | Alcohol and amines | Polyamines | 2 | - |
| Trigonelline | CoEnzyme and vitamins | CoEnzyme and vitamins | 2 | HMDB0000875 |
| Trimethylamine-N-Oxide | Alcohol and amines | Amines | 1 | HMDB0000925 |
| Tripropylamine | Alcohol and amines | Amines | 2 | HMDB0032545 |
| Trolox | Benzene and substituted derivatives | Benzene and substituted derivatives | 2 | - |
| Trp-Gly | Amino acid and Its metabolites | Small Peptide | 2 | - |
| Trp-Ile | Amino acid and Its metabolites | Small Peptide | 2 | - |
| Trp-Tyr-Tyr | Amino acid and Its metabolites | Small Peptide | 2 | - |
| Trp-Val-Arg | Amino acid and Its metabolites | Small Peptide | 2 | - |
| Tryptamine | Tryptamines,Cholines,Pigments | Tryptamines | 1 | HMDB0000303 |
| Tumonoic acid A | Organic acid and Its derivatives | Organic acid and Its derivatives | 2 | - |
| Tyr-Cys-Trp | Amino acid and Its metabolites | Small Peptide | 2 | - |
| Tyr-Gln-Asn-Glu | Amino acid and Its metabolites | Small Peptide | 2 | - |
| Tyr-Gln-Gln | Amino acid and Its metabolites | Small Peptide | 2 | - |
| Tyr-Gln-Leu-Lys | Amino acid and Its metabolites | Small Peptide | 2 | - |
| Tyr-Gln-Thr-Lys | Amino acid and Its metabolites | Small Peptide | 2 | - |
| Tyr-Glu | Amino acid and Its metabolites | Small Peptide | 2 | - |
| Tyr-Glu-Gln-Asp | Amino acid and Its metabolites | Small Peptide | 2 | - |
| Tyr-Glu-Val-Lys | Amino acid and Its metabolites | Small Peptide | 2 | - |
| Tyr-His-Arg-Arg | Amino acid and Its metabolites | Small Peptide | 2 | - |
| Tyr-His-Phe-Lys | Amino acid and Its metabolites | Small Peptide | 2 | - |
| Tyr-Leu | Amino acid and Its metabolites | Small Peptide | 2 | HMDB0029109 |
| Tyr-Leu-Ala-Lys | Amino acid and Its metabolites | Small Peptide | 2 | - |
| Tyr-Lys-Arg-Glu | Amino acid and Its metabolites | Small Peptide | 2 | - |
| Tyr-Lys-Val-Glu-Ile | Amino acid and Its metabolites | Small Peptide | 2 | - |
| Tyr-Phe-Asp-Arg | Amino acid and Its metabolites | Small Peptide | 2 | - |
| Tyr-Phe-Thr-Lys | Amino acid and Its metabolites | Small Peptide | 2 | - |
| Tyr-Ser | Amino acid and Its metabolites | Small Peptide | 2 | - |
| Tyr-Val-Ser-Arg | Amino acid and Its metabolites | Small Peptide | 2 | - |
| Unoprostone | FA | Oxidized lipids | 2 | - |
| Uracil | Nucleotide and Its metabolites | Nucleotide and Its metabolites | 1 | HMDB0000300 |
| Urea | Alcohol and amines | Amines | 1 | HMDB0000294 |
| Ureidoisobutyric Acid | Organic acid and Its derivatives | Organic acid and Its derivatives | 2 | HMDB0002031 |
| Ureidosuccinic acid | Amino acid and Its metabolites | Amino acid derivatives | 2 | HMDB0000828 |
| Uric acid | Organic acid and Its derivatives | Organic acid and Its derivatives | 1 | HMDB0000289 |
| Uridine | Nucleotide and Its metabolites | Nucleotide and Its metabolites | 1 | HMDB0000296 |
| Uridine triacetate | Others | Medicine | 1 | - |
| Urobilin | Tryptamines,Cholines,Pigments | Pigments | 1 | - |
| Urocanic Acid | Organic acid and Its derivatives | Organic acid and Its derivatives | 1 | HMDB0000301 |
| Val-Ala | Amino acid and Its metabolites | Small Peptide | 2 | - |
| Val-Arg | Amino acid and Its metabolites | Small Peptide | 2 | HMDB0029121 |
| Val-Arg-His-Arg | Amino acid and Its metabolites | Small Peptide | 2 | - |
| Val-Asn-Val-Asp-Glu | Amino acid and Its metabolites | Small Peptide | 2 | - |
| Val-Gly | Amino acid and Its metabolites | Small Peptide | 2 | HMDB0029127 |
| Val-His-Leu-Asp | Amino acid and Its metabolites | Small Peptide | 2 | - |
| Val-Ile-Asp-Lys | Amino acid and Its metabolites | Small Peptide | 2 | - |
| Val-Ile-Leu-Asp | Amino acid and Its metabolites | Small Peptide | 2 | - |
| Val-Ile-Lys-Asp | Amino acid and Its metabolites | Small Peptide | 2 | - |
| Val-Ile-Pro-Lys-Ser | Amino acid and Its metabolites | Small Peptide | 2 | - |
| Val-Leu-Asp-Phe-Glu | Amino acid and Its metabolites | Small Peptide | 2 | - |
| Val-Leu-Leu-Ser-Cys | Amino acid and Its metabolites | Small Peptide | 2 | - |
| Val-Leu-Ser-Pro-Ala | Amino acid and Its metabolites | Small Peptide | 2 | - |
| Val-Phe-Asp-Arg | Amino acid and Its metabolites | Small Peptide | 2 | - |
| Val-Phe-Phe-Asn-Gly | Amino acid and Its metabolites | Small Peptide | 2 | - |
| Val-Thr | Amino acid and Its metabolites | Small Peptide | 2 | - |
| Val-Thr-Tyr-Lys | Amino acid and Its metabolites | Small Peptide | 2 | - |
| Val-Thr-Val-Leu-Val | Amino acid and Its metabolites | Small Peptide | 2 | - |
| Val-Tyr-Gln-His-Val | Amino acid and Its metabolites | Small Peptide | 2 | - |
| Valproic acid | Organic acid and Its derivatives | Organic acid and Its derivatives | 2 | HMDB0001877 |
| Valylserine | Amino acid and Its metabolites | Small Peptide | 2 | HMDB0029136 |
| Vanillic acid 4-O-sulfate | Organic acid and Its derivatives | Sulfonic acids | 2 | HMDB0041788 |
| Verapamil | Benzene and substituted derivatives | Benzene and substituted derivatives | 2 | HMDB0001850 |
| Veratridine | Alkaloids | Alkaloids | 2 | - |
| Veratrine | Aldehyde,Ketones,Esters | Esters | 2 | - |
| Vitamin K | CoEnzyme and vitamins | CoEnzyme and vitamins | 2 | HMDB0015157 |
| Vorinostat | Benzene and substituted derivatives | Benzene and substituted derivatives | 2 | HMDB0015568 |
| Vulpinic acid | Benzene and substituted derivatives | Benzene and substituted derivatives | 2 | - |
| Xanthine | Nucleotide and Its metabolites | Nucleotide and Its metabolites | 1 | HMDB0000292 |
| Xanthosine | Nucleotide and Its metabolites | Nucleotide and Its metabolites | 1 | HMDB0000299 |
| Zearalanone | Aldehyde,Ketones,Esters | Ketones | 2 | - |
| Zidovudine | Nucleotide and Its metabolites | Nucleotide and Its metabolites | 2 | HMDB0014638 |
| Zoledronic acid | Organic acid and Its derivatives | Organic acid and Its derivatives | 2 | HMDB0014543 |
| Zonisamide | Benzene and substituted derivatives | Benzene and substituted derivatives | 2 | HMDB0015045 |
| [(1R)-1-(3-aminophenyl)-3-(3,4-dimethoxyphenyl)propyl] (2S)-1-(3,3-dimethyl-2-oxopentanoyl)piperidine-2-carboxylate | Heterocyclic compounds | Heterocyclic compounds | 2 | - |
| [(2S,6S,9R)-4,4,11,11-tetramethyl-3,5,7,10,12-pentaoxatricyclo[7.3.0.02,6]dodecan-6-yl]methyl sulfamate | Heterocyclic compounds | Heterocyclic compounds | 2 | HMDB0005034 |
| acetic acid [(1R,4bR,5R,8R,10S,10aS,12aS)-1-(3-furanyl)-8,10-dihydroxy-4b,7,7,10a,12a-pentamethyl-3-oxo-1,5,6,6a,8,9,10,10b,11,12-decahydronaphtho[2,1-f][2]benzopyran-5-yl] ester | Heterocyclic compounds | Heterocyclic compounds | 2 | - |
| aldehydo-D-ribose | Carbohydrates and Its metabolites | Sugars | 1 | - |
| alpha-CYANO-3-HYDROXYCINNAMIC ACID | Organic acid and Its derivatives | Organic acid and Its derivatives | 2 | - |
| alpha-D-Glucopyranoside,-beta-D-fructofuranosyl | Carbohydrates and Its metabolites | Sugars | 2 | - |
| alpha-Glutamylaspartic acid | Amino acid and Its metabolites | Small Peptide | 2 | HMDB0028815 |
| alpha-Ionone | Aldehyde,Ketones,Esters | Ketones | 2 | HMDB0059883 |
| alpha-Mangostin | Heterocyclic compounds | Heterocyclic compounds | 2 | HMDB0035796 |
| alpha-Methoxy-1H-indole-3-propanoic acid | Heterocyclic compounds | Indole and Its derivatives | 2 | HMDB0038339 |
| antimycin A1 | Benzene and substituted derivatives | Benzene and substituted derivatives | 2 | HMDB0248488 |
| benzyl 4-(1,3-dioxoisoindolin-2-yl)-2',2'-dimethyl-3',5-dioxo-2',3',4,5-tetrahydro-3H-spiro[furan-2,9'-imidazo[1,2-a]indole]-1'(9a'H)-carboxylate | Heterocyclic compounds | Indole and Its derivatives | 2 | - |
| brefeldin A | Heterocyclic compounds | Heterocyclic compounds | 2 | - |
| cyclo(glu-glu) | Amino acid and Its metabolites | Small Peptide | 2 | - |
| cyclo(gly-glu) | Amino acid and Its metabolites | Small Peptide | 2 | - |
| cyclo(pro-pro) | Amino acid and Its metabolites | Small Peptide | 2 | - |
| estrone 3-sulfate | Hormones and hormone related compounds | Hormones and hormone related compounds | 1 | HMDB0001425 |
| gamma-CEHC | Organic acid and Its derivatives | Organic acid and Its derivatives | 2 | - |
| gamma-Glu-Ala | Amino acid and Its metabolites | Small Peptide | 2 | HMDB0006248 |
| gamma-Glu-Phe | Amino acid and Its metabolites | polypeptide | 2 | HMDB0000594 |
| m-chlorophenylpiperazine (m-CPP) | Heterocyclic compounds | Heterocyclic compounds | 2 | HMDB0061008 |
| methylcarbamyl PAF | FA | Others | 2 | - |
| n,n-Diisopropyl-3-nitrobenzamide | Benzene and substituted derivatives | Benzene and substituted derivatives | 2 | - |
| n-Benzylacetamidine | Benzene and substituted derivatives | Benzene and substituted derivatives | 2 | - |
| p-Cresol glucuronide | Benzene and substituted derivatives | Benzene and substituted derivatives | 2 | - |
| prostaglandin E3 | Hormones and hormone related compounds | Hormones and hormone related compounds | 2 | HMDB0002664 |
| scytophycin C | Heterocyclic compounds | Heterocyclic compounds | 2 | - |
| stigmasta-7,22E-dien-3-ol | Steroids | Steroid | 2 | - |
| trans-4-Methylene-2-octyl-5-oxotetrahydrofuran-3-carboxylic acid | Heterocyclic compounds | Heterocyclic compounds | 2 | - |
| xi-Tetrahydro-6-propyl-2H-pyran-2-one | Aldehyde,Ketones,Esters | Ketones | 2 | HMDB0038310 |
| Β-Nicotinamide Mononucleotide | Nucleotide and Its metabolites | Nucleotide and Its metabolites | 2 | HMDB0000229 |
| Β-Pseudouridine | Nucleotide and Its metabolites | Nucleotide and Its metabolites | 1 | HMDB0000767 |
| γ-Aminobutyric Acid | Amino acid and Its metabolites | Amino acid derivatives | 2 | HMDB0000112 |
| γ-Glu-Cys | Amino acid and Its metabolites | Small Peptide | 2 | HMDB0001049 |
| γ-Glu-Met | Amino acid and Its metabolites | Small Peptide | 2 | - |
| notes: FA, fatty acids; GP, glycerophospholipids; SL, sphingolipids; GL, glycerolipids. | | | | |

| **Table S2. Comparison of targeted metabolites measured in Xiazhi with metabolites in Liuheng** | | |
| --- | --- | --- |
| Compound in Xiazhi | Index in Liuheng | Compound in Liuheng |
| LPC(14:0) | MEDP0336 | LPC(0:0/14:0) |
| LPC(15:0) | MEDP1692 | LPC(0:0/15:0) |
| LPC(18:0) | MEDP1700 | LPC(0:0/18:0) |
| LPC(18:0) | MEDP1337 | LPC(18:0/0:0) |
| LPC(18:1) | MEDP1701 | LPC(0:0/18:1) |
| LPC(18:1) | MEDP1339 | LPC(18:1/0:0) |
| LPE(16:0) | MEDN1285 | LPE(0:0/16:0) |
| LPE(16:0) | MEDN0366 | LPE(16:0/0:0) |
| LPE(18:0) | MEDN1279 | LPE(0:0/18:0) |
| LPE(18:0) | MEDN0364 | LPE(18:0/0:0) |
| LPC(13:0) | MEDP1779 | LPC(13:0/0:0) |
| LPC(16:0) | MEDP1346 | LPC(0:0/16:0) |
| LPA(16:0) | MEDN0372 | LPA(0:0/16:0) |
| LPA(16:0) | MEDN1600 | LPA(16:0/0:0) |
| 5,8,11-Eicosatrienoic acid | MW0054695 | 5,8,11-Eicosatrienoic acid |
| Deoxycholic acid(DCA) | MEDN1899 | Deoxycholic acid |
| Chenodeoxycholic acid(CDCA) | MEDN0115 | Chenodeoxycholic Acid |
| Histidine | MEDP0017 | L-Histidine |
| L-phenylalanine | FDATN01519 | D-phenylalanine |
| L-phenylalanine | MEDN0015 | L-Phenylalanine |
| Methionine | MEDP0020 | L-Methionine |
| Glutamate | MW0106392 | D-Glutamic acid |
| Glutamate | MEDN0011*018 | L-Glutamic Acid |
| Cysteine | MW0123496 | D-Cysteine |
| Proline | MEDP0022*108 | L-Proline |
| Glycine | MEDP0006 | L-Glycine |
| Serine | MEDN0018 | L-Serine |
| Tryptophan | MEDP0025 | L-Tryptophan |
| Valine | MEDP0026*110 | L-Valine |
| Leucine | MEDP0752*114 | DL-Leucine |
| Isoleucine | MEDP1144 | L-Isoleucine |
| Glutamine | MEDN0046 | L-Glutamine |
| Lysine | MEDP0011 | L-Lysine |
| L-Arginine | MW0107655 | DL-Arginine |
| L-Arginine | MEDN0007 | L-Arginine |
| L-cystine | MEDP0007 | L-Cystine |
| betaine | MEDP0039 | Betaine |
| TMAO | MEDP0084*101 | Trimethylamine-N-Oxide |
| cis-p-Coumaric acid | MEDP0101 | P-Coumaric Acid |
| Propionyl carnitine | MEDP1665 | Carnitine C3:0 |

| **Table S3. Comparison of metabolites measured in THSBC with metabolites in Liuheng** | | |
| --- | --- | --- |
| Compounds_liuheng | HMDB_liuheng | Compounds_THSBC |
| Indole-3-acetamide | HMDB0029739 | Indole-3-acetamide |
| Serotonin | HMDB0000259 | Serotonin |
| Orotidine | HMDB0000788 | Orotidine |
| Inosine | HMDB0000195 | Inosine |
| Adenosine | HMDB0000050 | Adenosine |
| Guanosine | HMDB0000133 | Guanosine |
| 2-Hydroxyadenosine | - | 2-Hydroxyadenosine |
| cyclo(pro-pro) | - | cyclo(pro-pro) |
| Porphobilinogen | HMDB0000245 | Porphobilinogen |
| 2'-O-methyluridine | HMDB0240328 | 2'-O-methyluridine |
| 3-Methyluridine | HMDB0004813 | 3-Methyluridine |
| cyclo(glu-glu) | - | cyclo(glu-glu) |
| 5-Methyluridine | HMDB0000884 | 5-Methyluridine |
| 2'-O-methylcytidine | - | 2'-O-methylcytidine |
| Cyclo(Pro-Val) | - | Cyclo(Pro-Val) |
| Biotinamide | HMDB0001458 | Biotinamide |
| Glu-Val | HMDB0028832 | Glu-Val |
| Leu-Asp | - | Leu-Asp |
| γ-Glu-Met | - | γ-Glu-Met |
| Glu-Met | HMDB0028825 | Glu-Met |
| Met-Glu | - | Met-Glu |
| Argininosuccinic acid | HMDB0000052 | Argininosuccinic acid |
| 8-Methylnonenoate | HMDB0012183 | (E)-8-Methyl-6-nonenoic-acid |
| Sebacate | HMDB0000792 | Sebacate |
| Arg-Asp | HMDB0028705 | Arg-Asp |
| 2-Octanamidoacetic acid | HMDB0000832 | 2-Octanamidoacetic acid |
| Carnitine C3:0 | HMDB0000824 | Carnitine C3:0 |
| Thr-Arg | - | Thr-Arg |
| 1-acetylindole | - | 1-acetylindole |
| N'-Formylkynurenine | HMDB0001200 | N'-Formylkynurenine |
| L-Tryptophanamide | HMDB0013318 | L-Tryptophanamide |
| N-Acetyl-L-phenylalanine | HMDB0000512 | N-Acetyl-L-phenylalanine |
| 5-Methoxytryptamine | HMDB0004095 | 5-Methoxytryptamine |
| Phe-Gly | - | Phe-Gly |
| 1-Methylinosine | HMDB0002721 | 1-Methylinosine |
| N4-Acetylcytidine | HMDB0005923 | N4-Acetylcytidine |
| 5'-Deoxy-5'-(Methylthio) Adenosine | HMDB0001173 | 5'-Deoxy-5'-(Methylthio) Adenosine |
| 2'-O-Methylguanosine | - | 2'-O-Methylguanosine |
| Cyclo(Pro-Leu) | HMDB0034276 | Cyclo(Pro-Leu) |
| N(Alpha)-Acetyl-Epsilon-(2-Propenal)Lysine | - | N(Alpha)-Acetyl-Epsilon-(2-Propenal)Lysine |
| Carnitine C4:DC | - | Carnitine C4:DC |
| Pro-Ile | - | Pro-Ile |
| Ile-Gln | - | Ile-Gln |
| Arg-Pro | HMDB0028717 | Arg-Pro |
| Ile-Val | - | Ile-Val |
| Leu-Val | HMDB0028942 | Leu-Val |
| N1,N8-diacetylspermidine | HMDB0041947 | N1,N8-diacetylspermidine |
| Quinmerac | HMDB0257043 | Quinmerac |
| 3-Indolebutyric Acid | HMDB0002096 | 3-Indolebutyric Acid |
| N-lactoyl-phenylalanine | HMDB0062175 | N-lactoyl-phenylalanine |
| Phe-Ala | HMDB0028988 | Phe-Ala |
| Phe-Ser | HMDB0029004 | Phe-Ser |
| 3-carboxy-4-methyl-5-propyl-2-furanpropionic acid | - | 3-carboxy-4-methyl-5-propyl-2-furanpropionic acid |
| N6-(2-Hydroxyethyl)adenosine | - | N6-(2-Hydroxyethyl)adenosine |
| Traumatic acid | HMDB0000933 | Traumatic acid |
| Carnitine C5:1 | HMDB0002366 | Carnitine C5:1 |
| Dodecanedioic Acid | HMDB0000623 | Dodecanedioic Aicd |
| Leu-Ile | - | Leu-Ile |
| Bis(1-inositol)-3,1'-phosphate 1-phosphate | - | Bis(1-inositol) -3,1'-phosphate 1-phosphate |
| Ile-Lys | HMDB0028912 | Ile-Lys |
| Lys-Ile | HMDB0028954 | Lys-Ile |
| Lys-Leu | HMDB0028955 | Lys-Leu |
| Hexaethylene-glycol | HMDB0061822 | Hexaethylene-glycol |
| N1-Acetylspermine | HMDB0001186 | N1-Acetylspermine |
| Antineoplaston A10 | - | Antineoplaston A10 |
| Trp-Gly | - | Trp-Gly |
| Securinine | - | Securinine |
| Phenylacetyl-L-Glutamine | HMDB0006344 | Phenylacetyl-L-Glutamine |
| Phe-Asn | - | Phe-Asn |
| Gly-Gly-Phe | - | Gly-Gly-Phe |
| N,N′-dicyclohexylcarbodiimide | - | N,N′-dicyclohexylcarbodiimide |
| 1,3-Dicyclohexylurea | - | 1,3-Dicyclohexylurea |
| Tridecanedioic acid | - | Tridecanedioic acid |
| Carnitine C6:0 | HMDB0000705 | Carnitine C6:0 |
| Cyclo(Phe-Glu) | - | Cyclo(Phe-Glu) |
| Phe-Pro | HMDB0011177 | Phe-Pro |
| Phe-Hyp | HMDB0011176 | Phe-Hyp |
| Glu-Tyr | HMDB0028831 | Glu-Tyr |
| Tyr-Glu | - | Tyr-Glu |
| 1-O-vanillyl-β-D-glucose | - | 1-O-vanillyl-β-D-glucose |
| Gln-Phe | HMDB0028804 | Gln-Phe |
| Carnitine ph-C1 | - | Carnitine ph-C1 |
| Phe-Val | HMDB0029008 | Phe-Val |
| Phe-Met | - | Phe-Met |
| CMPentylF | - | CMPentylF |
| N-acetyl-D-Lactosamine | HMDB0001542 | N-acetyl-D-Lactosamine |
| Carnitine C7:1 Isomer1 | - | Carnitine C7:1Isomer1 |
| Carnitine C7:1 | - | Carnitine C7:1 |
| Tetradecanedioic acid | HMDB0000872 | Tetradecanedioic acid |
| FFA(14:0) | HMDB0000806 | FFA(14:0) |
| (R)-3-Hydroxy-tetradecanoic acid | HMDB0010731 | (R)-3-Hydroxymyristic acid |
| Daidzein | HMDB0003312 | Daidzein |
| 3,3',5-Triiodo-L-Thyronine | HMDB0000265 | 3,3',5-Triiodo-L-Thyronine |
| gamma-CEHC | - | gamma-CEHC |
| Tyr-Leu | HMDB0029109 | Tyr-Leu |
| 2,6-Di-tert-butyl-4-(hydroxymethyl)phenol | HMDB0032048 | 2,6-Di-tert-butyl-4-(hydroxymethyl)phenol |
| Carnitine C8:1 | - | Carnitine C8:1 |
| FFA(15:1) | - | FFA(15:1) |
| Carnitine C8:0 | - | Carnitine C8:0 |
| Carnitine C8-OH | - | Carnitine C8-OH |
| Carnitine C9:1 | - | Carnitine C9:1 |
| Hexadecanedioic acid | HMDB0000672 | Hexadecanedioic acid |
| N-Myristoylglycine | HMDB0013250 | N-Myristoylglycine |
| Carnitine C9:0 | - | Carnitine C9:0 |
| N,N-Bis(2-hydroxyethyl)dodecanamide | HMDB0032358 | N,N-Bis(2-hydroxyethyl)dodecanamide |
| Riboflavin | HMDB0000244 | Riboflavin |
| Glu-Phe-Ala | - | Glu-Phe-Ala |
| Carnitine C9:1-OH | HMDB0015334 | Carnitine C9:1-OH |
| Carnitine C10:2 | - | Carnitine C10:2 |
| Carnitine C10:1 | - | Carnitine C10:1 |
| Carnitine C10:0 | - | Carnitine C10:0 |
| FFA(18:5) | - | FFA(18:5) |
| Phe-Ala-Leu | - | Phe-Ala-Leu |
| FFA(18:4) | - | FFA(18:4) |
| 9(S)-HpOTrE | - | 9(S)-HpOTrE |
| 13(R)-HODE | - | 13(R)-HODE |
| 9,10-DiHOME | HMDB0004704 | 9,10-DiHOME |
| 12,13-DiHOME | - | 12,13-DiHOME |
| Octadecanedioic acid | HMDB0000782 | Octadecanedioic acid |
| N-Palmitoylglycine | HMDB0013034 | N-Palmitoylglycine |
| Carnitine C11:0 | - | Carnitine C11:0 |
| 10-Hydroxystearic Acid | HMDB0037396 | 10-Hydroxystearic Acid |
| ST-638 | - | ST-638 |
| 2-Butyl-3-(4-hydroxybenzoyl)benzofuran | - | 2-Butyl-3-(4-hydroxybenzoyl)benzofuran |
| Androstenediol | HMDB0003818 | Androstenediol |
| Carnitine C12:0 | - | Carnitine C12:0 |
| Carnitine C12-OH | - | Carnitine C12-OH |
| LPA(0:0/16:0) | HMDB0007849 | LPA(0:0/16:0) |
| LPA(16:0/0:0) | HMDB0007853 | LPA(16:0/0:0) |
| LPE(14:0/0:0) | HMDB0011500 | LPE(14:0/0:0) |
| Phe-Trp | HMDB0029006 | Phe-Trp |
| Gly-Phe-Phe | - | Gly-Phe-Phe |
| Glutathione Oxidized | - | Glutathione Oxidized |
| (±)12-HETE | HMDB0006111 | (±)12-HETE |
| (±)5-HETE | HMDB0011134 | (±)5-HETE |
| TXB2 | HMDB0003252 | TXB2 |
| 20-Hydroxy Prostaglandin F2α | - | 20-Hydroxy Prostaglandin F2α |
| LPC(12:0/0:0) | - | LPC(12:0/0:0) |
| (E)-Guggulsterone | - | (E)-Guggulsterone |
| Corticosterone | HMDB0001547 | Corticosterone |
| Cortisol | HMDB0000063 | Cortisol |
| Carnitine C14:2-OH | - | Carnitine C14:2-OH |
| Carnitine C14:1-OH | - | Carnitine C14:1-OH |
| Carnitine C14:0 | - | Carnitine C14:0 |
| LPA(18:1/0:0) | HMDB0007855 | LPA(18:1/0:0) |
| LPE(0:0/16:1) | - | LPE(0:0/16:1) |
| Phosphatidylethanolamine lyso alkenyl 16:0 | - | Phosphatidylethanolamine lyso alkenyl 16:0 |
| LPE(16:0/0:0) | - | LPE(16:0/0:0) |
| LPE(0:0/16:0) | - | LPE(0:0/16:0) |
| Docodiendioicacid | - | Docodiendioicacid |
| LPC(0:0/14:0) | - | LPC(0:0/14:0) |
| Phosphatidylethanolamine lyso alkenyl 18:3 | - | Phosphatidylethanolamine lyso alkenyl 18:2 |
| LPE(0:0/18:2) | - | LPE(0:0/18:2) |
| LPE(18:1/0:0) | HMDB0011506 | LPE(18:1/0:0) |
| LPE(18:0/0:0) | HMDB0011130 | LPE(18:0/0:0) |
| LPE(0:0/18:0) | - | LPE(0:0/18:0) |
| LPC(0:0/15:0) | - | LPC(0:0/15:0) |
| Hyodeoxycholic acid | HMDB0000733 | Hyodeoxycholic acid |
| Chenodeoxycholic Acid | HMDB0000518 | Chenodeoxycholic Acid |
| Deoxycholic acid | HMDB0000626 | Deoxycholic acid |
| 3-Epideoxycholic acid | HMDB0000438 | 3-Epideoxycholic acid |
| LPC(O-16:1) | - | LPC(O-16:1/0:0) |
| LPC(0:0/16:0) | - | LPC(0:0/16:0) |
| LPC(O-16:0) | - | PC(O-16:0/0:0) |
| LPE(0:0/20:5) | - | LPE(0:0/20:5) |
| LPE(20:5/0:0) | - | LPE(20:5/0:0) |
| LPE(20:4/0:0) | - | LPE(20:4/0:0) |
| Glycerophospho-N-Arachidonoyl Ethanolamine | - | Glycerophospho-N-Arachidonoyl Ethanolamine |
| LPE(0:0/20:3) | - | LPE(0:0/20:3) |
| LPE(20:3/0:0) | - | LPE(20:3/0:0) |
| LPE(0:0/20:2) | - | LPE(0:0/20:2) |
| LPE(20:2/0:0) | - | LPE(20:2/0:0) |
| Glycodeoxycholic acid | HMDB0000631 | Glycine deoxycholic acid |
| Taurolithocholic acid | - | Taurolithocholic acid |
| LPC(0:0/18:3) | - | LPC(0:0/18:3) |
| LPC(18:3/0:0) | HMDB0010387 | LPC(18:3/0:0) |
| LPC(0:0/18:2) | - | LPC(0:0/18:2) |
| LPC(18:2/0:0) | HMDB0010386 | LPC(18:2/0:0) |
| LPC(18:1/0:0) | HMDB0010385 | LPC(18:1/0:0) |
| LPC(0:0/18:1) | - | LPC(0:0/18:1) |
| PC(16:0/2:0) | - | PC(16:0/2:0) |
| LPC(O-18:1) | - | LPC(O-18:1/0:0) |
| LPC(18:0/0:0) | HMDB0010384 | LPC(18:0/0:0) |
| LPC(0:0/18:0) | - | LPC(0:0/18:0) |
| LPE(0:0/22:6) | - | LPE(0:0/22:6) |
| LPE(22:6/0:0) | - | LPE(22:6/0:0) |
| 20,26-dihydroxyecdysone | - | 20,26-dihydroxyecdysone |
| LPE(0:0/22:5) | - | LPE(0:0/22:5) |
| LPE(22:5/0:0) | - | LPE(22:5/0:0) |
| LPC(0:0/20:4) | - | LPC(0:0/20:4) |
| LPC(0:0/20:3) | - | LPC(0:0/20:3) |
| LPC(20:3/0:0) | - | LPC(20:3/0:0) |
| LPC(0:0/20:2) | - | LPC(0:0/20:2) |
| Butenoyl-PAF | - | Butenoyl-PAF |
| LPC(0:0/20:1) | - | LPC(0:0/20:1) |
| PC(12:0/12:0) | - | PC(12:0/12:0) |
| Lythramine | - | Lythramine |
| Carnitine C22:2 | - | Carnitine C22:2 |
| L-Glycine | HMDB0000123 | L-Glycine |
| 2-Hydroxyethanesulfonate | HMDB0003903 | Isethionic acid |
| Taurine | HMDB0000251 | 2-Aminoethanesulfonic Acid |
| Methylguanidine | - | Methylguanidine |
| Cytochalasin H | - | Cytochalasin H |
| LPC(0:0/22:5) | - | LPC(0:0/22:5) |
| LPC(0:0/22:4) | - | LPC(0:0/22:4) |
| Chaps | - | Chaps |
| Urobilin | - | Urobilin |
| Malonic acid | HMDB0000691 | Malonicacid |
| Ammeline | - | Ammeline |
| Acrylamide | HMDB0004296 | Acrylamide |
| N-Formylglycine | - | N-Formylglycine |
| Aminomalonic Acid | HMDB0001147 | Aminomalonic Acid |
| L-Lactic Acid | HMDB0000190 | L-Lactic Acid |
| Sarcosine | HMDB0000271 | Sarcosine |
| O-Phospho-L-Serine | HMDB0000272 | O-Phospho-L-Serine |
| Hypotaurocyamine | - | Hypotaurocyamine |
| (R)-(-)-1-Amino-2-propanol | - | (R)-(-)-1-Amino-2-propanol |
| Creatine phosphate | HMDB0001511 | Creatine phosphate |
| Maleic Acid | HMDB0000176 | Maleic Acid |
| Creatinine | HMDB0000562 | Creatinine |
| Creatine | HMDB0000064 | Creatine |
| 3-Guanidinopropionic acid | HMDB0013222 | 3-Guanidinopropionic acid |
| Methylcysteine | HMDB0002108 | Methylcysteine |
| S-Methyl-L-Cysteine-S-oxide | - | S-Methyl-L-Cysteine-S-oxide |
| Hydroxypiperazic acid | - | Hydroxypiperazic acid |
| 3-Hydroxy-3-Methyl Butyric Acid | HMDB0000754 | 3-Hydroxy-3-Methyl Butyric Acid |
| 2-Hydroxy-2-Methyl Butyric acid | HMDB0001987 | 2-Hydroxy-2-Methyl Butyric acid |
| 2-Hydroxy-3-Methyl Butanoic Acid | - | 2-Hydroxy-3-Methyl Butanoic Acid |
| 3-Hydroxy-2-methylbutanoic acid | HMDB0000354 | 2-Methyl-3-hydroxybutyric acid |
| 4-Guanidinobutyric Acid | HMDB0003464 | 4-Guanidinobutyric Acid |
| Betaine | HMDB0000043 | Betaine |
| N-Methyl-α-aminoisobutyric acid | HMDB0002141 | N-Methyl-α-aminoisobutyric acid |
| L-Methionine | HMDB0000696 | L-Methionine |
| 2-Deoxyribose 1-Phosphate | HMDB0001351 | 2-Deoxyribose 1-Phosphate |
| D-Ornithine | - | D-Ornithine |
| L-Ornithine | HMDB0000214 | L-Ornithine |
| Agmatine | HMDB0001432 | Agmatine |
| Choline | HMDB0000097 | Choline |
| Pyrazine-2-carboxylic acid | HMDB0059734 | Pyrazine-2-carboxylic acid |
| Purine | HMDB0001366 | Purine |
| Allopurinol | HMDB0014581 | Allopurinol |
| Xanthine | HMDB0000292 | Xanthine |
| Oxypurinol | HMDB0000786 | Oxypurinol |
| Uric acid | HMDB0000289 | Uric acid |
| Adenine | HMDB0000034 | Adenine |
| L-Dihydroorotic Acid | HMDB0003349 | L-Dihydroorotic Acid |
| 4-Hydroxy-2-Oxoglutaric Acid | HMDB0002070 | 4-Hydroxy-2-Oxoglutaric Acid |
| 4-Aminomethylpyrimidine | - | 4-Aminomethylpyrimidine |
| 5-Methylcytosine | HMDB0002894 | 5-Methylcytosine |
| 1-pyrroline-4-hydroxy-2-carboxylate | HMDB0002234 | 1-pyrroline-4-hydroxy-2-carboxylate |
| 5,6-Dihydro-5-Methyluracil | HMDB0000079 | 5,6-Dihydro-5-Methyluracil |
| Ureidosuccinic acid | HMDB0000828 | Ureidosuccinic acid |
| 2-Methyl-1-Pyrroline | - | 2-Methyl-1-Pyrroline |
| Cyclocreatine | - | Cyclocreatine |
| N-Propionylglycine | HMDB0000783 | N-Propionylglycine |
| Cis-4-Hydroxy-L-Proline | HMDB0060460 | Cis-4-Hydroxy-D-Proline |
| O-Acetyl-L-serine | HMDB0003011 | O-Acetyl-L-serine |
| Ectoine | - | Ectoine |
| Ketoleucine | HMDB0000695 | 4-Methyl-2-oxovaleric acid |
| Adipic Acid | HMDB0000448 | Adipic Acid |
| 2-Methylglutaric Acid | HMDB0000422 | 2-Methylglutaric Acid |
| L-Gulonolactone | HMDB0003466 | L-Gulonolactone |
| N-acetylpyrrolidine | - | N-acetylpyrrolidine |
| Piperidine acid | HMDB0005960 | D-piperidine acid |
| Cycloleucine | HMDB0062225 | Cycloleucine |
| N-MethyTrans-4-Hydroxy-Proline | - | N-MethyTrans-4-Hydroxy-Proline |
| Ser-Ala | - | Ser-Ala |
| Ala-Ser | - | Ala-Ser |
| Gly-Thr | - | Gly-Thr |
| L-Cystine | HMDB0000192 | L-Cystine |
| 2-ethyl-2-hydroxybutyric acid | HMDB0001975 | 2-ethyl-2-hydroxybutyric acid |
| L-Fucose | HMDB0000174 | L-Fucose |
| D-Gluconic Acid | HMDB0000625 | D-Gluconic Acid |
| L-Citrulline | HMDB0000904 | L-Citrulline |
| D-Alloisoleucine | - | D-Allo-Isoleucine |
| Inositol 1-phosphate | HMDB00213 | Inositol 1-phosphate |
| L-Lysine | HMDB0000182 | L-Lysine |
| L-Arginine | HMDB0000517 | L-Arginine |
| Inositol 1,3,4-trisphosphate | HMDB0001143 | Inositol 1,3,4-trisphosphate |
| Triethyl-phosphate | HMDB0012228 | Triethyl-phosphate |
| Triethylenetetramine | - | Triethylenetetramine |
| 2,4-Dihydroxypteridine | - | 2,4-Dihydroxypteridine |
| Nicotinic Acid | HMDB0001488 | Nicotinic Acid |
| Isonicotinic acid | HMDB0060665 | Isonicotinic acid |
| 3-Chloroaniline | - | 3-Chloroaniline |
| Nicotinamide | HMDB0001406 | Nicotinamide |
| Urocanic Acid | HMDB0000301 | Urocanic Acid |
| Pyrocatechol | HMDB0000957 | Catechol |
| Dehydroascorbic acid | HMDB0001264 | Dehydroascorbic-acid |
| 4-Amino-5-hydroxymethyl-2-methylpyrimidine | HMDB0247327 | 4-Amino-5-hydroxymethyl-2-methylpyrimidine |
| 3-(pyrazol-1-yl)-L-alanine | - | 3-(pyrazol-1-yl)-L-alanine |
| cyclo(gly-glu) | - | cyclo(gly-glu) |
| 5-Acetylamino-6-amino-3-methyluracil | HMDB0004400 | 5-Acetylamino-6-amino-3-methyluracil |
| N-Acetylhistamine | HMDB0013253 | N-Acetylhistamine |
| 1-Methylhistidine | HMDB0000001 | 1-Methylhistidine |
| Oxaceprol | - | Oxaceprol |
| Gly-Pro | HMDB0000721 | Glyc-Pro |
| Proline betaine | HMDB0004827 | Stachydrine |
| Acetylvaline | HMDB0011757 | Acetylvaline |
| N-acetylornithine | HMDB0003357 | N-acetylornithine |
| Gly-Val | HMDB0028854 | Gly-Val |
| Val-Gly | HMDB0029127 | Val-Gly |
| L-Theanine | HMDB0034365 | L-Theanine |
| Acetylcholine | HMDB0000895 | Acetylcholine |
| 3-Carboxypropyltrimethylammonium | - | 3-Carboxypropyltrimethylammonium |
| DL-Carnitine | HMDB0000062 | DL-Carnitine |
| D-Sedoheptuiose 7-Phosphate | HMDB0001068 | D-Sedoheptuiose 7-Phosphate |
| L-Homoarginine | HMDB0000670 | L-Homoarginine |
| Spermidine | HMDB0001257 | Spermidine |
| Trigonelline | HMDB0000875 | Trigonelline |
| 6-Methylnicotinamide | HMDB0013704 | 6-Methylnicotinamide |
| 3-aminobenzamide | - | 3-aminobenzamide |
| 1,4-Dihydro-1-Methyl-4-Oxo-3-Pyridinecarboxamide | HMDB0004194 | 1,4-Dihydro-1-Methyl-4-Oxo-3-Pyridinecarboxamide |
| 3-Hydroxyphenylurea | - | 3-Hydroxyphenylurea |
| 4-Hydroxybenzyl alcohol | HMDB0011724 | 4-Hydroxybenzyl alcohol |
| 2-Amino-4-methylphenol | - | 2-Amino-4-methylphenol |
| Ethionamide | HMDB0014747 | Ethionamide |
| Caffeine | HMDB0001847 | Caffeine |
| N-Acetyl-L-Histidine | HMDB0032055 | N-Acetyl-L-Histidine |
| Cyclo(Ala-Pro) | - | Cyclo(Ala-Pro) |
| Barbital | HMDB0062202 | Barbital |
| Cys-Pro | - | Cys-Pro |
| Pro-Ser | - | Pro-Ser |
| Ser-Pro | - | Ser-Pro |
| γ-Glu-Cys | HMDB0001049 | γ-L-Glutamate-Cysteine |
| 5-Aminoimidazole ribonucleotide | HMDB0001235 | 5-Aminoimidazole ribonucleotide |
| Subericacid | HMDB0000893 | Subericacid |
| Hexanoyl Glycine | HMDB0000701 | Hexanoyl Glycine |
| N-Acetyl-L-Leucine | HMDB0011756 | N-Acetyl-L-Leucine |
| Val-Ala | - | Val-Ala |
| N6-Acetyl-L-Lysine | HMDB0000206 | N6-Acetyl-L-Lysine |
| Ser-Val | HMDB0029052 | Ser-Val |
| N-Acetylglucosamine 1-Phosphate | HMDB0001367 | N-Acetylglucosamine 1-Phosphate |
| 2-Hydroxycaprylic acid | HMDB0000711 | 2-Hydroxycaprylic acid |
| Lys-Gly | HMDB0028951 | Lys-Gly |
| Dl-2-Aminooctanoic Acid | HMDB0000991 | Dl-2-Aminooctanoic Acid |
| SDMA | HMDB0003334 | SDMA |
| N,N-Dimethylarginine | HMDB0001539 | N,N-Dimethylarginine |
| Tetraethylene-glycol | HMDB0094708 | Tetraethylene-glycol |
| Sn-Glycero-3-Phosphocholine | HMDB0000086 | Sn-Glycero-3-Phosphocholine |
| Terephthalic Acid | HMDB0002428 | Terephthalic Acid |
| Piperic acid | HMDB0032613 | Piperic acid |
| Indole | HMDB0000738 | Indole |
| Phenoxyacetic acid | HMDB0031609 | Phenoxyacetic acid |
| 3-Hydroxyphenylacetic acid | HMDB0000440 | 3-hydroxyphenylacetic acid |
| 4-acetoxyphenol | - | 4-acetoxyphenol |
| 5,6-Dimethylbenzimidazole | HMDB0003701 | 5,6-Dimethylbenzimidazole |
| (R)-(-)-2-Phenylpropionic Acid | - | (R)-(-)-2-Phenylpropionic Acid |
| M-toluene acetic acid | HMDB0002222 | M-toluene acetic acid |
| 3,4-Dimethylbenzoic acid | HMDB0002237 | 3,4-Dimethylbenzoic acid |
| 3-(2-Hydroxyphenyl)propanoic acid | HMDB0033752 | 3-(2-Hydroxyphenyl)propanoic acid |
| L-3-Phenyllactic acid | HMDB0000748 | (S)-2-Hydroxy-3-phenylpropanoic acid |
| (R)-2-Hydroxy-3-phenylpropionic acid | HMDB0000563 | (R)-2-Hydroxy-3-phenylpropionic-acid |
| Hydroxyphenyllactic acid | HMDB0000755 | Hydroxyphenyllactic acid |
| L-Phenylalanine | HMDB0000159 | L-Phenylalanine |
| Uridine | HMDB0000296 | Uridine |
| Β-Pseudouridine | HMDB0000767 | Β-Pseudouridine |
| 5-Hydroxy-2'-deoxyuridine | - | 5-Hydroxy-2'-deoxyuridine |
| 2-Phenylpropylamine | - | 2-Phenylpropylamine |
| Synephrine | HMDB0004826 | Synephrine |
| His-Ser | HMDB0028894 | His-Ser |
| Cytidine 5'-diphosphate | - | Cytidine 5'-diphosphate |
| Pro-Asn | - | Pro-Asn |
| Met-Asp | - | Met-Asp |
| Azelaic Acid | HMDB0000784 | Azelaic Acid |
| Thr-Gln | - | Thr-Gln |
| Carnitine C2:0 | HMDB0000201 | Carnitine C2:0 |
| Pantothenate | HMDB0000210 | Pantothenate |
| Leu-Ala | HMDB0028922 | Leu-Ala |
| Val-Thr | - | Val-Thr |
| Ser-Leu | HMDB0029043 | Ser-Leu |
| Ile-Ser | - | Ile-Ser |
| Lys-Ser | - | Lys-Ser |
| N6,N6,N6-Trimethyl-L-lysine | - | NE,NE,NE-TRIMETHYLLYSINE |
| N1-Acetylspermidine | - | N1-Acetylspermidine |
| Hydroxyquinoline | - | Hydroxyquinoline |
| Indole-3-carboxylic acid | HMDB0003320 | Indole-3-carboxylic acid |
| 3-Aminoquinoline | HMDB0036827 | 3-Aminoquinoline |
| 3-Hydroxycinnamic acid | HMDB0001713 | 3-Hydroxycinnamic acid |
| P-Coumaric Acid | HMDB0002035 | P-Coumaric Acid |
| 2-Hydroxycinnamic acid | HMDB0002641 | 2-Hydroxycinnamic acid |
| Caffeic Acid | HMDB0001964 | Caffeic Acid |
| Indole 3-carbinol | HMDB0005785 | Indole 3-carbinol |
| 4-Acetylaminobenzoic acid | - | 4-Acetylaminobenzoic acid |
| Methanesulfonic acid | - | Methanesulfonic acid |
| 4-Methylhippuric Acid | HMDB0013292 | 4-Methylhippuric Acid |
| Tryptamine | HMDB0000303 | Tryptamine |
| 4-Hydroxytryptamine | - | 4-Hydroxytryptamine |
| L-kynurenine | HMDB0000684 | L-kynurenine |
| Xanthosine | HMDB0000299 | Xanthosine |
| Propylparaben | HMDB0032574 | Propylparaben |
| 3-O-Methyldopa | - | 3-O-Methyldopa |
| Mycosporine-glycine | - | Mycosporine-glycine |
| Gln-Gln | - | Gln-Gln |
| Kynurenic Acid | HMDB0000715 | Kynurenic Acid |
| 3,4-Dimethoxycinnamic acid | HMDB0034315 | 3,4-Dimethoxycinnamic acid |
| N-Acetyl-L-Tyrosine | HMDB0000866 | N-Acetyl-L-Tyrosine |
| 4-tert-butylbenzoic acid | - | 4-tert-butylbenzoic-acid |
| 2-Methylguanosine | HMDB0005862 | 2-Methylguanosine |
| Glu-Leu | HMDB0028823 | Glu-Leu |
| Pantetheine | HMDB0003426 | Pantetheine |
| Lys-Val | HMDB0028964 | Lys-Val |
| Val-Arg | HMDB0029121 | Val-Arg |
| Tyr-Ser | - | Tyr-Ser |
| Ile-His | - | Ile-His |
| D-(+)-sucrose | HMDB0000258 | D-(+)-sucrose |
| FFA(12:0) | HMDB0000638 | FFA(12:0) |
| Asp-Phe | HMDB0000706 | Asp-Phe |
| S-(5-Adenosy)-L-Homocysteine | HMDB0000939 | S-(5-Adenosy)-L-Homocysteine |
| 1-Deoxyvaleric acid | - | 1-Deoxyvaleric-acid |
| FFA(16:1) | HMDB0003229 | FFA(16:1) |
| Trp-Ile | - | Trp-Ile |
| estrone 3-sulfate | HMDB0001425 | estrone 3-sulfate |
| FFA(18:2) | HMDB0000673 | FFA(18:2) |
| Carnitine C11:DC | - | Carnitine C11:DC |
| FAHFA(8:0/10:0) | - | FAHFA(8:0/10:0) |
| 9(S),12(S),13(S)-TriHOME | HMDB0004708 | 9(S),12(S),13(S)-TriHOME |
| Adrenosterone | HMDB0006772 | Adrenosterone |
| 6β-hydroxytestosterone | HMDB0006259 | 6β-hydroxytestosterone |
| Carnitine C12:1 | - | Carnitine C12:1 |
| AA | HMDB0001043 | AA |
| Carnitine C13:0 | HMDB0241308 | Carnitine C13:0 |
| Carnitine C14:3 | - | Carnitine C14:3 |
| Carnitine C14:1 | - | Carnitine C14:1 |
| Carnitine C14-OH | - | Carnitine C14-OH |
| LPA(0:0/18:0) | - | LPA(0:0/18:0) |
| FFA(22:4) | HMDB0002226 | FFA(22:4) |
| Carnitine C16:2 | - | Carnitine C16:2 |
| Carnitine C16:1 | - | Carnitine C16:1 |
| Carnitine C16-OH | HMDB0013336 | Carnitine C16-OH |
| Isochodeoxycholic acid | HMDB0000361 | Isochodeoxycholic acid |
| LPG(18:1) | HMDB0240602 | LPG(18:1/0:0) |
| Carnitine C18:3 | - | Carnitine C18:3 |
| Carnitine C18:2 | HMDB0006469 | Carnitine C18:2 |
| Carnitine C18:1-OH | - | Carnitine C18:1-OH |
| LPC(17:0/0:0) | HMDB0012108 | LPC(17:0/0:0) |
| Glycoursodeoxycholic Acid | HMDB0000708 | Glycoursodeoxycholic Acid |
| Glycohyodeoxycholic acid | - | Glycohyodeoxycholic acid |
| LPC(22:5/0:0) | - | LPC(22:5/0:0) |
| Endorphine-2-trifluoroacetate | HMDB0005774 | Endorphine-2-trifluoroacetate |
| Ammelide | - | Ammelide |
| Phosphoenolpyruvate | HMDB0000263 | Phosphoenolpyruvate |
| Guanidineacetic Acid | HMDB0000128 | Guanidineacetic Acid |
| L-Serine | HMDB0000187 | L-Serine |
| L-Isserine | - | L-Isserine |
| Uracil | HMDB0000300 | Uracil |
| 8-Azaguanine | - | 8-Azaguanine |
| L-Asparagine Anhydrous | HMDB0000168 | L-Asparagine Anhydrous |
| Methyl-L-alaninate | - | Methyl-L-alaninate |
| L-Glutamine | HMDB0000641 | L-Glutamine |
| L-lyxose | - | L-lyxose |
| 5-Aminovaleric Acid | HMDB0003355 | 5-Aminovaleric Acid |
| Hypoxanthine | HMDB0000157 | Hypoxanthine |
| 2-Aminomethylpyrimidine | - | 2-Aminomethylpyrimidine |
| N-Acetylcysteine | HMDB0001890 | N-Acetylcysteine |
| D-Glucoronic Acid | HMDB0000127 | D-Glucoronic Acid |
| D-Galacturonic Acid | HMDB0002545 | D-Galacturonic Acid |
| D-Fructose 6-Phosphate-Disodium Salt | - | D-Fructose 6-Phosphate-Disodium Salt |
| N-Acetylthreonine | HMDB0062557 | N-Acetylthreonine |
| D-Tagatose | HMDB0003418 | D-Tagatose |
| L-Rhamnonic acid | - | L-rhamnonic acid |
| L-Norleucine | HMDB0001645 | L-Norleucine |
| 7-Methyluric Acid | HMDB0011107 | 7-Methyluric Acid |
| 6-aminonicotinamide | - | 6-aminonicotinamide |
| Isocitric acid | HMDB0000193 | Isocitric acid |
| 3-Methylcrotonyl Glycine | HMDB0000459 | 3-Methylcrotonyl Glycine |
| Gln-Gly | HMDB0028797 | Gln-Gly |
| 1-Aminocyclohexanecarboxylic acid | - | 1-Aminocyclohexanoic acid |
| N-Acetyl-L-methionine | HMDB0011745 | N-Acetyl-L-methionine |
| Gly-His | HMDB0028843 | Gly-His |
| gamma-Glu-Ala | HMDB0006248 | (5-L-Glutamyl)-L-Amino Acid |
| Cork-oximate | - | Cork-oximate |
| Gly-Lys | - | Gly-Lys |
| 8-Aminooctanoic Acid | HMDB0247418 | 8-Aminooctanoic Acid |
| 4-Methoxysalicylic Acid | - | 4-Methoxysalicylic Acid |
| Acetanilide | HMDB0001250 | Acetanilide |
| 3-(3-Hydroxyphenyl)-3-hydroxypropanoic acid | HMDB0002643 | 3-(3-Hydroxyphenyl)-3-hydroxypropanoic acid |
| 3-Hydroxy-L-phenylalanine | - | 3-Hydroxy-L-phenylalanine |
| Pro-Asp | - | Pro-Asp |
| Cytidine-5-Monophosphate | HMDB0000095 | Cytidine-5-Monophosphate |
| Glu-Thr | HMDB0028829 | Glu-Thr |
| Ile-Ala | - | Ile-Ala |
| Ser-Lys | HMDB0029044 | Ser-Lys |
| 4-(Hydroxyamino)quinoline 1-oxide | - | 4-(Hydroxyamino)quinoline 1-oxide |

| **Table S4. Blood pressure associated metabolites including those taking antihypertensive medication in generalized linear models** | | | | | | |
| --- | --- | --- | --- | --- | --- | --- |
| Compound | Beta(95%CI) for SBP | P for SBP | FDR adjusted P for SBP | Beta(95%CI) for DBP | P for DBP | FDR adjusted P for DBP |
| Lythramine | 0.63(-0.36-1.61) | 2.11e-01 | 5.37e-01 | 0.90(0.34-1.46) | 1.73e-03 | 2.31e-02 |
| Bis(1-inositol)-3,1'-phosphate 1-phosphate | 1.70(0.71-2.69) | 7.45e-04 | 4.10e-02 | 1.09(0.53-1.66) | 1.58e-04 | 4.26e-03 |
| Perseitol | -1.26(-2.24--0.29) | 1.14e-02 | 1.68e-01 | -1.18(-1.74--0.62) | 3.49e-05 | 1.43e-03 |
| Cucurbitacin B | -1.05(-2.03--0.07) | 3.50e-02 | 2.62e-01 | -0.95(-1.51--0.39) | 8.56e-04 | 1.40e-02 |
| Butylate | -1.37(-2.34--0.40) | 5.74e-03 | 1.22e-01 | -0.30(-0.86-0.26) | 2.96e-01 | 5.96e-01 |
| Methyl reserpate | 2.19(1.06-3.31) | 1.41e-04 | 1.19e-02 | 1.17(0.52-1.81) | 3.91e-04 | 7.92e-03 |
| L-Glutamic Acid | 0.74(-0.35-1.84) | 1.81e-01 | 5.03e-01 | 1.29(0.66-1.91) | 5.30e-05 | 1.87e-03 |
| Asp-Phe | 0.29(-0.70-1.28) | 5.65e-01 | 8.26e-01 | 1.07(0.50-1.63) | 2.26e-04 | 5.58e-03 |
| N-lactoyl-phenylalanine | 1.90(0.85-2.96) | 4.23e-04 | 2.71e-02 | 1.36(0.76-1.97) | 1.03e-05 | 7.15e-04 |
| Nα-Acetyl-L-glutamine | 1.57(0.57-2.58) | 2.17e-03 | 7.73e-02 | 0.72(0.15-1.30) | 1.41e-02 | 9.97e-02 |
| N,N-Dimethylarginine | 1.53(0.49-2.56) | 3.79e-03 | 1.04e-01 | 0.42(-0.18-1.01) | 1.68e-01 | 4.37e-01 |
| 3-Hydroxyanthranilic Acid | 1.11(0.10-2.12) | 3.14e-02 | 2.46e-01 | 1.25(0.67-1.82) | 2.42e-05 | 1.08e-03 |
| 3-Amino-4-Hydroxybenzoic Acid | 1.11(0.10-2.12) | 3.14e-02 | 2.46e-01 | 1.25(0.67-1.82) | 2.42e-05 | 1.08e-03 |
| 4-acetoxyphenol | 0.61(-0.42-1.65) | 2.46e-01 | 5.70e-01 | 1.16(0.57-1.75) | 1.27e-04 | 3.62e-03 |
| CP 47,497-C8-homolog C-8-hydroxy metabolite | 1.34(0.36-2.32) | 7.16e-03 | 1.39e-01 | 0.96(0.40-1.52) | 8.37e-04 | 1.38e-02 |
| 6,6'-(1,2-phenylene)bis(1,3,5-triazine-2,4-diamine) | 1.14(0.16-2.12) | 2.32e-02 | 2.24e-01 | 0.51(-0.05-1.08) | 7.62e-02 | 2.83e-01 |
| Melibiose | -0.87(-1.84-0.11) | 8.05e-02 | 3.57e-01 | -0.68(-1.24--0.12) | 1.66e-02 | 1.12e-01 |
| N-acetyl-D-Lactosamine | 2.21(1.23-3.18) | 9.87e-06 | 3.16e-03 | 0.88(0.32-1.45) | 2.07e-03 | 2.50e-02 |
| Lactitol | 1.51(0.53-2.50) | 2.66e-03 | 8.04e-02 | 0.56(-0.01-1.13) | 5.41e-02 | 2.29e-01 |
| FFA(18:4) | 1.60(0.60-2.61) | 1.71e-03 | 6.86e-02 | 1.26(0.69-1.83) | 1.70e-05 | 9.13e-04 |
| FFA(17:1) | 0.49(-0.49-1.46) | 3.30e-01 | 6.44e-01 | 1.02(0.46-1.58) | 3.44e-04 | 7.44e-03 |
| FFA(16:2) | 1.96(0.97-2.96) | 1.09e-04 | 1.16e-02 | 1.33(0.76-1.90) | 4.85e-06 | 4.08e-04 |
| FFA(14:1) | 0.57(-0.43-1.58) | 2.63e-01 | 5.88e-01 | 0.99(0.41-1.56) | 7.53e-04 | 1.27e-02 |
| Carnitine C20:5 | 1.65(0.64-2.65) | 1.31e-03 | 6.18e-02 | 1.26(0.69-1.84) | 1.72e-05 | 9.13e-04 |
| Carnitine C16:1 | 1.19(0.19-2.19) | 1.93e-02 | 2.05e-01 | 0.69(0.12-1.26) | 1.81e-02 | 1.17e-01 |
| Carnitine C16-OH | 1.56(0.57-2.55) | 2.06e-03 | 7.63e-02 | 0.85(0.28-1.42) | 3.31e-03 | 3.54e-02 |
| Bovinic acid | 2.14(1.12-3.15) | 3.92e-05 | 6.97e-03 | 1.54(0.96-2.12) | 2.21e-07 | 7.07e-05 |
| 13-Tetradecynoic acid | 1.95(0.96-2.94) | 1.20e-04 | 1.16e-02 | 0.97(0.40-1.54) | 8.28e-04 | 1.38e-02 |
| 5,8,11-Eicosatrienoic acid | 1.05(0.07-2.02) | 3.64e-02 | 2.64e-01 | 0.91(0.35-1.47) | 1.50e-03 | 2.07e-02 |
| Ethyl palmitoleate | 1.62(0.62-2.61) | 1.48e-03 | 6.22e-02 | 1.09(0.51-1.66) | 2.00e-04 | 5.08e-03 |
| MG(0:0/22:6/0:0) | 0.87(-0.15-1.89) | 9.56e-02 | 3.78e-01 | 1.13(0.54-1.71) | 1.62e-04 | 4.26e-03 |
| MG(22:6/0:0/0:0) | 0.87(-0.15-1.89) | 9.56e-02 | 3.78e-01 | 1.13(0.54-1.71) | 1.62e-04 | 4.26e-03 |
| LPE(14:0/0:0) | 1.16(0.17-2.16) | 2.23e-02 | 2.20e-01 | 1.04(0.47-1.61) | 3.55e-04 | 7.48e-03 |
| LPA(0:0/18:0) | 1.09(0.12-2.07) | 2.76e-02 | 2.40e-01 | 1.16(0.60-1.71) | 4.57e-05 | 1.74e-03 |
| LPA(0:0/16:0) | 1.46(0.48-2.43) | 3.50e-03 | 1.00e-01 | 1.26(0.70-1.81) | 1.07e-05 | 7.17e-04 |
| LPE(0:0/16:1) | 0.71(-0.30-1.72) | 1.68e-01 | 4.95e-01 | 0.90(0.32-1.48) | 2.22e-03 | 2.64e-02 |
| LPA(16:0/0:0) | 0.73(-0.25-1.71) | 1.45e-01 | 4.57e-01 | 0.87(0.31-1.44) | 2.30e-03 | 2.70e-02 |
| LPG(16:0) | 1.10(0.12-2.08) | 2.75e-02 | 2.40e-01 | 0.98(0.42-1.54) | 6.27e-04 | 1.08e-02 |
| LPA(22:6) | 1.27(0.30-2.23) | 1.06e-02 | 1.67e-01 | 0.91(0.36-1.47) | 1.34e-03 | 1.95e-02 |
| LPC(0:0/14:0) | 1.67(0.67-2.67) | 1.07e-03 | 5.37e-02 | 1.48(0.91-2.05) | 4.23e-07 | 1.13e-04 |
| PC(12:0/12:0) | 0.86(-0.14-1.86) | 9.35e-02 | 3.77e-01 | 1.01(0.44-1.58) | 5.34e-04 | 9.83e-03 |
| LPC(22:5/0:0) | 1.10(0.12-2.08) | 2.79e-02 | 2.40e-01 | 0.99(0.43-1.56) | 5.29e-04 | 9.83e-03 |
| LPC(0:0/20:3) | 0.97(-0.03-1.97) | 5.66e-02 | 3.16e-01 | 1.08(0.51-1.65) | 2.12e-04 | 5.31e-03 |
| LPC(20:3/0:0) | 1.05(0.05-2.05) | 3.91e-02 | 2.72e-01 | 1.14(0.57-1.71) | 9.89e-05 | 2.88e-03 |
| LPC(18:0/0:0) | 1.20(0.23-2.18) | 1.58e-02 | 1.84e-01 | 1.26(0.70-1.82) | 9.87e-06 | 7.15e-04 |
| LPC(0:0/16:0) | 1.32(0.35-2.30) | 8.02e-03 | 1.47e-01 | 1.13(0.57-1.69) | 7.97e-05 | 2.50e-03 |
| LPC(12:0/0:0) | 0.93(-0.06-1.91) | 6.46e-02 | 3.31e-01 | 0.76(0.19-1.32) | 8.47e-03 | 6.85e-02 |
| LPC(0:0/18:0) | 1.10(0.12-2.08) | 2.79e-02 | 2.40e-01 | 1.15(0.59-1.71) | 5.87e-05 | 1.96e-03 |
| PC(O-1:0/O-16:0) | 1.25(0.27-2.23) | 1.26e-02 | 1.73e-01 | 1.22(0.66-1.78) | 2.07e-05 | 1.01e-03 |
| 2,3-Dihydroxypropyl 2-[(octadec-9-enoyl)amino]ethyl hydrogen phosphate | 0.54(-0.44-1.52) | 2.80e-01 | 6.03e-01 | 0.83(0.27-1.39) | 3.83e-03 | 3.91e-02 |
| Glycerophospho-N-Palmitoyl Ethanolamine | 1.34(0.35-2.34) | 8.02e-03 | 1.47e-01 | 1.25(0.68-1.82) | 1.71e-05 | 9.13e-04 |
| 1,2-Dihexanoyl-sn-glycero-3-phosphocholine | -1.55(-2.53--0.57) | 2.00e-03 | 7.63e-02 | -1.16(-1.72--0.60) | 5.49e-05 | 1.87e-03 |
| 1-Oleoyl lysophosphatidic acid sodium salt | 1.32(0.34-2.30) | 8.10e-03 | 1.47e-01 | 1.00(0.44-1.56) | 4.96e-04 | 9.45e-03 |
| Hydroxypiperazic acid | -1.15(-2.14--0.15) | 2.40e-02 | 2.29e-01 | -0.69(-1.26--0.12) | 1.79e-02 | 1.17e-01 |
| Imidazoleacetic acid | 2.30(1.31-3.30) | 5.76e-06 | 3.16e-03 | 1.06(0.49-1.63) | 2.92e-04 | 6.63e-03 |
| 4-Amino-5-hydroxymethyl-2-methylpyrimidine | 2.30(1.30-3.29) | 6.77e-06 | 3.16e-03 | 1.05(0.47-1.62) | 3.64e-04 | 7.48e-03 |
| (1R,2R,5R,8R,9S,10R,12S)-12-Hydroxy-11-methyl-6-methylidene-16-oxo-15-oxapentacyclo[9.3.2.15,8.01,10.02,8]heptadecane-9-carboxylic acid | 1.25(0.25-2.25) | 1.43e-02 | 1.84e-01 | 1.31(0.74-1.88) | 7.43e-06 | 5.66e-04 |
| Foetidin | 1.90(0.93-2.88) | 1.34e-04 | 1.19e-02 | 1.50(0.95-2.06) | 1.39e-07 | 6.13e-05 |
| Cortisol | 3.57(2.59-4.56) | 1.94e-12 | 3.10e-09 | 1.85(1.29-2.42) | 1.97e-10 | 3.15e-07 |
| 20,26-dihydroxyecdysone | 1.32(0.35-2.30) | 8.01e-03 | 1.47e-01 | 1.18(0.62-1.74) | 3.67e-05 | 1.47e-03 |
| 7-Ketocholesterol | -0.32(-1.29-0.65) | 5.20e-01 | 7.98e-01 | -0.76(-1.32--0.21) | 7.31e-03 | 6.23e-02 |
| Cortisone | 1.70(0.71-2.70) | 7.94e-04 | 4.10e-02 | 1.40(0.83-1.97) | 1.34e-06 | 1.65e-04 |
| 11-dehydro-TXB3 | 2.03(1.05-3.02) | 5.27e-05 | 8.05e-03 | 0.89(0.33-1.46) | 2.02e-03 | 2.50e-02 |
| 16-phenoxy tetranor Prostaglandin A2 | 2.22(1.01-3.43) | 3.26e-04 | 2.27e-02 | 1.02(0.33-1.72) | 3.97e-03 | 4.02e-02 |
| Prednisone | 1.37(0.37-2.37) | 7.04e-03 | 1.39e-01 | 1.31(0.74-1.88) | 6.51e-06 | 5.21e-04 |
| prostaglandin E3 | 1.72(0.72-2.72) | 7.93e-04 | 4.10e-02 | 1.15(0.58-1.73) | 8.54e-05 | 2.58e-03 |
| 11-Deoxyprostaglandin F1alpha | -0.83(-1.80-0.15) | 9.72e-02 | 3.80e-01 | -0.91(-1.47--0.35) | 1.42e-03 | 2.01e-02 |
| 1-Methylxanthine | 1.24(0.24-2.24) | 1.54e-02 | 1.84e-01 | 0.58(0.01-1.15) | 4.75e-02 | 2.12e-01 |
| Xanthine | 0.71(-0.33-1.74) | 1.80e-01 | 5.01e-01 | 1.19(0.60-1.78) | 8.44e-05 | 2.58e-03 |
| 3-Methylxanthine | 1.24(0.24-2.24) | 1.54e-02 | 1.84e-01 | 0.58(0.01-1.15) | 4.75e-02 | 2.12e-01 |
| 7-Methylxanthine | 1.24(0.24-2.24) | 1.54e-02 | 1.84e-01 | 0.58(0.01-1.15) | 4.75e-02 | 2.12e-01 |
| 2-(Dimethylamino)Guanosine | 1.14(0.04-2.23) | 4.15e-02 | 2.85e-01 | 0.82(0.20-1.45) | 1.02e-02 | 8.00e-02 |
| Creatine phosphate | 0.32(-0.67-1.31) | 5.27e-01 | 8.01e-01 | 0.90(0.34-1.47) | 1.78e-03 | 2.32e-02 |
| N6-(2-Hydroxyethyl)adenosine | 1.32(0.23-2.42) | 1.75e-02 | 1.98e-01 | 0.94(0.32-1.57) | 3.14e-03 | 3.40e-02 |
| 2-Hydroxyisocaproic Acid | 1.31(0.25-2.37) | 1.56e-02 | 1.84e-01 | 0.95(0.35-1.56) | 2.06e-03 | 2.50e-02 |
| L-Lactic Acid | 1.54(0.54-2.53) | 2.46e-03 | 7.82e-02 | 1.27(0.70-1.83) | 1.33e-05 | 8.18e-04 |
| 5-Hydroxyhexanoic Acid | 1.31(0.25-2.37) | 1.56e-02 | 1.84e-01 | 0.95(0.35-1.56) | 2.06e-03 | 2.50e-02 |
| Uric acid | 0.95(-0.09-1.99) | 7.44e-02 | 3.49e-01 | 0.90(0.31-1.50) | 3.08e-03 | 3.37e-02 |
| 2-Hydroxy-2-Methyl Butyric acid | 1.43(0.33-2.53) | 1.09e-02 | 1.67e-01 | 1.03(0.40-1.66) | 1.45e-03 | 2.03e-02 |
| (S)-Leucic acid | 1.31(0.25-2.37) | 1.56e-02 | 1.84e-01 | 0.95(0.35-1.56) | 2.06e-03 | 2.50e-02 |
| 2-Hydroxyhexanoic acid | 1.31(0.25-2.37) | 1.56e-02 | 1.84e-01 | 0.95(0.35-1.56) | 2.06e-03 | 2.50e-02 |
| 2-Hydroxy-3-Methyl Butanoic Acid | 1.39(0.28-2.49) | 1.37e-02 | 1.82e-01 | 1.01(0.38-1.64) | 1.78e-03 | 2.32e-02 |
| 2-Methoxyacetic acid | 1.09(0.11-2.07) | 2.85e-02 | 2.44e-01 | 0.94(0.39-1.50) | 9.33e-04 | 1.49e-02 |
| 3-Amino-5-hydroxybenzoic acid | 1.11(0.10-2.12) | 3.14e-02 | 2.46e-01 | 1.25(0.67-1.82) | 2.42e-05 | 1.08e-03 |
| 2-Methyllactic acid | 1.77(0.74-2.80) | 7.83e-04 | 4.10e-02 | 1.48(0.89-2.07) | 9.09e-07 | 1.32e-04 |
| (R)-2-Hydroxybutyric acid | 1.77(0.74-2.80) | 7.83e-04 | 4.10e-02 | 1.48(0.89-2.07) | 9.09e-07 | 1.32e-04 |
| Spermidic acid | 2.05(1.06-3.05) | 5.78e-05 | 8.05e-03 | 1.05(0.48-1.63) | 3.28e-04 | 7.18e-03 |
| Icosa-5,14-dienoic acid | 1.39(0.41-2.37) | 5.46e-03 | 1.22e-01 | 0.89(0.33-1.45) | 1.97e-03 | 2.50e-02 |
| Uridine triacetate | 1.27(0.28-2.26) | 1.21e-02 | 1.73e-01 | 1.17(0.60-1.74) | 5.41e-05 | 1.87e-03 |
| (Z)-2-tetracos-15-enamidoethanesulfonic acid | 1.31(0.33-2.29) | 8.77e-03 | 1.47e-01 | 1.14(0.58-1.70) | 6.93e-05 | 2.22e-03 |
| SPH(d18:1) | 0.64(-0.34-1.62) | 2.01e-01 | 5.28e-01 | 0.94(0.38-1.50) | 1.08e-03 | 1.67e-02 |

| **Table S5. Blood pressure associated metabolites stratified by sex in generalized linear models** | | | | | | | | | | | | |
| --- | --- | --- | --- | --- | --- | --- | --- | --- | --- | --- | --- | --- |
| Compounds | Beta(95%CI) for SBP in female | FDR adjusted P for SBP in female | Beta(95%CI) for DBP in female | FDR adjusted P for DBP in female | OR(95%CI) for Hypertension in female | FDR adjusted P for Hypertension in female | Beta(95%CI) for SBP in male | FDR adjusted P for SBP in male | Beta(95%CI) for DBP in male | FDR adjusted P for DBP in male | OR(95%CI) for Hypertension in male | FDR adjusted P for Hypertension in male |
| Lythramine | 0.51(-0.82-1.85) | 9.86e-01 | 0.81(0.04-1.58) | 4.97e-01 | 0.98(0.82-1.17) | 9.96e-01 | 1.64(-0.41-3.69) | 3.64e-01 | 1.39(0.21-2.57) | 1.32e-01 | 1.16(0.89-1.52) | 7.43e-01 |
| Bis(1-inositol)-3,1'-phosphate 1-phosphate | 1.64(0.35-2.94) | 5.24e-01 | 1.24(0.49-1.99) | 1.62e-01 | 1.16(0.98-1.38) | 8.81e-01 | 2.23(0.06-4.39) | 2.10e-01 | 1.49(0.24-2.74) | 1.28e-01 | 1.23(0.93-1.64) | 6.37e-01 |
| Perseitol | -1.18(-2.50-0.13) | 8.21e-01 | -0.89(-1.65--0.12) | 4.11e-01 | 0.88(0.74-1.05) | 9.28e-01 | -2.83(-5.01--0.65) | 1.15e-01 | -1.86(-3.12--0.61) | 5.02e-02 | 0.77(0.58-1.01) | 5.45e-01 |
| Cucurbitacin B | -1.27(-2.60-0.06) | 8.13e-01 | -1.03(-1.79--0.26) | 3.16e-01 | 0.96(0.81-1.15) | 9.96e-01 | -2.05(-4.29-0.20) | 2.85e-01 | -1.34(-2.64--0.04) | 1.94e-01 | 0.88(0.66-1.17) | 8.01e-01 |
| Butylate | -1.17(-2.48-0.14) | 8.32e-01 | -0.33(-1.09-0.43) | 8.95e-01 | 0.84(0.70-0.99) | 8.81e-01 | -3.30(-5.17--1.43) | 3.86e-02 | -1.25(-2.35--0.15) | 1.50e-01 | 0.84(0.65-1.07) | 6.43e-01 |
| Methyl reserpate | 2.48(0.99-3.96) | 1.30e-01 | 1.22(0.36-2.07) | 2.89e-01 | 1.32(1.08-1.61) | 7.25e-01 | 2.11(-0.61-4.82) | 3.76e-01 | 1.67(0.10-3.24) | 1.80e-01 | 1.06(0.76-1.49) | 9.43e-01 |
| L-Glutamic Acid | -0.49(-1.95-0.97) | 9.88e-01 | 0.38(-0.46-1.23) | 8.93e-01 | 0.93(0.77-1.14) | 9.92e-01 | 3.34(1.09-5.59) | 6.68e-02 | 2.57(1.28-3.86) | 9.51e-03 | 1.56(1.16-2.11) | 3.03e-01 |
| Asp-Phe | 0.31(-1.03-1.64) | 9.88e-01 | 0.92(0.15-1.69) | 3.81e-01 | 1.06(0.89-1.27) | 9.92e-01 | 1.02(-1.20-3.23) | 6.40e-01 | 1.30(0.03-2.58) | 2.02e-01 | 1.03(0.78-1.36) | 9.58e-01 |
| N-lactoyl-phenylalanine | 2.76(1.32-4.20) | 4.43e-02 | 1.78(0.95-2.61) | 1.91e-02 | 1.30(1.08-1.59) | 7.25e-01 | 3.63(1.30-5.95) | 5.43e-02 | 2.21(0.86-3.55) | 2.87e-02 | 1.72(1.26-2.38) | 1.41e-01 |
| Nα-Acetyl-L-glutamine | 2.31(1.02-3.60) | 7.93e-02 | 0.99(0.24-1.74) | 3.33e-01 | 1.22(1.02-1.45) | 8.81e-01 | 1.19(-1.41-3.79) | 6.40e-01 | 0.91(-0.60-2.41) | 4.76e-01 | 1.09(0.79-1.52) | 8.87e-01 |
| N,N-Dimethylarginine | 2.13(0.79-3.47) | 1.72e-01 | 0.63(-0.15-1.41) | 7.36e-01 | 1.27(1.06-1.52) | 7.25e-01 | 1.57(-0.81-3.94) | 4.73e-01 | 0.29(-1.09-1.67) | 8.30e-01 | 1.24(0.92-1.68) | 6.54e-01 |
| 3-Hydroxyanthranilic Acid | 0.15(-1.23-1.53) | 9.90e-01 | 0.49(-0.31-1.28) | 8.08e-01 | 1.04(0.87-1.25) | 9.96e-01 | 3.48(1.35-5.61) | 4.59e-02 | 2.31(1.09-3.54) | 1.19e-02 | 1.54(1.16-2.06) | 2.96e-01 |
| 3-Amino-4-Hydroxybenzoic Acid | 0.15(-1.23-1.53) | 9.90e-01 | 0.49(-0.31-1.28) | 8.08e-01 | 1.04(0.87-1.25) | 9.96e-01 | 3.48(1.35-5.61) | 4.59e-02 | 2.31(1.09-3.54) | 1.19e-02 | 1.54(1.16-2.06) | 2.96e-01 |
| 4-acetoxyphenol | 0.04(-1.32-1.41) | 9.90e-01 | 0.51(-0.28-1.31) | 8.05e-01 | 1.01(0.84-1.21) | 9.96e-01 | 2.52(0.27-4.77) | 1.71e-01 | 2.06(0.77-3.35) | 3.19e-02 | 1.36(1.02-1.82) | 5.09e-01 |
| CP 47,497-C8-homolog C-8-hydroxy metabolite | 0.78(-0.54-2.10) | 9.47e-01 | 0.65(-0.11-1.42) | 7.18e-01 | 1.11(0.93-1.32) | 9.61e-01 | 3.02(0.92-5.12) | 7.22e-02 | 1.71(0.49-2.92) | 6.42e-02 | 1.27(0.97-1.67) | 5.81e-01 |
| 6,6'-(1,2-phenylene)bis(1,3,5-triazine-2,4-diamine) | 1.11(-0.21-2.43) | 8.88e-01 | 0.15(-0.61-0.92) | 9.61e-01 | 1.22(1.02-1.46) | 8.81e-01 | 3.32(1.28-5.37) | 4.64e-02 | 2.01(0.83-3.20) | 2.39e-02 | 1.23(0.95-1.61) | 6.03e-01 |
| Melibiose | -1.28(-2.57-0.00) | 7.84e-01 | -0.94(-1.69--0.20) | 3.40e-01 | 0.88(0.74-1.04) | 9.28e-01 | -1.68(-3.81-0.44) | 3.66e-01 | -1.07(-2.30-0.16) | 2.90e-01 | 0.90(0.69-1.18) | 8.31e-01 |
| N-acetyl-D-Lactosamine | 2.40(1.11-3.70) | 5.68e-02 | 0.87(0.11-1.62) | 4.13e-01 | 1.33(1.12-1.59) | 6.33e-01 | 2.51(0.45-4.57) | 1.33e-01 | 1.17(-0.03-2.36) | 2.25e-01 | 1.35(1.03-1.78) | 4.68e-01 |
| Lactitol | 1.75(0.41-3.08) | 4.63e-01 | 0.61(-0.16-1.39) | 7.40e-01 | 1.18(0.99-1.40) | 8.81e-01 | 2.22(0.09-4.36) | 2.02e-01 | 1.19(-0.05-2.43) | 2.32e-01 | 1.20(0.91-1.58) | 6.90e-01 |
| FFA(18:4) | 1.48(0.10-2.86) | 7.44e-01 | 1.15(0.36-1.95) | 2.89e-01 | 1.10(0.92-1.32) | 9.88e-01 | 2.74(0.67-4.81) | 1.03e-01 | 1.85(0.66-3.04) | 3.86e-02 | 1.23(0.95-1.62) | 6.09e-01 |
| FFA(17:1) | 0.19(-1.13-1.51) | 9.90e-01 | 0.69(-0.07-1.46) | 6.57e-01 | 1.06(0.89-1.26) | 9.92e-01 | 1.54(-0.53-3.61) | 3.97e-01 | 1.56(0.37-2.74) | 8.50e-02 | 1.15(0.89-1.49) | 7.53e-01 |
| FFA(16:2) | 1.26(-0.04-2.56) | 8.02e-01 | 0.98(0.23-1.73) | 3.40e-01 | 1.07(0.90-1.27) | 9.92e-01 | 3.04(0.91-5.16) | 7.64e-02 | 1.94(0.71-3.17) | 3.34e-02 | 1.33(1.01-1.77) | 5.09e-01 |
| FFA(14:1) | -0.14(-1.50-1.22) | 9.90e-01 | 0.51(-0.28-1.30) | 8.05e-01 | 1.02(0.85-1.21) | 9.96e-01 | 3.10(1.00-5.21) | 6.77e-02 | 2.28(1.08-3.49) | 1.19e-02 | 1.30(1.00-1.72) | 5.40e-01 |
| Carnitine C20:5 | 1.49(-0.01-2.99) | 7.84e-01 | 1.10(0.23-1.97) | 3.40e-01 | 1.15(0.95-1.41) | 9.28e-01 | 2.39(0.44-4.35) | 1.33e-01 | 1.73(0.60-2.86) | 4.14e-02 | 1.21(0.94-1.55) | 6.34e-01 |
| Carnitine C16:1 | 0.60(-0.74-1.95) | 9.80e-01 | -0.11(-0.89-0.67) | 9.69e-01 | 1.10(0.92-1.31) | 9.88e-01 | 5.03(2.86-7.19) | 1.39e-02 | 2.76(1.50-4.02) | 4.90e-03 | 1.48(1.12-2.00) | 3.11e-01 |
| Carnitine C16-OH | 1.39(-0.01-2.80) | 7.84e-01 | 0.48(-0.33-1.30) | 8.11e-01 | 1.22(1.02-1.48) | 8.81e-01 | 3.13(1.12-5.15) | 5.50e-02 | 1.74(0.57-2.91) | 4.89e-02 | 1.58(1.22-2.09) | 1.44e-01 |
| Bovinic acid | 1.85(0.51-3.19) | 3.73e-01 | 0.99(0.21-1.76) | 3.40e-01 | 1.16(0.97-1.39) | 8.81e-01 | 3.57(1.41-5.73) | 4.44e-02 | 2.42(1.18-3.66) | 1.04e-02 | 1.45(1.09-1.95) | 3.31e-01 |
| 13-Tetradecynoic acid | 1.34(0.05-2.64) | 7.84e-01 | 0.49(-0.27-1.24) | 8.05e-01 | 1.15(0.97-1.37) | 8.81e-01 | 2.67(0.52-4.82) | 1.25e-01 | 1.37(0.13-2.62) | 1.63e-01 | 1.02(0.78-1.34) | 9.73e-01 |
| 5,8,11-Eicosatrienoic acid | 0.64(-0.67-1.95) | 9.75e-01 | 0.78(0.03-1.54) | 5.13e-01 | 1.03(0.87-1.22) | 9.96e-01 | 2.55(0.34-4.75) | 1.57e-01 | 1.36(0.08-2.64) | 1.80e-01 | 1.13(0.85-1.49) | 8.07e-01 |
| Ethyl palmitoleate | 0.95(-0.40-2.30) | 9.16e-01 | 0.44(-0.34-1.22) | 8.19e-01 | 1.13(0.95-1.36) | 9.28e-01 | 3.93(1.91-5.95) | 2.50e-02 | 2.25(1.08-3.42) | 1.12e-02 | 1.44(1.11-1.91) | 3.11e-01 |
| MG(0:0/22:6/0:0) | 0.49(-0.88-1.86) | 9.88e-01 | 0.70(-0.09-1.49) | 6.91e-01 | 1.07(0.90-1.29) | 9.92e-01 | 3.15(0.97-5.32) | 7.16e-02 | 2.47(1.22-3.71) | 9.51e-03 | 1.31(1.00-1.74) | 5.36e-01 |
| MG(22:6/0:0/0:0) | 0.49(-0.88-1.86) | 9.88e-01 | 0.70(-0.09-1.49) | 6.91e-01 | 1.07(0.90-1.29) | 9.92e-01 | 3.15(0.97-5.32) | 7.16e-02 | 2.47(1.22-3.71) | 9.51e-03 | 1.31(1.00-1.74) | 5.36e-01 |
| LPE(14:0/0:0) | 0.94(-0.37-2.25) | 9.16e-01 | 0.80(0.04-1.56) | 4.97e-01 | 1.04(0.87-1.23) | 9.96e-01 | 2.95(0.70-5.19) | 1.09e-01 | 2.16(0.87-3.45) | 2.58e-02 | 1.37(1.02-1.86) | 5.09e-01 |
| LPA(0:0/18:0) | 0.73(-0.58-2.05) | 9.47e-01 | 0.86(0.10-1.63) | 4.18e-01 | 1.04(0.88-1.24) | 9.96e-01 | 1.73(-0.35-3.82) | 3.38e-01 | 1.36(0.16-2.56) | 1.53e-01 | 1.34(1.02-1.76) | 5.09e-01 |
| LPA(0:0/16:0) | 1.53(0.25-2.82) | 6.12e-01 | 1.04(0.29-1.78) | 2.89e-01 | 1.17(0.98-1.39) | 8.81e-01 | 1.59(-0.54-3.72) | 3.97e-01 | 1.63(0.40-2.85) | 7.97e-02 | 1.03(0.79-1.35) | 9.58e-01 |
| LPE(0:0/16:1) | 0.29(-1.03-1.61) | 9.88e-01 | 0.59(-0.17-1.36) | 7.57e-01 | 0.98(0.83-1.17) | 9.96e-01 | 2.81(0.57-5.06) | 1.21e-01 | 1.88(0.59-3.18) | 5.46e-02 | 1.29(0.96-1.75) | 5.87e-01 |
| LPA(16:0/0:0) | 0.65(-0.64-1.93) | 9.66e-01 | 0.81(0.07-1.55) | 4.51e-01 | 1.07(0.90-1.28) | 9.92e-01 | 1.59(-0.60-3.78) | 4.15e-01 | 1.44(0.18-2.70) | 1.47e-01 | 1.11(0.85-1.47) | 8.27e-01 |
| LPG(16:0) | 1.48(0.16-2.80) | 6.86e-01 | 1.16(0.40-1.92) | 2.83e-01 | 1.09(0.91-1.30) | 9.92e-01 | 1.02(-1.10-3.14) | 6.24e-01 | 1.02(-0.21-2.24) | 3.20e-01 | 1.02(0.78-1.33) | 9.82e-01 |
| LPA(22:6) | 1.19(-0.11-2.48) | 8.16e-01 | 0.87(0.12-1.62) | 4.13e-01 | 1.09(0.92-1.30) | 9.92e-01 | 0.89(-1.24-3.01) | 6.75e-01 | 1.00(-0.23-2.22) | 3.30e-01 | 1.12(0.86-1.47) | 8.01e-01 |
| LPC(0:0/14:0) | 1.05(-0.30-2.40) | 9.16e-01 | 1.06(0.28-1.84) | 3.16e-01 | 1.06(0.88-1.26) | 9.92e-01 | 4.02(1.97-6.08) | 2.50e-02 | 2.91(1.74-4.08) | 1.11e-03 | 1.47(1.12-1.96) | 3.11e-01 |
| PC(12:0/12:0) | 0.74(-0.59-2.08) | 9.47e-01 | 0.80(0.03-1.57) | 5.13e-01 | 1.06(0.89-1.27) | 9.92e-01 | 2.04(-0.15-4.23) | 2.75e-01 | 1.19(-0.08-2.46) | 2.47e-01 | 1.14(0.87-1.52) | 7.91e-01 |
| LPC(22:5/0:0) | 0.40(-0.88-1.67) | 9.88e-01 | 0.54(-0.20-1.27) | 8.01e-01 | 1.01(0.85-1.19) | 9.99e-01 | 3.18(0.94-5.43) | 7.72e-02 | 2.21(0.91-3.50) | 2.39e-02 | 1.25(0.94-1.68) | 6.08e-01 |
| LPC(0:0/20:3) | 0.66(-0.66-1.97) | 9.66e-01 | 0.77(0.01-1.53) | 5.38e-01 | 1.09(0.91-1.29) | 9.92e-01 | 2.39(0.19-4.59) | 1.85e-01 | 1.34(0.07-2.62) | 1.84e-01 | 1.21(0.91-1.61) | 6.80e-01 |
| LPC(20:3/0:0) | 0.81(-0.52-2.14) | 9.47e-01 | 0.92(0.15-1.69) | 3.81e-01 | 1.10(0.93-1.32) | 9.88e-01 | 2.36(0.17-4.56) | 1.89e-01 | 1.24(-0.03-2.51) | 2.24e-01 | 1.19(0.90-1.58) | 7.06e-01 |
| LPC(18:0/0:0) | 0.70(-0.62-2.01) | 9.48e-01 | 0.87(0.11-1.63) | 4.13e-01 | 0.98(0.82-1.16) | 9.96e-01 | 2.54(0.46-4.62) | 1.33e-01 | 1.95(0.76-3.15) | 2.87e-02 | 1.23(0.94-1.60) | 6.09e-01 |
| LPC(0:0/16:0) | 1.17(-0.12-2.46) | 8.16e-01 | 0.82(0.07-1.57) | 4.51e-01 | 1.08(0.91-1.28) | 9.92e-01 | 3.16(0.97-5.35) | 7.16e-02 | 2.54(1.29-3.79) | 9.51e-03 | 1.19(0.90-1.58) | 7.02e-01 |
| LPC(12:0/0:0) | 0.84(-0.41-2.08) | 9.36e-01 | 0.67(-0.06-1.39) | 6.46e-01 | 1.10(0.93-1.29) | 9.88e-01 | 2.46(0.23-4.70) | 1.79e-01 | 1.83(0.54-3.12) | 6.06e-02 | 1.14(0.85-1.53) | 8.01e-01 |
| LPC(0:0/18:0) | 0.67(-0.64-1.97) | 9.57e-01 | 0.73(-0.03-1.48) | 5.91e-01 | 0.97(0.81-1.15) | 9.96e-01 | 2.54(0.43-4.66) | 1.37e-01 | 2.00(0.79-3.22) | 2.82e-02 | 1.25(0.96-1.64) | 5.96e-01 |
| PC(O-1:0/O-16:0) | 1.25(-0.04-2.54) | 8.01e-01 | 1.02(0.28-1.77) | 3.14e-01 | 1.07(0.90-1.27) | 9.92e-01 | 3.05(0.80-5.29) | 9.11e-02 | 2.41(1.12-3.69) | 1.28e-02 | 1.25(0.94-1.66) | 6.22e-01 |
| 2,3-Dihydroxypropyl 2-[(octadec-9-enoyl)amino]ethyl hydrogen phosphate | -0.02(-1.33-1.30) | 9.93e-01 | 0.37(-0.40-1.13) | 8.91e-01 | 0.98(0.83-1.17) | 9.96e-01 | 2.66(0.60-4.72) | 1.15e-01 | 2.08(0.90-3.26) | 1.96e-02 | 1.24(0.95-1.63) | 5.98e-01 |
| Glycerophospho-N-Palmitoyl Ethanolamine | 0.63(-0.70-1.96) | 9.75e-01 | 0.79(0.03-1.56) | 5.13e-01 | 1.04(0.87-1.24) | 9.96e-01 | 3.76(1.63-5.89) | 3.86e-02 | 2.67(1.45-3.89) | 4.90e-03 | 1.47(1.11-1.98) | 3.11e-01 |
| 1,2-Dihexanoyl-sn-glycero-3-phosphocholine | -2.48(-3.80--1.17) | 4.65e-02 | -1.39(-2.15--0.63) | 8.62e-02 | 0.87(0.73-1.04) | 9.11e-01 | -0.92(-3.13-1.29) | 6.75e-01 | -1.11(-2.39-0.16) | 2.90e-01 | 0.94(0.71-1.24) | 9.12e-01 |
| 1-Oleoyl lysophosphatidic acid sodium salt | 1.31(0.03-2.60) | 7.84e-01 | 0.94(0.19-1.68) | 3.40e-01 | 1.12(0.94-1.33) | 9.28e-01 | 1.89(-0.37-4.15) | 3.37e-01 | 1.93(0.64-3.23) | 4.89e-02 | 1.16(0.87-1.54) | 7.69e-01 |
| Hydroxypiperazic acid | -0.03(-1.42-1.35) | 9.93e-01 | -0.07(-0.88-0.73) | 9.84e-01 | 0.89(0.74-1.06) | 9.28e-01 | -3.07(-5.00--1.15) | 5.10e-02 | -2.26(-3.36--1.16) | 9.51e-03 | 0.53(0.39-0.69) | 2.09e-02 |
| Imidazoleacetic acid | 2.61(1.29-3.93) | 3.22e-02 | 1.34(0.57-2.11) | 1.18e-01 | 1.36(1.14-1.64) | 4.75e-01 | 2.11(-0.04-4.25) | 2.40e-01 | 1.42(0.18-2.66) | 1.45e-01 | 1.31(0.99-1.74) | 5.41e-01 |
| 4-Amino-5-hydroxymethyl-2-methylpyrimidine | 3.05(1.74-4.36) | 3.79e-03 | 1.32(0.55-2.09) | 1.20e-01 | 1.32(1.11-1.58) | 7.16e-01 | 1.93(-0.43-4.28) | 3.50e-01 | 1.21(-0.15-2.57) | 2.81e-01 | 1.38(1.01-1.88) | 5.09e-01 |
| (1R,2R,5R,8R,9S,10R,12S)-12-Hydroxy-11-methyl-6-methylidene-16-oxo-15-oxapentacyclo[9.3.2.15,8.01,10.02,8]heptadecane-9-carboxylic acid | 0.75(-0.54-2.04) | 9.47e-01 | 0.87(0.13-1.62) | 4.01e-01 | 0.98(0.83-1.16) | 9.96e-01 | 3.35(1.07-5.62) | 6.90e-02 | 2.48(1.17-3.78) | 1.19e-02 | 1.51(1.13-2.05) | 3.11e-01 |
| Foetidin | 1.32(-0.00-2.64) | 7.84e-01 | 0.87(0.10-1.63) | 4.18e-01 | 1.12(0.94-1.34) | 9.28e-01 | 3.51(1.55-5.48) | 3.86e-02 | 2.47(1.35-3.60) | 4.90e-03 | 1.24(0.97-1.61) | 5.91e-01 |
| Cortisol | 3.78(2.53-5.04) | 1.10e-05 | 1.80(1.07-2.53) | 3.27e-03 | 1.44(1.21-1.73) | 7.58e-02 | 3.53(1.29-5.77) | 5.33e-02 | 2.56(1.27-3.84) | 9.51e-03 | 1.40(1.04-1.89) | 4.48e-01 |
| 20,26-dihydroxyecdysone | 1.19(-0.11-2.50) | 8.16e-01 | 0.97(0.22-1.73) | 3.40e-01 | 1.07(0.90-1.27) | 9.92e-01 | 3.34(1.17-5.52) | 5.78e-02 | 2.50(1.25-3.74) | 9.51e-03 | 1.25(0.95-1.66) | 6.01e-01 |
| 7-Ketocholesterol | -0.20(-1.47-1.08) | 9.90e-01 | -0.88(-1.62--0.14) | 3.84e-01 | 1.07(0.91-1.27) | 9.92e-01 | -1.86(-4.05-0.33) | 3.32e-01 | -1.69(-2.95--0.43) | 7.78e-02 | 1.12(0.85-1.48) | 8.27e-01 |
| Cortisone | 1.77(0.49-3.06) | 3.73e-01 | 1.13(0.39-1.87) | 2.83e-01 | 1.14(0.96-1.35) | 9.13e-01 | 1.77(-0.49-4.02) | 3.73e-01 | 1.89(0.60-3.18) | 5.29e-02 | 1.08(0.81-1.44) | 8.91e-01 |
| 11-dehydro-TXB3 | 1.56(0.26-2.86) | 6.12e-01 | 0.36(-0.39-1.12) | 8.91e-01 | 1.14(0.96-1.36) | 9.13e-01 | 2.43(0.28-4.58) | 1.66e-01 | 1.35(0.10-2.59) | 1.70e-01 | 1.02(0.78-1.34) | 9.73e-01 |
| 16-phenoxy tetranor Prostaglandin A2 | 2.69(1.06-4.33) | 1.30e-01 | 1.28(0.34-2.23) | 3.16e-01 | 1.21(0.97-1.50) | 8.81e-01 | 3.39(0.44-6.34) | 1.59e-01 | 1.40(-0.32-3.11) | 3.30e-01 | 1.45(1.00-2.14) | 5.20e-01 |
| Prednisone | 0.95(-0.32-2.21) | 9.16e-01 | 0.99(0.26-1.72) | 3.16e-01 | 0.97(0.82-1.14) | 9.96e-01 | 3.14(0.83-5.45) | 9.11e-02 | 2.42(1.10-3.75) | 1.53e-02 | 1.49(1.10-2.03) | 3.12e-01 |
| prostaglandin E3 | 0.92(-0.47-2.31) | 9.38e-01 | 0.63(-0.17-1.43) | 7.52e-01 | 1.13(0.94-1.35) | 9.28e-01 | 3.88(1.81-5.95) | 3.01e-02 | 1.89(0.68-3.09) | 3.65e-02 | 1.39(1.06-1.84) | 4.16e-01 |
| 11-Deoxyprostaglandin F1alpha | -1.05(-2.37-0.26) | 8.93e-01 | -0.98(-1.73--0.22) | 3.40e-01 | 0.96(0.80-1.13) | 9.96e-01 | -1.11(-3.14-0.92) | 5.66e-01 | -1.04(-2.21-0.13) | 2.81e-01 | 0.87(0.68-1.12) | 7.49e-01 |
| 1-Methylxanthine | 0.94(-0.35-2.23) | 9.16e-01 | 0.48(-0.26-1.23) | 8.05e-01 | 1.18(0.99-1.40) | 8.81e-01 | 3.74(1.58-5.89) | 3.86e-02 | 1.99(0.74-3.24) | 3.29e-02 | 1.70(1.26-2.32) | 1.41e-01 |
| Xanthine | 0.19(-1.19-1.58) | 9.90e-01 | 0.56(-0.25-1.36) | 8.05e-01 | 1.04(0.87-1.24) | 9.96e-01 | 2.62(0.39-4.85) | 1.48e-01 | 2.04(0.77-3.32) | 3.19e-02 | 1.35(1.02-1.81) | 5.09e-01 |
| 3-Methylxanthine | 0.94(-0.35-2.23) | 9.16e-01 | 0.48(-0.26-1.23) | 8.05e-01 | 1.18(0.99-1.40) | 8.81e-01 | 3.74(1.58-5.89) | 3.86e-02 | 1.99(0.74-3.24) | 3.29e-02 | 1.70(1.26-2.32) | 1.41e-01 |
| 7-Methylxanthine | 0.94(-0.35-2.23) | 9.16e-01 | 0.48(-0.26-1.23) | 8.05e-01 | 1.18(0.99-1.40) | 8.81e-01 | 3.74(1.58-5.89) | 3.86e-02 | 1.99(0.74-3.24) | 3.29e-02 | 1.70(1.26-2.32) | 1.41e-01 |
| 2-(Dimethylamino)Guanosine | 0.31(-1.18-1.79) | 9.88e-01 | 0.25(-0.61-1.10) | 9.41e-01 | 1.26(1.03-1.53) | 8.81e-01 | 4.14(2.09-6.19) | 2.00e-02 | 2.17(0.98-3.37) | 1.65e-02 | 1.84(1.37-2.51) | 5.82e-02 |
| Creatine phosphate | 1.40(0.03-2.76) | 7.84e-01 | 1.34(0.55-2.13) | 1.29e-01 | 1.28(1.07-1.53) | 7.25e-01 | 1.29(-0.76-3.34) | 4.98e-01 | 1.16(-0.03-2.34) | 2.24e-01 | 1.15(0.89-1.50) | 7.48e-01 |
| N6-(2-Hydroxyethyl)adenosine | 0.63(-0.86-2.13) | 9.82e-01 | 0.39(-0.48-1.25) | 8.95e-01 | 1.31(1.07-1.60) | 7.25e-01 | 3.99(1.89-6.09) | 2.92e-02 | 2.20(0.98-3.42) | 1.70e-02 | 1.76(1.32-2.38) | 7.22e-02 |
| 2-Hydroxyisocaproic Acid | 1.09(-0.39-2.57) | 9.16e-01 | 0.64(-0.21-1.50) | 7.83e-01 | 1.14(0.93-1.38) | 9.28e-01 | 3.68(1.49-5.88) | 4.17e-02 | 1.68(0.40-2.96) | 8.50e-02 | 1.36(1.02-1.82) | 5.09e-01 |
| L-Lactic Acid | 1.92(0.61-3.24) | 2.59e-01 | 1.47(0.71-2.23) | 4.91e-02 | 1.16(0.97-1.39) | 8.81e-01 | 2.65(0.46-4.84) | 1.37e-01 | 1.78(0.52-3.05) | 6.40e-02 | 1.47(1.11-1.98) | 3.11e-01 |
| 5-Hydroxyhexanoic Acid | 1.09(-0.39-2.57) | 9.16e-01 | 0.64(-0.21-1.50) | 7.83e-01 | 1.14(0.93-1.38) | 9.28e-01 | 3.68(1.49-5.88) | 4.17e-02 | 1.68(0.40-2.96) | 8.50e-02 | 1.36(1.02-1.82) | 5.09e-01 |
| Uric acid | 0.48(-0.93-1.88) | 9.88e-01 | 0.42(-0.39-1.23) | 8.63e-01 | 1.03(0.86-1.24) | 9.96e-01 | 3.37(1.16-5.58) | 5.89e-02 | 2.43(1.17-3.70) | 1.12e-02 | 1.29(0.97-1.72) | 5.66e-01 |
| 2-Hydroxy-2-Methyl Butyric acid | 0.97(-0.55-2.50) | 9.47e-01 | 0.73(-0.15-1.61) | 7.26e-01 | 1.09(0.90-1.34) | 9.92e-01 | 4.57(2.31-6.82) | 2.00e-02 | 2.15(0.83-3.47) | 2.93e-02 | 1.54(1.14-2.11) | 3.11e-01 |
| (S)-Leucic acid | 1.09(-0.39-2.57) | 9.16e-01 | 0.64(-0.21-1.50) | 7.83e-01 | 1.14(0.93-1.38) | 9.28e-01 | 3.68(1.49-5.88) | 4.17e-02 | 1.68(0.40-2.96) | 8.50e-02 | 1.36(1.02-1.82) | 5.09e-01 |
| 2-Hydroxyhexanoic acid | 1.09(-0.39-2.57) | 9.16e-01 | 0.64(-0.21-1.50) | 7.83e-01 | 1.14(0.93-1.38) | 9.28e-01 | 3.68(1.49-5.88) | 4.17e-02 | 1.68(0.40-2.96) | 8.50e-02 | 1.36(1.02-1.82) | 5.09e-01 |
| 2-Hydroxy-3-Methyl Butanoic Acid | 0.94(-0.58-2.47) | 9.47e-01 | 0.71(-0.17-1.60) | 7.36e-01 | 1.09(0.89-1.33) | 9.92e-01 | 4.55(2.30-6.81) | 2.00e-02 | 2.16(0.84-3.48) | 2.87e-02 | 1.53(1.13-2.10) | 3.11e-01 |
| 2-Methoxyacetic acid | 1.10(-0.20-2.41) | 8.88e-01 | 0.73(-0.03-1.48) | 5.88e-01 | 1.14(0.96-1.36) | 9.13e-01 | 1.48(-0.62-3.59) | 4.35e-01 | 1.28(0.07-2.49) | 1.84e-01 | 1.27(0.97-1.68) | 5.65e-01 |
| 3-Amino-5-hydroxybenzoic acid | 0.15(-1.23-1.53) | 9.90e-01 | 0.49(-0.31-1.28) | 8.08e-01 | 1.04(0.87-1.25) | 9.96e-01 | 3.48(1.35-5.61) | 4.59e-02 | 2.31(1.09-3.54) | 1.19e-02 | 1.54(1.16-2.06) | 2.96e-01 |
| 2-Methyllactic acid | 1.28(-0.08-2.64) | 8.16e-01 | 1.11(0.33-1.89) | 2.89e-01 | 1.13(0.94-1.35) | 9.28e-01 | 3.13(0.88-5.37) | 8.39e-02 | 2.53(1.25-3.81) | 9.51e-03 | 1.46(1.10-1.97) | 3.12e-01 |
| (R)-2-Hydroxybutyric acid | 1.28(-0.08-2.64) | 8.16e-01 | 1.11(0.33-1.89) | 2.89e-01 | 1.13(0.94-1.35) | 9.28e-01 | 3.13(0.88-5.37) | 8.39e-02 | 2.53(1.25-3.81) | 9.51e-03 | 1.46(1.10-1.97) | 3.12e-01 |
| Spermidic acid | 2.72(1.41-4.02) | 1.74e-02 | 1.32(0.56-2.08) | 1.18e-01 | 1.30(1.09-1.55) | 7.16e-01 | 1.52(-0.91-3.95) | 4.99e-01 | 1.07(-0.34-2.47) | 3.64e-01 | 1.37(1.00-1.89) | 5.36e-01 |
| Icosa-5,14-dienoic acid | 1.11(-0.21-2.43) | 8.88e-01 | 0.78(0.02-1.54) | 5.27e-01 | 1.10(0.92-1.31) | 9.88e-01 | 2.70(0.54-4.86) | 1.21e-01 | 1.53(0.28-2.78) | 1.17e-01 | 1.25(0.95-1.66) | 5.97e-01 |
| Uridine triacetate | 0.87(-0.39-2.14) | 9.16e-01 | 0.86(0.13-1.59) | 3.91e-01 | 1.00(0.84-1.18) | 9.99e-01 | 2.82(0.48-5.16) | 1.37e-01 | 2.15(0.81-3.50) | 3.19e-02 | 1.44(1.06-1.97) | 4.16e-01 |
| (Z)-2-tetracos-15-enamidoethanesulfonic acid | 1.18(-0.12-2.47) | 8.16e-01 | 0.85(0.11-1.60) | 4.18e-01 | 1.09(0.92-1.30) | 9.88e-01 | 3.16(0.94-5.39) | 7.67e-02 | 2.49(1.22-3.76) | 1.03e-02 | 1.25(0.94-1.66) | 6.09e-01 |
| SPH(d18:1) | 1.09(-0.25-2.42) | 8.93e-01 | 1.05(0.28-1.83) | 3.16e-01 | 1.12(0.94-1.34) | 9.28e-01 | 1.25(-0.90-3.39) | 5.38e-01 | 1.41(0.18-2.64) | 1.47e-01 | 1.02(0.78-1.34) | 9.68e-01 |
| notes: FA, fatty acids; GP, glycerophospholipids; SL, sphingolipids; GL, glycerolipids. | | | | | | | | | | | | |

| **Table S6. Replicated Blood pressure-associated metabolites in Xiazhi** | | | | | |
| --- | --- | --- | --- | --- | --- |
| Metabolites | Trait | Cohort | Beta (95%CI) | *P* | FDR adjusted *P* |
| LPC(0:0/14:0) | SBP | Liuheng | 2.05(0.93-3.17) | 3.30E-04 | 2.11E-02 |
|  |  | Xiazhi | 1.71(0.69-2.74) | 1.01E-03 | 5.94E-02 |
|  | DBP | Liuheng | 1.71(1.07-2.36) | 2.11E-07 | 1.12E-04 |
|  |  | Xiazhi | 1.34(0.73-1.96) | 2.08E-05 | 1.23E-03 |
| LPC(0:0/16:0) | SBP | Liuheng | 1.71(0.61-2.82) | 2.40E-03 | 5.67E-02 |
|  |  | Xiazhi | 1.05(0.05-2.06) | 4.02E-02 | 3.51E-01 |
|  | DBP | Liuheng | 1.33(0.69-1.96) | 5.01E-05 | 2.97E-03 |
|  |  | Xiazhi | 0.74(0.13-1.35) | 1.69E-02 | 1.01E-01 |
| CI, confidence interval; SBP, systolic blood pressure; DBP, diastolic blood pressure | | | | | |

| **Table S7. Blood pressure change associated metabolite in generalized linear models*** | | | | | | | | | | |
| --- | --- | --- | --- | --- | --- | --- | --- | --- | --- | --- |
| Compound | Class I | Beta(95%CI) for SBP change | P for SBP Change | FDR adjusted P for SBP Change | Beta(95%CI) for DBP change | P for DBP Change | FDR adjusted P for DBP Change | OR(95%CI) for Incident Hypertension | P for Incident Hypertension | FDR adjusted P for Incident Hypertension |
| N-lactoyl-phenylalanine | Amino acid and Its metabolites | -0.04(-0.28-0.20) | 7.63e-01 | 9.72e-01 | 0.01(-0.25-0.27) | 9.21e-01 | 9.92e-01 | 1.24(1.01-1.53) | 4.07e-02 | 4.24e-01 |
| N-acetyl-D-Lactosamine | Carbohydrates and Its metabolites | 0.14(-0.08-0.37) | 2.13e-01 | 9.72e-01 | 0.31(0.07-0.55) | 1.19e-02 | 7.32e-01 | 1.09(0.90-1.33) | 3.76e-01 | 8.62e-01 |
| notes: *FDR adjusted P for metabolites and blood pressure < 0.05 & P for metabolites and changes of blood pressure < 0.05 | | | | | | | | | | |

| **Table S8. Summary of pathway analysis of blood pressure associated metabolites** | | | | | | | | | |
| --- | --- | --- | --- | --- | --- | --- | --- | --- | --- |
|  | Total | Expected | Hits | Raw p | minuslogP | Holm adjust | FDR | Impact | Metabolites |
| **Caffeine metabolism** | **10** | **0.10** | **2** | **0.00** | **2.44** | **0.29** | **0.29** | **0.31** | **1-Methylxanthine; 7-Methylxanthine** |
| Histidine metabolism | 16 | 0.15 | 2 | 0.01 | 2.03 | 0.74 | 0.38 | 0.00 |  |
| Arginine and proline metabolism | 36 | 0.34 | 2 | 0.04 | 1.35 | 1.00 | 0.89 | 0.00 |  |
| **Glycerophospholipid metabolism** | **36** | **0.34** | **2** | **0.04** | **1.35** | **1.00** | **0.89** | **0.16** | **LPA(0:0/16:0); LPC(18:0/0:0)** |
| Nitrogen metabolism | 6 | 0.06 | 1 | 0.06 | 1.25 | 1.00 | 0.89 | 0.00 |  |
| Arginine biosynthesis | 14 | 0.13 | 1 | 0.13 | 0.90 | 1.00 | 1.00 | 0.12 |  |
| Butanoate metabolism | 15 | 0.14 | 1 | 0.13 | 0.87 | 1.00 | 1.00 | 0.00 |  |
| Purine metabolism | 70 | 0.67 | 2 | 0.14 | 0.85 | 1.00 | 1.00 | 0.01 |  |
| Glycerolipid metabolism | 16 | 0.15 | 1 | 0.14 | 0.85 | 1.00 | 1.00 | 0.01 |  |
| Pyruvate metabolism | 23 | 0.22 | 1 | 0.20 | 0.70 | 1.00 | 1.00 | 0.00 |  |
| Steroid hormone biosynthesis | 87 | 0.83 | 2 | 0.20 | 0.70 | 1.00 | 1.00 | 0.04 |  |
| Glycolysis / Gluconeogenesis | 26 | 0.25 | 1 | 0.22 | 0.65 | 1.00 | 1.00 | 0.00 |  |
| Galactose metabolism | 27 | 0.26 | 1 | 0.23 | 0.64 | 1.00 | 1.00 | 0.06 |  |
| Glutathione metabolism | 28 | 0.27 | 1 | 0.24 | 0.63 | 1.00 | 1.00 | 0.02 |  |
| Alanine, aspartate and glutamate metabolism | 28 | 0.27 | 1 | 0.24 | 0.63 | 1.00 | 1.00 | 0.20 |  |
| Porphyrin metabolism | 31 | 0.30 | 1 | 0.26 | 0.59 | 1.00 | 1.00 | 0.00 |  |
| Glyoxylate and dicarboxylate metabolism | 32 | 0.30 | 1 | 0.27 | 0.58 | 1.00 | 1.00 | 0.00 |  |
| Sphingolipid metabolism | 32 | 0.30 | 1 | 0.27 | 0.58 | 1.00 | 1.00 | 0.06 |  |
| Tryptophan metabolism | 41 | 0.39 | 1 | 0.33 | 0.48 | 1.00 | 1.00 | 0.05 |  |

| **Table S9. F-statistics for blood pressure-associated metabolites and ratios in Liuheng** | | |
| --- | --- | --- |
| Metabolites/Ratios | Compound | F statistics |
| Metabolites | Asp-Phe | 22.68 |
| Metabolites | L-Glutamic Acid | 21.67 |
| Metabolites | Nα-Acetyl-L-glutamine | 22.01 |
| Metabolites | N-acetyl-D-Lactosamine | 20.90 |
| Metabolites | 13-Tetradecynoic acid | 25.80 |
| Metabolites | Carnitine C16:1 | 22.86 |
| Metabolites | Cortisol | 23.70 |
| Metabolites | prostaglandin E3 | 26.02 |
| Metabolites | Creatine phosphate | 24.93 |
| Metabolites | SPH(d18:1) | 22.70 |
| Ratios | Cortisol / Cortisone | 22.38 |
| Ratios | Creatine / Phosphocreatine | 22.43 |

| **Table S10. Replicated potential causal relationships of metabolites with blood pressure in THSBC** | | | | | | |
| --- | --- | --- | --- | --- | --- | --- |
| Metabolites | Metabolite GWAS | Blood pressure GWAS | Trait | Nsnp | Beta(95%CI) | *P* |
| Asp-Phe | Liuheng | BBJ | DBP | 11 | 0.023(0.011-0.036) | 3.09E-04 |
|  | THSBC | BBJ | DBP | 5 | 0.020(0.005-0.034) | 7.85E-03 |
|  | THSBC | KoGES | Hypertension | 5 | 0.065(0.006-0.123) | 2.94E-02 |
| N-acetyl-D-Lactosamine | Liuheng | KoGES | DBP | 10 | 0.026(0.010-0.042) | 1.16E-03 |
|  | Liuheng | TWB | DBP | 11 | 0.018(0.004-0.031) | 8.85E-03 |
|  | THSBC | BBJ | Hypertension | 6 | 0.069(0.003-0.135) | 4.13E-02 |
| Creatine phosphate | Liuheng | BBJ | DBP | 6 | 0.016(0.003-0.029) | 1.59E-02 |
|  | THSBC | BBJ | Hypertension | 4 | 0.089(0.002-0.176) | 4.46E-02 |
| notes: THSBC, the Tongji-Huaxi-Shuangliu Birth Cohort; SBP, systolic blood pressure; DBP, diastolic blood pressure; CI, confidential interval; GWAS, genome-wide association study; BBJ, the BioBank Japan; KoGES, the Korean Genome and Epidemiology Study; TWB, the Taiwan Biobank. | | | | | | |

| **Table S11. Potential causal relationships of blood pressure with metabolites in IVW analysis in Liuheng** | | | | |
| --- | --- | --- | --- | --- |
| Compound | Nsnp | Beta(95%CI) | P | Label |
| Asp-Phe | 36 | 0.001 ( -0.555 - 0.558 ) | 9.97e-01 | SBP-Metabolite(BBJ) |
| Asp-Phe | 21 | 0.163 ( -0.303 - 0.628 ) | 4.94e-01 | SBP-Metabolite(KoGES) |
| Asp-Phe | 40 | 0.213 ( -0.264 - 0.691 ) | 3.81e-01 | SBP-Metabolite(TWB) |
| Asp-Phe | 21 | 0.003 ( -0.685 - 0.690 ) | 9.93e-01 | DBP-Metabolite(BBJ) |
| Asp-Phe | 21 | 0.421 ( -0.068 - 0.910 ) | 9.15e-02 | DBP-Metabolite(KoGES) |
| Asp-Phe | 36 | 0.076 ( -0.366 - 0.518 ) | 7.35e-01 | DBP-Metabolite(TWB) |
| Asp-Phe | 17 | -0.011 ( -0.210 - 0.188 ) | 9.12e-01 | Hypertension-Metabolite(BBJ) |
| Asp-Phe | 24 | 0.162 ( -0.032 - 0.355 ) | 1.01e-01 | Hypertension-Metabolite(KoGES) |
| L-Glutamic Acid | 36 | -0.004 ( -0.644 - 0.636 ) | 9.91e-01 | SBP-Metabolite(BBJ) |
| L-Glutamic Acid | 21 | -0.154 ( -0.782 - 0.473 ) | 6.30e-01 | SBP-Metabolite(KoGES) |
| L-Glutamic Acid | 40 | -0.327 ( -0.784 - 0.131 ) | 1.62e-01 | SBP-Metabolite(TWB) |
| L-Glutamic Acid | 21 | 0.347 ( -0.459 - 1.153 ) | 3.99e-01 | DBP-Metabolite(BBJ) |
| L-Glutamic Acid | 21 | 0.253 ( -0.415 - 0.922 ) | 4.58e-01 | DBP-Metabolite(KoGES) |
| L-Glutamic Acid | 36 | -0.147 ( -0.621 - 0.327 ) | 5.44e-01 | DBP-Metabolite(TWB) |
| L-Glutamic Acid | 17 | -0.037 ( -0.275 - 0.201 ) | 7.61e-01 | Hypertension-Metabolite(BBJ) |
| L-Glutamic Acid | 24 | 0.060 ( -0.142 - 0.263 ) | 5.59e-01 | Hypertension-Metabolite(KoGES) |
| Nα-Acetyl-L-glutamine | 36 | 0.100 ( -0.629 - 0.828 ) | 7.89e-01 | SBP-Metabolite(BBJ) |
| Nα-Acetyl-L-glutamine | 21 | 0.391 ( -0.140 - 0.921 ) | 1.49e-01 | SBP-Metabolite(KoGES) |
| Nα-Acetyl-L-glutamine | 40 | -0.252 ( -0.684 - 0.180 ) | 2.53e-01 | SBP-Metabolite(TWB) |
| Nα-Acetyl-L-glutamine | 21 | 0.755 ( -0.176 - 1.686 ) | 1.12e-01 | DBP-Metabolite(BBJ) |
| Nα-Acetyl-L-glutamine | 21 | 0.558 ( 0.053 - 1.064 ) | 3.02e-02 | DBP-Metabolite(KoGES) |
| Nα-Acetyl-L-glutamine | 36 | -0.086 ( -0.523 - 0.351 ) | 6.99e-01 | DBP-Metabolite(TWB) |
| Nα-Acetyl-L-glutamine | 17 | 0.142 ( -0.073 - 0.358 ) | 1.95e-01 | Hypertension-Metabolite(BBJ) |
| Nα-Acetyl-L-glutamine | 24 | 0.081 ( -0.142 - 0.304 ) | 4.76e-01 | Hypertension-Metabolite(KoGES) |
| N-acetyl-D-Lactosamine | 36 | 0.544 ( -0.056 - 1.145 ) | 7.56e-02 | SBP-Metabolite(BBJ) |
| N-acetyl-D-Lactosamine | 21 | 0.633 ( 0.163 - 1.102 ) | 8.31e-03 | SBP-Metabolite(KoGES) |
| N-acetyl-D-Lactosamine | 40 | 0.315 ( -0.126 - 0.756 ) | 1.61e-01 | SBP-Metabolite(TWB) |
| N-acetyl-D-Lactosamine | 21 | 0.802 ( 0.004 - 1.601 ) | 4.88e-02 | DBP-Metabolite(BBJ) |
| N-acetyl-D-Lactosamine | 21 | 0.733 ( 0.240 - 1.227 ) | 3.59e-03 | DBP-Metabolite(KoGES) |
| N-acetyl-D-Lactosamine | 36 | 0.254 ( -0.197 - 0.704 ) | 2.70e-01 | DBP-Metabolite(TWB) |
| N-acetyl-D-Lactosamine | 17 | 0.313 ( 0.112 - 0.514 ) | 2.29e-03 | Hypertension-Metabolite(BBJ) |
| N-acetyl-D-Lactosamine | 24 | 0.138 ( -0.027 - 0.302 ) | 1.01e-01 | Hypertension-Metabolite(KoGES) |
| 13-Tetradecynoic acid | 36 | -0.671 ( -1.220 - -0.122 ) | 1.65e-02 | SBP-Metabolite(BBJ) |
| 13-Tetradecynoic acid | 21 | -0.052 ( -0.596 - 0.492 ) | 8.51e-01 | SBP-Metabolite(KoGES) |
| 13-Tetradecynoic acid | 40 | 0.032 ( -0.400 - 0.464 ) | 8.84e-01 | SBP-Metabolite(TWB) |
| 13-Tetradecynoic acid | 21 | -1.178 ( -1.856 - -0.500 ) | 6.57e-04 | DBP-Metabolite(BBJ) |
| 13-Tetradecynoic acid | 21 | 0.071 ( -0.512 - 0.653 ) | 8.12e-01 | DBP-Metabolite(KoGES) |
| 13-Tetradecynoic acid | 36 | -0.228 ( -0.719 - 0.262 ) | 3.61e-01 | DBP-Metabolite(TWB) |
| 13-Tetradecynoic acid | 17 | -0.120 ( -0.317 - 0.077 ) | 2.32e-01 | Hypertension-Metabolite(BBJ) |
| 13-Tetradecynoic acid | 24 | -0.220 ( -0.381 - -0.059 ) | 7.48e-03 | Hypertension-Metabolite(KoGES) |
| Carnitine C16:1 | 36 | 0.563 ( -0.037 - 1.163 ) | 6.57e-02 | SBP-Metabolite(BBJ) |
| Carnitine C16:1 | 21 | 0.468 ( -0.039 - 0.975 ) | 7.06e-02 | SBP-Metabolite(KoGES) |
| Carnitine C16:1 | 40 | 0.215 ( -0.280 - 0.711 ) | 3.95e-01 | SBP-Metabolite(TWB) |
| Carnitine C16:1 | 21 | 0.147 ( -0.541 - 0.834 ) | 6.76e-01 | DBP-Metabolite(BBJ) |
| Carnitine C16:1 | 21 | 0.085 ( -0.409 - 0.579 ) | 7.36e-01 | DBP-Metabolite(KoGES) |
| Carnitine C16:1 | 36 | 0.025 ( -0.440 - 0.490 ) | 9.16e-01 | DBP-Metabolite(TWB) |
| Carnitine C16:1 | 17 | -0.030 ( -0.231 - 0.172 ) | 7.74e-01 | Hypertension-Metabolite(BBJ) |
| Carnitine C16:1 | 24 | 0.031 ( -0.132 - 0.195 ) | 7.07e-01 | Hypertension-Metabolite(KoGES) |
| Cortisol | 36 | 0.297 ( -0.284 - 0.879 ) | 3.16e-01 | SBP-Metabolite(BBJ) |
| Cortisol | 21 | 0.305 ( -0.160 - 0.770 ) | 1.98e-01 | SBP-Metabolite(KoGES) |
| Cortisol | 40 | 0.490 ( 0.007 - 0.972 ) | 4.68e-02 | SBP-Metabolite(TWB) |
| Cortisol | 21 | 0.270 ( -0.437 - 0.977 ) | 4.54e-01 | DBP-Metabolite(BBJ) |
| Cortisol | 21 | 0.520 ( 0.031 - 1.008 ) | 3.71e-02 | DBP-Metabolite(KoGES) |
| Cortisol | 36 | 0.468 ( -0.029 - 0.964 ) | 6.49e-02 | DBP-Metabolite(TWB) |
| Cortisol | 17 | -0.006 ( -0.205 - 0.193 ) | 9.52e-01 | Hypertension-Metabolite(BBJ) |
| Cortisol | 24 | 0.133 ( -0.062 - 0.329 ) | 1.81e-01 | Hypertension-Metabolite(KoGES) |
| prostaglandin E3 | 36 | -0.199 ( -0.741 - 0.344 ) | 4.72e-01 | SBP-Metabolite(BBJ) |
| prostaglandin E3 | 21 | -0.103 ( -0.557 - 0.351 ) | 6.57e-01 | SBP-Metabolite(KoGES) |
| prostaglandin E3 | 40 | -0.131 ( -0.557 - 0.295 ) | 5.48e-01 | SBP-Metabolite(TWB) |
| prostaglandin E3 | 21 | -0.171 ( -0.841 - 0.499 ) | 6.18e-01 | DBP-Metabolite(BBJ) |
| prostaglandin E3 | 21 | 0.005 ( -0.471 - 0.482 ) | 9.82e-01 | DBP-Metabolite(KoGES) |
| prostaglandin E3 | 36 | -0.039 ( -0.471 - 0.392 ) | 8.58e-01 | DBP-Metabolite(TWB) |
| prostaglandin E3 | 17 | -0.169 ( -0.380 - 0.042 ) | 1.17e-01 | Hypertension-Metabolite(BBJ) |
| prostaglandin E3 | 24 | -0.093 ( -0.252 - 0.066 ) | 2.50e-01 | Hypertension-Metabolite(KoGES) |
| Creatine phosphate | 36 | 0.213 ( -0.424 - 0.851 ) | 5.12e-01 | SBP-Metabolite(BBJ) |
| Creatine phosphate | 21 | 0.393 ( -0.075 - 0.861 ) | 1.00e-01 | SBP-Metabolite(KoGES) |
| Creatine phosphate | 40 | 0.135 ( -0.305 - 0.574 ) | 5.48e-01 | SBP-Metabolite(TWB) |
| Creatine phosphate | 21 | 0.629 ( -0.062 - 1.320 ) | 7.43e-02 | DBP-Metabolite(BBJ) |
| Creatine phosphate | 21 | 0.242 ( -0.250 - 0.734 ) | 3.35e-01 | DBP-Metabolite(KoGES) |
| Creatine phosphate | 36 | 0.133 ( -0.312 - 0.578 ) | 5.57e-01 | DBP-Metabolite(TWB) |
| Creatine phosphate | 17 | 0.058 ( -0.142 - 0.259 ) | 5.69e-01 | Hypertension-Metabolite(BBJ) |
| Creatine phosphate | 24 | 0.034 ( -0.136 - 0.203 ) | 6.97e-01 | Hypertension-Metabolite(KoGES) |
| SPH(d18:1) | 36 | -0.182 ( -0.741 - 0.378 ) | 5.25e-01 | SBP-Metabolite(BBJ) |
| SPH(d18:1) | 21 | -0.121 ( -0.590 - 0.347 ) | 6.12e-01 | SBP-Metabolite(KoGES) |
| SPH(d18:1) | 40 | -0.004 ( -0.444 - 0.436 ) | 9.87e-01 | SBP-Metabolite(TWB) |
| SPH(d18:1) | 21 | -0.157 ( -0.883 - 0.569 ) | 6.72e-01 | DBP-Metabolite(BBJ) |
| SPH(d18:1) | 21 | 0.080 ( -0.412 - 0.572 ) | 7.51e-01 | DBP-Metabolite(KoGES) |
| SPH(d18:1) | 36 | -0.023 ( -0.468 - 0.423 ) | 9.20e-01 | DBP-Metabolite(TWB) |
| SPH(d18:1) | 17 | -0.119 ( -0.320 - 0.082 ) | 2.44e-01 | Hypertension-Metabolite(BBJ) |
| SPH(d18:1) | 24 | -0.055 ( -0.219 - 0.110 ) | 5.14e-01 | Hypertension-Metabolite(KoGES) |

| **Table S12. Potential causal relationships of metabolites with blood pressure in MR-RAP and Egger analysis in Liuheng** | | | | | | |
| --- | --- | --- | --- | --- | --- | --- |
| Compound | Beta(95%CI) for MR-RAPS | P for MR-RAPS | Beta(95%CI) for Egger | P for Egger | Intercept P for Egger | Label |
| Asp-Phe | 0.017(0.007-0.027) | 1.18e-03 | 0.005(-0.083-0.092) | 9.15e-01 | 8.08e-01 | Metabolite-SBP(BBJ) |
| Asp-Phe | 0.009(-0.005-0.024) | 2.16e-01 | -0.015(-0.145-0.115) | 8.18e-01 | 7.23e-01 | Metabolite-SBP(KoGES) |
| Asp-Phe | 0.025(0.013-0.037) | 7.13e-05 | -0.101(-0.224-0.022) | 1.09e-01 | 7.67e-02 | Metabolite-SBP(TWB) |
| Asp-Phe | 0.025(0.014-0.036) | 8.99e-06 | 0.056(-0.028-0.140) | 1.91e-01 | 4.60e-01 | Metabolite-DBP(BBJ) |
| Asp-Phe | 0.013(-0.002-0.028) | 8.60e-02 | -0.035(-0.150-0.080) | 5.52e-01 | 4.35e-01 | Metabolite-DBP(KoGES) |
| Asp-Phe | 0.025(0.012-0.037) | 1.54e-04 | -0.100(-0.254-0.055) | 2.06e-01 | 1.54e-01 | Metabolite-DBP(TWB) |
| Asp-Phe | 0.022(-0.036-0.079) | 4.60e-01 | 0.124(-0.366-0.614) | 6.20e-01 | 6.85e-01 | Metabolite-Hypertension(BBJ) |
| Asp-Phe | 0.036(-0.006-0.079) | 9.15e-02 | 0.046(-0.296-0.388) | 7.92e-01 | 9.46e-01 | Metabolite-Hypertension(KoGES) |
| L-Glutamic Acid | 0.007(-0.004-0.019) | 1.89e-01 | -0.089(-0.219-0.040) | 1.76e-01 | 1.87e-01 | Metabolite-SBP(BBJ) |
| L-Glutamic Acid | 0.009(-0.008-0.027) | 3.06e-01 | -0.067(-0.185-0.050) | 2.63e-01 | 2.40e-01 | Metabolite-SBP(KoGES) |
| L-Glutamic Acid | -0.005(-0.020-0.010) | 5.14e-01 | -0.007(-0.093-0.079) | 8.72e-01 | 9.62e-01 | Metabolite-SBP(TWB) |
| L-Glutamic Acid | 0.017(0.004-0.029) | 9.42e-03 | -0.057(-0.140-0.027) | 1.81e-01 | 1.28e-01 | Metabolite-DBP(BBJ) |
| L-Glutamic Acid | -0.004(-0.021-0.014) | 6.71e-01 | -0.089(-0.225-0.047) | 2.01e-01 | 2.53e-01 | Metabolite-DBP(KoGES) |
| L-Glutamic Acid | -0.001(-0.017-0.015) | 8.62e-01 | 0.015(-0.076-0.105) | 7.53e-01 | 7.37e-01 | Metabolite-DBP(TWB) |
| L-Glutamic Acid | -0.036(-0.104-0.032) | 3.00e-01 | -0.289(-0.682-0.104) | 1.50e-01 | 2.40e-01 | Metabolite-Hypertension(BBJ) |
| L-Glutamic Acid | -0.009(-0.059-0.041) | 7.25e-01 | -0.141(-0.427-0.145) | 3.34e-01 | 3.89e-01 | Metabolite-Hypertension(KoGES) |
| Nα-Acetyl-L-glutamine | 0.012(-0.007-0.031) | 2.27e-01 | 0.081(-0.073-0.235) | 3.02e-01 | 4.31e-01 | Metabolite-SBP(BBJ) |
| Nα-Acetyl-L-glutamine | 0.024(-0.001-0.049) | 5.92e-02 | 0.020(-0.082-0.123) | 7.02e-01 | 9.52e-01 | Metabolite-SBP(KoGES) |
| Nα-Acetyl-L-glutamine | -0.007(-0.028-0.014) | 5.02e-01 | 0.009(-0.086-0.104) | 8.51e-01 | 7.56e-01 | Metabolite-SBP(TWB) |
| Nα-Acetyl-L-glutamine | 0.007(-0.014-0.028) | 5.12e-01 | 0.069(-0.047-0.184) | 2.45e-01 | 3.66e-01 | Metabolite-DBP(BBJ) |
| Nα-Acetyl-L-glutamine | 0.013(-0.011-0.037) | 2.96e-01 | 0.035(-0.085-0.155) | 5.66e-01 | 7.25e-01 | Metabolite-DBP(KoGES) |
| Nα-Acetyl-L-glutamine | 0.012(-0.010-0.033) | 2.99e-01 | -0.015(-0.120-0.090) | 7.78e-01 | 6.48e-01 | Metabolite-DBP(TWB) |
| Nα-Acetyl-L-glutamine | 0.073(-0.042-0.188) | 2.12e-01 | 0.122(-0.513-0.756) | 7.07e-01 | 8.87e-01 | Metabolite-Hypertension(BBJ) |
| Nα-Acetyl-L-glutamine | -0.004(-0.072-0.064) | 9.06e-01 | -0.022(-0.265-0.220) | 8.56e-01 | 8.88e-01 | Metabolite-Hypertension(KoGES) |
| N-acetyl-D-Lactosamine | 0.004(-0.008-0.016) | 5.17e-01 | -0.018(-0.077-0.041) | 5.45e-01 | 4.80e-01 | Metabolite-SBP(BBJ) |
| N-acetyl-D-Lactosamine | 0.019(0.002-0.036) | 2.66e-02 | -0.030(-0.104-0.045) | 4.36e-01 | 2.35e-01 | Metabolite-SBP(KoGES) |
| N-acetyl-D-Lactosamine | 0.015(0.002-0.028) | 2.71e-02 | 0.053(-0.005-0.111) | 7.18e-02 | 2.11e-01 | Metabolite-SBP(TWB) |
| N-acetyl-D-Lactosamine | -0.001(-0.014-0.011) | 8.12e-01 | -0.068(-0.143-0.007) | 7.77e-02 | 1.22e-01 | Metabolite-DBP(BBJ) |
| N-acetyl-D-Lactosamine | 0.027(0.010-0.044) | 2.04e-03 | -0.024(-0.092-0.044) | 4.95e-01 | 1.76e-01 | Metabolite-DBP(KoGES) |
| N-acetyl-D-Lactosamine | 0.018(0.004-0.033) | 1.13e-02 | 0.048(-0.012-0.108) | 1.16e-01 | 3.35e-01 | Metabolite-DBP(TWB) |
| N-acetyl-D-Lactosamine | 0.025(-0.042-0.092) | 4.63e-01 | -0.095(-0.452-0.262) | 6.03e-01 | 5.28e-01 | Metabolite-Hypertension(BBJ) |
| N-acetyl-D-Lactosamine | 0.011(-0.037-0.058) | 6.61e-01 | 0.047(-0.143-0.237) | 6.30e-01 | 7.09e-01 | Metabolite-Hypertension(KoGES) |
| 13-Tetradecynoic acid | 0.002(-0.012-0.016) | 7.50e-01 | 0.018(-0.046-0.082) | 5.85e-01 | 6.59e-01 | Metabolite-SBP(BBJ) |
| 13-Tetradecynoic acid | 0.005(-0.017-0.027) | 6.67e-01 | 0.031(-0.074-0.136) | 5.63e-01 | 6.50e-01 | Metabolite-SBP(KoGES) |
| 13-Tetradecynoic acid | 0.020(0.001-0.040) | 4.16e-02 | 0.016(-0.079-0.111) | 7.38e-01 | 9.41e-01 | Metabolite-SBP(TWB) |
| 13-Tetradecynoic acid | 0.010(-0.005-0.024) | 2.03e-01 | 0.015(-0.080-0.111) | 7.51e-01 | 9.04e-01 | Metabolite-DBP(BBJ) |
| 13-Tetradecynoic acid | 0.010(-0.012-0.032) | 3.64e-01 | 0.051(-0.066-0.168) | 3.94e-01 | 5.34e-01 | Metabolite-DBP(KoGES) |
| 13-Tetradecynoic acid | 0.008(-0.013-0.028) | 4.64e-01 | 0.035(-0.065-0.135) | 4.95e-01 | 6.23e-01 | Metabolite-DBP(TWB) |
| 13-Tetradecynoic acid | -0.002(-0.084-0.079) | 9.54e-01 | -0.174(-0.541-0.194) | 3.54e-01 | 4.19e-01 | Metabolite-Hypertension(BBJ) |
| 13-Tetradecynoic acid | -0.036(-0.096-0.024) | 2.40e-01 | -0.300(-0.601-0.002) | 5.16e-02 | 1.77e-01 | Metabolite-Hypertension(KoGES) |
| Carnitine C16:1 | -0.007(-0.019-0.005) | 2.75e-01 | 0.001(-0.041-0.043) | 9.49e-01 | 7.21e-01 | Metabolite-SBP(BBJ) |
| Carnitine C16:1 | -0.005(-0.024-0.014) | 6.07e-01 | -0.011(-0.077-0.054) | 7.31e-01 | 8.49e-01 | Metabolite-SBP(KoGES) |
| Carnitine C16:1 | 0.021(0.003-0.040) | 2.46e-02 | -0.011(-0.087-0.064) | 7.66e-01 | 4.59e-01 | Metabolite-SBP(TWB) |
| Carnitine C16:1 | 0.000(-0.013-0.013) | 9.99e-01 | 0.013(-0.030-0.056) | 5.50e-01 | 5.68e-01 | Metabolite-DBP(BBJ) |
| Carnitine C16:1 | -0.006(-0.025-0.013) | 5.30e-01 | 0.001(-0.088-0.089) | 9.88e-01 | 8.88e-01 | Metabolite-DBP(KoGES) |
| Carnitine C16:1 | 0.014(-0.005-0.033) | 1.40e-01 | -0.026(-0.112-0.060) | 5.54e-01 | 4.23e-01 | Metabolite-DBP(TWB) |
| Carnitine C16:1 | 0.004(-0.065-0.073) | 9.09e-01 | -0.060(-0.325-0.204) | 6.55e-01 | 6.47e-01 | Metabolite-Hypertension(BBJ) |
| Carnitine C16:1 | -0.002(-0.054-0.050) | 9.45e-01 | -0.088(-0.348-0.172) | 5.07e-01 | 5.35e-01 | Metabolite-Hypertension(KoGES) |
| Cortisol | 0.025(0.009-0.041) | 2.71e-03 | -0.035(-0.176-0.106) | 6.26e-01 | 4.69e-01 | Metabolite-SBP(BBJ) |
| Cortisol | 0.010(-0.018-0.037) | 4.92e-01 | 0.013(-0.175-0.202) | 8.90e-01 | 9.71e-01 | Metabolite-SBP(KoGES) |
| Cortisol | -0.022(-0.041--0.003) | 2.07e-02 | -0.148(-0.316-0.020) | 8.34e-02 | 2.29e-01 | Metabolite-SBP(TWB) |
| Cortisol | 0.009(-0.007-0.026) | 2.67e-01 | 0.005(-0.113-0.123) | 9.36e-01 | 9.44e-01 | Metabolite-DBP(BBJ) |
| Cortisol | -0.006(-0.033-0.020) | 6.40e-01 | -0.097(-0.414-0.220) | 5.49e-01 | 6.26e-01 | Metabolite-DBP(KoGES) |
| Cortisol | -0.020(-0.040-0.000) | 5.16e-02 | -0.146(-0.279--0.013) | 3.18e-02 | 1.57e-01 | Metabolite-DBP(TWB) |
| Cortisol | 0.057(-0.034-0.148) | 2.20e-01 | -0.255(-0.903-0.392) | 4.40e-01 | 4.12e-01 | Metabolite-Hypertension(BBJ) |
| Cortisol | -0.020(-0.096-0.056) | 6.01e-01 | 0.324(-0.386-1.033) | 3.71e-01 | 4.39e-01 | Metabolite-Hypertension(KoGES) |
| prostaglandin E3 | -0.014(-0.027-0.000) | 5.27e-02 | -0.041(-0.156-0.073) | 4.76e-01 | 6.51e-01 | Metabolite-SBP(BBJ) |
| prostaglandin E3 | 0.023(0.003-0.043) | 2.11e-02 | -0.043(-0.141-0.054) | 3.82e-01 | 2.49e-01 | Metabolite-SBP(KoGES) |
| prostaglandin E3 | 0.011(-0.005-0.027) | 1.94e-01 | -0.046(-0.127-0.035) | 2.65e-01 | 2.23e-01 | Metabolite-SBP(TWB) |
| prostaglandin E3 | -0.019(-0.034--0.005) | 8.36e-03 | -0.076(-0.208-0.057) | 2.62e-01 | 4.49e-01 | Metabolite-DBP(BBJ) |
| prostaglandin E3 | 0.025(0.005-0.045) | 1.38e-02 | -0.017(-0.116-0.083) | 7.41e-01 | 4.57e-01 | Metabolite-DBP(KoGES) |
| prostaglandin E3 | 0.001(-0.015-0.018) | 8.81e-01 | -0.114(-0.198--0.029) | 8.21e-03 | 4.17e-02 | Metabolite-DBP(TWB) |
| prostaglandin E3 | -0.023(-0.102-0.057) | 5.73e-01 | 0.020(-0.414-0.454) | 9.28e-01 | 8.57e-01 | Metabolite-Hypertension(BBJ) |
| prostaglandin E3 | -0.009(-0.061-0.042) | 7.23e-01 | -0.155(-0.532-0.222) | 4.20e-01 | 4.79e-01 | Metabolite-Hypertension(KoGES) |
| Creatine phosphate | 0.008(-0.005-0.021) | 2.35e-01 | -0.023(-0.074-0.029) | 3.87e-01 | 2.97e-01 | Metabolite-SBP(BBJ) |
| Creatine phosphate | 0.003(-0.016-0.022) | 7.59e-01 | -0.042(-0.125-0.041) | 3.20e-01 | 3.34e-01 | Metabolite-SBP(KoGES) |
| Creatine phosphate | 0.008(-0.008-0.025) | 3.30e-01 | 0.047(-0.013-0.106) | 1.24e-01 | 2.58e-01 | Metabolite-SBP(TWB) |
| Creatine phosphate | 0.016(0.002-0.030) | 2.49e-02 | 0.011(-0.042-0.063) | 6.92e-01 | 8.36e-01 | Metabolite-DBP(BBJ) |
| Creatine phosphate | -0.006(-0.025-0.013) | 5.39e-01 | -0.041(-0.122-0.040) | 3.18e-01 | 4.26e-01 | Metabolite-DBP(KoGES) |
| Creatine phosphate | 0.018(0.001-0.036) | 4.25e-02 | 0.036(-0.062-0.134) | 4.69e-01 | 7.09e-01 | Metabolite-DBP(TWB) |
| Creatine phosphate | 0.043(-0.033-0.119) | 2.68e-01 | -0.080(-0.366-0.205) | 5.81e-01 | 4.35e-01 | Metabolite-Hypertension(BBJ) |
| Creatine phosphate | -0.005(-0.059-0.050) | 8.68e-01 | -0.017(-0.224-0.190) | 8.71e-01 | 9.08e-01 | Metabolite-Hypertension(KoGES) |
| SPH(d18:1) | 0.006(-0.005-0.018) | 2.80e-01 | -0.032(-0.081-0.018) | 2.14e-01 | 1.87e-01 | Metabolite-SBP(BBJ) |
| SPH(d18:1) | -0.007(-0.025-0.010) | 4.05e-01 | 0.012(-0.060-0.083) | 7.50e-01 | 6.14e-01 | Metabolite-SBP(KoGES) |
| SPH(d18:1) | -0.004(-0.019-0.011) | 6.06e-01 | -0.017(-0.078-0.044) | 5.88e-01 | 6.82e-01 | Metabolite-SBP(TWB) |
| SPH(d18:1) | 0.013(0.000-0.025) | 4.50e-02 | -0.002(-0.054-0.050) | 9.53e-01 | 6.10e-01 | Metabolite-DBP(BBJ) |
| SPH(d18:1) | -0.014(-0.032-0.003) | 1.05e-01 | 0.009(-0.062-0.081) | 7.98e-01 | 5.35e-01 | Metabolite-DBP(KoGES) |
| SPH(d18:1) | -0.010(-0.026-0.005) | 1.86e-01 | -0.016(-0.080-0.049) | 6.33e-01 | 8.71e-01 | Metabolite-DBP(TWB) |
| SPH(d18:1) | -0.000(-0.066-0.066) | 9.94e-01 | -0.019(-0.412-0.374) | 9.25e-01 | 9.28e-01 | Metabolite-Hypertension(BBJ) |
| SPH(d18:1) | -0.035(-0.084-0.014) | 1.60e-01 | -0.014(-0.214-0.186) | 8.91e-01 | 8.43e-01 | Metabolite-Hypertension(KoGES) |

| **Table S13. Potential causal relationships of blood pressure with metabolites in MR-RAP and Egger analysis in Liuheng** | | | | | | |
| --- | --- | --- | --- | --- | --- | --- |
| Compound | Beta(95%CI) for MR-RAPS | P for MR-RAPS | Beta(95%CI) for Egger | P for Egger | Intercept P for Egger | Label |
| N-acetyl-D-Lactosamine | 0.200(-0.221-0.621) | 3.52e-01 | 0.907(-0.328-2.143) | 1.50e-01 | 2.33e-01 | SBP-Metabolite(BBJ) |
| N-acetyl-D-Lactosamine | 0.219(-0.119-0.558) | 2.04e-01 | 1.099(0.054-2.144) | 3.92e-02 | 7.99e-02 | SBP-Metabolite(KoGES) |
| N-acetyl-D-Lactosamine | 0.128(-0.205-0.460) | 4.51e-01 | 0.835(-0.294-1.964) | 1.47e-01 | 2.00e-01 | SBP-Metabolite(TWB) |
| N-acetyl-D-Lactosamine | 0.286(-0.189-0.761) | 2.38e-01 | 1.940(0.514-3.365) | 7.67e-03 | 1.72e-02 | DBP-Metabolite(BBJ) |
| N-acetyl-D-Lactosamine | 0.484(0.111-0.858) | 1.11e-02 | 1.206(0.127-2.286) | 2.85e-02 | 1.61e-01 | DBP-Metabolite(KoGES) |
| N-acetyl-D-Lactosamine | 0.224(-0.117-0.564) | 1.97e-01 | 0.551(-0.588-1.690) | 3.43e-01 | 5.49e-01 | DBP-Metabolite(TWB) |
| N-acetyl-D-Lactosamine | 0.320(0.103-0.536) | 3.84e-03 | 0.338(-0.245-0.922) | 2.56e-01 | 9.29e-01 | Hypertension-Metabolite(BBJ) |
| N-acetyl-D-Lactosamine | 0.059(-0.064-0.182) | 3.49e-01 | 0.375(0.017-0.734) | 4.02e-02 | 6.92e-02 | Hypertension-Metabolite(KoGES) |
| Cortisol | 0.258(-0.159-0.675) | 2.26e-01 | 0.485(-0.769-1.739) | 4.48e-01 | 6.97e-01 | SBP-Metabolite(BBJ) |
| Cortisol | 0.560(0.222-0.897) | 1.15e-03 | 0.100(-0.873-1.073) | 8.40e-01 | 3.47e-01 | SBP-Metabolite(KoGES) |
| Cortisol | 0.277(-0.051-0.605) | 9.82e-02 | 0.412(-0.788-1.611) | 5.01e-01 | 8.05e-01 | SBP-Metabolite(TWB) |
| Cortisol | 0.299(-0.171-0.768) | 2.13e-01 | 0.044(-1.513-1.601) | 9.56e-01 | 7.47e-01 | DBP-Metabolite(BBJ) |
| Cortisol | 0.404(0.035-0.773) | 3.19e-02 | 0.447(-0.727-1.620) | 4.55e-01 | 9.19e-01 | DBP-Metabolite(KoGES) |
| Cortisol | 0.124(-0.212-0.461) | 4.69e-01 | 0.057(-1.103-1.217) | 9.24e-01 | 9.11e-01 | DBP-Metabolite(TWB) |
| Cortisol | -0.006(-0.216-0.203) | 9.53e-01 | -0.501(-1.078-0.076) | 8.86e-02 | 9.32e-02 | Hypertension-Metabolite(BBJ) |
| Cortisol | 0.078(-0.043-0.199) | 2.08e-01 | 0.336(-0.033-0.705) | 7.39e-02 | 1.46e-01 | Hypertension-Metabolite(KoGES) |

| **Table S14. Potential causal relationships of metabolites ratios with blood pressure in MR-RAP and Egger analysis in Liuheng** | | | | | | |
| --- | --- | --- | --- | --- | --- | --- |
| Ratio | Beta(95%CI) for MR-RAPS | P for MR-RAPS | Beta(95%CI) for Egger | P for Egger | Intercept P for Egger | Label |
| Creatine / Phosphocreatine | -0.009(-0.021-0.003) | 1.46e-01 | -0.055(-0.125-0.015) | 1.25e-01 | 2.34e-01 | Ratio-SBP(BBJ) |
| Creatine / Phosphocreatine | 0.003(-0.012-0.017) | 7.28e-01 | 0.084(0.014-0.153) | 1.79e-02 | 4.08e-02 | Ratio-SBP(KoGES) |
| Creatine / Phosphocreatine | -0.005(-0.018-0.008) | 4.43e-01 | -0.025(-0.073-0.023) | 3.02e-01 | 4.13e-01 | Ratio-SBP(TWB) |
| Creatine / Phosphocreatine | -0.018(-0.031--0.005) | 5.85e-03 | -0.058(-0.133-0.018) | 1.35e-01 | 3.25e-01 | Ratio-DBP(BBJ) |
| Creatine / Phosphocreatine | -0.002(-0.017-0.012) | 7.52e-01 | 0.058(0.003-0.112) | 3.82e-02 | 5.26e-02 | Ratio-DBP(KoGES) |
| Creatine / Phosphocreatine | -0.006(-0.019-0.008) | 4.17e-01 | -0.023(-0.085-0.038) | 4.58e-01 | 5.66e-01 | Ratio-DBP(TWB) |
| Creatine / Phosphocreatine | 0.014(-0.057-0.085) | 6.92e-01 | 0.076(-0.265-0.416) | 6.64e-01 | 7.30e-01 | Ratio-Hypertension(BBJ) |
| Creatine / Phosphocreatine | -0.012(-0.053-0.028) | 5.50e-01 | 0.132(-0.023-0.286) | 9.43e-02 | 9.16e-02 | Ratio-Hypertension(KoGES) |
| Cortisol / Cortisone | 0.020(0.005-0.036) | 1.11e-02 | 0.021(-0.074-0.115) | 6.67e-01 | 9.84e-01 | Ratio-SBP(BBJ) |
| Cortisol / Cortisone | 0.001(-0.021-0.023) | 9.11e-01 | -0.107(-0.218-0.004) | 5.95e-02 | 1.24e-01 | Ratio-SBP(KoGES) |
| Cortisol / Cortisone | 0.010(-0.009-0.029) | 2.91e-01 | 0.087(-0.008-0.182) | 7.29e-02 | 1.80e-01 | Ratio-SBP(TWB) |
| Cortisol / Cortisone | 0.010(-0.006-0.027) | 2.03e-01 | 0.025(-0.065-0.114) | 5.88e-01 | 7.70e-01 | Ratio-DBP(BBJ) |
| Cortisol / Cortisone | -0.002(-0.023-0.020) | 8.67e-01 | -0.162(-0.274--0.050) | 4.49e-03 | 4.58e-02 | Ratio-DBP(KoGES) |
| Cortisol / Cortisone | 0.002(-0.018-0.021) | 8.67e-01 | -0.048(-0.194-0.097) | 5.15e-01 | 5.32e-01 | Ratio-DBP(TWB) |
| Cortisol / Cortisone | -0.015(-0.100-0.070) | 7.25e-01 | 0.286(-0.478-1.049) | 4.64e-01 | 4.92e-01 | Ratio-Hypertension(BBJ) |
| Cortisol / Cortisone | -0.030(-0.092-0.032) | 3.39e-01 | 0.114(-0.298-0.527) | 5.87e-01 | 5.29e-01 | Ratio-Hypertension(KoGES) |

| **Table S15. Potential causal relationships of metabolites with blood pressure in MR-RAPS and Egger analysis in THSBC** | | | | | | |
| --- | --- | --- | --- | --- | --- | --- |
| Compound | Beta(95%CI) for MR-RAPS | P for MR-RAPS | Beta(95%CI) for Egger | P for Egger | Intercept P for Egger | Label |
| Asp-Phe | 0.012(-0.002-0.026) | 8.83E-02 | -0.005(-0.058-0.047) | 8.39E-01 | 5.53E-01 | Metabolite-SBP(BBJ) |
| Asp-Phe | 0.016(-0.006-0.037) | 1.59E-01 | -0.065(-0.154-0.024) | 1.53E-01 | 1.70E-01 | Metabolite-SBP(KoGES) |
| Asp-Phe | -0.009(-0.027-0.010) | 3.69E-01 | -0.011(-0.091-0.070) | 7.97E-01 | 9.62E-01 | Metabolite-SBP(TWB) |
| Asp-Phe | 0.020(0.006-0.035) | 6.14E-03 | 0.040(-0.024-0.105) | 2.17E-01 | 5.60E-01 | Metabolite-DBP(BBJ) |
| Asp-Phe | 0.016(-0.006-0.037) | 1.47E-01 | -0.041(-0.172-0.089) | 5.33E-01 | 4.49E-01 | Metabolite-DBP(KoGES) |
| Asp-Phe | 0.008(-0.012-0.027) | 4.40E-01 | 0.033(-0.072-0.138) | 5.40E-01 | 6.60E-01 | Metabolite-DBP(TWB) |
| Asp-Phe | 0.052(-0.026-0.131) | 1.90E-01 | 0.220(-0.081-0.521) | 1.52E-01 | 3.39E-01 | Metabolite-Hypertension(BBJ) |
| Asp-Phe | 0.067(0.005-0.129) | 3.39E-02 | -0.069(-0.317-0.179) | 5.86E-01 | 3.57E-01 | Metabolite-Hypertension(KoGES) |
| N-acetyl-D-Lactosamine | -0.001(-0.013-0.011) | 8.55E-01 | -0.045(-0.101-0.012) | 1.19E-01 | 1.96E-01 | Metabolite-SBP(BBJ) |
| N-acetyl-D-Lactosamine | -0.004(-0.024-0.017) | 7.24E-01 | -0.015(-0.155-0.124) | 8.30E-01 | 8.78E-01 | Metabolite-SBP(KoGES) |
| N-acetyl-D-Lactosamine | 0.005(-0.012-0.022) | 5.51E-01 | 0.072(-0.043-0.187) | 2.20E-01 | 3.29E-01 | Metabolite-SBP(TWB) |
| N-acetyl-D-Lactosamine | 0.003(-0.010-0.015) | 6.94E-01 | -0.031(-0.090-0.028) | 2.99E-01 | 3.15E-01 | Metabolite-DBP(BBJ) |
| N-acetyl-D-Lactosamine | 0.011(-0.009-0.031) | 2.88E-01 | 0.010(-0.150-0.170) | 9.04E-01 | 9.94E-01 | Metabolite-DBP(KoGES) |
| N-acetyl-D-Lactosamine | 0.007(-0.011-0.025) | 4.58E-01 | 0.111(0.017-0.205) | 2.11E-02 | 1.14E-01 | Metabolite-DBP(TWB) |
| N-acetyl-D-Lactosamine | 0.071(-0.000-0.142) | 5.02E-02 | 0.072(-0.250-0.395) | 6.61E-01 | 9.84E-01 | Metabolite-Hypertension(BBJ) |
| N-acetyl-D-Lactosamine | 0.021(-0.035-0.078) | 4.56E-01 | -0.071(-0.421-0.278) | 6.89E-01 | 6.37E-01 | Metabolite-Hypertension(KoGES) |
| Cortisol | -0.007(-0.022-0.007) | 3.36E-01 | 0.009(-0.050-0.069) | 7.62E-01 | 6.38E-01 | Metabolite-SBP(BBJ) |
| Cortisol | -0.016(-0.037-0.006) | 1.57E-01 | -0.007(-0.112-0.097) | 8.88E-01 | 9.00E-01 | Metabolite-SBP(KoGES) |
| Cortisol | -0.002(-0.020-0.016) | 8.36E-01 | -0.015(-0.090-0.061) | 7.01E-01 | 7.63E-01 | Metabolite-SBP(TWB) |
| Cortisol | 0.003(-0.012-0.018) | 6.92E-01 | 0.029(-0.033-0.091) | 3.63E-01 | 4.90E-01 | Metabolite-DBP(BBJ) |
| Cortisol | -0.013(-0.034-0.008) | 2.39E-01 | -0.024(-0.158-0.110) | 7.26E-01 | 8.73E-01 | Metabolite-DBP(KoGES) |
| Cortisol | -0.010(-0.029-0.009) | 3.12E-01 | -0.006(-0.085-0.074) | 8.87E-01 | 9.30E-01 | Metabolite-DBP(TWB) |
| Cortisol | -0.003(-0.084-0.079) | 9.49E-01 | 0.039(-0.301-0.379) | 8.24E-01 | 8.30E-01 | Metabolite-Hypertension(BBJ) |
| Cortisol | -0.028(-0.086-0.030) | 3.47E-01 | -0.221(-0.579-0.137) | 2.27E-01 | 3.87E-01 | Metabolite-Hypertension(KoGES) |
| Creatine phosphate | -0.009(-0.025-0.007) | 2.66E-01 | -0.034(-0.373-0.305) | 8.45E-01 | 8.96E-01 | Metabolite-SBP(BBJ) |
| Creatine phosphate | -0.006(-0.030-0.017) | 5.96E-01 | -0.300(-0.549--0.051) | 1.81E-02 | 1.46E-01 | Metabolite-SBP(KoGES) |
| Creatine phosphate | 0.014(-0.005-0.033) | 1.60E-01 | 0.095(-0.390-0.581) | 7.00E-01 | 7.67E-01 | Metabolite-SBP(TWB) |
| Creatine phosphate | -0.002(-0.019-0.015) | 8.26E-01 | -0.062(-0.264-0.139) | 5.44E-01 | 6.15E-01 | Metabolite-DBP(BBJ) |
| Creatine phosphate | 0.007(-0.016-0.031) | 5.39E-01 | -0.322(-0.572--0.073) | 1.13E-02 | 1.22E-01 | Metabolite-DBP(KoGES) |
| Creatine phosphate | 0.010(-0.010-0.030) | 3.45E-01 | -0.024(-0.484-0.436) | 9.19E-01 | 9.01E-01 | Metabolite-DBP(TWB) |
| Creatine phosphate | 0.092(-0.002-0.185) | 5.49E-02 | -0.247(-1.513-1.019) | 7.02E-01 | 6.54E-01 | Metabolite-Hypertension(BBJ) |
| Creatine phosphate | -0.036(-0.104-0.031) | 2.94E-01 | -0.524(-1.226-0.179) | 1.44E-01 | 3.05E-01 | Metabolite-Hypertension(KoGES) |

| **Table S16. Summary for all identified independent metaboQTLs** | | | | | | | | | | |
| --- | --- | --- | --- | --- | --- | --- | --- | --- | --- | --- |
| SNP | Compound | Class.I | CHR | BP | A1 | A2 | Freq | Beta | Se | P |
| rs7394579 | FFA(18:4) | FA | 11 | 61581450 | G | A | 0.44 | -0.29 | 0.04 | 3.54E-12 |
| rs4767939 | LPC(0:0/14:0) | GP | 12 | 112206895 | A | G | 0.43 | 0.25 | 0.04 | 1.40E-09 |
| rs116852776 | Creatine phosphate | Nucleotide and Its metabolites | 2 | 73856064 | T | C | 0.09 | -0.42 | 0.07 | 5.48E-09 |
| rs76309274 | Cortisone | Hormones and hormone related compounds | 9 | 71743290 | A | G | 0.13 | -0.35 | 0.06 | 2.20E-08 |
| rs11204914 | 11-dehydro-TXB3 | Hormones and hormone related compounds | 1 | 151881093 | G | A | 0.37 | -0.28 | 0.04 | 2.96E-10 |
| rs7542137 | 13-Tetradecynoic acid | FA | 1 | 151868892 | C | T | 0.39 | -0.28 | 0.04 | 8.00E-11 |
| rs4767939 | Glycerophospho-N-Palmitoyl Ethanolamine | GP | 12 | 112206895 | A | G | 0.43 | 0.24 | 0.04 | 1.51E-08 |
| rs174529 | prostaglandin E3 | Hormones and hormone related compounds | 11 | 61543961 | C | T | 0.46 | -0.31 | 0.04 | 7.40E-14 |
| rs6747843 | 6,6'-(1,2-phenylene)bis(1,3,5-triazine-2,4-diamine) | Benzene and substituted derivatives | 2 | 234664354 | A | G | 0.13 | 0.35 | 0.06 | 1.59E-08 |
| rs4149056 | 11-Deoxyprostaglandin F1alpha | Hormones and hormone related compounds | 12 | 21331549 | C | T | 0.12 | 0.41 | 0.07 | 6.97E-10 |
| notes: FA, fatty acids; GP, glycerophospholipids. | |  |  |  |  |  |  |  |  |  |

| **Table S17. The metaboQTLs overlapping with liver and eQTLs from the GTEx project** | | | | | |
| --- | --- | --- | --- | --- | --- |
| SNP | Gencode Id | Gene Symbol | P-Value | NES | Tissue |
| rs174529 | ENSG00000134824.13 | FADS2 | 4.90E-41 | 0.62 | Whole Blood |
| rs174529 | ENSG00000149485.18 | FADS1 | 1.10E-05 | 0.17 | Whole Blood |
| rs174529 | ENSG00000149485.18 | FADS1 | 4.40E-05 | -0.33 | Liver |
| rs4767939 | ENSG00000111275.13 | ALDH2 | 8.80E-17 | -0.24 | Artery - Aorta |
| rs4767939 | ENSG00000111275.13 | ALDH2 | 2.70E-15 | -0.20 | Artery - Tibial |
| rs4767939 | ENSG00000111275.13 | ALDH2 | 3.00E-06 | -0.21 | Heart - Atrial Appendage |
| rs4767939 | ENSG00000111275.13 | ALDH2 | 1.30E-04 | -0.10 | Heart - Left Ventricle |
| notes: NES, normalized effect size. | |  |  |  |  |

| **Table S18. The metaboQTLs overlapping with serum pQTLs identified in the UK Biobank** | | | | | | | | |
| --- | --- | --- | --- | --- | --- | --- | --- | --- |
| SNP | UKBPPP.ProteinID | Assay.Target | Target.UniProt | A1FREQ | BETA | SE | log10(p) | cis/trans |
| rs4149056 | MBL2:P11226:OID30759:v1 | MBL2 | P11226 | 0.15 | -0.06 | 0.01 | 24.29 | trans |
| rs4149056 | SHBG:P04278:OID30685:v1 | SHBG | P04278 | 0.15 | -0.07 | 0.01 | 17.55 | trans |

| **Table S19. Significant associations between metabolites and lifestyles** | | | | |
| --- | --- | --- | --- | --- |
| Lifestyle | Metabolites | Beta(95%CI) | P | FDR adjusted P |
| WC | L-Glutamic Acid | 0.43(0.38-0.48) | 2.28e-54 | 2.28e-53 |
| BMI | L-Glutamic Acid | 0.39(0.34-0.44) | 1.93e-46 | 1.93e-45 |
| Healthy lifestyle score | L-Glutamic Acid | -0.28(-0.35--0.21) | 1.89e-15 | 1.89e-14 |
| WC | Asp-Phe | 0.14(0.08-0.19) | 2.61e-06 | 4.35e-06 |
| BMI | Asp-Phe | 0.11(0.05-0.16) | 2.35e-04 | 3.91e-04 |
| WC | Nα-Acetyl-L-glutamine | 0.18(0.12-0.24) | 9.62e-10 | 4.81e-09 |
| Healthy lifestyle score | Nα-Acetyl-L-glutamine | -0.10(-0.17--0.02) | 8.04e-03 | 4.02e-02 |
| BMI | Nα-Acetyl-L-glutamine | 0.18(0.13-0.24) | 9.64e-11 | 4.82e-10 |
| BMI | N-acetyl-D-Lactosamine | 0.12(0.06-0.18) | 4.50e-05 | 9.00e-05 |
| WC | N-acetyl-D-Lactosamine | 0.14(0.08-0.20) | 1.89e-06 | 3.78e-06 |
| BMI | 13-Tetradecynoic acid | -0.07(-0.13--0.02) | 1.17e-02 | 1.67e-02 |
| WC | 13-Tetradecynoic acid | -0.09(-0.15--0.03) | 2.24e-03 | 3.20e-03 |
| WC | Cortisol | -0.16(-0.22--0.11) | 3.67e-08 | 1.22e-07 |
| BMI | Cortisol | -0.18(-0.23--0.12) | 9.47e-10 | 3.16e-09 |
| BMI | SPH(d18:1) | 0.14(0.08-0.19) | 2.43e-06 | 6.08e-06 |
| WC | SPH(d18:1) | 0.14(0.09-0.20) | 1.29e-06 | 3.22e-06 |

| **Table S20. Significant associations between lifestyles and blood pressure** | | | | |
| --- | --- | --- | --- | --- |
| Lifestyle | Blood pressure | Beta(95%CI) | P | FDR adjusted P |
| aquatic_products_seafood_scaled | SBP | -0.62(-1.92-0.68) | 3.47e-01 | 6.36e-01 |
| BMI | SBP | 3.92(2.81-5.04) | 1.07e-11 | 6.52e-11 |
| drink_2015 | SBP | 1.05(-2.86-4.96) | 5.99e-01 | 7.32e-01 |
| fruit_scaled | SBP | -0.85(-2.09-0.38) | 1.74e-01 | 4.07e-01 |
| HLS | SBP | -1.91(-3.37--0.45) | 1.04e-02 | 3.82e-02 |
| pa_met_scaled | SBP | 0.77(-0.37-1.92) | 1.85e-01 | 4.07e-01 |
| red_meat_scaled | SBP | 0.45(-0.89-1.78) | 5.10e-01 | 7.02e-01 |
| sleep_scaled | SBP | -0.47(-1.66-0.72) | 4.35e-01 | 6.83e-01 |
| smoke_2015 | SBP | -0.50(-5.01-4.01) | 8.28e-01 | 8.28e-01 |
| vegetable_scaled | SBP | -0.22(-1.42-0.98) | 7.17e-01 | 7.88e-01 |
| WC | SBP | 4.03(2.88-5.18) | 1.19e-11 | 6.52e-11 |
| aquatic_products_seafood_scaled | DBP | -0.02(-0.80-0.77) | 9.62e-01 | 9.97e-01 |
| BMI | DBP | 3.76(3.11-4.41) | 5.28e-28 | 2.91e-27 |
| drink_2015 | DBP | -0.12(-2.48-2.25) | 9.23e-01 | 9.97e-01 |
| fruit_scaled | DBP | -0.00(-0.75-0.74) | 9.97e-01 | 9.97e-01 |
| HLS | DBP | -1.94(-2.82--1.06) | 1.74e-05 | 6.39e-05 |
| pa_met_scaled | DBP | -0.15(-0.84-0.55) | 6.77e-01 | 9.97e-01 |
| red_meat_scaled | DBP | 0.47(-0.34-1.27) | 2.55e-01 | 7.01e-01 |
| sleep_scaled | DBP | -0.00(-0.72-0.71) | 9.91e-01 | 9.97e-01 |
| smoke_2015 | DBP | -0.06(-2.78-2.66) | 9.65e-01 | 9.97e-01 |
| vegetable_scaled | DBP | -0.27(-0.99-0.45) | 4.66e-01 | 9.97e-01 |
| WC | DBP | 3.92(3.25-4.59) | 1.06e-28 | 1.17e-27 |

| **Table S21. Mediation effects of metabolites in associations between lifestyle and blood pressure** | | | | | | | | | |
| --- | --- | --- | --- | --- | --- | --- | --- | --- | --- |
| Lifestyle | Compound | Blood pressure | Beta(95%CI) for total effect | P for total effect | Beta(95%CI) for direct effect | P for direct effect | Beta(95%CI) for indirect effect | P for indirect effect | Mediation proportion(95%CI) |
| BMI | L-Glutamic Acid | DBP | 3.68(3.03-4.41) | P<0.0001 | 3.20(2.47-3.99) | P<0.0001 | 0.48(0.18-0.78) | 0.002 | 0.1295(0.0474-0.2241) |
| Healthy lifestyle score | L-Glutamic Acid | DBP | -1.91(-2.83--0.97) | P<0.0001 | -1.31(-2.19--0.43) | 0.008 | -0.60(-0.86--0.36) | P<0.0001 | 0.3153(0.1746-0.6023) |
| WC | L-Glutamic Acid | DBP | 3.82(3.13-4.45) | P<0.0001 | 3.36(2.59-4.07) | P<0.0001 | 0.46(0.16-0.78) | 0.002 | 0.1216(0.0418-0.2132) |
| BMI | Nα-Acetyl-L-glutamine | SBP | 3.84(2.72-4.99) | P<0.0001 | 3.45(2.36-4.59) | P<0.0001 | 0.38(0.17-0.66) | P<0.0001 | 0.1003(0.0439-0.1735) |
| WC | Nα-Acetyl-L-glutamine | SBP | 3.91(2.73-5.03) | P<0.0001 | 3.53(2.32-4.64) | P<0.0001 | 0.38(0.16-0.63) | P<0.0001 | 0.0967(0.0413-0.1874) |
| BMI | N-acetyl-D-Lactosamine | SBP | 3.84(2.72-4.99) | P<0.0001 | 3.59(2.46-4.75) | P<0.0001 | 0.25(0.08-0.48) | 0.004 | 0.0648(0.0181-0.1273) |
| WC | N-acetyl-D-Lactosamine | SBP | 3.91(2.73-5.03) | P<0.0001 | 3.63(2.41-4.74) | P<0.0001 | 0.28(0.09-0.50) | P<0.0001 | 0.0725(0.0242-0.1369) |
